# Reversible C–C bond formation in group 4 metal complexes: nitrile extrusion *via* β-aryl elimination†

**DOI:** 10.1039/d4sc02173h

**Published:** 2024-08-27

**Authors:** Pavel S. Kulyabin, Georgy P. Goryunov, Andrei N. Iashin, Dmitry Y. Mladentsev, Dmitry V. Uborsky, Christian Ehm, Jo Ann M. Canich, John R. Hagadorn, Alexander Z. Voskoboynikov

**Affiliations:** a Department of Chemistry, M. V. Lomonosov Moscow State University Leninskie Gory, 1/3 Moscow 119991 Russian Federation; b Dipartimento di Scienze Chimiche, Università di Napoli Federico II Via Cintia Napoli 80126 Italy; c Baytown Technology and Engineering Complex, ExxonMobil Technology and Engineering Company Baytown Texas 77520 USA

## Abstract

Pyridylamides of zirconium and hafnium with [C,N,N]-ligands reversibly insert nitriles into M–C_Ar_ bonds leading to an observable equilibrium between the starting [C,N,N]-complexes and newly formed [N,N,N]-complexes with a ketimide moiety in a 7-membered metallacycle. The discovered reversible insertion of nitriles into M–C_Ar_ bonds represents an unprecedented example of β-aryl elimination from a ketimide ligand in early transition metal complexes. Experimental and computational studies suggest thermodynamic and electronic reasons for this reactivity. Weak orbital overlap between the ketimide nitrogen and the metal, and an unfavorable 7-membered metallacycle destabilize the product of insertion into the M–C_Ar_ bond, while the pyridylamide moiety acts as a directing group making the reverse process viable. The influence of non-chelate spectator ligands on the metal center and substituents in nitrile on the thermodynamic stability of the [N,N,N]-complexes was also studied. Exploiting β-carbon elimination in complexes of early transition metals may extend the range of catalysts that are accessible for C–C activation processes in the future.

## Abstract 2

Zr and Hf pyridylamides reversibly insert nitriles into M–C_Ar_ bond and release them back with cleavage of the formed C–C bond, regeneration of the M–C_Ar_ bond and the free nitrile: a first example of β-aryl elimination in early transition metals.

## Graphical abstract

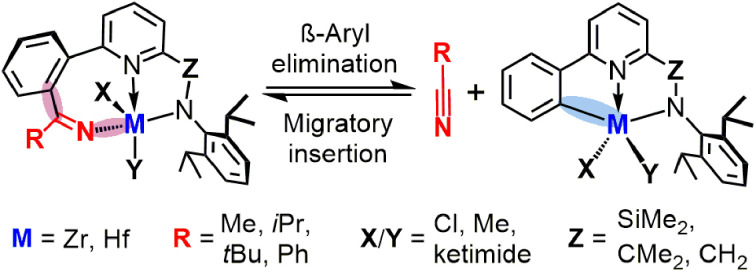

## Introduction

**Fig. 1:**
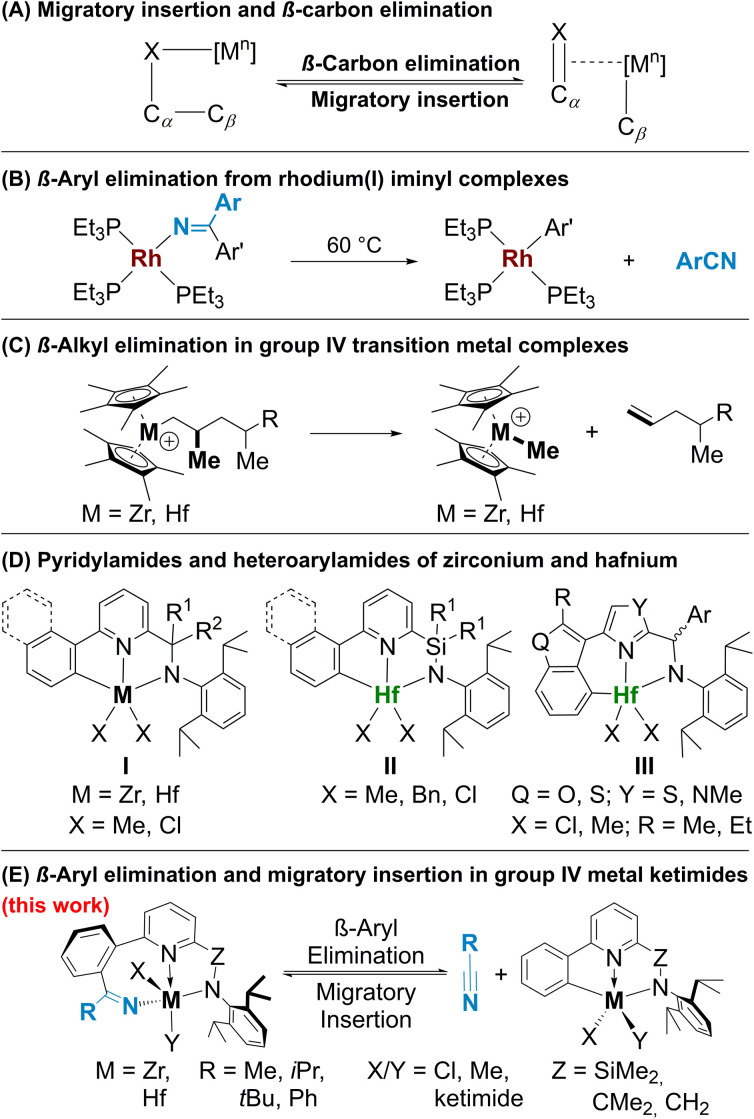
β-Carbon elimination and migratory insertion in complexes of zirconium and hafnium.

Over the recent decades, the cleavage of C–C bonds has garnered significant attention due to its wide-ranging applications in catalytic transformations,^1–6^ including highly sought after chemical methods for polyolefin degradation.^7^ Since the first seminal reports^8–11^ this area of research has witnessed substantial advancements and continues to be a subject of immense interest in the scientific community. One of the main strategies for transition metal catalysed C–C bond activation is β-carbon elimination,^4,12,13^ the microscopic reverse of migratory insertion (Fig. 1A; X = C, heteroatom). In terms of mechanisms, the process of β-carbon elimination is akin to that of β-hydride elimination, though it is less explored due to the challenges in designing systems for its study.^12^ Generally, migratory insertion is thermodynamically favoured; reversing this transformation requires an additional driving force: (a) formation of stronger C_sp_–M, C_sp_^2^–M^14,15^ or C
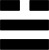
X bonds,^16^ (b) release of ring strain,^17^ and (c) relief of steric strain.^2,3^ β-Carbon elimination processes do not require a change in metal oxidation states and are known for early transition metals.^18^

Since the first reports of β-aryl elimination employing palladium and rhodium alkoxy and ketimide complexes by Uemura,^19,20^ Miura^21^ and Hartwig^16,22,23^ (Fig. 1B) in the 2000s, this reaction has gained importance for catalytic C–C bond activation.^4,24^ Presently, this type of reactivity has been demonstrated for alcoholates of Mn, Pd, Rh, Co, Ni, Cu,^4^ Ru^25^ and Re^15^ and for ketimides of Pd^20,24^ and Rh.^16,22^ The driving force of β-carbon elimination in complexes of these metals is the irreversible release of π-bond containing molecules, while examples of reversible abstraction of ketones or nitriles *via* these mechanisms are still very rare.^16,26^ Meanwhile, β-alkyl elimination is an important (endergonic) chain release mechanism in olefin polymerization catalysed by cationic group 4 metal complexes (Fig. 1C);^27^ however, β-carbon elimination from alcoholate or ketimide complexes of these metals has not been reported yet. The strength of M–X (X = O or N) bonds in these cases^28^ presents a significant hurdle in designing systems that can provide an adequate driving force.

One of the approaches to facilitate C–C bond activation is the promotion of metal–carbon interactions *via* directing groups which dates back to the 1980s with the use of nitrogen heterocycles.^8^ The choice of directing group not only influences the selectivity for a specific C–C bond activation but also reduces activation barriers by participating in the formation of stable metallacyclic intermediates. Pyridine is one of the most popular directing groups in C–H activation;^29^ unsurprisingly, it found application in C–C activation as well. Thus, while complexes of Pd^19–21^ and Rh^16^ do not require directing groups in the substrate to extrude a nitrile or a ketone, the corresponding reactions of Co^30^ and Mn^31^ complexes necessitate the presence of a coordinating nitrogen heterocycle.

Recently, some of us explored the migratory insertion of small polar molecules such as ketones, nitriles, isocyanides, isocyanates, azides, and imines into the M–C_Ar_ bond of pyridylamide^32–34^ (Fig. 1D, I and II) and heteroarylamide^35,36^ (Fig. 1D, III) complexes of hafnium and zirconium with [C,N,N]-ligands.^36–38^ These complexes are renown olefin polymerization precatalysts which upon activation *via* cationization with methylaluminoxane (MAO) or boron-based cocatalysts are modified *in situ* by initial monomer insertion into the M–C_Ar_ bond.^39–41^ Similarly, dimethylated complexes of type III, even in the presence of Hf–CH_3_ moieties, insert nitriles exclusively into the Hf–C_Ar_ bond forming an 8-membered metallacycle with an N(ketimide)–Hf bond.^36^

Here, we report an intriguing case of reversible carbon–carbon bond formation *via* nitrile insertion and extrusion in complexes of types I and II (Fig. 1E). While migratory insertion of nitriles^36^ and isonitriles^42,43^ in group 4 metal–carbon bonds is a well-known transformation, β-carbon elimination with the release of nitrile has never been reported for group 4 metal ketimides. Nitrile release *via* [4 + 2]-retrocycloaddition has been reported by Frye *et al.*^44^ while benzonitrile extrusion has been observed for a hafnium complex by Ghana *et al.*^45^ however, in both examples the reactions are irreversible. The reversibility of migratory insertion in our case allowed us to study the β-carbon elimination process in detail using NMR spectroscopy and DFT calculations establishing the reasons for such unique reactivity.

## Results and discussion

### Reactions of 1-HfCl_2_ and 1-HfMe_2_ with *i*PrCN: experimental study

Late-stage functionalization of hafnium [C,N,N]-ligated heteroarylamide complexes *via* insertion of small polar molecules into Hf–C_Ar_ bonds^36^ was demonstrated to be a convenient way to obtain a series of variously substituted precatalysts for olefin polymerization from a common precursor in one step. We decided to apply this method for functionalization of recently studied [C,N,N]-complexes of type II (Fig. 1D).^34^

**Scheme 1:**
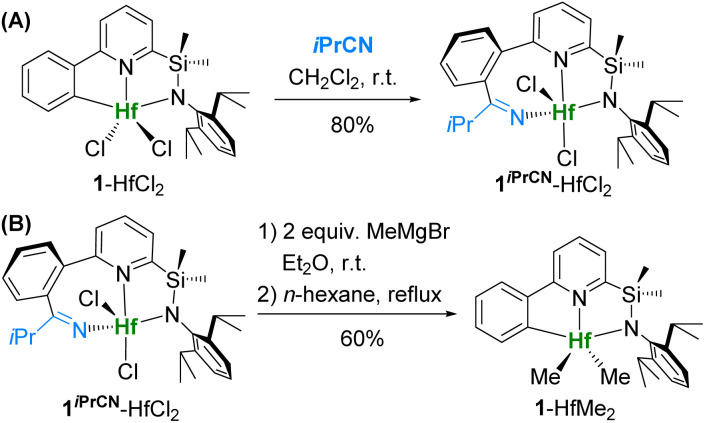
Synthesis of 1^*i*PrCN^-HfCl_2_ and methylation with nitrile extrusion.

Reaction of dichloride complex 1-HfCl_2_ with isobutyronitrile smoothly gives [N,N,N]-ligated product 1^*i*PrCN^-HfCl_2_ in 80% isolated yield (Scheme 1A). In order to obtain dimethyl complex 1^*i*PrCN^-HfMe_2_, which could potentially be activated with borate cocatalysts (such as B(C_6_F_5_)_3_, [Ph_3_C][B(C_6_F_5_)_4_], and [Me_2_HNPh][B(C_6_F_5_)_4_]), dichloride 1^*i*PrCN^-HfCl_2_ was treated with MeMgBr in diethyl ether at room temperature. Surprisingly, after extraction of the product from the crude mixture with hot hexane, we isolated dimethyl complex 1-HfMe_2_ with the original [C,N,N]-ligand as in 1-HfCl_2_ in 60% yield instead of the expected 1^*i*PrCN^-HfMe_2_ (Scheme 1B). This suggests that formation of 1-HfMe_2_ must have occurred *via* extrusion of the nitrile during the methylation of 1^*i*PrCN^-HfCl_2_.

**Scheme 2:**
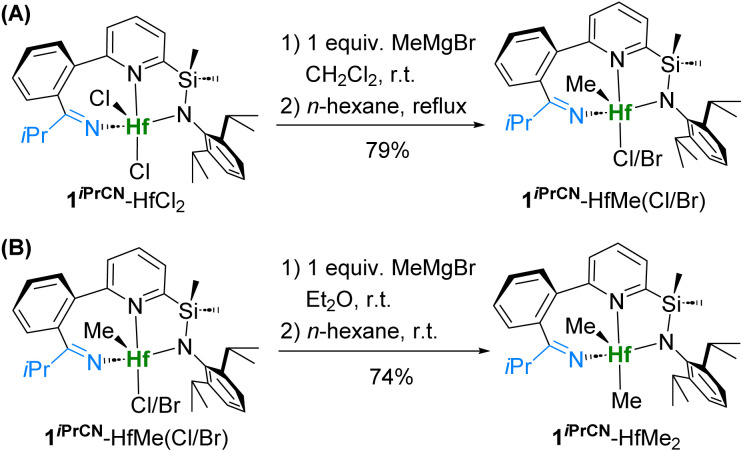
Stepwise methylation of 1^*i*PrCN^-HfCl_2_.

Next, we attempted to substitute the two chloride ligands sequentially to elucidate at which step the nitrile extrusion takes place. The first methylation of 1^*i*PrCN^-HfCl_2_ with 1 equiv. of MeMgBr (Scheme 2A) resulted in selective substitution of the first chloride, and partial substitution of the second one with bromide from the Grignard reagent, giving a mixture of monomethylated complexes 1^*i*PrCN^-HfMe(Cl/Br). The X-ray structure (Fig. S130†) confirmed the presence of the inserted nitrile forming a 7-membered metallacycle. The second methylation of 1^*i*PrCN^-HfMe(Cl/Br) with 1 equiv. of MeMgBr gave dimethyl complex 1^*i*PrCN^-HfMe_2_ still containing the nitrile. As a result, 1^*i*PrCN^-HfMe_2_ was obtained from 1^*i*PrCN^-HfCl_2_ in 57% yield over two steps (Scheme 2B).

**Scheme 3:**
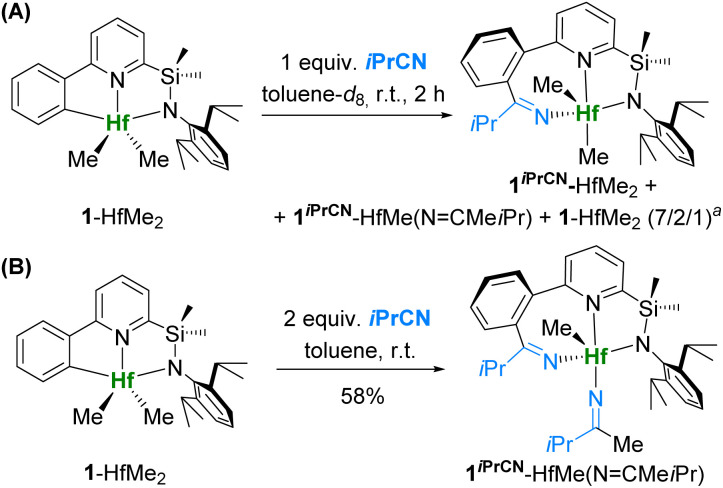
Reactions of 1-HfMe_2_ with isobutyronitrile. ^*a*^ Molar ratio of the products.

An alternative attempt to synthesize 1^*i*PrCN^-HfMe_2_ from dimethyl complex 1-HfMe_2_ through the addition of 1 equiv. of isobutyronitrile to a solution of 1-HfMe_2_ gave a mixture of the following complexes: 1^*i*PrCN^-HfMe_2_ as a major product, 1^*i*PrCN^-HfMe(N=CMe*i*Pr) – a product of double insertion of the nitrile into the Hf–C_Ar_ and Hf–CH_3_ bonds – as a minor product, and unreacted 1-HfMe_2_ in molar ratio = 7/2/1 (Scheme 3A). This experiment demonstrates that the insertion of the nitrile into the Hf–C_Ar_ bond is faster in comparison with insertion into Hf–CH_3_, analogous to what we found earlier for complexes of type III (Fig. 1D).^36^

Addition of two equivalents of nitrile to 1-HfMe_2_ (Scheme 3B) led to quantitative formation of 1^*i*PrCN^-HfMe(N=CMe*i*Pr) (isolated yield 58%), whose structure was further confirmed by 2D NMR (Fig. S23–S33†) and X-ray diffraction crystallography (*vide infra*). Addition of three equivalents of the nitrile to 1-HfMe_2_ yields exclusively 1^*i*PrCN^-HfMe(N=CMe*i*Pr); no product of triple insertion of isobutyronitrile was observed.

**Scheme 4:**
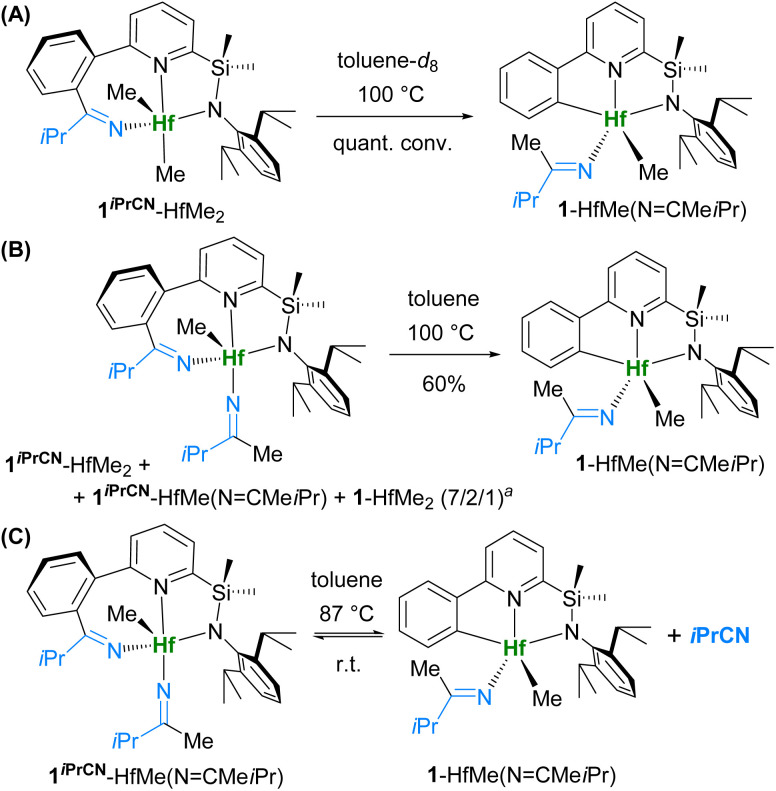
β-Carbon elimination from 1^*i*PrCN^-HfMe_2_ and 1*^i^*^PrCN^-HfMe(N=CMe*i*Pr). Quant. conv. = quantitative conversion. ^*a*^ Molar ratio.

Heating a solution of 1^*i*PrCN^-HfMe_2_ in toluene-*d*_8_ in an NMR tube for 3 h led to formation of 1*^i^*^PrCN^-HfMe(N=CMe*i*Pr) along with 1-HfMe_2_ and 1-HfMe(N=CMe*i*Pr), a product of isobutyronitrile insertion into the Hf–CH_3_ bond only (Fig. S35†). Overnight heating of the reaction mixture resulted in exclusive formation of 1-HfMe(N=CMe*i*Pr) (Scheme 4A), whose structure was confirmed by 2D NMR (Fig. S18–S20†). This experiment convincingly demonstrates that nitrile insertion into the Hf–C_Ar_ bond of 1-HfMe_2_ is indeed reversible. Thus, complex 1-HfMe(N=CMe*i*Pr) is the thermodynamic product of the reaction of 1-HfMe_2_ with isobutyronitrile, whereas 1^*i*PrCN^-HfMe_2_ is the kinetic product. Additionally, 1^*i*PrCN^-HfMe_2_ was dissolved in toluene-*d*_8_ at three concentrations, and the solutions were kept at room temperature and analysed by ^1^H NMR at four time points from 20 min to 2 days. The identical character of the kinetic curves (Fig. S139†) regardless of the concentration, allows us to conclude that the rate-limiting step is unimolecular, and that the migration of the nitrile from C_Ar_ to methyl occurs *via* the nitrile release and reinsertion rather than *via* a bimolecular reaction between two molecules of complexes exchanging the nitrile fragment.

**Fig. 2:**
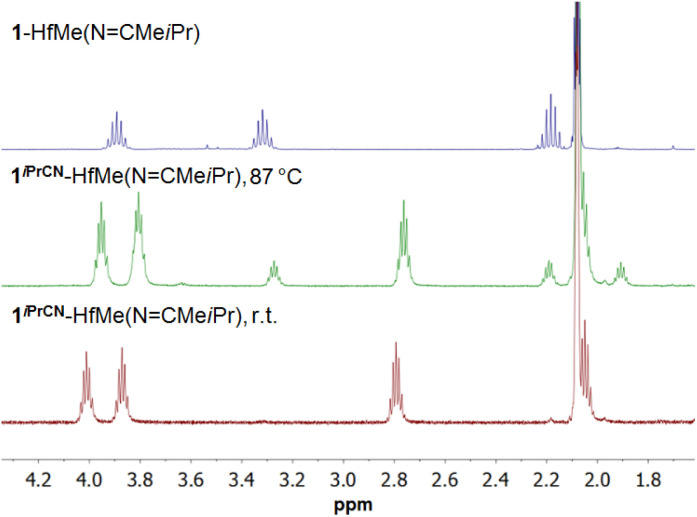
Fragments of ^1^H NMR spectra of complexes 1^*i*PrCN^-HfMe(N=CMe*i*Pr) and 1-HfMe(N=CMe*i*Pr) in toluene-*d*_8_ at room temperature (r.t.) and complex 1^*i*PrCN^-HfMe(N=CMe*i*Pr) in toluene-*d*_8_ at 87 °C.

Heating the mixture of 1-HfMe_2_, 1^*i*PrCN^-HfMe_2_, and 1^*i*PrCN^-HfMe(N=CMe*i*Pr), prepared from 1-HfMe_2_ and *i*PrCN in toluene (Scheme 3A), resulted in exclusive formation of 1-HfMe(N=CMe*i*Pr) as well (Scheme 4B) which was isolated in 60% yield. These observations indicate that nitrile insertion into the Hf–C_Ar_ bond of 1-HfMe(N=CMe*i*Pr) giving 1^*i*PrCN^-HfMe(N=CMe*i*Pr) is reversible, too. Indeed, the ^1^H NMR spectrum of complex 1^*i*PrCN^-HfMe(N=CMe*i*Pr) in toluene-*d*_8_ at 87 °C evidences the formation of complex 1-HfMe(N=CMe*i*Pr) (Scheme 4C and Fig. 2) which disappears again upon cooling to room temperature.

The observed nitrile release can be classified as an example of a β-aryl elimination reaction. While well-known for complexes of Pd^4,14,21,24^ and Rh,^4,16,22,23^ the process has not been reported for group 4 metal complexes before.

The well-defined solution equilibrium of 1^*i*PrCN^-HfMe(N=CMe*i*Pr) (Table 1) at temperatures higher than 60 °C provided an opportunity to study the thermodynamics of the β-aryl elimination process. Dissolving pure 1^*i*PrCN^-HfMe(N=CMe*i*Pr) in toluene-*d*_8_ and heating the solution gives an equilibrium mixture of 1^*i*PrCN^-HfMe(N=CMe*i*Pr), 1-HfMe(N=CMe*i*Pr) and *i*PrCN. A Van't Hoff analysis over a 30 K range yielded thermal parameters shown in Table 1 (entry 1) with Δ*G*_298_ estimated to be 6.4 kcal mol^−1^. Repeating the experiment with dichloride complex 1^*i*PrCN^-HfCl_2_ in toluene-*d*_8_ yields an equilibrium mixture of 1^*i*PrCN^-HfCl_2_, 1-HfCl_2_ and *i*PrCN upon heating it at temperatures higher than 70 °C. A Van't Hoff analysis over a 20 K range yielded thermal parameters shown in Table 1 (entry 2) with Δ*G*_298_ estimated to be 9.2 kcal mol^−1^. Interestingly, switching the solvent to *ortho*-dichlorodeuterobenzene shifts the equilibrium slightly, Δ*G*_298_ = 7.8 kcal mol^−1^ (Table 1, entry 3) for 1^*i*PrCN^-HfCl_2_ ⇆ 1-HfCl_2_ + *i*PrCN, which can be observed already at 60 °C. These findings demonstrate that the spectator ligands on hafnium and the solvent affect the reaction energetics: (a) electron acceptors like chloride stabilize the product of nitrile insertion towards β-aryl elimination and (b) a solvent with a higher dielectric constant destabilizes the product of nitrile insertion towards β-aryl elimination.

Experimental and calculated energetic parameters for the β-aryl elimination reaction of complexes 1^*i*PrCN^-HfCl_2_ and 1^*i*PrCN^-HfMe(N=CMe*i*Pr) in toluene-*d*_8_ or *ortho*-dichlorodeuterobenzene (*o-*C_6_D_4_Cl_2_)

**Table:** 

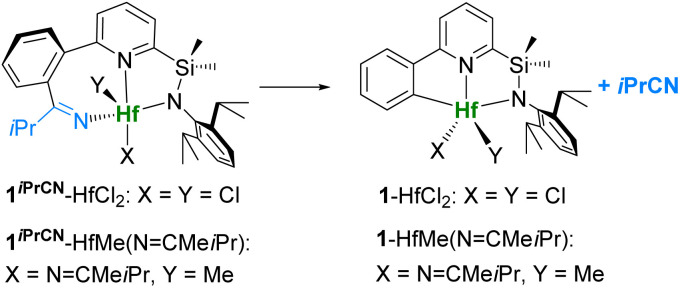					
Entry		Solvent	Δ*H*^a^	Δ*S*^b^	Δ*G*_298_^a^
1	1^*i*PrCN^-HfMe(N=CMe*i*Pr), NMR	Toluene-*d*_8_	17.5(8)	37(2)	6.4
2	1^*i*PrCN^-HfCl_2_, NMR	Toluene-*d*_8_	22(2)	45(7)	9.2
3	1^*i*PrCN^-HfCl_2_, NMR	*o*-C_6_D_4_Cl_2_	17(1)	32(3)	7.8
4	1^*i*PrCN^-HfCl_2_, DFT	Toluene	24.6	48	10.3

a kcal mol^−1^.

b cal mol^−1^ K^−1^.

**Table:** 

Entry		Solvent	Δ*H*^‡^ a	Δ*S*^‡^ b	Δ*G*^‡^_298_^a^
5	1^*i*PrCN^-HfCl_2_, NMR	*o*-C_6_D_4_Cl_2_	20(2)	−10(5)	22.7
6	1^*i*PrCN^-HfCl_2_, DFT	Toluene	21.2	−3	22.1

The rate of interconversion of 1^*i*PrCN^-HfCl_2_ ⇆ 1-HfCl_2_ + *i*PrCN was measured through spin saturation transfer difference (SSTD) experiments in *ortho*-dichlorodeuterobenzene.^46–48^ On-resonance frequency was selected at 2.319 ppm (CH proton of *i*PrCN), which affected the resonance at 2.758 ppm (CH proton of the isopropyl group in 1^*i*PrCN^-HfCl_2_). SSTD data were collected at temperatures between 72.0 and 92.0 °C, and the rate constants were plotted according to the Eyring equation (see the ESI†). This method yields activation parameters for the transformation of Δ*H*^‡^ = 20 ± 2 kcal mol^−1^, and Δ*G*^‡^_298_ = 22.7 kcal mol^−1^ (Table 1, entry 5). The entropy of activation was found to be slightly negative Δ*S*^‡^ = −10 ± 5 cal mol^−1^, possibly due to the higher polarizing effect of *o*-C_6_D_4_Cl_2_ and/or coordination of the latter to the hafnium.

The activation energy for nitrile extrusion from 1^*i*PrCN^-HfCl_2_ implies that the equilibrium 1^*i*PrCN^-HfCl_2_ ⇆ 1-HfCl_2_ + *i*PrCN is already viable at room temperature. Indeed, after addition of PhCN to a solution of 1^*i*PrCN^-HfCl_2_ in *o*-C_6_D_4_Cl_2_, isobutyronitrile was almost completely substituted by benzonitrile in the chelate ligand after 94 h at room temperature (Fig. S120†).

### Reaction of 1^*i*PrCN^-HfMe_2_ with *i*PrCN: DFT calculations

**Fig. 3:**
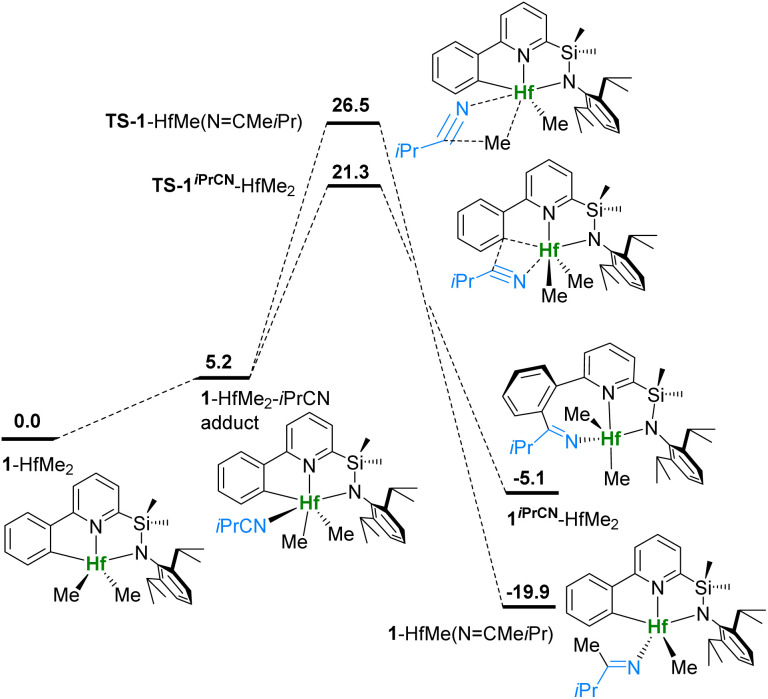
Potential energy surface for insertion of *i*PrCN into Hf–C bonds of 1^*i*PrCN^-HfMe_2_. Gibbs free energies in kcal mol^−1^ at 298 K, 1 atm and 1 equiv. of *i*PrCN.

DFT calculations, according to established protocols,^49,50^ at the TPSSh-D0(SMD)/cc-pVTZ(-PP)//TPSSh/cc-pVTZ level of theory interrogate the reversible insertion of *i*PrCN into the Hf–C_Ar_ bond.^51–62^ Thermal and activation parameters for the 1^*i*PrCN^-HfCl_2_ ⇆ 1-HfCl_2_ + *i*PrCN equilibrium are well reproduced (see Table 1). The competition of insertion into the Hf–C_Ar_ and Hf–CH_3_ bonds of 1-HfMe_2_ is shown in Fig. 3. Coordination of *i*PrCN to 1-HfMe_2_ is endergonic by 5.2 kcal mol^−1^, in line with the observation of free nitrile. Insertion into the Hf–C_Ar_ bond *via*TS-1^*i*PrCN^-HfMe_2_ has a barrier of 21.3 kcal mol^−1^ and is preferred by 5.2 kcal over insertion into the Hf–CH_3_ bond *via*TS-1-HfMe(N=CMe*i*Pr). However, formation of 1^*i*PrCN^-HfMe_2_ is only exergonic by 5.1 kcal mol^−1^ and the reversible C–C elimination barrier (26.4 kcal mol^−1^) is accessible at elevated temperatures. Conversely, insertion into the Hf–CH_3_ bond and formation of 1-HfMe(N=CMe*i*Pr) are highly exergonic (−19.9 kcal mol^−1^) and irreversible even at 100 °C (C–C elimination barrier 46.4 kcal mol^−1^).

**Fig. 4:**
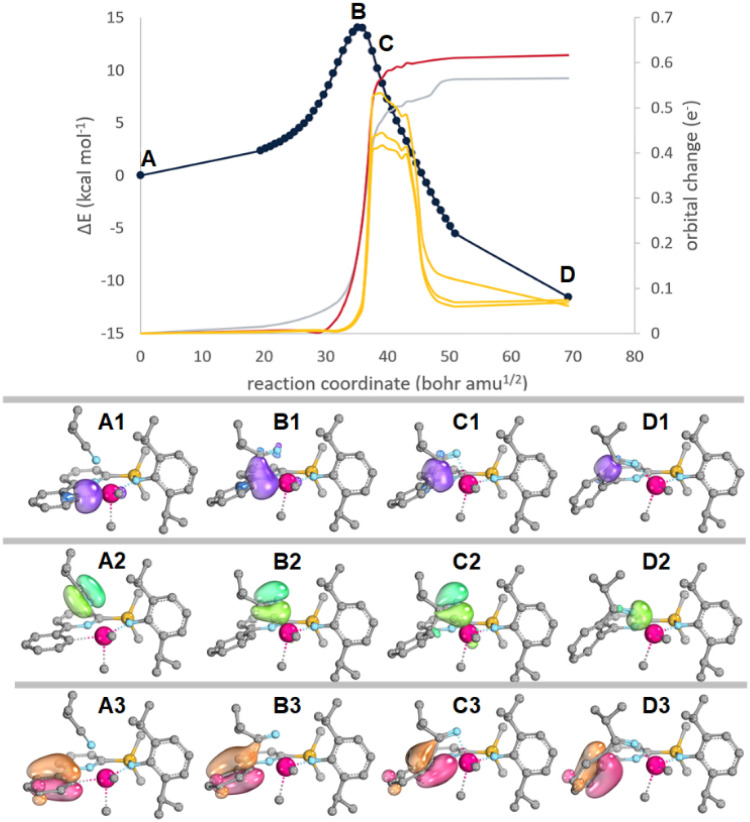
Top: Plot of the root of the sum of square deviations (RSSD) of the partial charge distribution changes along the IRC for *i*PrCN insertion into the Hf–C_Ar_ bond leading to 1-HfMe(N=CMe*i*Pr) *via*TS-1-HfMe(N=CMe*i*Pr). Hf–C_Ar_ (red), the C≡N π (grey) and the three aryl based π IBOs. Bottom: Depiction of the Hf–C_Ar_ (purple, top), C≡N π (green, middle), and one selected aryl π IBO (orange-red) along the IRC. H-atoms omitted for clarity. Movies showing IBOs along the reaction coordinate for *i*PrCN insertion into 1-HfCl_2_ and 1-HfMe_2_ can be found as gif files in ESI (see also page S115).†

Intrinsic bond orbital (IBO)^63–65^ and transition state analyses were employed to analyse the differences in insertion barriers (kinetics). The Hf–C_Ar_ insertion TS leading to 1^*i*PrCN^-HfMe_2_ (TS-1^*i*PrCN^-HfMe_2_) is characterized by smaller distortion energies than the Hf–CH_3_ insertion TS leading to 1-HfMe(N=CMe*i*Pr) (TS-1-HfMe(N=CMe*i*Pr), 27.9 *vs.* 32.0 kcal mol^−1^). Both TSs are energetically early^66^ with respect to the 1-HfMe_2_-*i*PrCN adduct but geometrically central with TS-1^*i*PrCN^-HfMe_2_ showing more short C–C contacts below the van-der-Waals limit than TS-1-HfMe(N=CMe*i*Pr), indicating higher steric strain. The electron flow along the reaction coordinate for the IBOs with the largest change for insertion into the Hf–C_Ar_ bond (TS-1^*i*PrCN^-HfMe_2_) is depicted in Fig. 4. The IBO associated with the Hf–C_Ar_ bond (C: 1.798 e^−^, Hf 0.128 e^−^) becomes the new C–C bond while the IBO associated with the C≡N π-bond transforms into a σ-bond that is largely centred on N (N: 1.856 e^−^, Hf 0.131 e^−^). Therefore, the transformation of the Hf–C_Ar_ bond into the C–C bond in the product can be identified as a nucleophilic attack by the aryl ligand on the electrophilic carbon of the nitrile ligand.

These findings are mirrored for insertion into the Hf–CH_3_ bond (TS-1-HfMe(N=CMe*i*Pr)), with one notable exception: the aryl π-orbitals also significantly change temporarily along the reaction coordinate indicating stabilizing π-donation into metal-based orbitals (Fig. 4, C3). In our opinion, this stabilizing IBO overlap of the aromatic pi-system with the Hf-centre compensates for electronic changes occurring in the Hf–C_Ar_ and N based orbitals along the reaction coordinate. Subsequently, TS-1^*i*PrCN^-HfMe_2_ is shifted and occurs earlier on the reaction coordinate than would be expected without these stabilizing interactions. In fact, the sum of the charges of Hf–C_Ar_/CH_3_ and the N-based orbital is much smaller at the TS for TS-1^*i*PrCN^-HfMe_2_ (0.36 e^−^) than for TS-1-HfMe(N=CMe*i*Pr) (0.46 e^−^). It appears likely that the lower insertion barrier leading to product 1^*i*PrCN^-HfMe_2_ is due to underlying electronic factors, rather than steric differences. Meanwhile, the much lower exergonicity of the insertion into the Hf–C_Ar_ bond forming 1^*i*PrCN^-HfMe_2_ compared to insertion into the Hf–CH_3_ bond forming 1-HfMe(N=CMe*i*Pr) (thermodynamics) results from steric strain and a worse orbital overlap in the former. Formation of 1-HfMe(N=CMe*i*Pr) results in a strain free system with a Hf–N–C_*i*Pr_ angle of 176° while this angle reaches 144° in 1^*i*PrCN^-HfMe_2_. Wiberg bond indices for the largely ionic bonds are lower for 1^*i*PrCN^-HfMe_2_ than 1-HfMe(N=CMe*i*Pr) (IBO: 0.334 *vs.* 0.362; NBO 0.701 *vs.* 0.856).

### Scope of the reaction of RCN with Zr and Hf pyridylamides

**Scheme 5:**
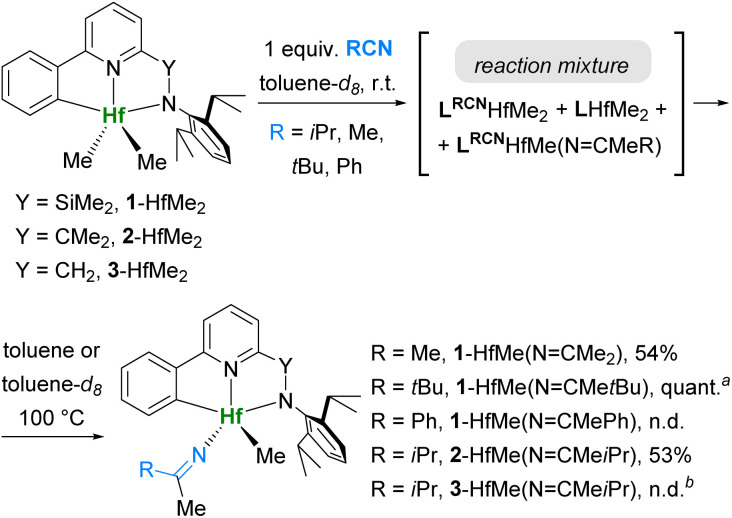
Reactions of L-HfMe_2_ complexes with 1 equiv. of nitriles. ^*a*^ Quantitative conversion by NMR. ^*b*^ Unresolved mixture of products.

**Scheme 6:**
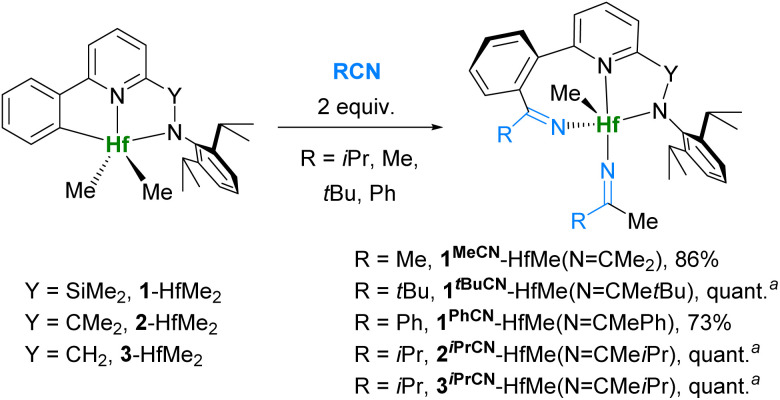
Reactions of L-HfMe_2_ complexes with 2 equiv. of nitriles. ^*a*^ NMR yield.

**Scheme 7:**
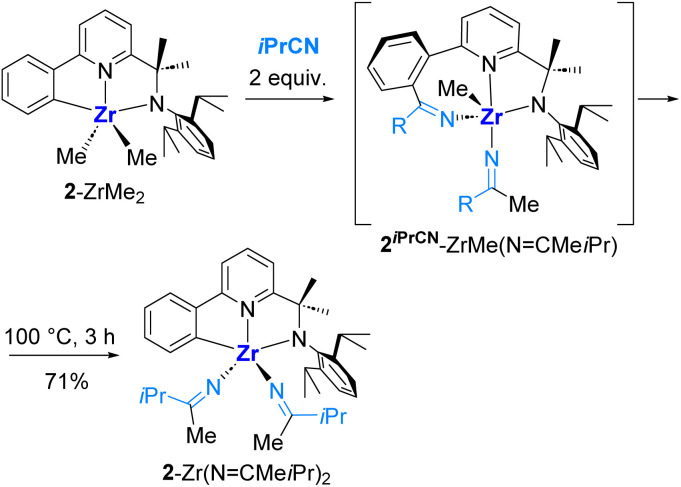
Nitrile extrusion and reinsertion in complex 2^*i*PrCN^-Zr(N=CMe*i*Pr).

Next, we investigated how changes in the pyridylamide ligand, the metal, and the nitrile influence the reactivity pathways. Thus, we chose MeCN, *t*BuCN, and PhCN to study their reactions with 1-HfCl_2_/1-HfMe_2_. Additionally, we studied the reactivity of pyridylamide complexes 2-HfMe_2_, 2-ZrMe_2_, and 3-HfMe_2_ (Scheme 5–7) with *i*PrCN.

Reactions of dimethyl complexes L-HfMe_2_ with 1 equiv. of RCN at r.t. gave mixtures of products of insertions L^RCN^-HfMe_2_ and L^RCN^-HfMe(N=CMeR) with the starting complexes (Scheme 5). Upon stirring at 100 °C, most of these mixtures were converted into the thermodynamic products L-HfMe(N=CMeR), suggesting that the β-carbon elimination from both L^RCN^-HfMe_2_ and L^RCN^-HfMe(N=CMeR) took place.

Contrary to expectations, in the case of reaction between 1-HfMe_2_ and PhCN, heating the mixture did not lead to the formation of pure 1-HfMe(N=CMePh), and after stirring at 100 °C for two days, we found that the major component of the mixture was 1^PhCN^-HfMe(N=CMePh) (Fig. S73†). Accumulation of this double insertion product indicates that the extrusion of PhCN from 1^PhCN^-HfMe(N=CMePh) did not occur at 100 °C, whereas it did from 1^PhCN^-HfMe_2_, which was initially observed in the mixture. Indeed, NMR experiments demonstrated that 1^PhCN^-HfMe(N=CMePh) prepared separately from 1-HfMe_2_ and 2 equiv. of PhCN (Scheme 6) did not release nitrile even at elevated temperatures (Fig. S76†).

One more common transformation was the reaction of complexes L-MMe_2_ with 2 equiv. of nitrile (Scheme 6) giving products of double insertion L^RCN^-MMe(N=CMeR) after few hours at r.t. except for the reaction with bulky *t*BuCN, which required additional stirring at 100 °C for 1.5 days to complete. Complexes 1*^t^*^BuCN^-HfMe(N=CMe*t*Bu) and 2^*i*PrCN^-HfMe(N=CMe*i*Pr) extruded the nitriles reversibly and cleanly upon heating. Meanwhile, complexes 1^MeCN^-HfMe(N=CMe_2_) and 3^*i*PrCN^-HfMe(N=CMe*i*Pr) gave unresolved mixtures after heating their solutions in toluene-*d*_8_ at 100 °C.

Another notable exception was 2^*i*PrCN^-ZrMe(N=CMe*i*Pr), which, upon heating, was found to transform into 2-Zr(N=CMe*i*Pr)_2_, a product of double insertion of *i*PrCN into both Zr–Me bonds, which was obviously formed *via* nitrile extrusion from the former complex and isolated in 71% preparative yield (Scheme 7).

### Molecular structures

In total, we have been able to obtain single crystals for seven complexes, representative of all variations of the ligand framework studied herein (Table 2). Structurally, the complexes can be divided into two groups: with chelate ligands of [C,N,N]- (1-HfMe_2_, 1-HfMe(N=CMe_2_), and 2-Zr(N=CMe*i*Pr)_2_) and [N,N,N]-types (1^*i*PrCN^-HfMe(Cl/Br), 1^*i*PrCN^-HfMe(N=CMe*i*Pr), 1*^t^*^BuCN^-HfMe_2_, and 1^PhCN^-HfMe(N=CMePh).

**Table:** Selected bond distances (Å) and angles (deg) for 1-HfMe_2_, 1^*i*PrCN^-HfMe(Cl/Br), 1*^t^*^BuCN^-HfMe_2_, 1-HfMe(N=CMe_2_), 1^*i*PrCN^-HfMe(N=CMe*i*Pr), 1^PhCN^-HfMe(N=CMePh) and 2-Zr(N=CMe*i*Pr)_2_

Metric	1-HfMe_2_	1^*i*PrCN^-HfMe(Cl/Br)	1*^t^*^BuCN^-HfMe_2_	1-HfMe(N=CMe_2_)	1^*i*PrCN^-HfMe(N=CMe*i*Pr)	1^PhCN^-HfMe(N=CMePh)	2-Zr(N=CMe*i*Pr)_2_
*d*[Hf–C1]	2.204(5)	2.320(11)	2.221(10)	2.208(4)	2.249(5)	2.248(4)	—
*d*[Hf–C2]	2.242(5)	—	2.286(9)	—	—	—	—
*d*[M^a^–C3]	2.271(5)	—	—	2.285(3)	—	—	2.316(4)
*d*[M^a^–N1]	2.328(4)	2.335(8)	2.434(7)	2.362(3)	2.404(4)	2.421(3)	2.341(3)
*d*[M^a^–N2]	2.105(4)	2.054(9)	2.090(9)	2.114(2)	2.097(3)	2.083(3)	2.114(3)
*d*[Hf–N3]	—	2.034(8)	2.034(9)	—	2.053(4)	2.068(3)	—
*d*[M^a^–N4]/*d* [M^a^–N5]	—	—	—	1.979(3)	1.997(4)	1.998(3)	2.023(4)/1.999(3)
*d*[C3–C4]	—	1.496(15)	1.526(14)	—	1.510(6)	1.508(5)	—
*d*[N3–C4]	—	1.273(13)	1.250(12)	—	1.267(6)	1.267(5)	—
*d*[N4–C5]/*d* [N5–C6]	—	—	—	1.259(4)	1.257(6)	1.272(5)	1.262(5)/1.247(10)
∠[Hf–N3–C4]	—	134.5(8)	142.0(8)	—	136.3(3)	138.9(3)	—
∠[M^a^–N4(N5)–C5(C6)]	—	—	—	169.8(3)	174.8(4)	168.6(3)	173.3(3)/168.4(6)

a M = Hf for 1-HfMe_2_, 1^*i*PrCN^-HfMe(Cl/Br), 1*^t^*^BuCN^-HfMe_2_, 1-HfMe(N=CMe_2_), 1^*i*PrCN^-HfMe(N=CMe*i*Pr), and 1^PhCN^-HfMe(N=CMePh) and M = Zr for 2-Zr(N=CMe*i*Pr)_2_.

**Fig. 5:**
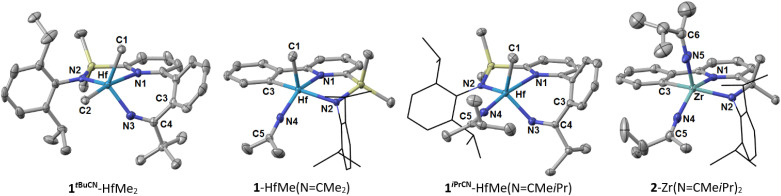
Solid state molecular structure of 1*^t^*^BuCN^-HfMe_2_, 1-HfMe(N=CMe_2_), 1^*i*PrCN^-HfMe(N=CMe*i*Pr), and 2-Zr(N=CMe*i*Pr)_2_ with ellipsoids drawn at the 50% probability level. The hydrogen atoms are removed for clarity.

All seven complexes share the pyridine-Y-*N*-diisopropylphenyl moiety (where Y = SiMe_2_ or CMe_2_) whose geometrical parameters fall in the range known for Zr and Hf complexes containing the same fragments (*ca.* 2.25–2.45 Å for M–N1 and 2.05–2.15 Å for M–N2, Fig. 5)^33,34,67,68^ and differ insignificantly between the two groups of the chelate ligands. Although NMR spectra of the symmetrically substituted [C,N,N]-complexes 1-HfMe_2_ and 2-Zr(N=CMe*i*Pr)_2_ exhibit *C*_s_-symmetry in solution, the geometry of the coordination surrounding the metal in the solid-state is best described as a distorted square pyramid. Insertion of nitrile results in expansion of the 5-membered metallacycle containing the C_Ar_–M bond (C3–M in 1-HfMe_2_, 1-HfMe(N=CMe_2_) and 2-Zr(N=CMe*i*Pr)_2_), Fig. 5) to the 7-membered metallacycle, and change in the coordination polyhedron to a distorted trigonal bipyramid.

There are four types of metal–nitrogen bonds in the studied complexes (Table 2). The coordination bonds M–N1(pyridine) of 2.328(4)–2.434(7) Å are the longest. The shortest bonds are M–N4 (“linear” ketimides) of 1.979(3)–1.923(4) Å which are considerably shorter than M–N2(amide) bonds of 2.054(9)–2.114(3) Å. The bond shortening can be traced to a bond order higher than one. Indeed, sp-hybridization of the nitrogen atom in the ketimide is additionally witnessed by the large M–N4(N5)–C5(C6) angle of 168.4(6)–174.8(4)°. A similar geometry was observed previously for several other “linear” ketimides of group 4 metals.^45,69–71^ At the same time, Hf–N3 (“bent” ketimide) bonds of 2.034(9)–2.068(3) Å are notably longer than Hf–N4. In fact, Hf–N3 bond lengths have values that are in between those of Hf–N2 and Hf–N4 bonds. This may be related to the smaller Hf–N3–C4 angle of 134.5(8)–142.0(8)° which hinders donation of the N3-nitrogen lone pair into hafnium based orbitals. The M–N bond elongation on decreasing the M–N–C angle is typical for ketimides of group 4 metals and was reported for tethered^45,72,73^ and sterically hindered complexes^74,75^ previously. Lengths of double C=N bonds (N3–C4, N4–C5 and N5–C6 in Table 2) of 1.250(12)–1.273(13) Å are virtually the same for “linear” and “bent” ketimides of Hf and Zr which is in line with previous observations for “linear” non-tethered ketimides (≈1.259 Å)^69,71^ and “bent” tethered ligands (≈1.261–1.269 Å).^72,73^

## Conclusions

Reaction of pyridylamido hafnium complexes 1-HfCl_2_, 1-HfMe_2_, 2-HfMe_2_, 3-HfMe_2_ and 2-ZrMe_2_ with isobutyronitrile predominantly gives the products of the nitrile insertion into the Hf–C_Ar_ bond 1^*i*PrCN^-HfCl_2_, 1^*i*PrCN^-HfMe_2_, 2^*i*PrCN^-HfMe_2_, 3^*i*PrCN^-HfMe_2_ and 2^*i*PrCN^-ZrMe_2_. Surprisingly, these products were found to be unstable with respect to nitrile extrusion in solution already at room temperature. The observed reversible nitrile insertion represents the first example of β-aryl elimination in ketimides of early transition metals, previously reported only for complexes of Pd^20^ and Rh.^22^ Moreover, complexes 1^*i*PrCN^-HfCl_2_ and 1^*i*PrCN^-HfMe(N=CMe*i*Pr) are able to reversibly release isobutyronitrile in solution forming the well-defined equilibria (1^*i*PrCN^-HfCl_2_ ⇆ 1-HfCl_2_ + *i*PrCN and 1^*i*PrCN^-HfMe(N=CMe*i*Pr) ⇆ 1-HfMe(N=CMe*i*Pr) + *i*PrCN) which allowed us to study them in detail with NMR spectroscopy.

Analysis of the nitrile insertion and extrusion processes by DFT computations allowed us to conclude that the “bent” structure of ketimide plays a significant role in promoting the β-carbon elimination event in the described complexes but this is not the only prerequisite for this reactivity. While weak orbital overlap between N(ketimide) and metal and an unfavourable 7-membered metallacycle destabilize the product of insertion into the M–C_Ar_ bond, it is the backbone of the pyridylamide ligand that makes the reverse process viable. The pyridyl group linked with an amide fragment serves as a directing group to maintain proximity between the metal centre and the phenylene fragment required for Hf–C_Ar_ bond formation and, furthermore, assures the formation of the stable 5-membered metallacycle. The complexes described in the literature, which contain “bent” ketimide ligands,^45,72,73^ lack such a directing group, and as a result, they are unable to undergo β-carbon elimination.

Electronic properties of the spectator ligands markedly influence thermodynamic stability of the complexes as it was demonstrated for complexes 1^*i*PrCN^-HfCl_2_, 1^*i*PrCN^-HfMe_2_ and 1^*i*PrCN^-HfMe(N=CMe*i*Pr) in Table 1. Likely, electron withdrawing substituents on the metal increase the N(“bent” ketimide)–M bond energy and stabilize the complex. At the same time, substituents on the inserted nitrile were also found to be important. Thus, while complex 1*^t^*^BuCN^-HfMe_2_ with inserted *t*BuCN was isolated in good yield, complex 1^MeCN^-HfMe_2_ with inserted acetonitrile could only be traced in the NMR spectrum. Moreover, even though complexes 1^*i*PrCN^-HfMe(N=CMe*i*Pr) and 1^PhCN^-HfMe(N=CMePh) have almost identical structural parameters (Table 2), 1^*i*PrCN^-HfMe(N=CMe*i*Pr) is in equilibrium with 1-HfMe(N=CMe*i*Pr) and *i*PrCN at 60 °C, whereas 1^PhCN^-HfMe(N=CMePh) does not eliminate PhCN even at 100 °C, implying that the conjugation of the C=N bond and phenyl group provides an additional stabilization of the inserted product.

The transformations reported in this work demonstrate for the first time that ketimides of Zr and Hf are able to undergo β-carbon elimination analogously to late transition metal complexes. Given the growing importance of such reactions in the field of catalysis for carbon–carbon bond activation, our study paves the path towards the application of cheap and earth abundant group 4 metals in these transformations in the future.

## Data availability

The data supporting this article have been included as part of the ESI.† Crystallographic data for 1^*i*PrCN^-HfMe(Cl/Br), 1-HfMe_2_, 1*^t^*^BuCN^-HfMe_2_, 1-HfMe(N=CMe_2_), 1^*i*PrCN^-HfMe(N=CMe*i*Pr), 1^PhCN^-HfMe(N=CMePh) and 2-Zr(N=CMe*i*Pr)_2_ have been deposited and are available free of charge from the Cambridge Crystallographic Data Center (CCDC No. 2301574–2301580) and can be obtained from https://www.ccdc.cam.ac.uk.

## Author contributions

PSK: investigation, writing original draft (organometallic part); GPG, ANY, and DYM: investigation (organometallic part); DVU: supervision (MSU team), writing – review & editing; CE: investigation and writing original draft (computational part); JAMC and JRH: conceptualization; AZV: project administration, funding acquisition, resources.

## Conflicts of interest

There are no conflicts to declare.

## Supplementary Material

SC-OLF-D4SC02173H-s001

SC-OLF-D4SC02173H-s002

SC-OLF-D4SC02173H-s003

SC-OLF-D4SC02173H-s004

SC-OLF-D4SC02173H-s005

SC-OLF-D4SC02173H-s006

SC-OLF-D4SC02173H-s007
