# Supplementary figures and images for: Reversible C–C bond formation in group 4 metal complexes: nitrile extrusion via β-aryl elimination
Source: Chem Sci. 2024 Aug 27;15(38):15825–34. doi: 10.1039/d4sc02173h (PMC11388100; doi:10.1039/d4sc02173h)

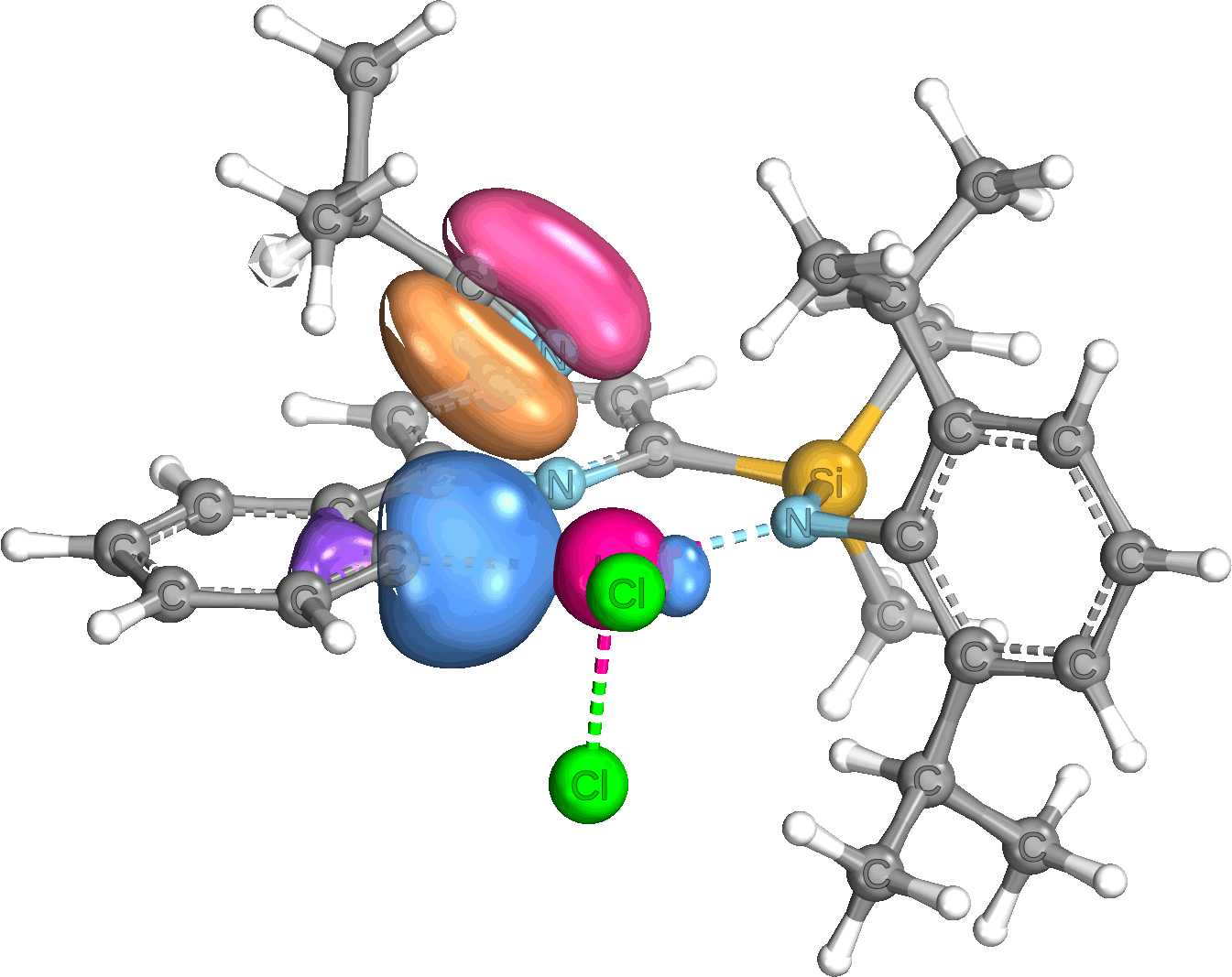

Supplement: SC-015-D4SC02173H-s001 [file SC-015-D4SC02173H-s001.gif]

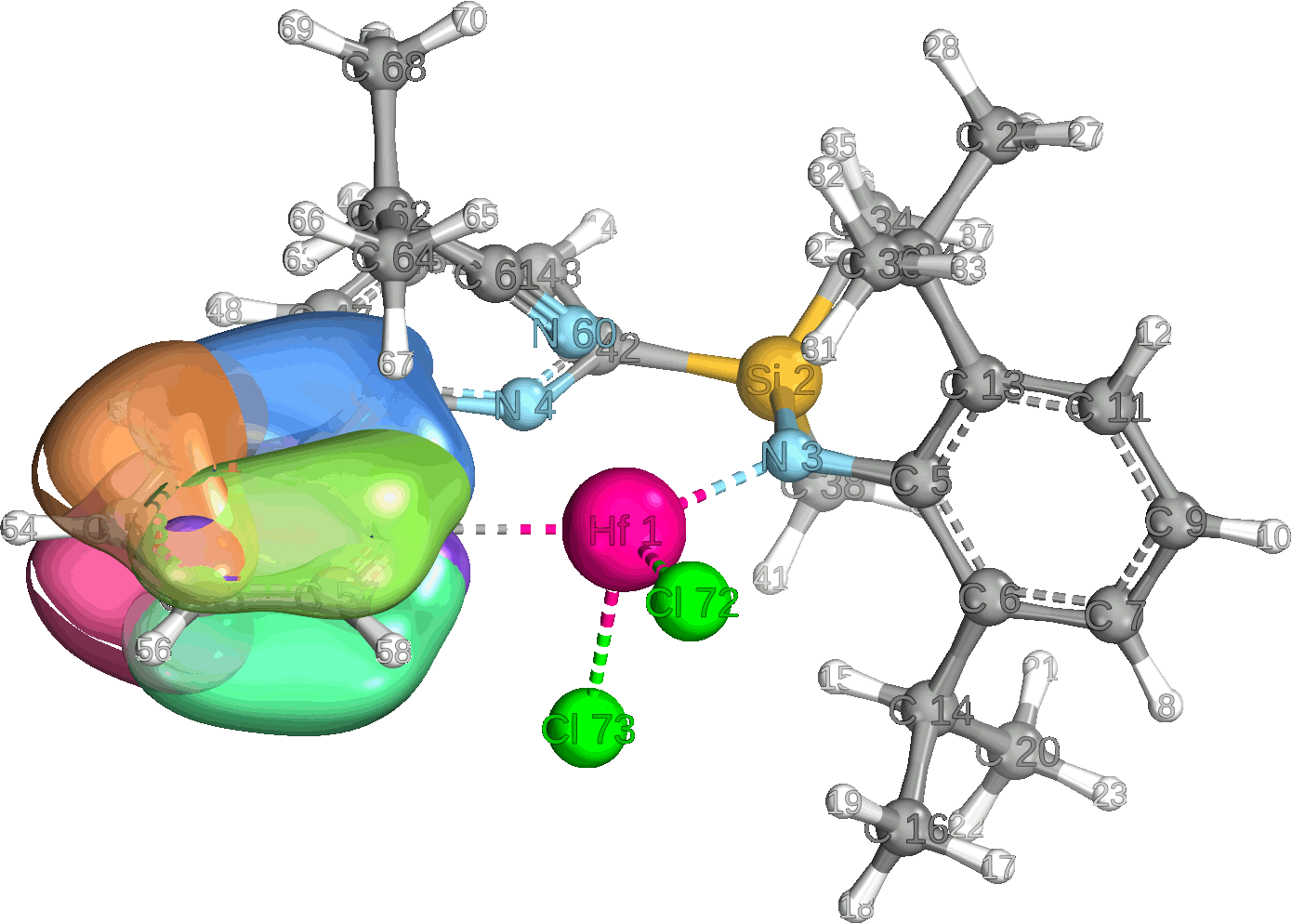

Supplement: SC-015-D4SC02173H-s002 [file SC-015-D4SC02173H-s002.gif]

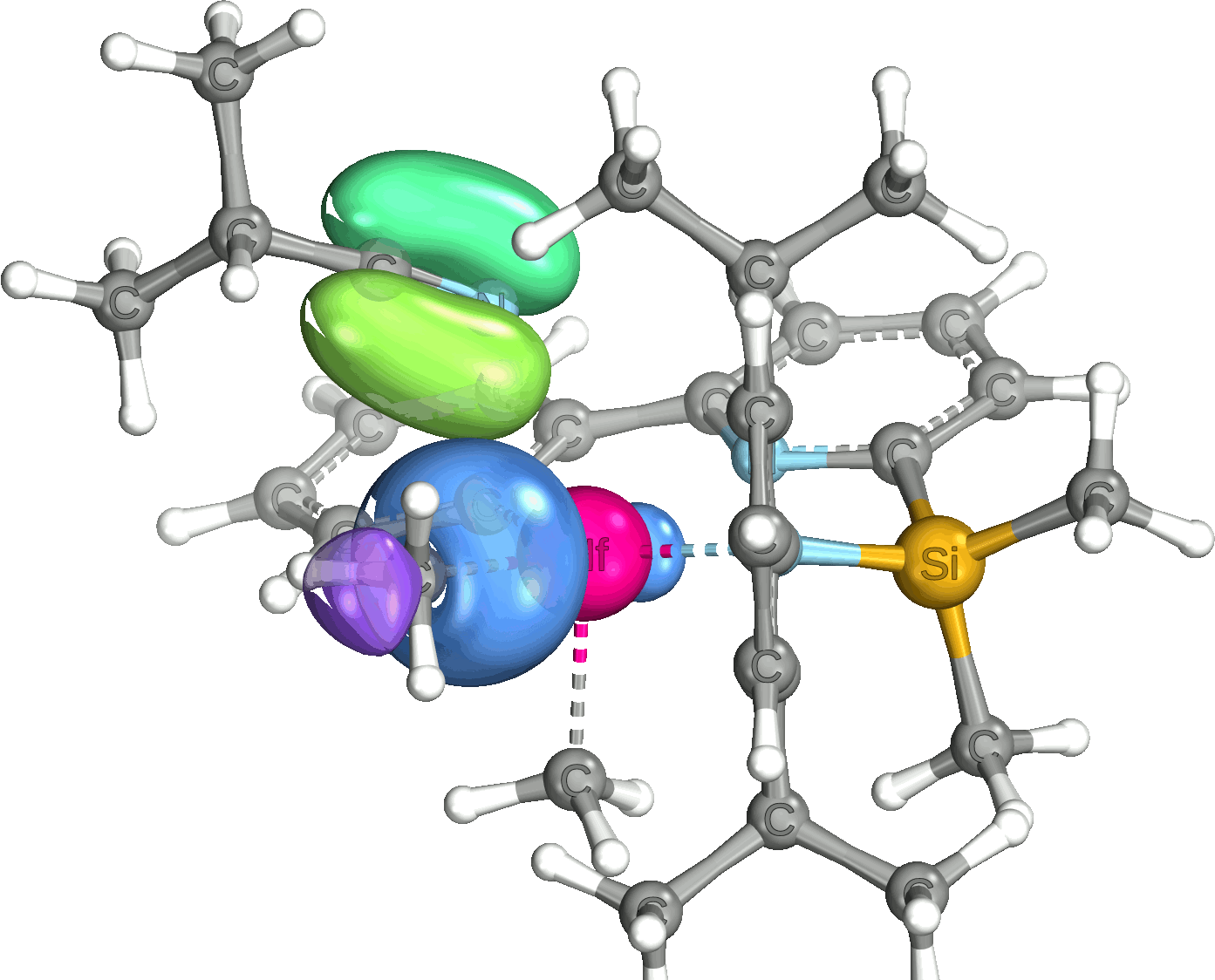

Supplement: SC-015-D4SC02173H-s003 [file SC-015-D4SC02173H-s003.gif]

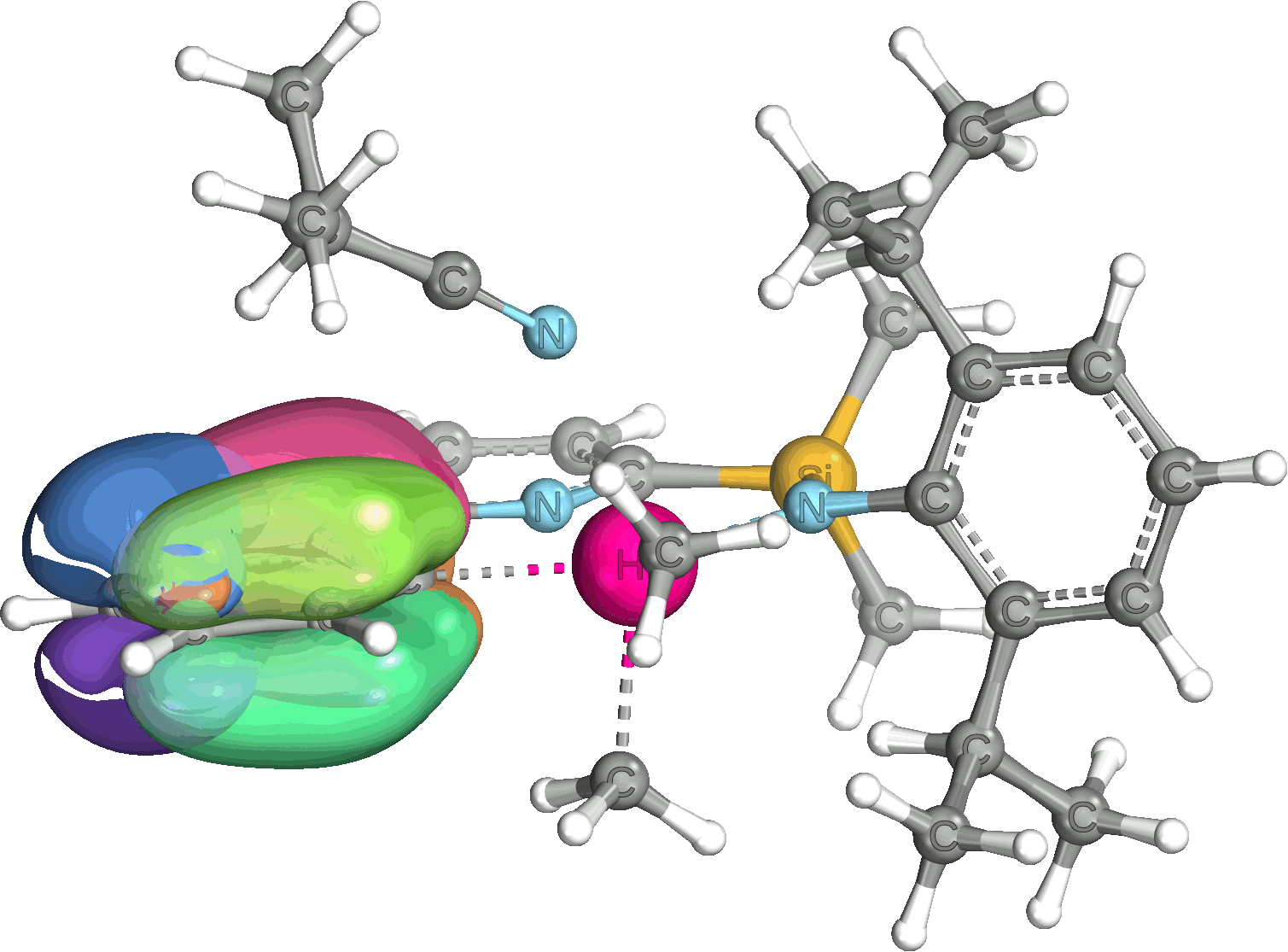

Supplement: SC-015-D4SC02173H-s004 [file SC-015-D4SC02173H-s004.gif]

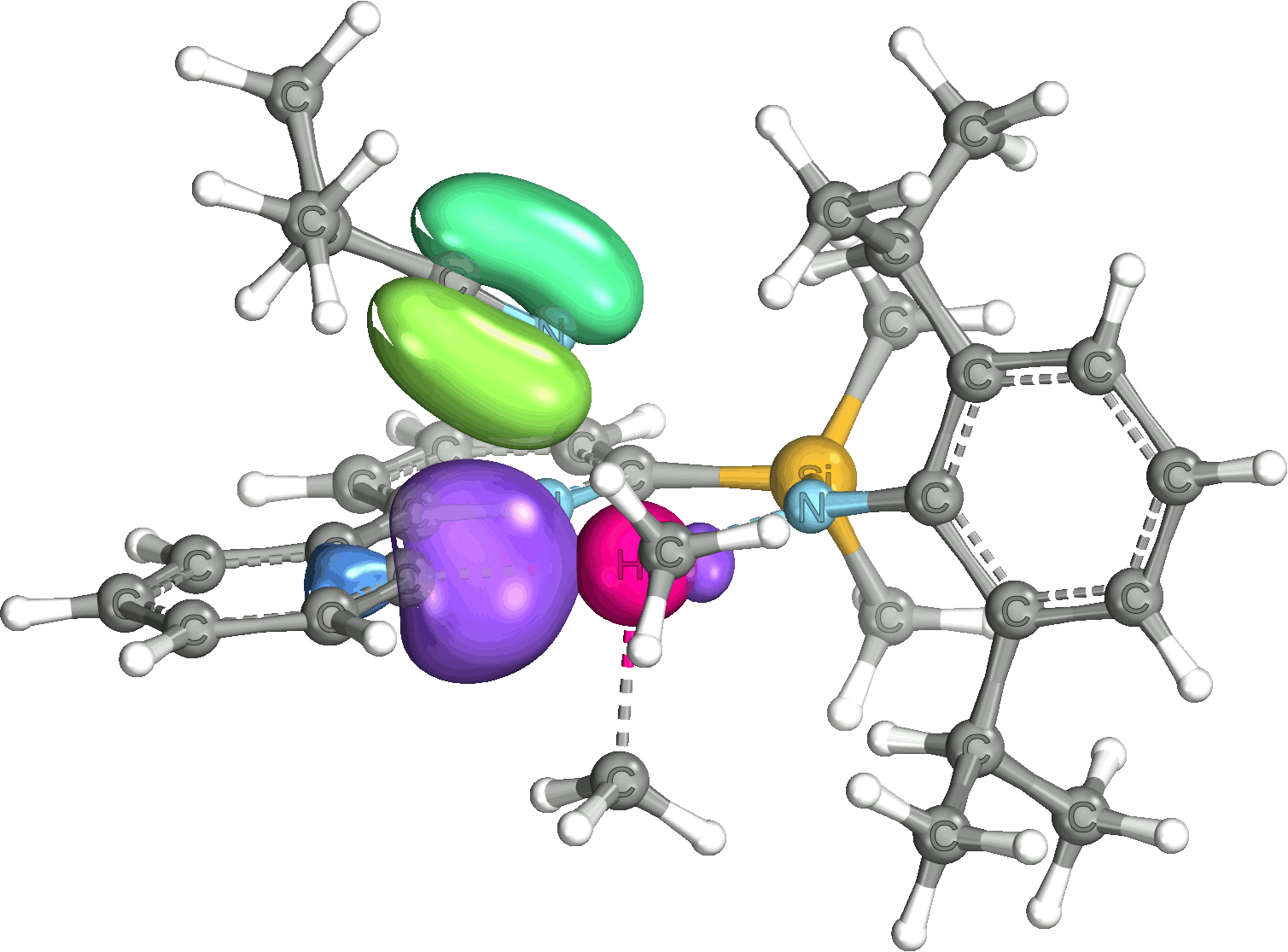

Supplement: SC-015-D4SC02173H-s005 [file SC-015-D4SC02173H-s005.gif]
