# Supplementary material for: Reversible C–C bond formation in group 4 metal complexes: nitrile extrusion via β-aryl elimination
Source: Chem Sci. 2024 Aug 27;15(38):15825–34. doi: 10.1039/d4sc02173h (PMC11388100; doi:10.1039/d4sc02173h)
Supplement: SC-015-D4SC02173H-s006 [file SC-015-D4SC02173H-s006.pdf]

Supplementary Information (SI) for Chemical Science.
This journal is © The Royal Society of Chemistry 2024

Reversible C-C Bond Formation in Group 4 Metal Complexes: Nitrile Extrusion via *β*-Aryl Elimination

Pavel S. Kulyabin,[a] Georgy P. Goryunov,[a] Andrei N. Iashin,[a] Dmitry Y. Mladentsev,[a] Dmitry V. Uborsky,[a] Christian Ehm,[b] Jo Ann M. Canich,[c] John R. Hagadorn,[c] and Alexander Z. Voskoboynikov*[a]

[a] Department of Chemistry, M. V. Lomonosov Moscow State University, Leninskie Gory, 1/3, Moscow 119991 (Russian Federation)

[b] Dipartimento di Scienze Chimiche, Università di Napoli Federico II, Via Cintia, 80126 Napoli (Italy)

[c] Baytown Technology and Engineering Complex, ExxonMobil Chemical Company, Baytown, TX, 77520 (USA)

* E-mail: voskoboy@med.chem.msu.ru

Table of contents

1. General Experimental 2
2. Syntheses, spectroscopic data and illustrations of the NMR spectra 3
3. Variable temperature van't Hoff study of the equilibrium of **1^{iPrCN}-HfMe(N=CMe*i*Pr)** and **1-HfMe(N=CMe*i*Pr)** 98
4. Variable temperature van't Hoff study of the equilibrium of **1^{iPrCN}-HfCl$_2$** and **1-HfCl$_2$** 99
5. Variable temperature van't Hoff study of the equilibrium of **1^{iPrCN}-HfCl$_2$** and **1-HfCl$_2$** 100
6. Spin saturation transfer difference for **1^{iPrCN}-HfCl$_2$** 101
7. Crystal structure determinations. 106
8. Computational Details 115
9. Kinetic curves for the *i*PrCN extrusion from **1^{iPrCN}-HfMe$_2$** at different concentrations 120
10. References 121

1. General Experimental

Reactions involving air or moisture sensitive compounds were carried out under inert atmosphere (argon or nitrogen) using standard Schlenk techniques or a glove box environment (Vacuum Atmospheres Co. and M. Braun Inertgas-Systeme GmbH). All solvents were dried over 4 Å molecular sieves prior to use. The amount of residual water present in the THF and diethyl ether was quantified, using a Karl Fischer Coulometer C20 (Mettler Toledo), and found to be below 10.0 ppm. Acetonitrile, isobutyronitrile, trimethylacetonitrile and benzonitrile were dried over 4 Å molecular sieves prior to use. $HfBn_2Cl_2(Et_2O)$ was prepared by reaction of one equivalent of $HfBn_4$ (Strem) with $HfCl_4$ (Strem) in ether for 5 h followed by filtration and crystallization of the product from the filtrate. (2,6-Diisopropylphenyl)[dimethyl(6-phenylpyridin-2-yl)silyl]amine [1], 2,6-diisopropyl-*N*-(2-(6-phenylpyridin-2-yl)propan-2-yl)aniline [2] and 2,6-diisopropyl-*N*-((6-phenylpyridin-2-yl)methyl)aniline [3] were synthesized according to the literature procedures. Nuclear magnetic resonance spectra were recorded on Bruker AVANCE 400 (400 MHz) and Bruker AVANCE-II (600 MHz) spectrometers at ambient temperature (unless otherwise stated). NOESY experiments were carried out with 250 ms mixing time while for ROESY experiment value of 300 ms was used. Chemical shifts are quoted in parts per million (ppm) and were referenced to residual solvent peaks using values provided by the MestReNova processing software. Coupling constants (J) are quoted in Hertz. Coupling patterns are written using the following abbreviations: br s (broad), s (singlet), d (doublet), t (triplet), q (quartet), m (multiplet) and app (apparent). GC/MS analysis was performed on an Agilent Technologies 8890 GC/5977C MSD system. C, H, N microanalyses were done using a Perkin Elmer 2400 Series II CHNS/O elemental analyzer.

2. Syntheses, spectroscopic data and illustrations of the NMR spectra

2.1. Complex 1-HfCl$_2$

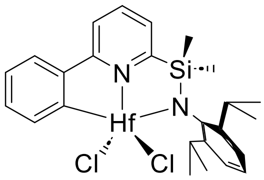

(2,6-Diisopropylphenyl)[dimethyl(6-phenylpyridin-2-yl)silyl]amine (1.00 g, 2.57 mmol) was dissolved in benzene (80 mL) and HfCl$_2$Bn$_2$(Et$_2$O) (1.30 g, 2.57 mmol) was added in one portion. The resulting solution was stirred overnight in a pressure vessel at 70 °C forming a slurry of a white precipitate in a yellow solution. The mixture was then filtered through a short pad of Celite® in a Glovebox, the filtrate was evaporated to dryness, and the residue was washed with hot hexanes (30 mL). The obtained solid was recrystallized from a toluene-hexane mixture giving **1-HfCl$_2$** as a yellowish crystalline solid (1.16 g, 71%). Anal. calc. for $C_{25}H_{30}Cl_2HfN_2Si$: C, 47.21; H, 4.75; N, 4.40. Found: C, 47.44; H, 4.83; N, 4.24.

^{1}H NMR (400 MHz, CD_2Cl_2) δ_H: 8.17 (1H, m), 7.97–8.06 (2H, m), 7.90 (1H, m), 7.63 (1H, m), 7.39 (2H, m), 7.07–7.16 (3H, m), 3.39 (2H, m, C*H*Me$_2$), 1.25 (6H, d, $^3J_{HH}$ = 6.9 Hz, ArCH(C*H*$_3$)$_2$), 1.15 (6H, d, $^3J_{HH}$ = 6.7 Hz, ArCH(C*H*$_3$)$_2$), 0.39 (6H, s, Si(C*H*$_3$)$_2$).

^{13}C{^{1}H} NMR (101 MHz, CD_2Cl_2) δ_C: 199.6 (C_{Ar}-Hf), 170.2, 164.0, 146.2, 144.4, 144.1, 143.5, 141.2, 131.1, 130.3, 128.7, 128.0, 124.8, 124.2, 123.7, 199.5, 28.7, 26.1, 24.4, -0.2 ((CH$_3$)$_2$Si).

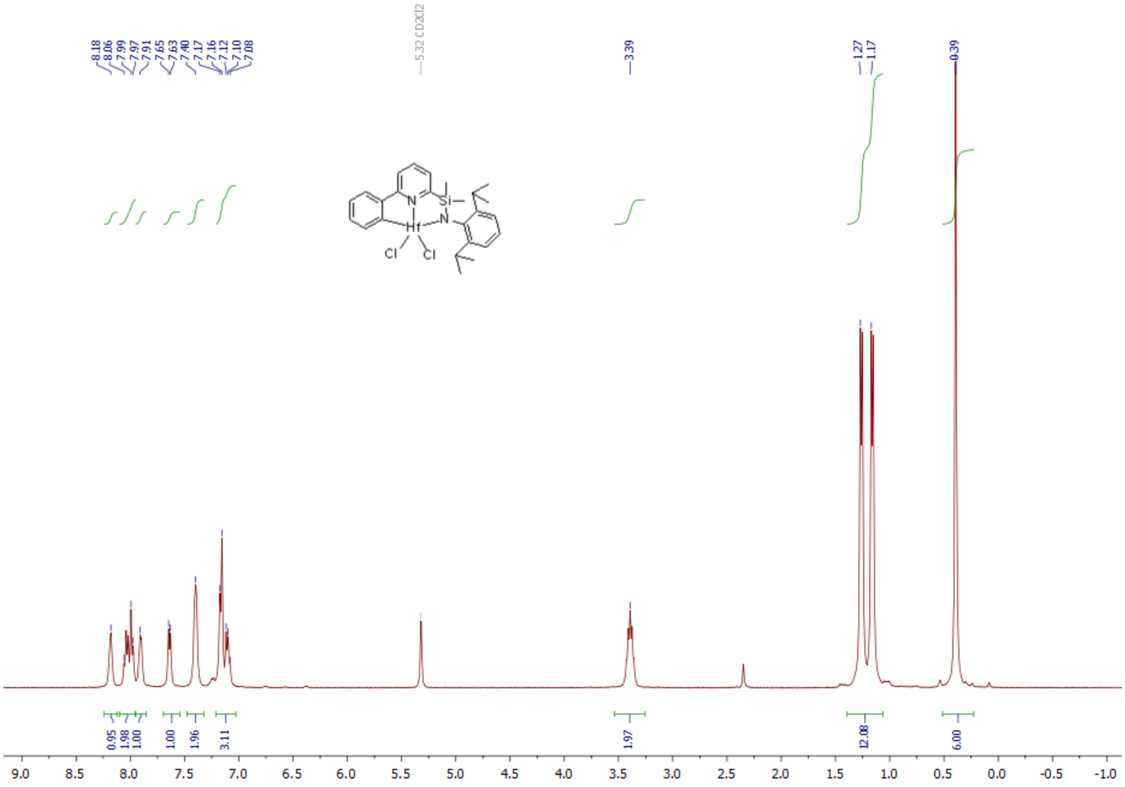

Figure S1. ^{1}H NMR spectrum of **1-HfCl$_2$** in CD_2Cl_2 at room temperature.

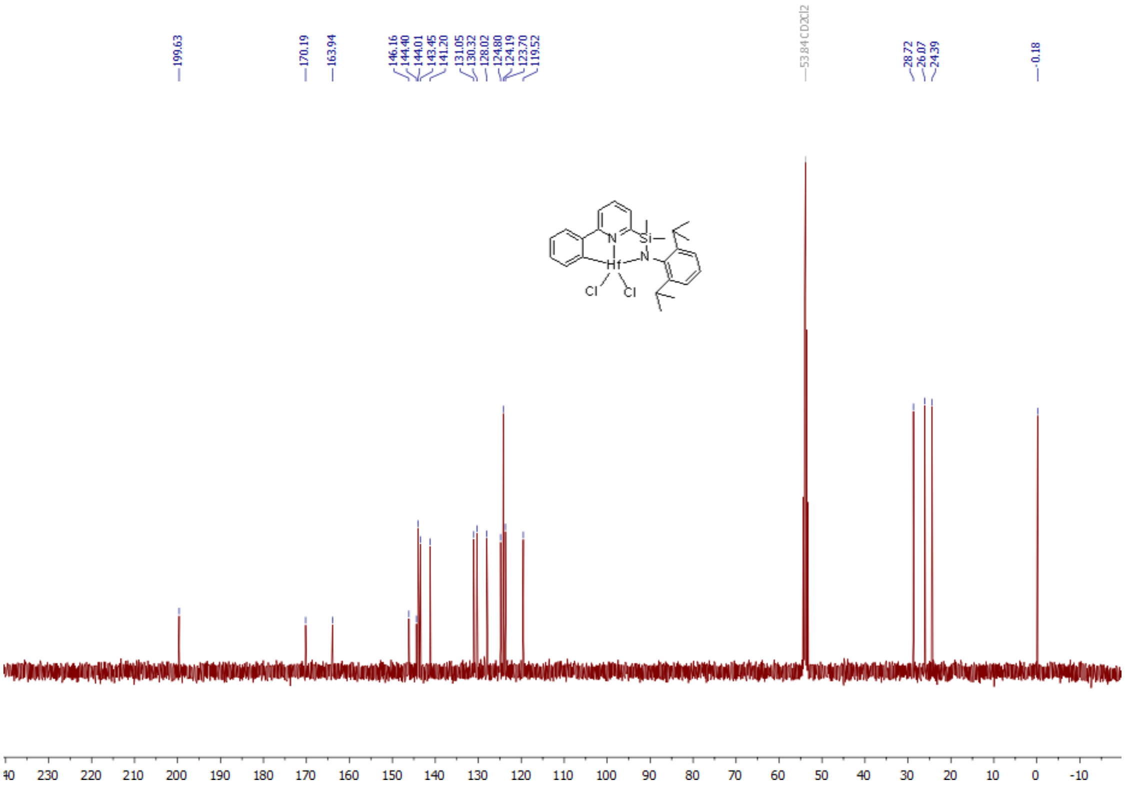

Figure S2. $^{13}C\{^1H\}$ NMR spectrum of **1-HfCl$_2$** in CD_2Cl_2 at room temperature.

2.2. Complex 1^{iPrCN}-$HfCl_2$

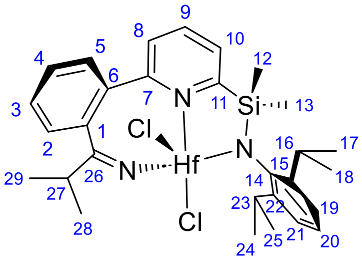

Complex **1-HfCl$_2$** (286 mg, 0.45 mmol) was dissolved in dichloromethane (20 mL) and isobutyronitrile (32 mg, 0.45 mmol) was added. The resulting yellow solution was stirred for 30 min at room temperature. After that hexane (30 mL) was added, and volatiles were evaporated until precipitation occurred. The precipitate was filtered off while still cold and then washed with hexane (2 × 5 mL) and dried in vacuum. Complex **1^{iPrCN}-HfCl$_2$** was obtained as an amorphous yellow solid (255 mg, 80%). Anal. calc. for $C_{29}H_{37}Cl_2HfN_3Si$: C, 49.40; H, 5.29; N, 5.96. Found: C, 49.71; H, 5.45; N, 5.65.

^{1}H NMR (400 MHz, CD_2Cl_2) δ_H: 8.00 (1H, m, C(9)H), 7.70 (1H, m, C(10)H), 7.48–7.55 (5H, m, C(8)H and C(2)H), 7.11–7.20 (3H, m, aryl C*H*), 3.78 (1H, m, C(16)*H*), 3.65 (1H, m, C(23)*H*), 3.03 (1H, m, C(27)*H*), 1.42 (3H, d, $^3J_{HH}$ = 6.7 Hz), 1.31 (3H, d, $^3J_{HH}$ = 6.8 Hz), 1.25–1.27 (6H, m), 1.08 (3H, d, $^3J_{HH}$ = 6.4 Hz, C(28)*H*), 0.65 (3H, d, $^3J_{HH}$ = 7.1 Hz, C(29)*H*), 0.42 (3H, s, C(12)*H*), 0.35 (3H, s, C(13)*H*).

^{13}C{^{1}H} NMR (101 MHz, CD_2Cl_2) δ_C: 182.0 (C-26), 167.4 (C-11), 158.7 (C-7), 147.4, 145.3, 145.1, 141.8, 138.4, 136.3, 132.3, 129.7, 129.3, 128.7, 125.3, 124.6, 124.4, 123.7, 37.7, 28.0, 27.8, 26.7, 26.6, 24.9, 24.8, 19.8, 18.9, 1.1, 0.8.

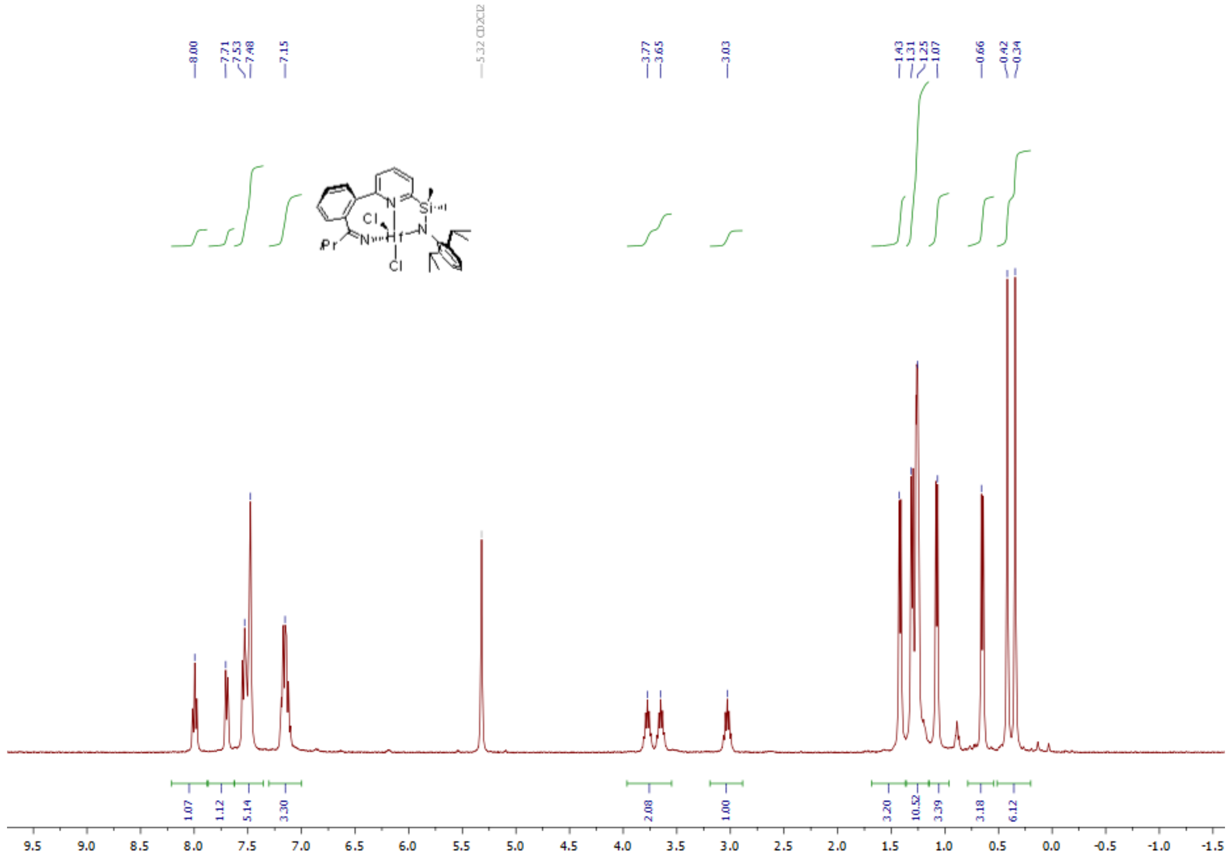

Figure S3. ^{1}H NMR spectrum of $\mathbf{1}^{iPrCN}$-$\mathbf{HfCl_2}$ in CD_2Cl_2 at room temperature.

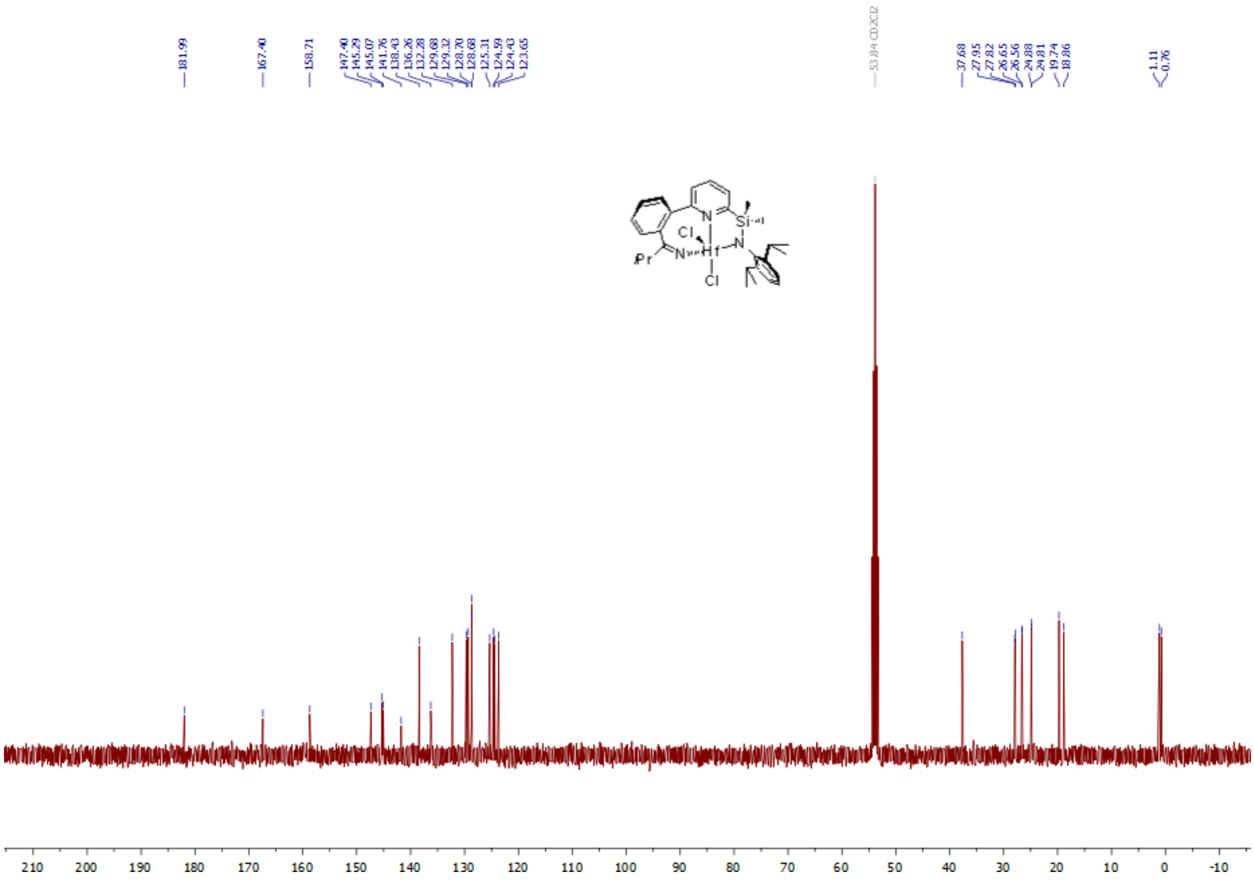

Figure S4. $^{13}C\{^{1}H\}$ NMR spectrum of $\mathbf{1}^{iPrCN}$-$\mathbf{HfCl_2}$ in CD_2Cl_2 at room temperature.

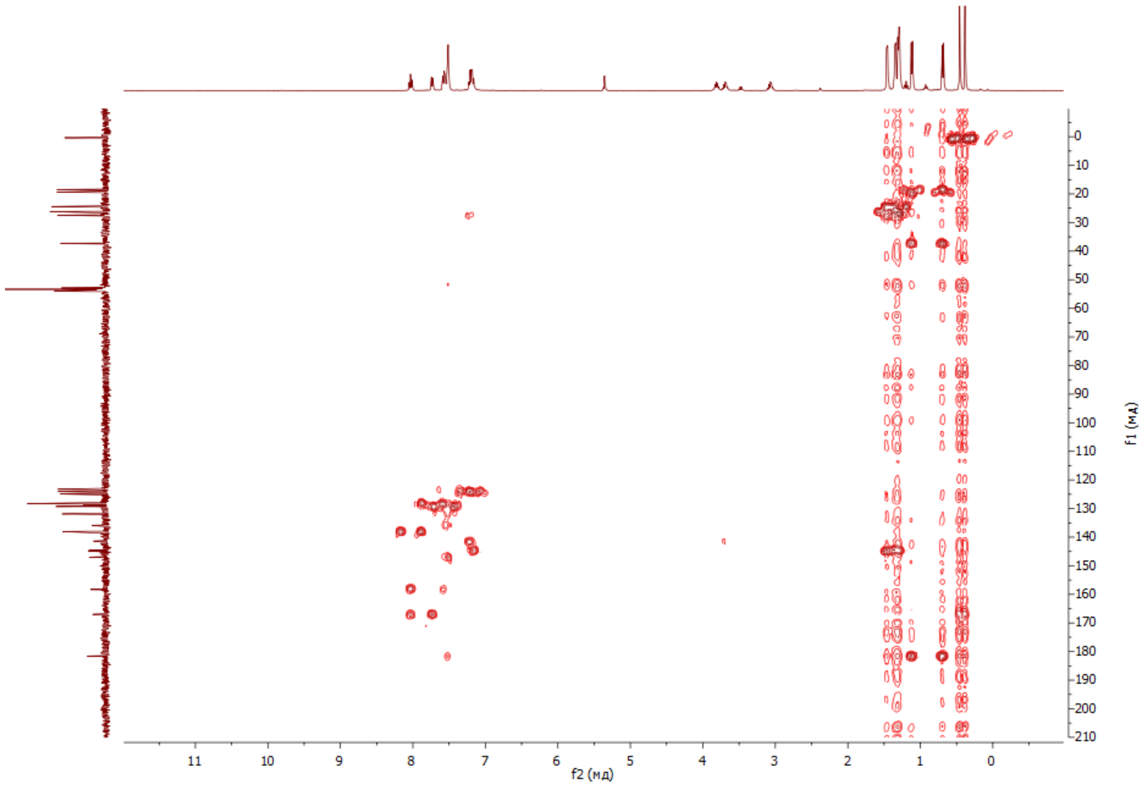

Figure S5. ^{13}C-^{1}H HMBC NMR spectrum of $\mathbf{1}^{iPrCN}$**-HfCl₂** in CD_2Cl_2 at room temperature.

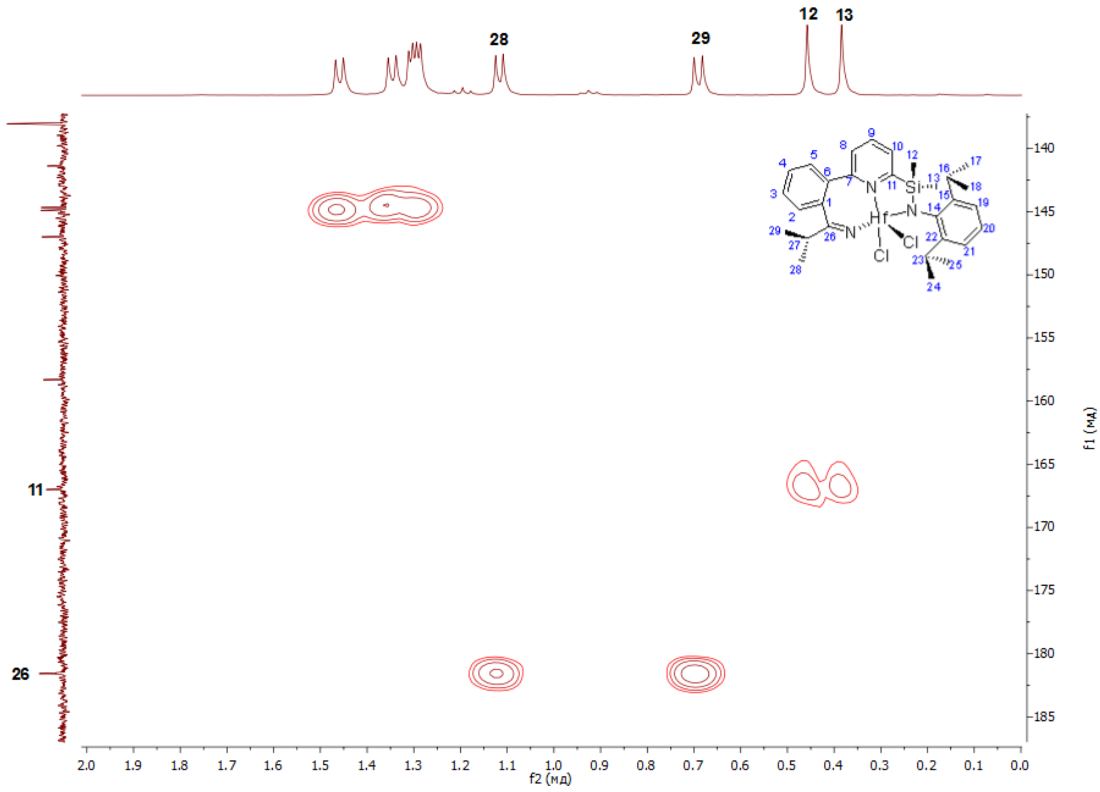

Figure S6. Fragment of ^{13}C-^{1}H HMBC NMR spectrum of $\mathbf{1}^{iPrCN}$**-HfCl₂** in CD_2Cl_2 at room temperature.

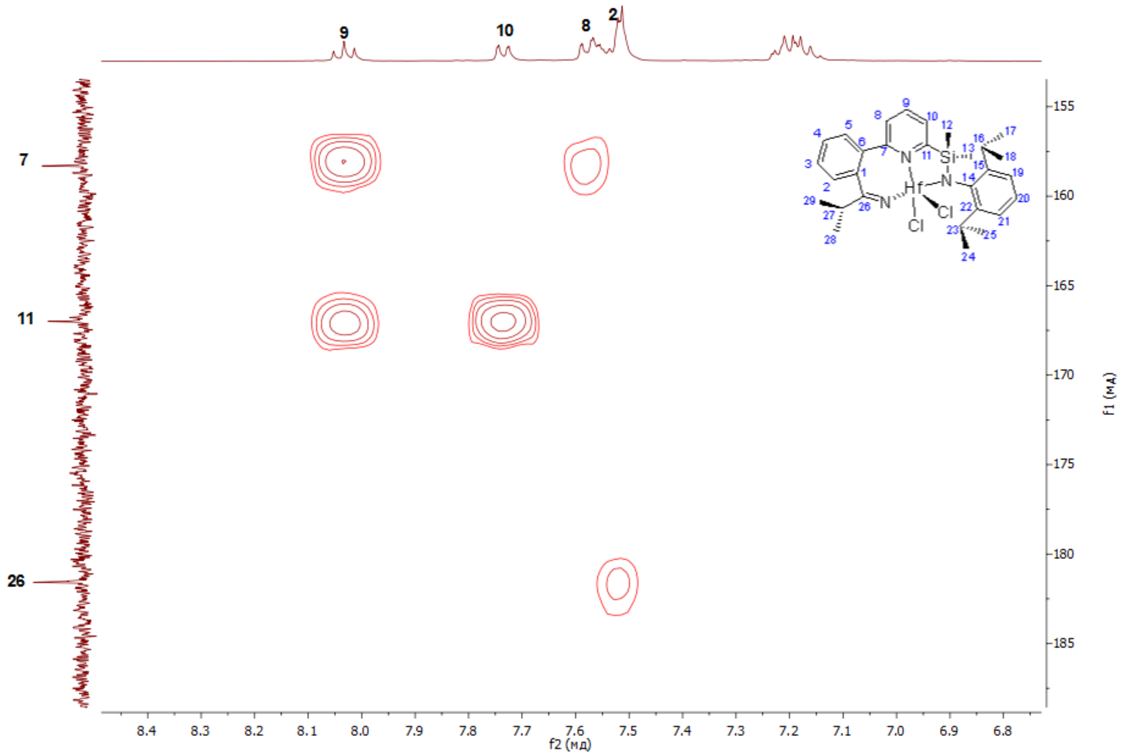

Figure S7. Fragment of ^{13}C-^{1}H HMBC NMR spectrum of $\mathbf{1}^{iPrCN}$**-HfCl$_2$** in CD_2Cl_2 at room temperature. Correlation of ketimide carbon C26 with aromatic proton H2 demonstrates the formation of bond C1–C26.

2.3. Complexes 1^{iPrCN}-HfMe(Cl/Br)

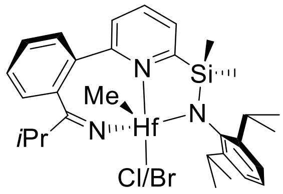

Complex **1^{iPrCN}-HfCl$_2$** (400 mg, 0.567 mmol) was dissolved in dichloromethane (20 mL) and then a solution of MeMgBr in diethyl ether (0.2 mL, 0.58 mmol, 2.9 M) was added via syringe at - 30 °C. The resulting mixture was allowed to slowly warm up to room temperature and stirred overnight. After that the volatiles were evaporated, and the residue was extracted with hot hexane by filtering the hot slurry through a short pad of Celite®. The filtrate was evaporated to dryness, and the resulting solid was washed with cold pentane giving **1^{iPrCN}-HfMe(Cl/Br)** as a pale yellow solid (309 mg, 79%).

^{1}H NMR (400 MHz, CD_2Cl_2, a mixture of **1^{iPrCN}-HfMeCl** and **1^{iPrCN}-HfMeBr**) δ_H: 7.95 (1H, m), 7.68 (1H, m), 7.38–7.55 (5H, m), 7.10–7.18 (3H, m), 3.77 (1H, m), 3.58 (1H, m), 3.04 (1H, m), 1.20–1.38 (12H, m), 1.03 (3H, m), 0.67 (3H, m), 0.43 (3H, m), 0.32 (3H, s), -0.36 (3H, m).

^{13}C{^{1}H} NMR (101 MHz, toluene-d_8, a mixture of **1^{iPrCN}-HfMeCl** and **1^{iPrCN}-HfMeBr**) δ_C: 178.3, 177.9, 168.22, 168.17, 159.4, 159.3, 148.2, 148.2, 145.23, 145.20, 144.89, 144.87, 141.5, 140.2, 136.2, 136.9, 136.8, 131.70, 131.66, 128.7, 128.6, 127.7, 127.6, 127.5, 125.6, 125.3, 124.8, 124.7, 124.54, 124.50, 123.31, 123.26, 53.1, 50.0, 37.2, 37.1, 28.09, 28.07, 27.92, 27.90, 27.2, 27.1, 26.5, 26.3, 25.5, 25.3, 25.1, 24.9, 19.8, 19.11, 19.05, 2.2, 2.1, 0.3, 0.2.

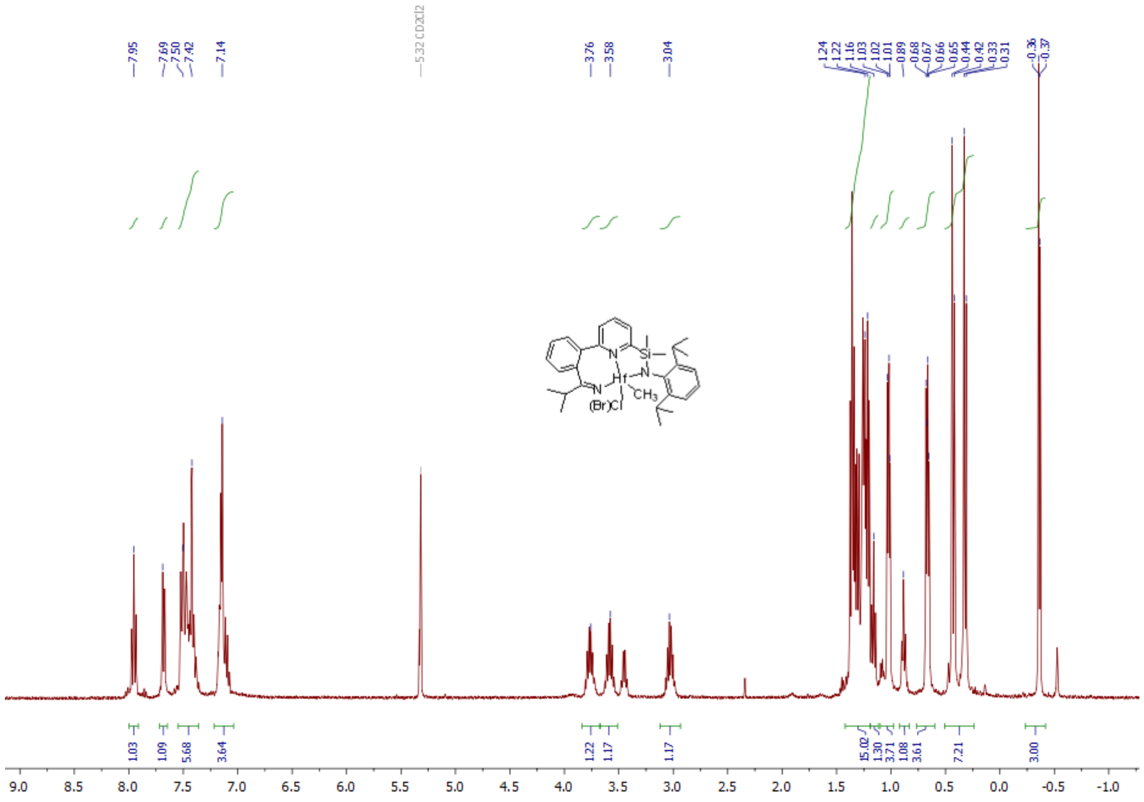

Figure S8. ^{1}H NMR spectrum of a ca. 1:1 mixture of **1**^{iPrCN}**-HfMeCl** and **1**^{iPrCN}**-HfMeBr** in CD_2Cl_2 at room temperature.

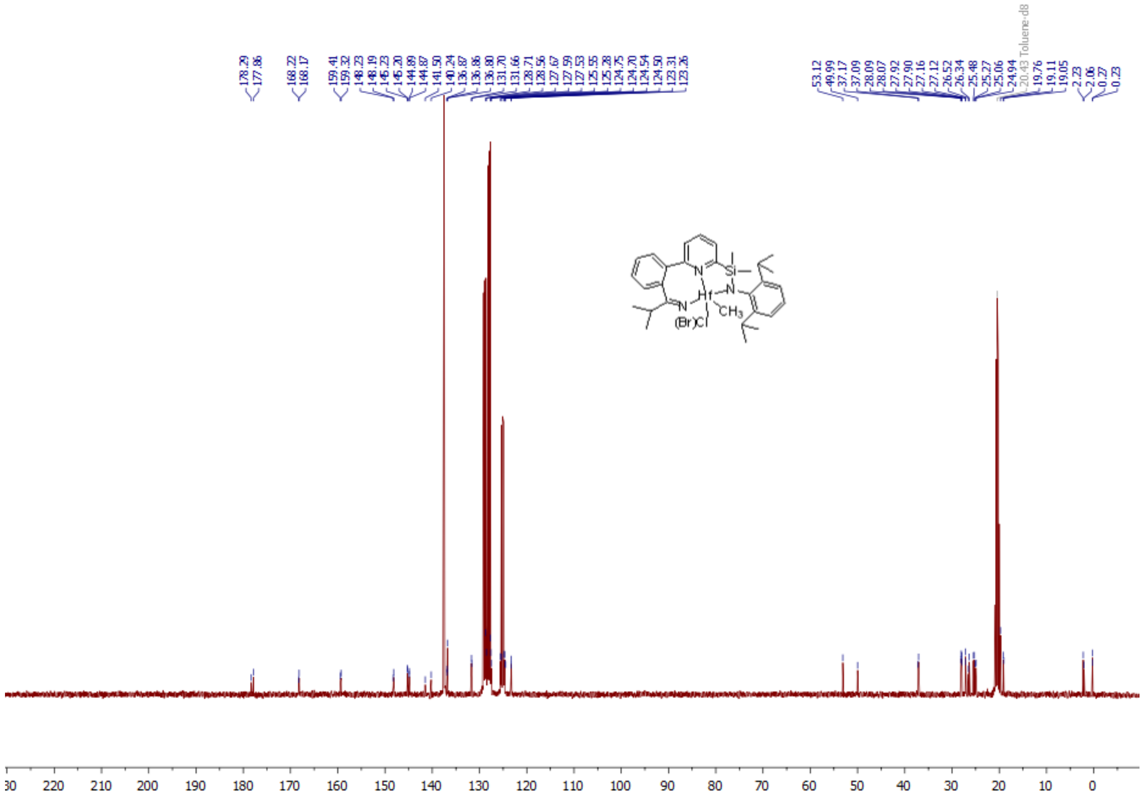

Figure S9. $^{13}C\{^{1}H\}$ NMR spectrum of a ca. 1:1 mixture of **1**^{iPrCN}**-HfMeCl** and **1**^{iPrCN}**-HfMeBr** in toluene-d_8 at room temperature.

2.4. Complex 1-HfMe$_2$

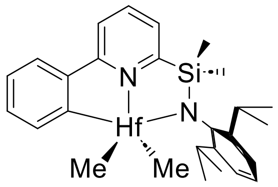

Complex **1-HfCl$_2$** (1.01 g, 1.59 mmol) was suspended in toluene (50 mL), and solution of MeMgBr in diethyl ether (1.15 mL, 3.33 mmol, 2.9 M) was added via syringe at room temperature. The resulting mixture was stirred at room temperature overnight. After that, all volatiles were evaporated, and toluene (50 mL) was added to the residue. The resulting slurry was filtered through a short pad of Celite® giving a yellow solution. The filtrate was evaporated to dryness, and the obtained residue was recrystallized from a toluene-hexane mixture giving **1-HfMe$_2$** (689 mg, 73%) as pale-yellow crystals. Anal. calc. for $C_{27}H_{36}HfN_2Si$: C, 54.49; H, 6.10; N, 4.71. Found: C, 54.60; H, 6.27; N, 4.49.

^{1}H NMR (400 MHz, toluene-d_8) δ_H: 8.34 (1H, d, $^3J_{HH}$ = 6.9 Hz), 7.48 (1H, d, $^3J_{HH}$ = 7.8 Hz, 1H), 7.32 (1H, td, $^3J_{HH}$ = 7.1 Hz, $^4J_{HH}$ = 0.8 Hz), 7.16 (1H, td, $^3J_{HH}$ = 7.5 Hz, $^4J_{HH}$ = 1.3 Hz), 7.09–7.14 (3H, m), 7.02 (2H, m), 6.88 (1H, dd, $^3J_{HH}$ = 7.2 Hz, $^4J_{HH}$ = 1.1 Hz), 3.71 (2H, m, ArC*H*(CH$_3$)$_2$), 1.27 (6H, d, $^3J_{HH}$ = 6.9 Hz, ArCH(C*H*$_3$)$_2$), 1.15 (6H, d, $^3J_{HH}$ = 6.9 Hz, ArCH(C*H*$_3$)$_2$), 0.62 (6H, s, (C*H*$_3$)$_2$Hf), 0.32 (6H, s, (C*H*$_3$)$_2$Si).

^{13}C{^{1}H} NMR (101 MHz, toluene-d_8) δ_C: 202.6 (C_{Ar}-Hf), 170.7, 164.8, 146.5, 144.3, 142.1, 140.0, 139.1, 130.5, 128.4, 126.4, 124.0, 123.8, 123.0, 117.9, 64.6, 28.1, 25.4, 24.4, 0.6 ((CH$_3$)$_2$Si).

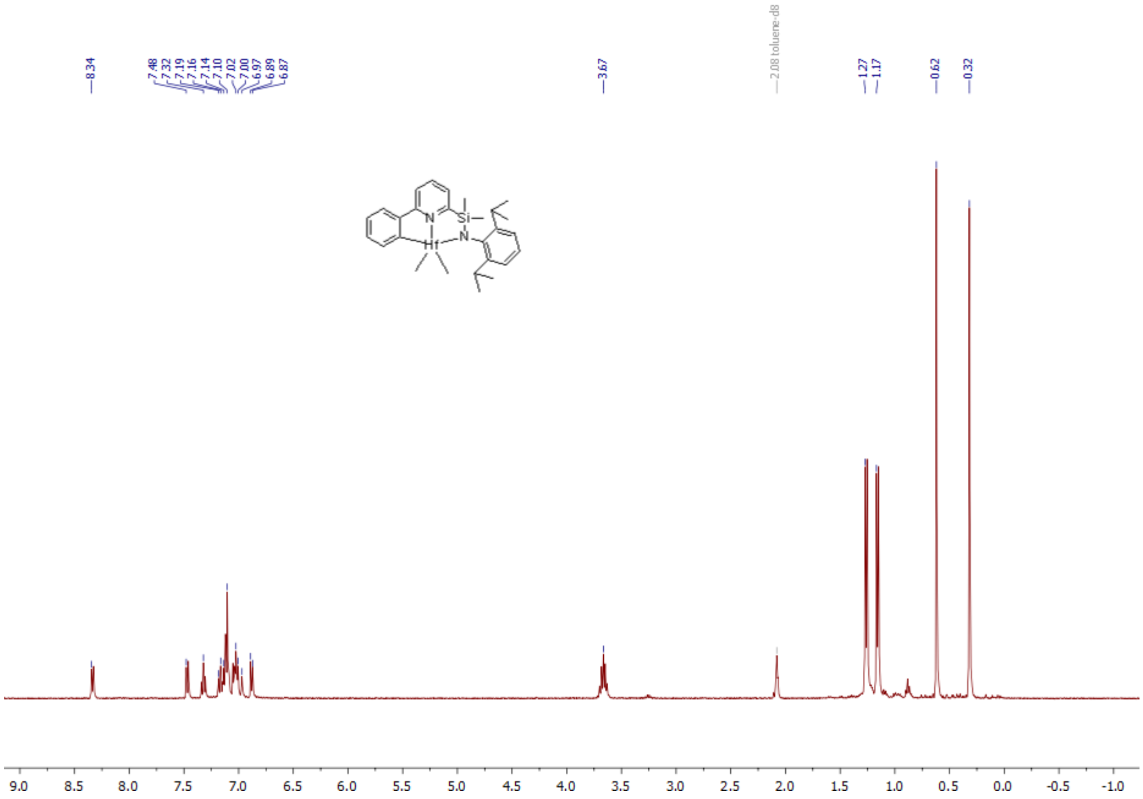

Figure S10. ^{1}H NMR spectrum of **1-HfMe$_2$** in toluene-d_8 at room temperature.

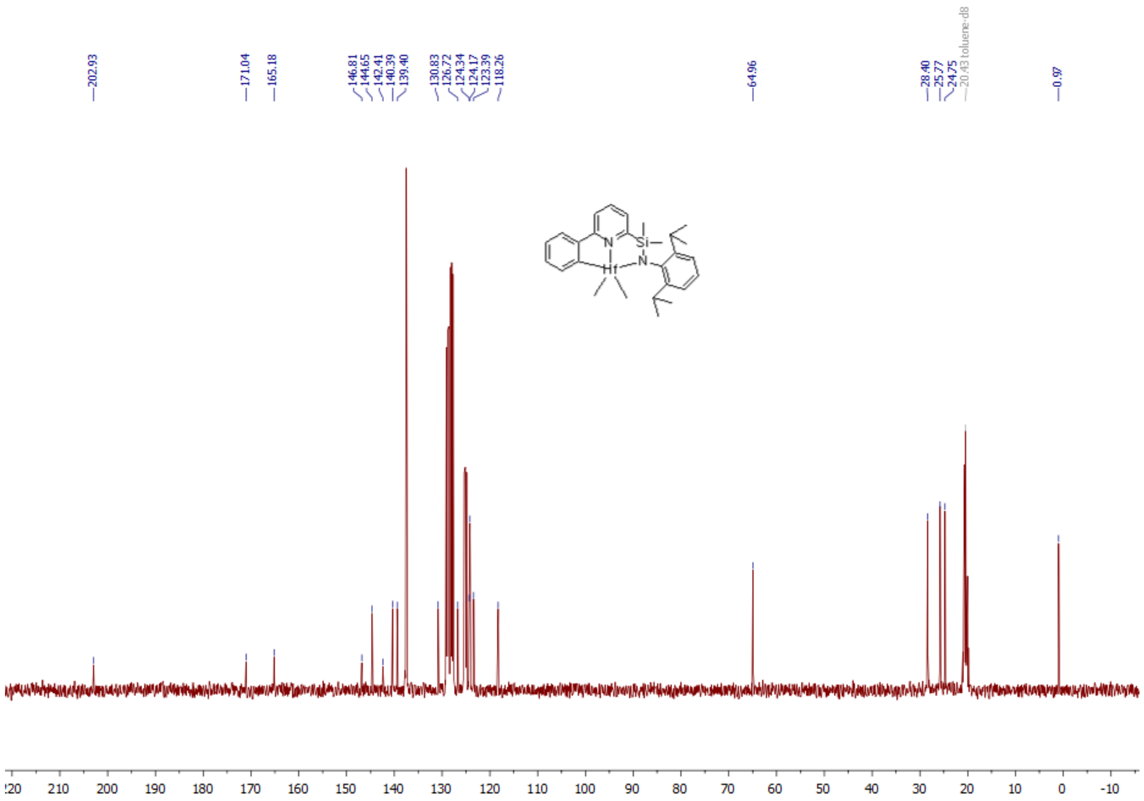

Figure S11. $^{13}C\{^{1}H\}$ NMR spectrum of **1-HfMe₂** in toluene-d_8 at room temperature.

2.5. Complex 1^{iPrCN}-HfMe$_2$

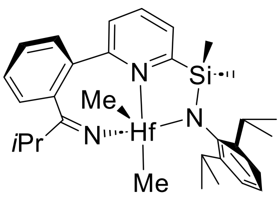

Method 1: Mixture of complexes **1^{iPrCN}-HfMe(Cl/Br)** (150 mg, 0.212 mmol) was suspended in diethyl ether (10 mL), and then solution of MeMgBr (0.08 mL, 0.232 mmol, 2.9M) in diethyl ether was added via syringe in one portion at -30 °C. The resulting mixture was allowed to slowly warmand stirred at room temperature for 2 h giving a yellowish solution with a white precipitate, then volatiles were evaporated to dryness without heating. The solid residue was extracted with hexane (60 mL) at room temperature filtering the obtained suspension through a short pad of Celite®. The filtrate was concentrated without heating until precipitation occured. The formed precipitate was filtered and washed with cold pentane (pre-chilled to -30 °C in a freezer) giving **1^{iPrCN}-HfMe$_2$** as a pale yellow solid (105 mg, 74%).

Method 2: Complex **1-HfMe$_2$** (50 mg, 75 µmol) was dissolved in toluene-d_8 (0.6 mL) in J Young NMR tube, and then isobutyronitrile (7.5 µL, 5.8 mg, 83 µmol) was added via Eppendorf pipette. After 2 h, the mixture was analyzed by NMR spectroscopy (Figure S12, bottom).

^{1}H NMR (400 MHz, toluene-d_8) δ_H: 7.19–7.21 (2H, m), 6.93–7.13 (7H, m), 6.81 (1H, dd, $^3J_{HH}$ = 7.9 Hz, $^4J_{HH}$ = 1.3 Hz), 4.13 (1H, br.s), 3.80 (1H, br.s), 2.82 (1H, sept, $^3J_{HH}$ = 6.9 Hz), 1.59 (3H, br.s), 1.52 (3H, br.s), 1.32 (6H, d, $^3J_{HH}$ = 6.9 Hz), 1.15 (3H, br.s), 0.62 (3H, br.s), 0.37 (3H, br.s), 0.23 (3H, br.s), 0.17 (3H, br.s), -0.02 (3H, br.s).

^{13}C{^{1}H} NMR (101 MHz, toluene-d_8) δ_C: 177.3, 168.5, 160.1, 148.5, 145.8, 139.0, 136.2, 131.4, 128.3, 127.8, 127.2, 127.0, 125.0, 124.6, 123.4, 64.9, 56.1, 53.3, 43.3, 37.5, 27.9, 27.0, 26.4, 25.5, 25.2, 19.7, 19.3, 2.9, 0.2.

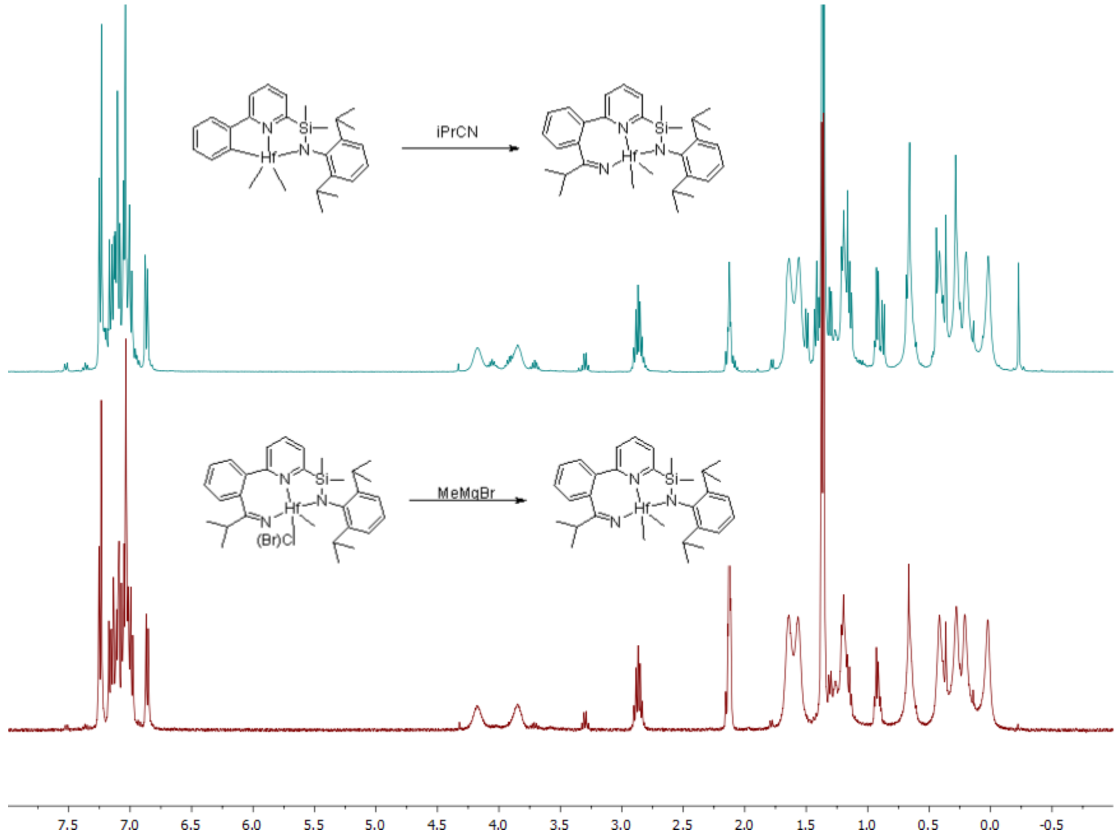

Figure S12. Comparison of ^{1}H NMR spectra (in toluene-*d*$_8$) of complex **1**^{iPrCN}**-HfMe**$_2$ obtained by two different methods: (1) via addition of *i*PrCN to **1-HfMe**$_2$ (top, green; the spectrum includes resonances attributed to complex **1**^{iPrCN}**-HfMe(N=CMe*i*Pr)** impurity); (2) via reaction of MeMgBr with **1**^{iPrCN}**-HfMe(Cl/Br)** (bottom, red).

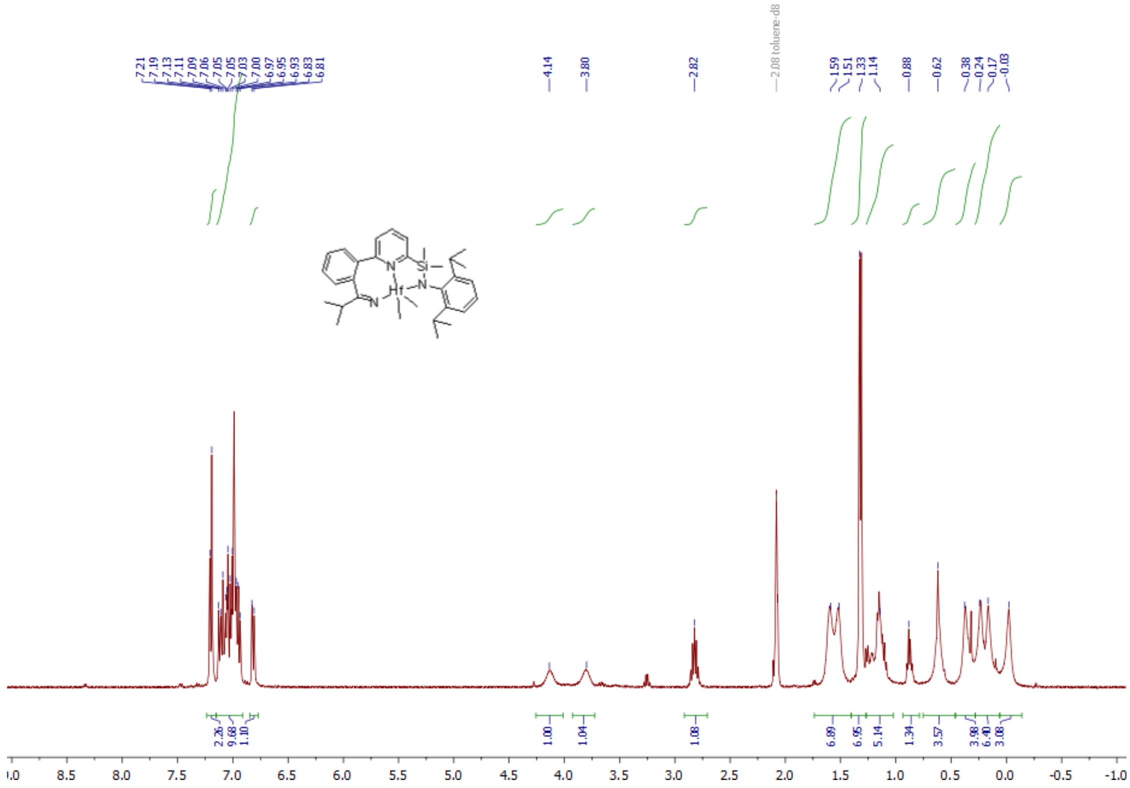

Figure S13. ^{1}H NMR spectrum of complex **1**^{iPrCN}**-HfMe**$_2$ in toluene-*d*$_8$ at room temperature.

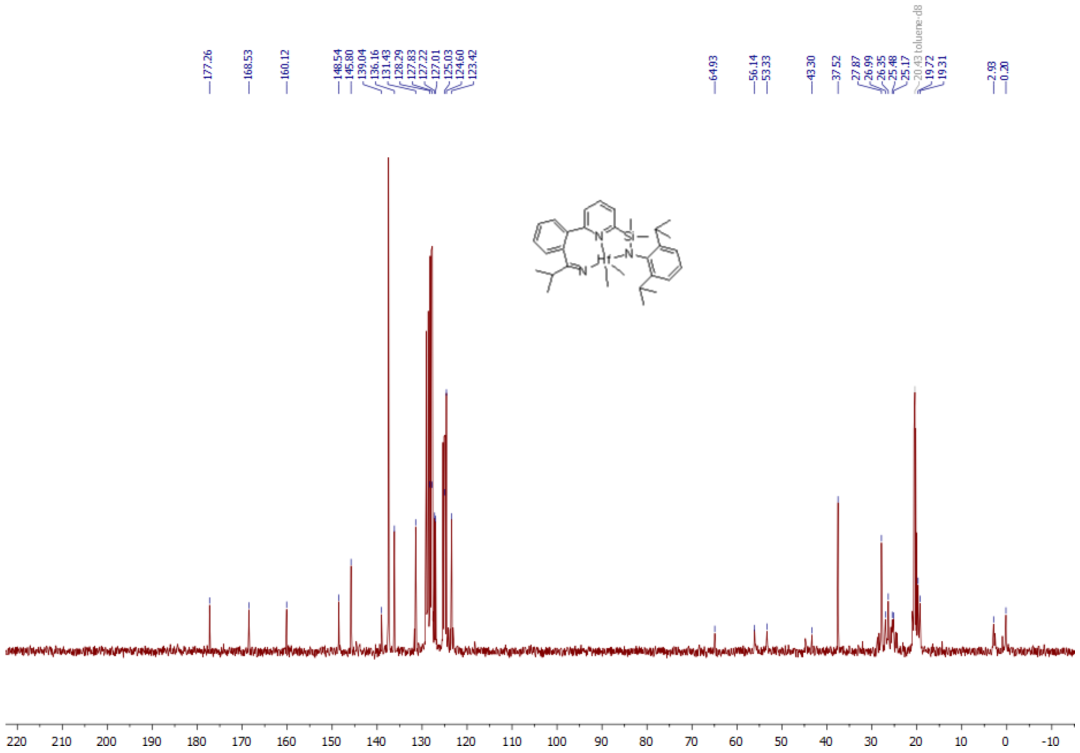

Figure S14. $^{13}C\{^{1}H\}$ NMR spectrum of complex $\mathbf{1}^{iPrCN}$-$\mathbf{HfMe_2}$ in toluene-d_8 at room temperature.

2.6. Complex 1-HfMe(N=CMe*i*Pr)

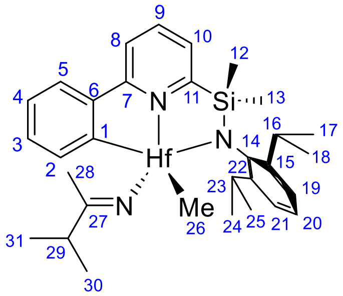

Method 1: Complex **1-HfMe$_2$** (213 mg, 0.358 mmol) was dissolved in toluene (5 mL) in 8 mL vial, and then isobutyronitrile (32.1 µL, 24.7 mg, 0.358 mmol) was added in one portion via Eppendorf pipette. The resulting mixture was stirred overnight at 100 °C in a sealed vial giving an orange solution. Thereafter, the volatiles were evaporated to dryness. The residue was recrystallized from hexane (1.3 mL) at -30 °C giving **1-HfMe(N=CMe*i*Pr)** as a yellow crystalline solid (142 mg, 60% yield). Anal. calc. for $C_{31}H_{43}HfN_3Si$: C, 56.05; H, 6.52; N, 6.33. Found: C, 56.33; H, 6.75; N, 6.07.

Method 2: Complex **1-HfMe$_2$** (25 mg, 42 µmol) was dissolved in toluene-d_8 (0.6 mL) in J Young NMR tube. Next, isobutyronitrile (3.8 µL, 2.9 mg, 42 µmol) was added in one portion via Eppendorf pipette. The resulting orange solution was heated overnight at 100 °C followed by analysis with NMR spectroscopy.

^{1}H NMR (600 MHz, toluene-d_8) δ_H: 8.22 (1H, dd, $^3J_{HH}$ = 6.8 Hz, $^4J_{HH}$ = 0.7 Hz), 7.54 (1H, d, $^3J_{HH}$ = 7.8 Hz), 7.31 (1H, m), 7.22 (1H, m), 7.17 (1H, m), 7.09–7.11 (2H, m), 7.01 (2H, m), 6.96 (1H, d, $^3J_{HH}$ = 7.6 Hz), 3.89 (1H, sept, $^3J_{HH}$ = 6.8 Hz, C(16)H), 3.32 (1H, sept, $^3J_{HH}$ = 6.9 Hz, C(23)H), 2.18 (1H, sept, $^3J_{HH}$ = 6.9 Hz, C(29)H), 1.31 (3H, d, $^3J_{HH}$ = 6.8 Hz, C(17)H), 1.30 (3H, d, $^3J_{HH}$ = 6.8 Hz, C(18)H), 1.29 (3H, s, C(28)H), 1.04 (3H, d, $^3J_{HH}$ = 6.9 Hz, C(24)H), 1.02 (3H, d, $^3J_{HH}$ = 6.9 Hz, C(30)H), 1.00 (3H, d, $^3J_{HH}$ = 6.9 Hz, C(31)H), 0.98 (3H, d, $^3J_{HH}$ = 6.9 Hz, C(25)H), 0.61 (3H, s, C(12)H), 0.43 (3H, s, C(26)H), 0.14 (3H, s, C(13)H).

^{13}C{^{1}H} NMR (150 MHz, toluene-d_8) δ_C: 200.8 (C-1), 190.5, 171.1, 164.8, 147.0, 145.6, 143.8, 141.4, 139.0, 130.1, 128.2, 126.7, 123.8, 123.34, 123.28, 122.9, 118.2, 51.0, 44.7, 28.8, 28.5, 27.7, 26.5, 25.6, 25.3, 23.2, 20.1, 19.9, 2.5, -1.1.

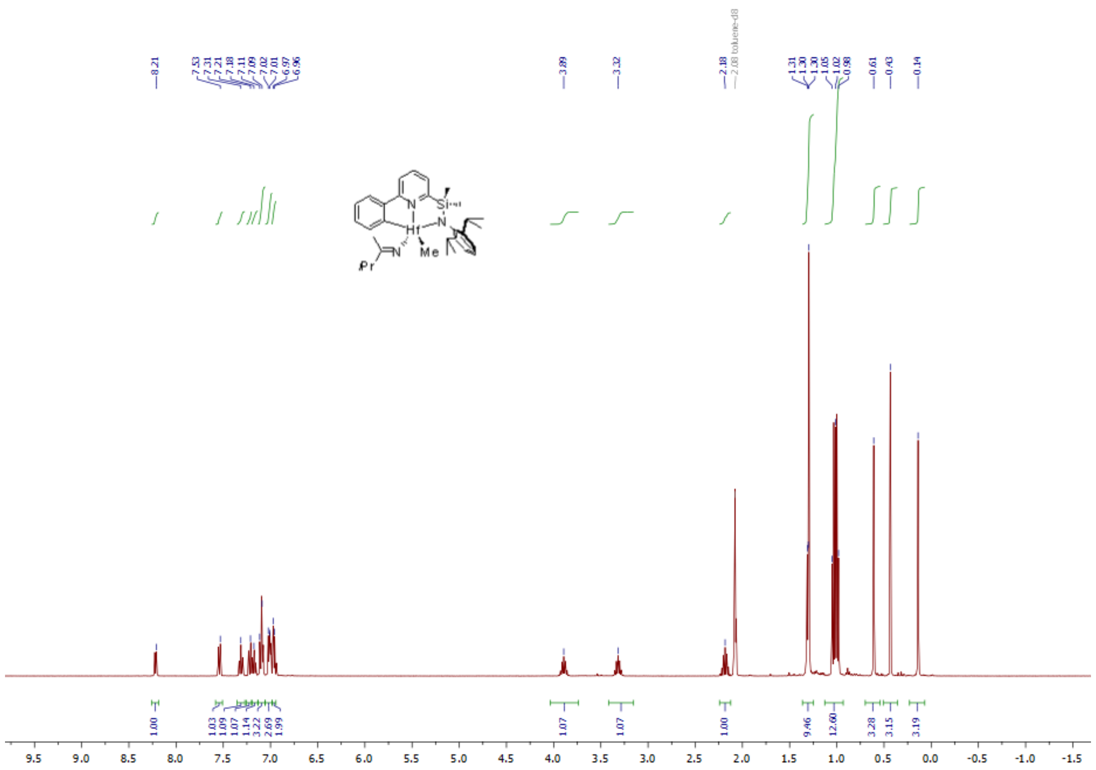

Figure S15. ^{1}H NMR spectrum of complex **1-HfMe(N=CMe*i*Pr)** obtained via method 1 in toluene-d_8 at room temperature.

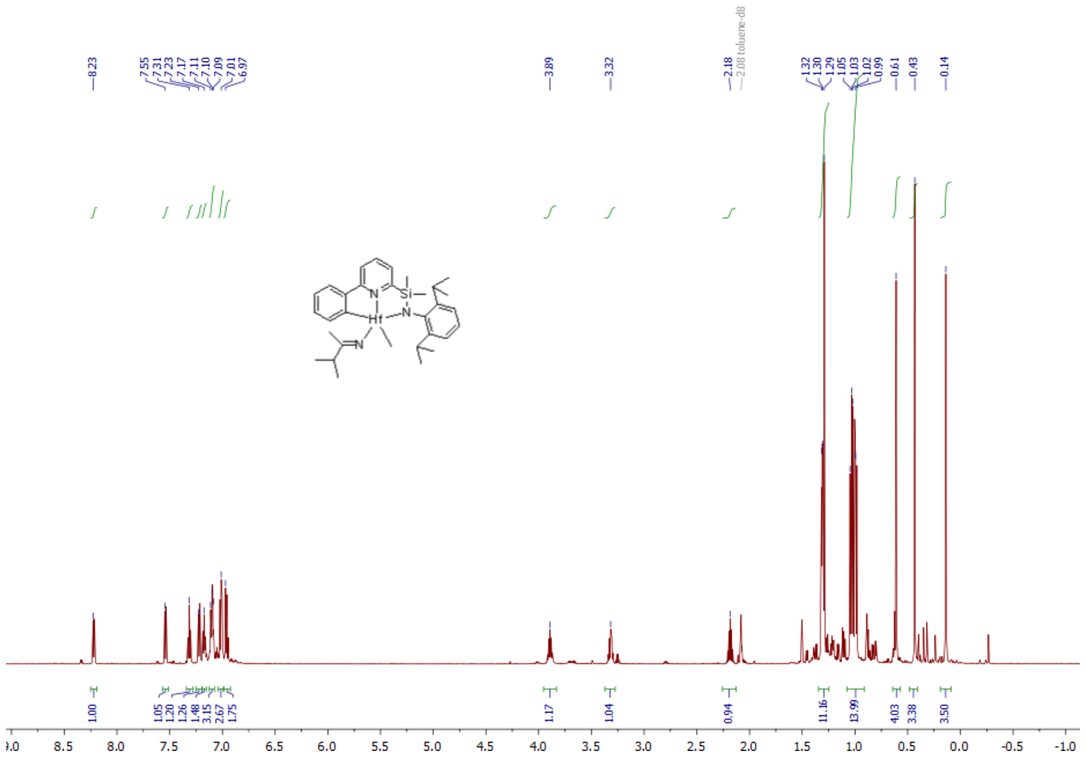

Figure S16. ^{1}H NMR spectrum of complex **1-HfMe(N=CMe*i*Pr)** obtained via method 2 in toluene-d_8 at room temperature.

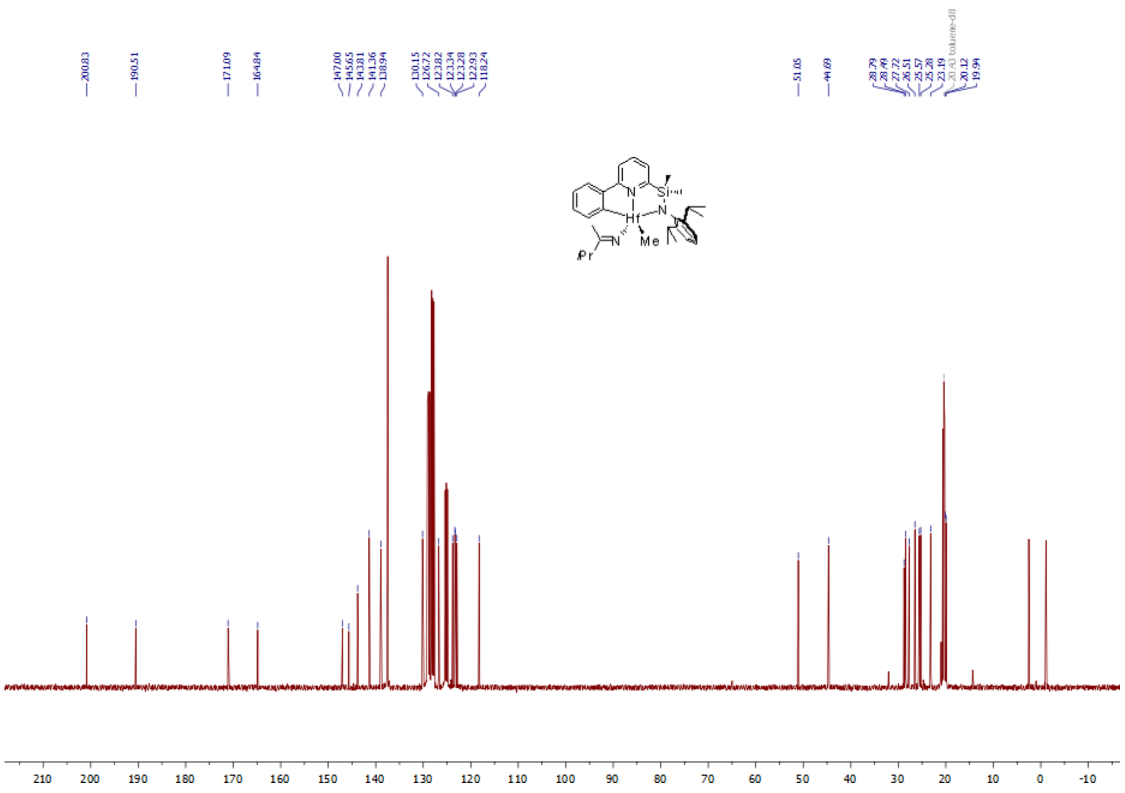

Figure S17. $^{13}C\{^{1}H\}$ NMR spectrum of complex **1-HfMe(N=CMe*i*Pr)** obtained by method 1 in toluene-d_8 at room temperature.

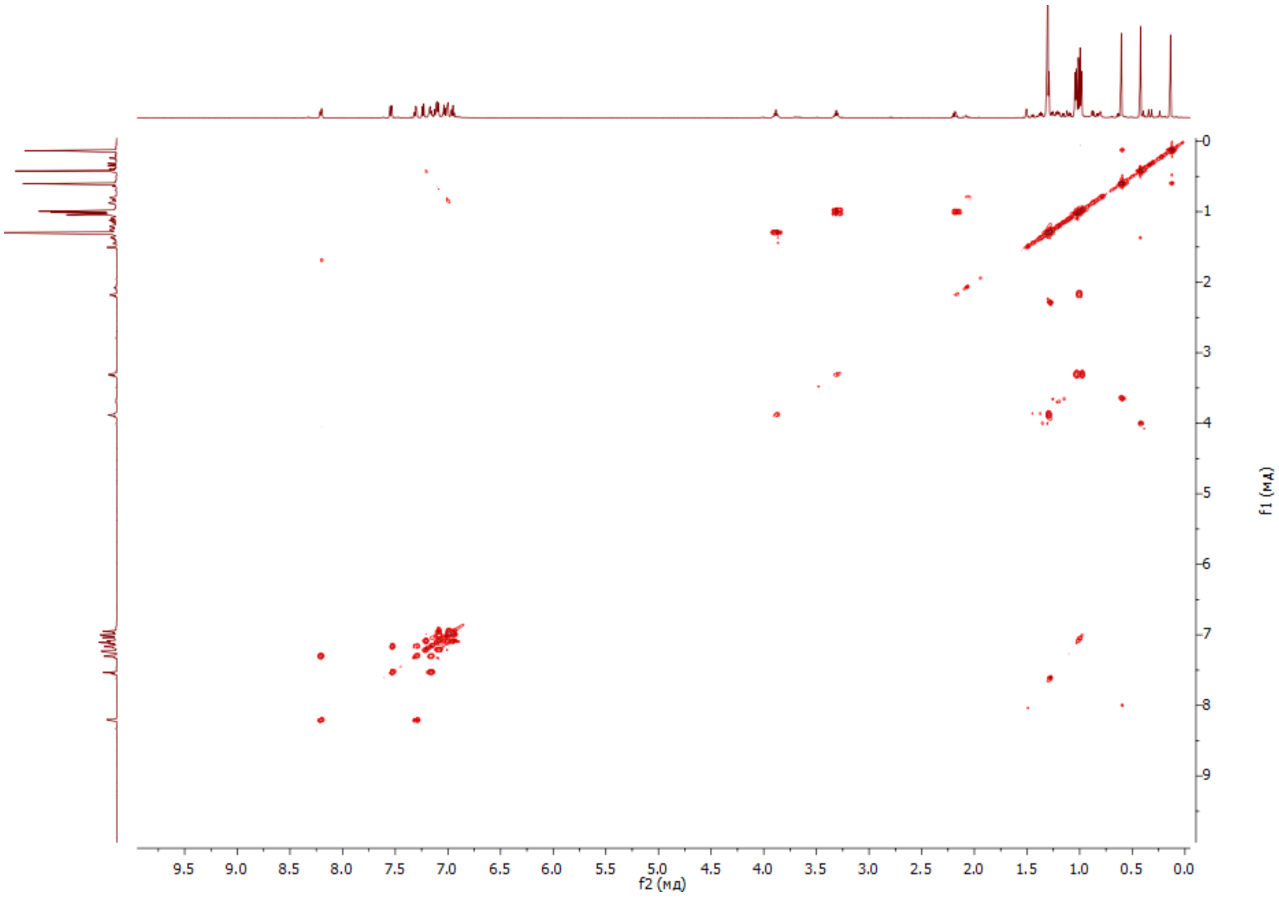

Figure S18. ^{1}H-^{1}H COSY NMR spectrum of complex **1-HfMe(N=CMe*i*Pr)** in toluene-d_8 at room temperature.

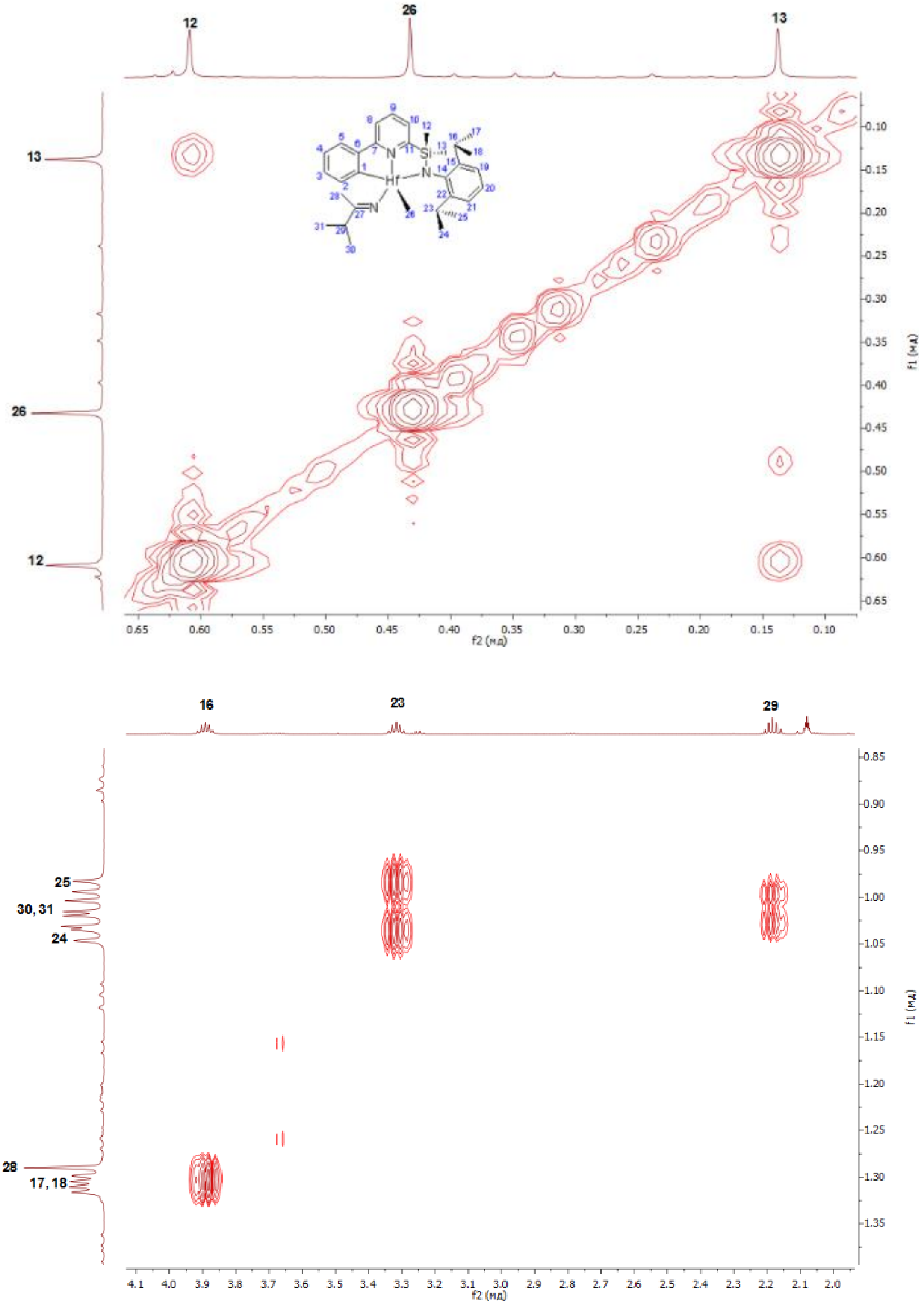

Figure S19. Fragments of ^{1}H-^{1}H COSY NMR spectrum of complex **1-HfMe(N=CMe*i*Pr)** in toluene-d_8 at room temperature.

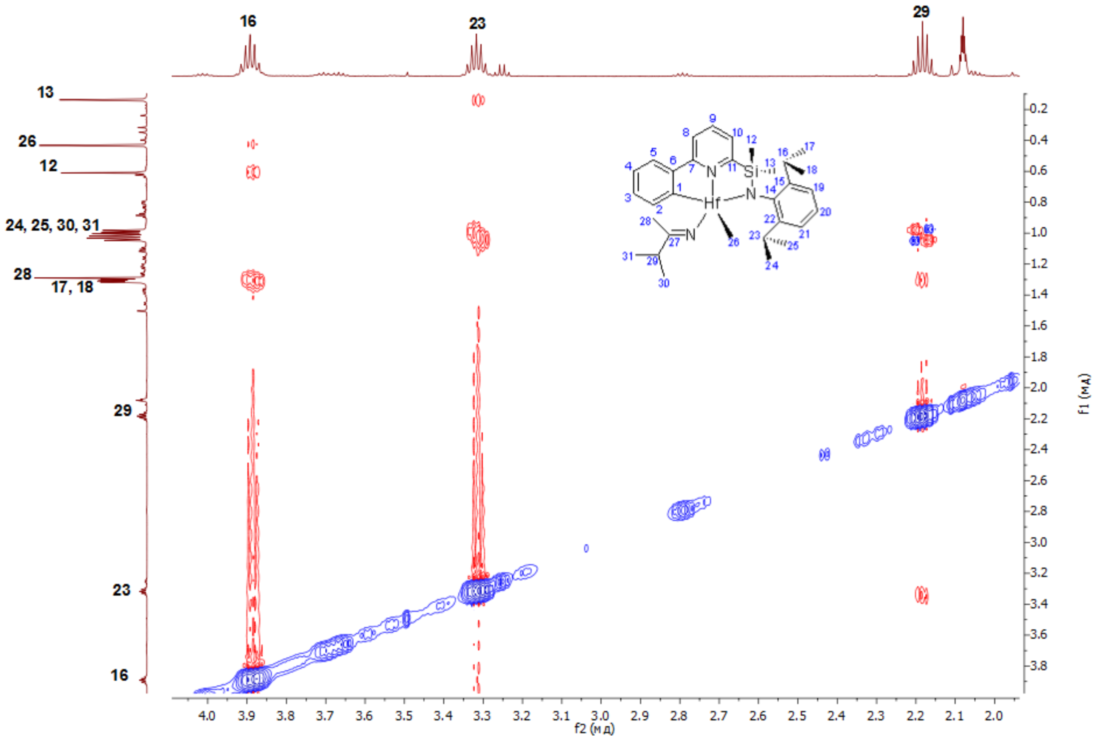

Figure S20. Fragment of ^{1}H-^{1}H ROESY NMR spectrum of complex **1-HfMe(N=CMe*i*Pr)** in toluene-d_8 at room temperature.

2.7. Complex 1^{iPrCN}-HfMe(N=CMe*i*Pr)

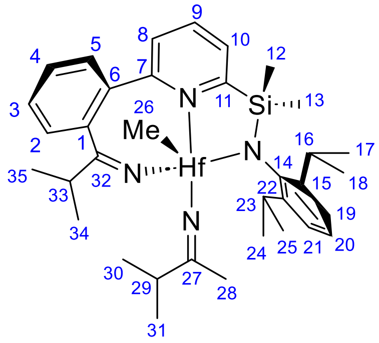

<u>Method 1:</u> Complex **1-HfMe₂** (180 mg, 0.302 mmol) was dissolved in toluene (5 mL) in 8 mL vial, and then isobutyronitrile (54.2 µL, 41.7 mg, 0.604 mmol) was added in one portion via Eppendorf pipette. This mixture was stirred overnight at room temperature giving orange solution. Thereafter, volatiles were evaporated to dryness, and the residue was recrystallized from hexane (2.0 mL) at -30 °C giving **1^{iPrCN}-HfMe(N=CMe*i*Pr)** as a yellow crystalline solid (128 mg, 58% yield). Anal. calc. for $C_{35}H_{50}HfN_4Si$: C, 57.32; H, 6.87; N, 7.64. Found: C, 57.61; H, 7.11; N, 7.46.

<u>Method 2:</u> Complex **1-HfMe₂** (25 mg, 42 µmol) was dissolved in toluene-*d₈* (0.6 mL) in J Young NMR tube. Then, isobutyronitrile (7.5 µL, 5.8 mg, 84 µmol) was added in one portion via Eppendorf pipette. The resulting orange solution analyzed by NMR after 1 h (Figure S34) and then was heated overnight at 100 °C followed by analysis with NMR spectroscopy.

^{1}H NMR (600 MHz, toluene-d_8) δ_H: 7.14–7.18 (4H, m, C(21)H, C(2)H, C(19)H, C(9)H), 7.07–7.10 (2H, m, C(5)H and C(10)H), 7.03–7.07 (2H, m, C(3)H and C(20)H), 6.98–7.03 (1H, m, C(4)H), 6.93 (1H, m, C(8)H), 3.99 (1H, sept, $^3J_{HH}$ = 6.8 Hz, C(16)H), 3.85 (1H, sept, $^3J_{HH}$ = 6.7 Hz, C(23)H), 2.78 (1H, sept, $^3J_{HH}$ = 6.9 Hz, C(33)H), 2.05 (1H, sept, $^3J_{HH}$ = 6.8 Hz, C(29)H), 1.42 (3H, d, $^3J_{HH}$ = 6.7 Hz, C(24)H), 1.35 (3H, d, $^3J_{HH}$ = 6.9 Hz, C(25)H), 1.34 (3H, d, $^3J_{HH}$ = 6.9 Hz, C(17)H), 1.29 (3H, d, $^3J_{HH}$ = 6.8 Hz, C(18)H), 1.12 (3H, s, C(28)H), 1.08 (3H, d, $^3J_{HH}$ = 6.7 Hz, C(34)H), 0.86 (3H, d, $^3J_{HH}$ = 6.8 Hz, C(30)H), 0.81 (3H, d, $^3J_{HH}$ = 6.8 Hz, C(31)H), 0.63 (3H, d, $^3J_{HH}$ = 6.9 Hz, C(35)H), 0.39 (3H, s, C(12)H), 0.24 (3H, s, C(13)H), –0.31 (3H, s, C(26)H).

^{13}C{^{1}H} NMR (150 MHz, toluene-d_8): δ_C: 186.0 (C-27), 174.1 (C-32), 168.7 (C-11), 159.7 (C-7), 148.6 (C-1), 145.9 (C-14), 144.0 (C-22), 143.8 (C-15), 137.2 (C-6), 136.3 (C-9), 131.7 (C-5), 128.4 (C-3), 127.6 (C-10), 127.5 (C-8), 126.9 (C-4), 123.9 (C-21), 123.8 (C-19), 123.12 (C-2), 123.05 (C-20), 44.8 (C-26), 43.3 (C-29), 37.5 (C-33), 28.7 (C-28), 27.9 (C-23), 27.7 (C-16), 26.8 (C-24), 26.4 (C-18), 24.6 (C-17), 24.4 (C-25), 19.9 (C-35), 19.7 (C-30 and C-31), 19.3 (C-34), 2.6 (C-12), 0.2 (C-13).

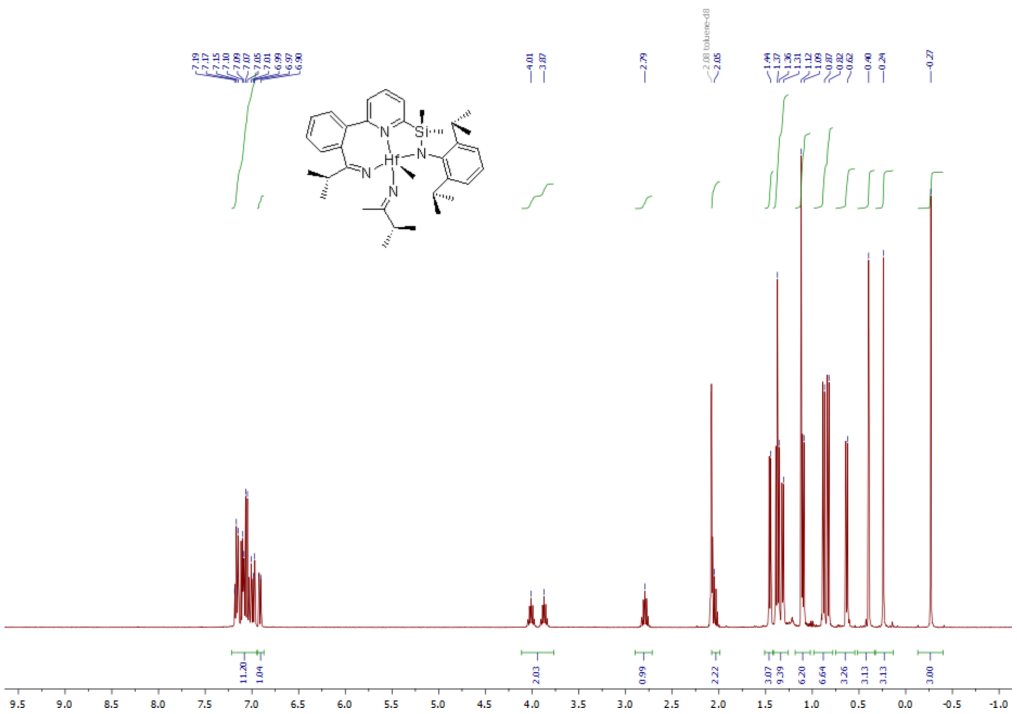

Figure S21. ^{1}H NMR spectrum of complex **1^{iPrCN}-HfMe(N=CMe*i*Pr)** (obtained via method 1) in toluene-d_8 at room temperature.

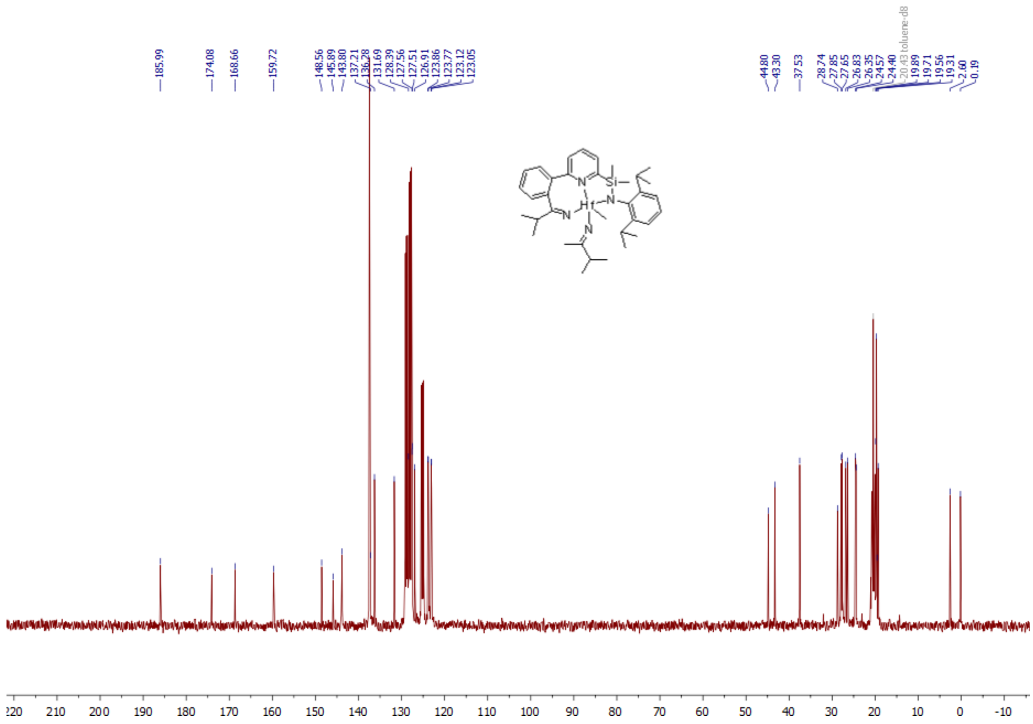

Figure S22. ^{13}C{^{1}H} NMR spectrum of complex **1^{iPrCN}-HfMe(N=CMe*i*Pr)** (obtained via method 1) in toluene-d_8 at room temperature.

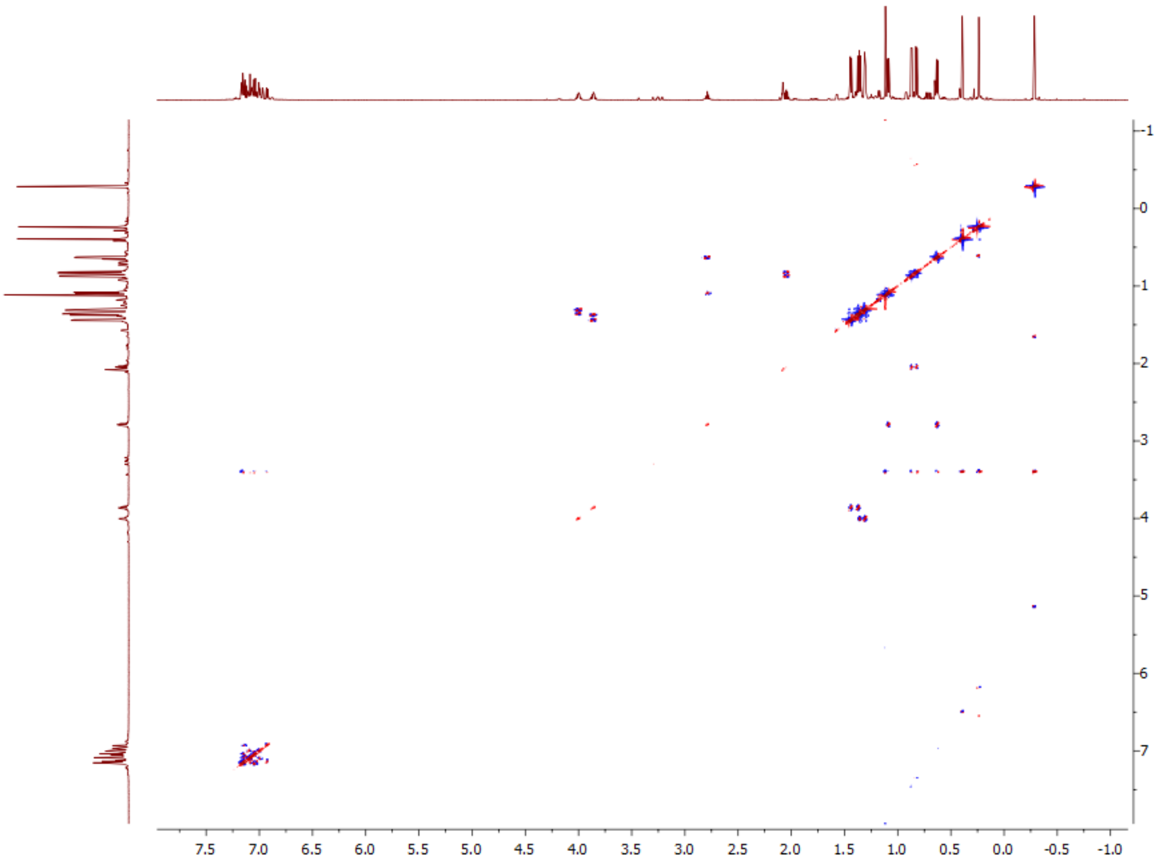

Figure S23. ^{1}H-^{1}H COSY NMR spectrum of **1**^{iPrCN}**-HfMe(N=CMe*i*Pr)** in toluene-d_8 at room temperature.

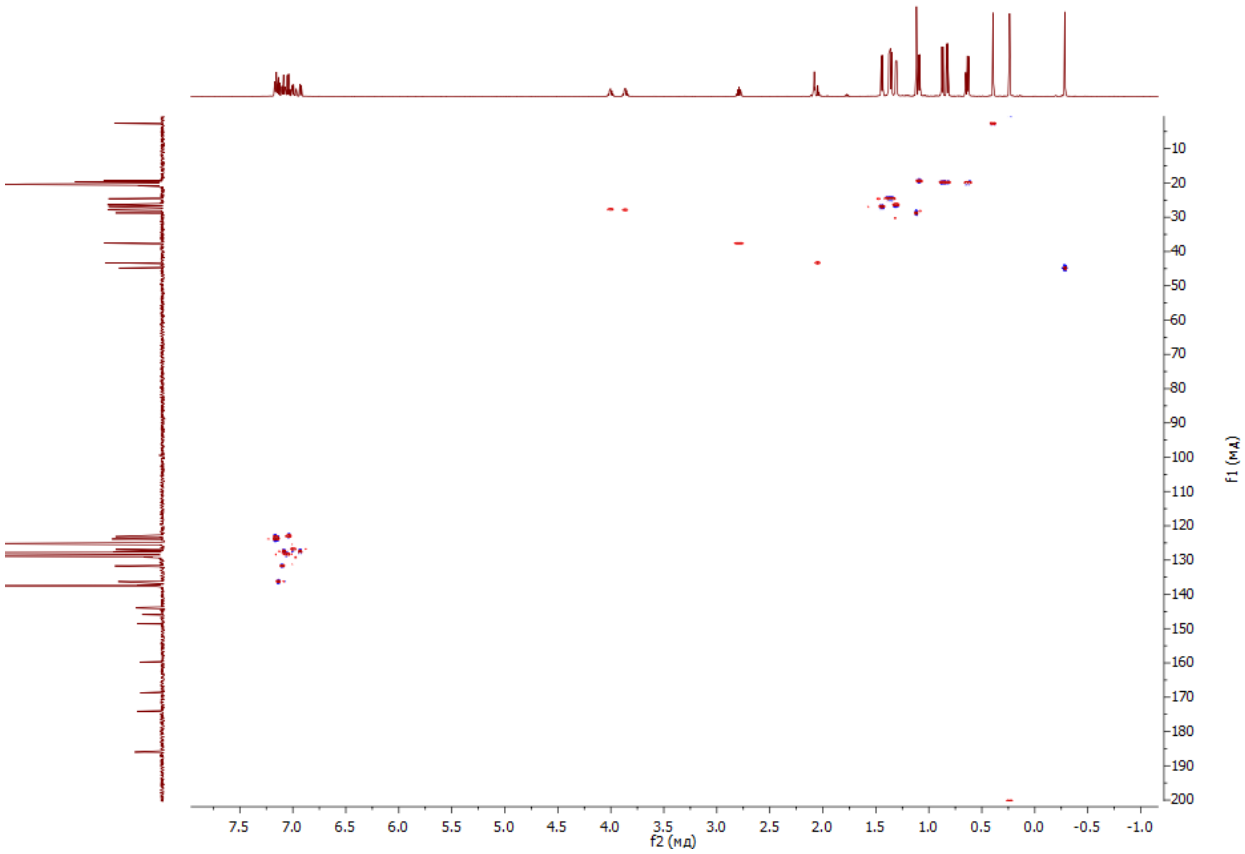

Figure S24. ^{1}H-^{13}C HSQC NMR spectrum of complex **1**^{iPrCN}**-HfMe(N=CMe*i*Pr)** in toluene-d_8 at room temperature.

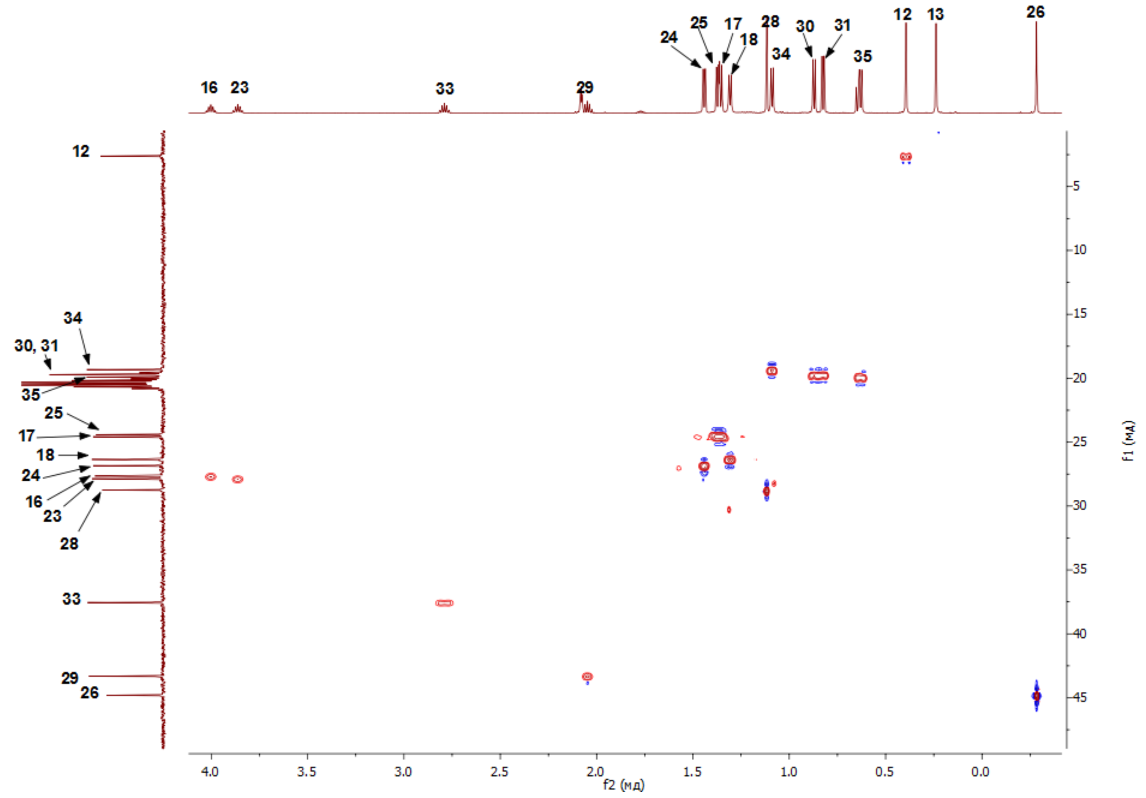

Figure S25. Fragment of ^{1}H-^{13}C HSQC NMR spectrum of complex **1^{iPrCN}-HfMe(N=CMe*i*Pr)** in toluene-d_8 at room temperature.

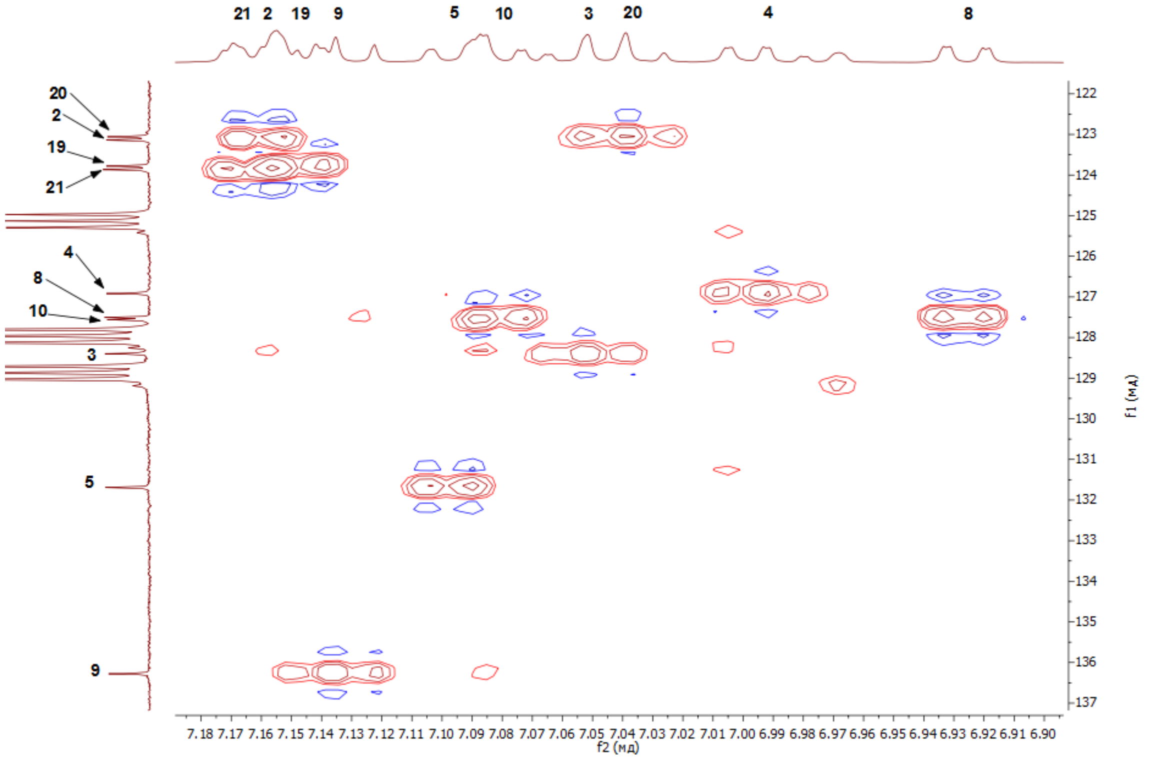

Figure S26. Fragment of ^{1}H-^{13}C HSQC NMR spectrum of complex **1^{iPrCN}-HfMe(N=CMe*i*Pr)** in toluene-d_8 at room temperature.

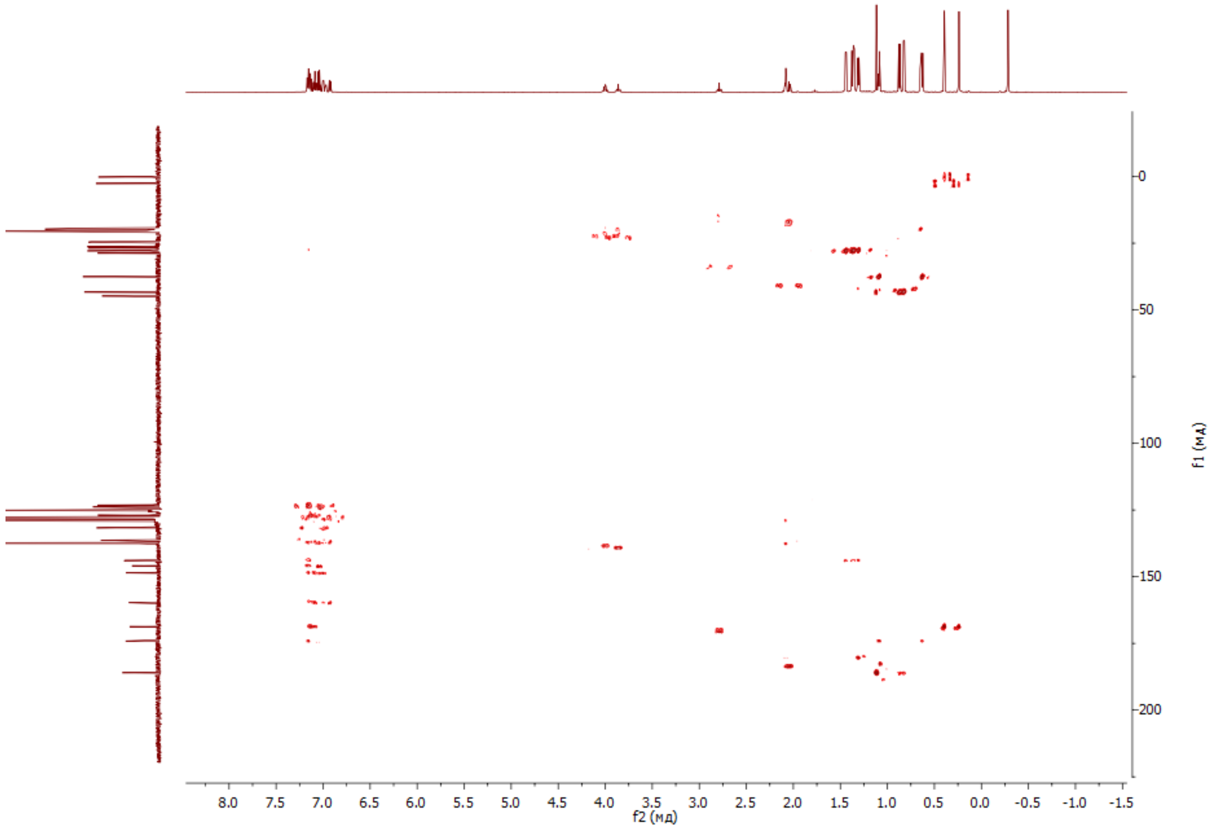

Figure S27. ^{1}H-^{13}C HMBC NMR spectrum of complex **1^{iPrCN}-HfMe(N=CMe*i*Pr)** in toluene-*d$_8$* at room temperature.

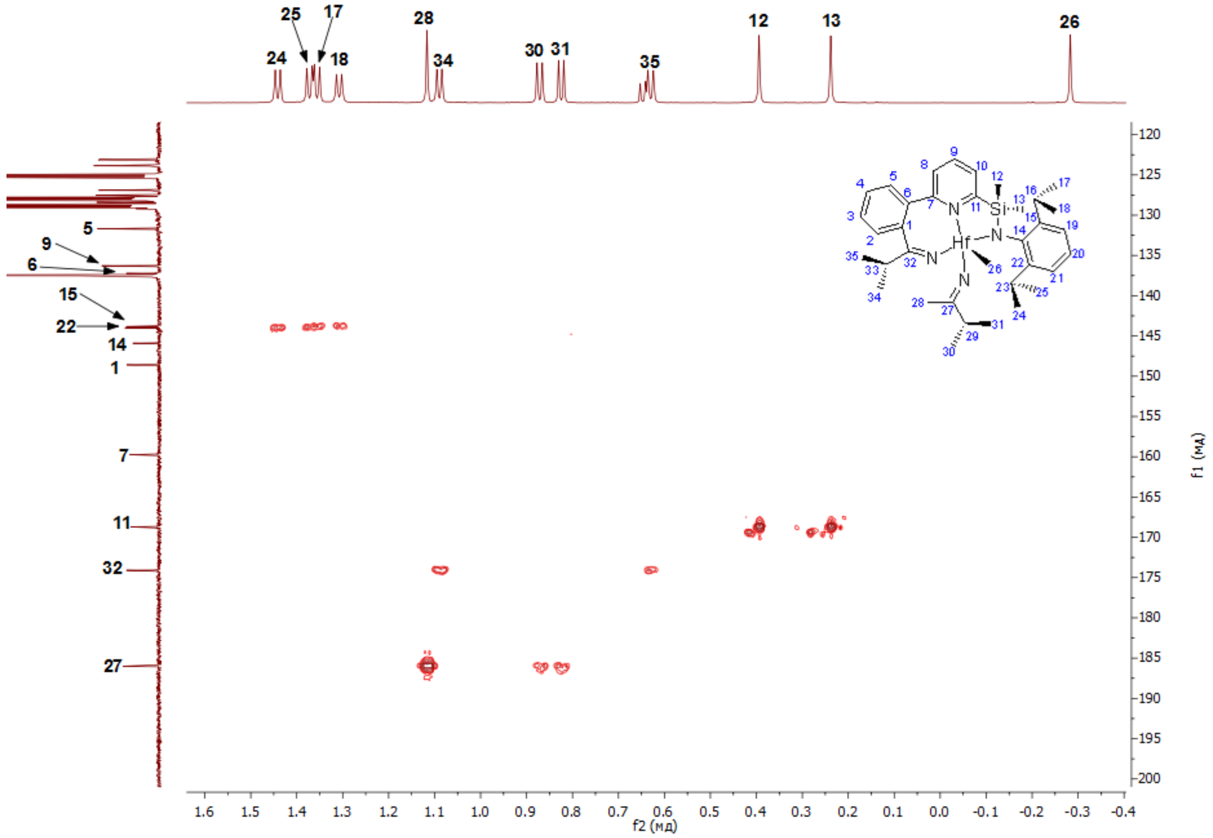

Figure S28. Fragment of ^{1}H-^{13}C HMBC NMR spectrum of complex **1^{iPrCN}-HfMe(N=CMe*i*Pr)** in toluene-*d$_8$* at room temperature.

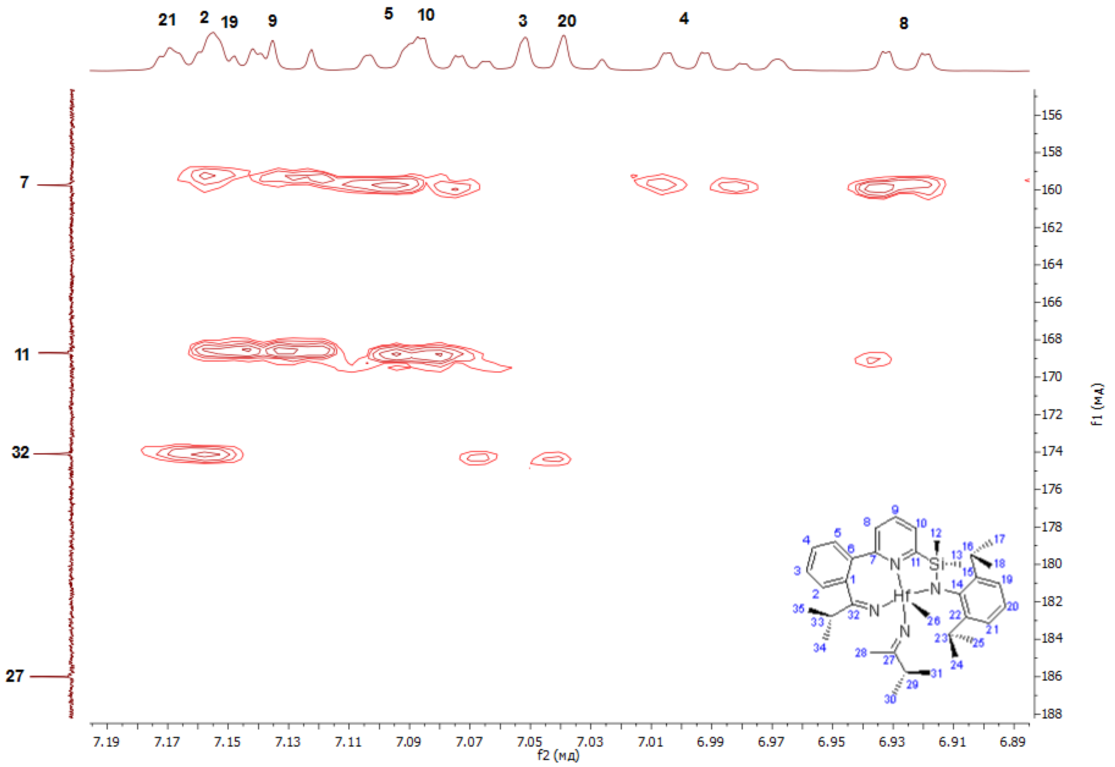

Figure S29. Fragment of ^{1}H-^{13}C HMBC NMR spectrum of complex **1^{iPrCN}-HfMe(N=CMe*i*Pr)** in toluene-d_8 at room temperature.

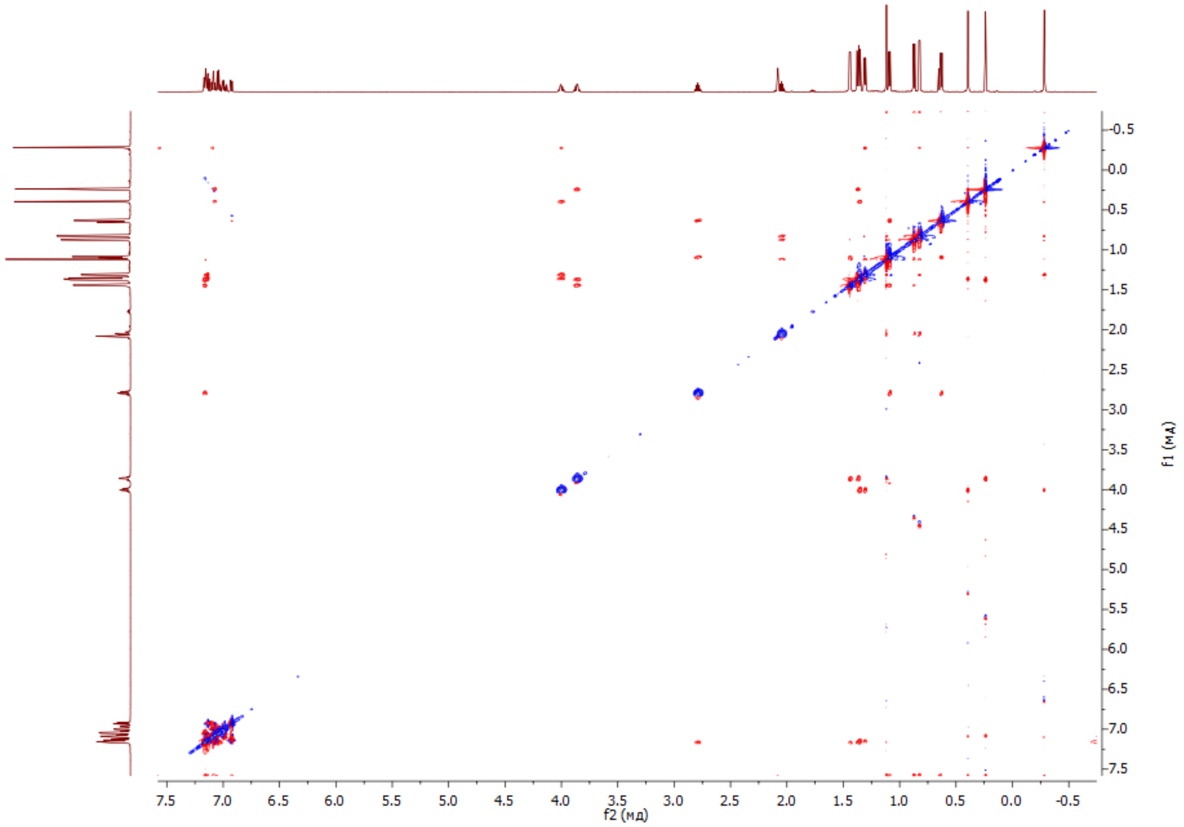

Figure S30. ^{1}H-^{1}H ROESY NMR spectrum of **1^{iPrCN}-HfMe(N=CMe*i*Pr)** in toluene-d_8 at room temperature.

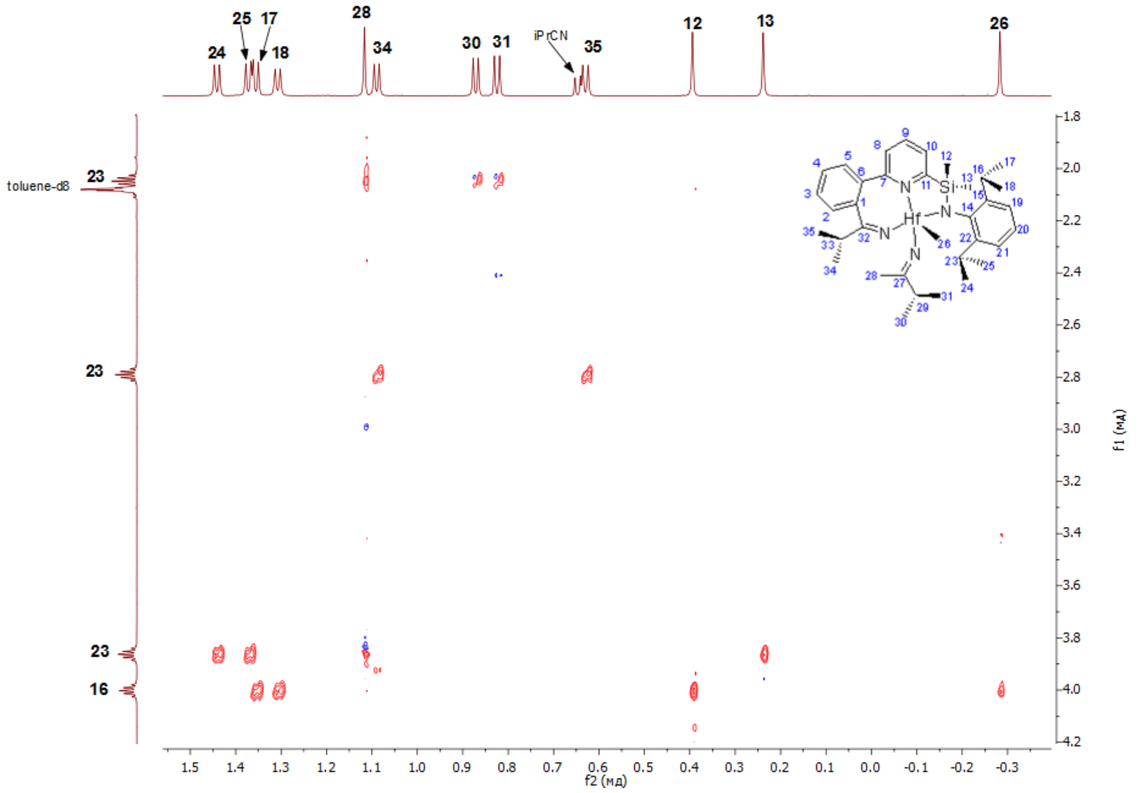

Figure S31. Fragment of ^{1}H-^{1}H ROESY NMR spectrum of **1^{iPrCN}-HfMe(N=CMe*i*Pr)** in toluene-d_8 at room temperature.

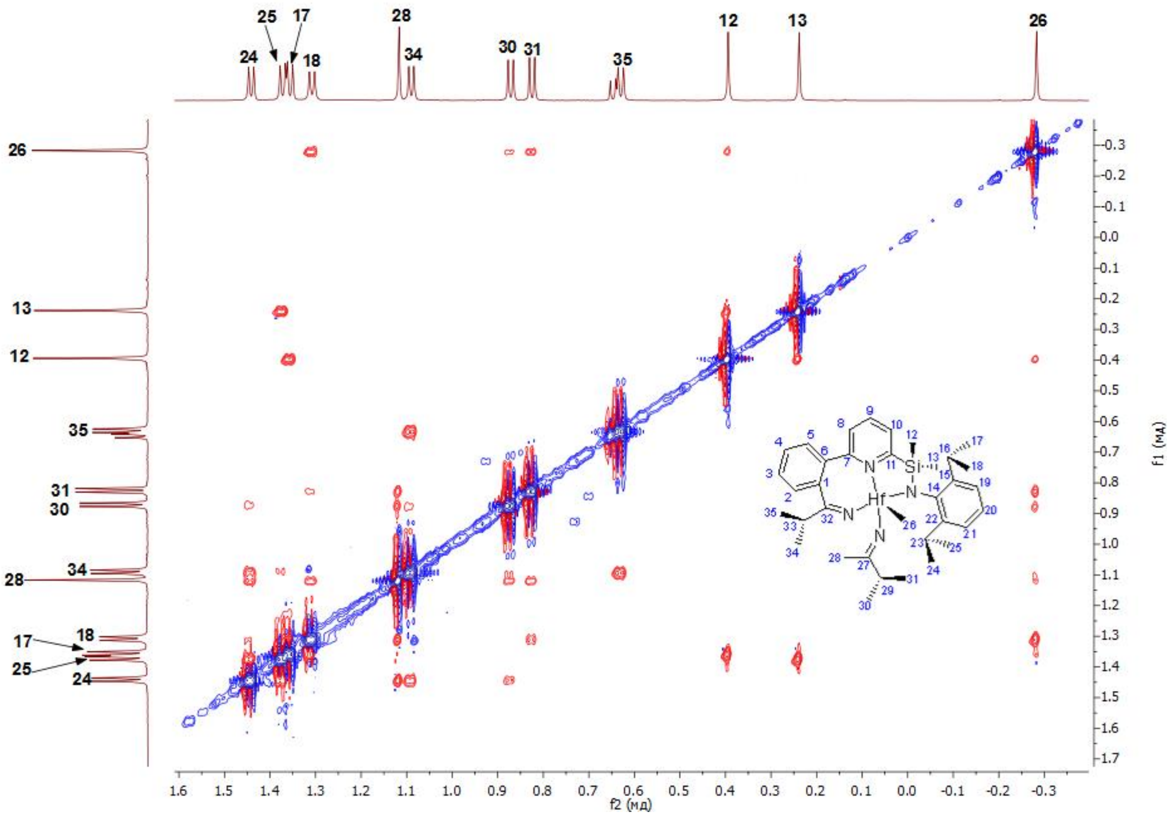

Figure S32. Fragment of ^{1}H-^{1}H ROESY NMR spectrum of **1^{iPrCN}-HfMe(N=CMe*i*Pr)** in toluene-d_8 at room temperature.

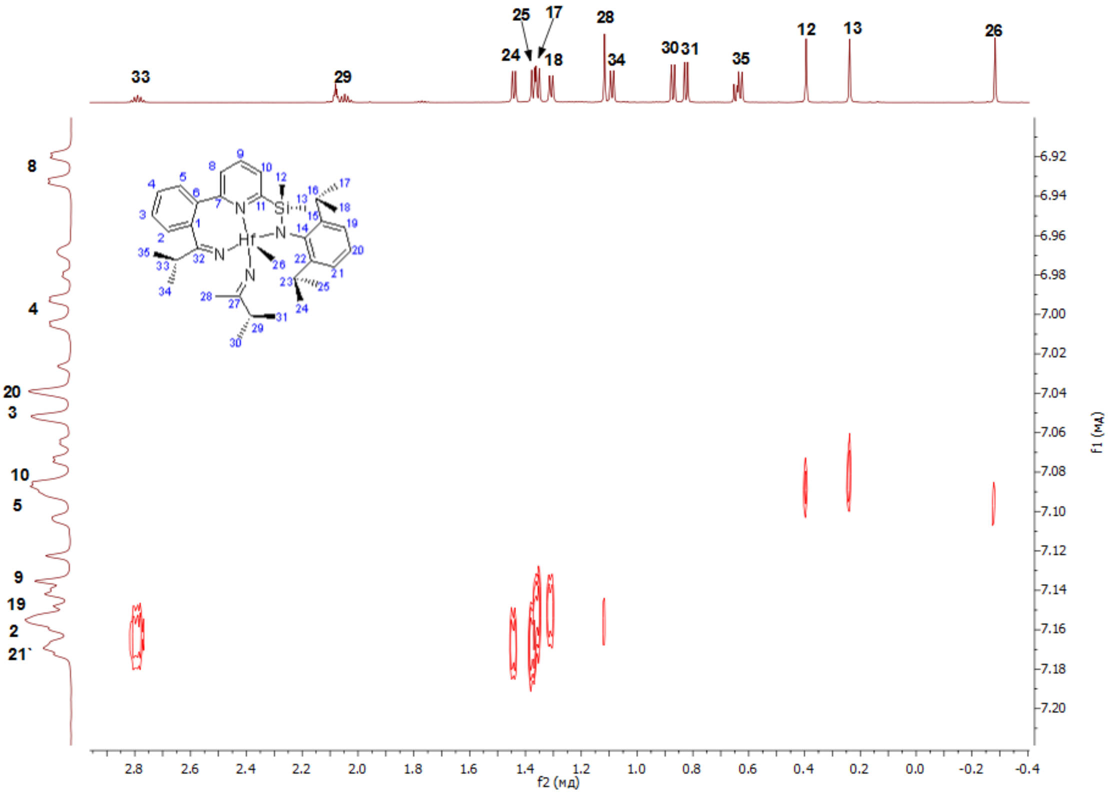

Figure S33. Fragment of ^{1}H-^{1}H ROESY NMR spectrum of **1^{iPrCN}-HfMe(N=CMe*i*Pr)** in toluene-d_8 at room temperature.

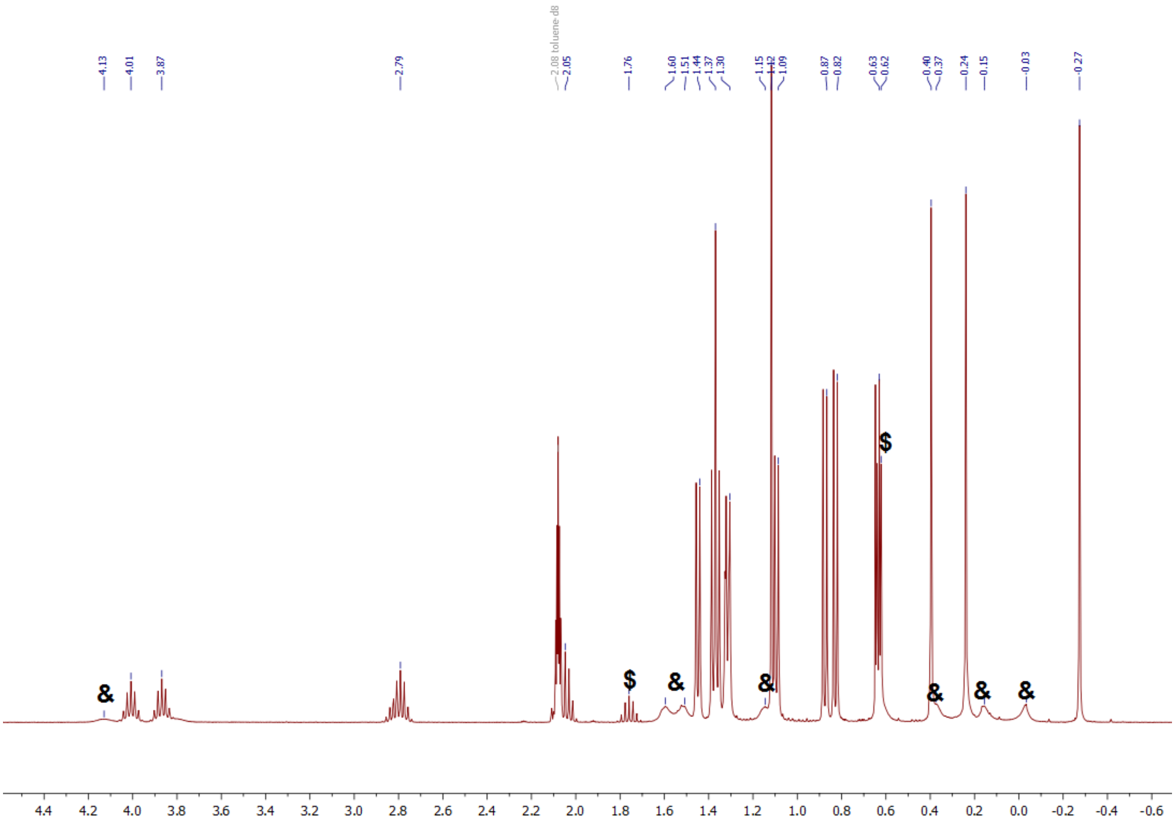

Figure S34. Fragment of ^{1}H NMR spectrum of a mixture of **1^{iPrCN}-HfMe(N=CMe*i*Pr)**, **1^{iPrCN}-HfMe$_2$** and *i*PrCN after 1 h upon mixing of **1-HfMe$_2$** and 2 equiv. of *i*PrCN in toluene-d_8 at room temperature (method 2). A character (&) denotes **1^{iPrCN}-HfMe$_2$**, ($) – *i*PrCN, and the rest of the signals belong to **1^{iPrCN}-HfMe(N=CMe*i*Pr)**.

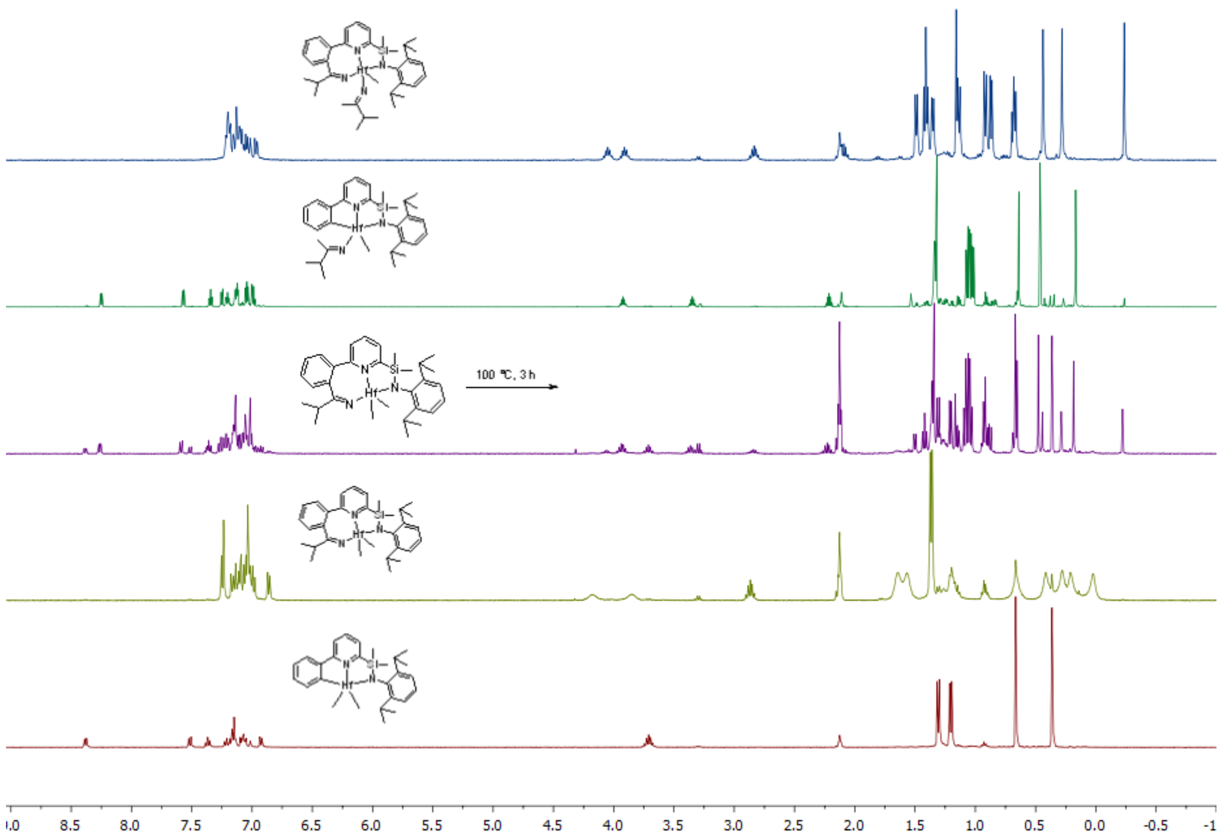

Figure S35. Spectra of complexes (from bottom to top) **1-HfMe$_2$**, **1^{iPrCN}-HfMe$_2$**, **1^{iPrCN}-HfMe$_2$** after 3 h at 100°C, **1-HfMe(N=CMe*i*Pr)** (**1^{iPrCN}-HfMe$_2$** after overnight at 100 °C) and **1^{iPrCN}-HfMe(N=CMe*i*Pr)**.

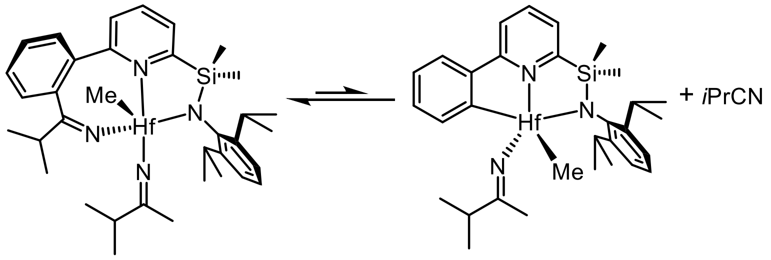

1^{iPrCN}-HfMe(N=CMe*i*Pr) **1**-HfMe(N=CMe*i*Pr)

Scheme S1. Reversible extrusion of *i*PrCN from complex **1^{iPrCN}-HfMe(N=CMe*i*Pr)**.

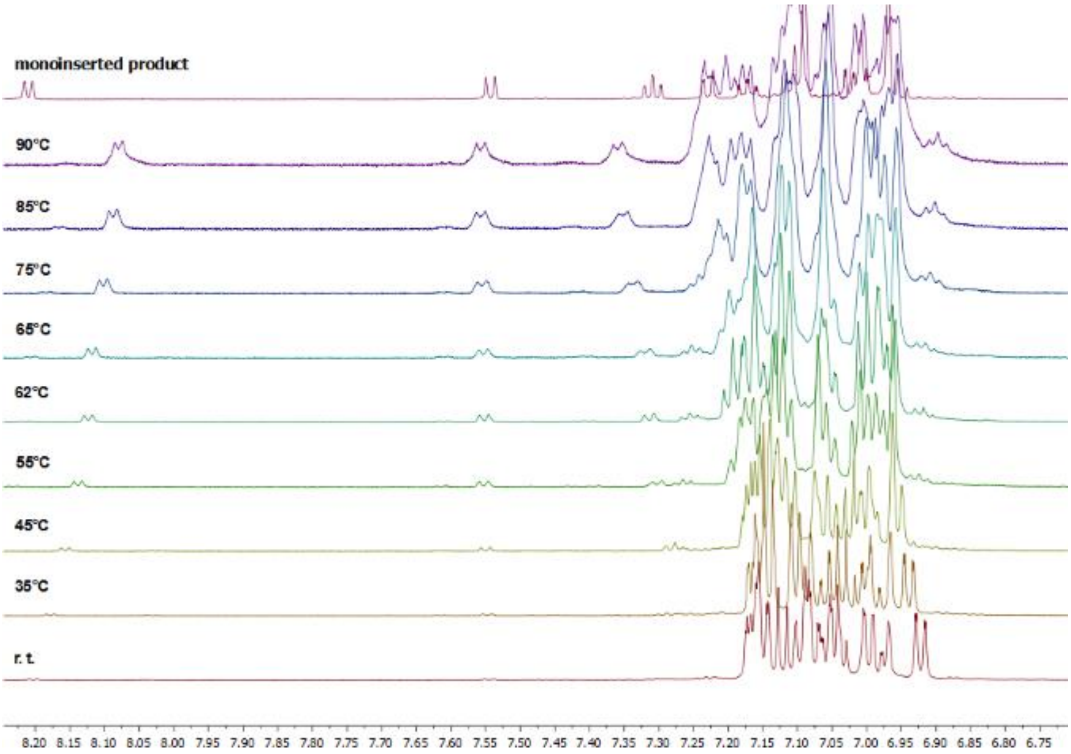

Figure S36. Fragments of ^{1}H NMR spectra of the reaction mixture (shown on Scheme S1) at different temperatures.

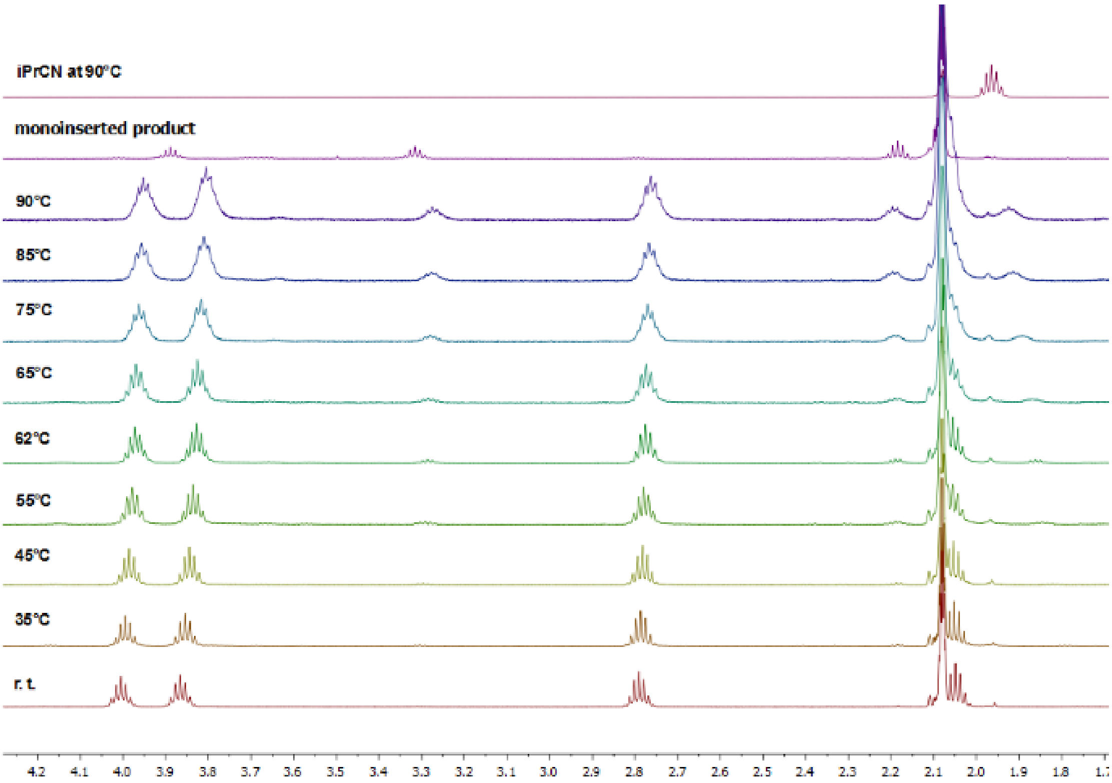

Figure S37. Fragments of ^{1}H NMR spectra of the reaction mixture (shown on Scheme S1) at different temperatures.

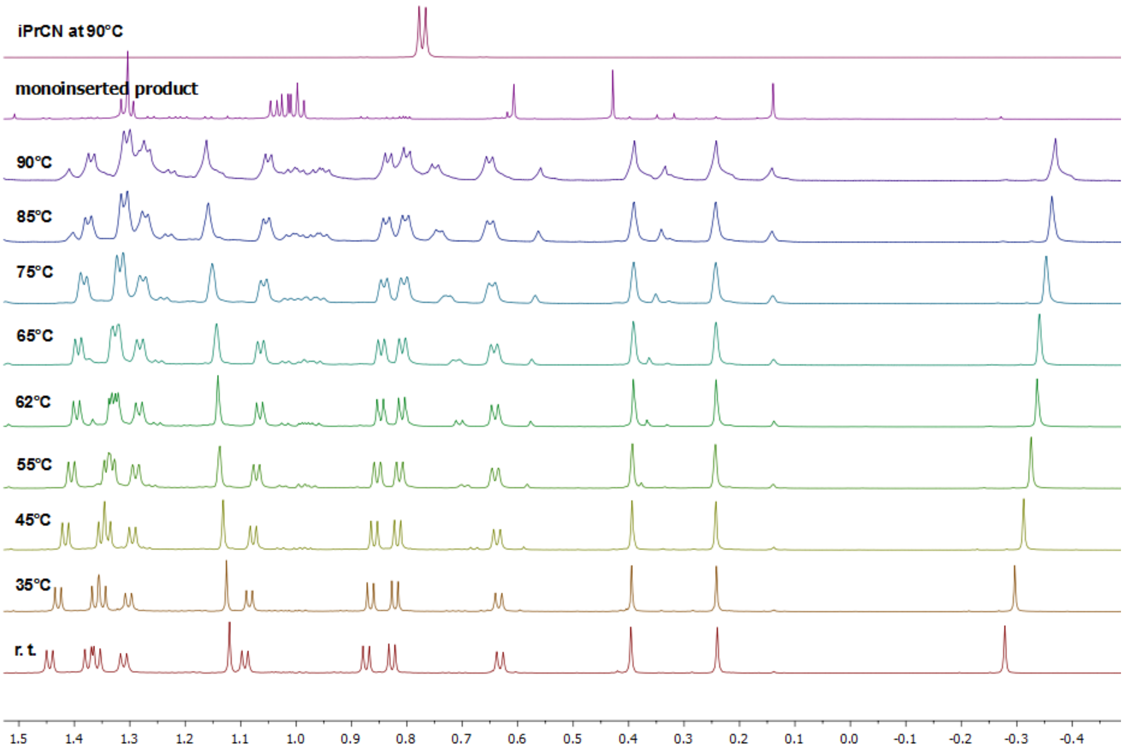

Figure S38. Fragments of ^{1}H NMR spectra of the reaction mixture (shown on Scheme S1) at different temperatures.

2.8. Complex 1^{MeCN}-$HfMe_2$

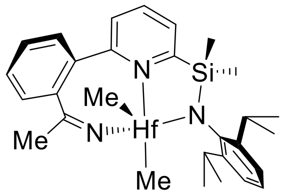

Complex **1-HfMe₂** (21.8 mg, 37 µmol) was dissolved in toluene-*d₈* (0.6 mL) in a J Young NMR tube, then the tube was cooled to -30 °C in a Glovebox freezer, and after that acetonitrile was added (1.9 µL, 1.5 mg, 37 µmol) in one portion via Eppendorf pipette. The resulting yellow solution was immediately analyzed with NMR spectroscopy.

^{1}H NMR (400 MHz, toluene-d_8) δ_H: 7.20 (2H, m), 7.08–7.13 (3H, m), 7.04 (1H, m), 6.93–6.98 (3H, m), 6.81 (1H, dd, $^3J_{HH}$ = 7.9 Hz, $^4J_{HH}$ = 1.4 Hz), 3.95 (2H, br.s), 2.01 (3H, s), 1.56 (6H, d, $^3J_{HH}$ = 6.7 Hz), 1.33 (6H, d, $^3J_{HH}$ =6.9 Hz), 0.29 (6H, br.s), 0.09 (6H, br.s).

^{13}C{^{1}H} NMR (101 MHz, toluene-d_8, selected signals) δ_C: 170.2, 168.6, 160.0, 148.5, 145.8, 138.9, 136.7, 136.2, 132.2, 127.5, 127.3, 125.1, 124.6, 123.9, 28.7, 27.9, 26.6, 25.4.

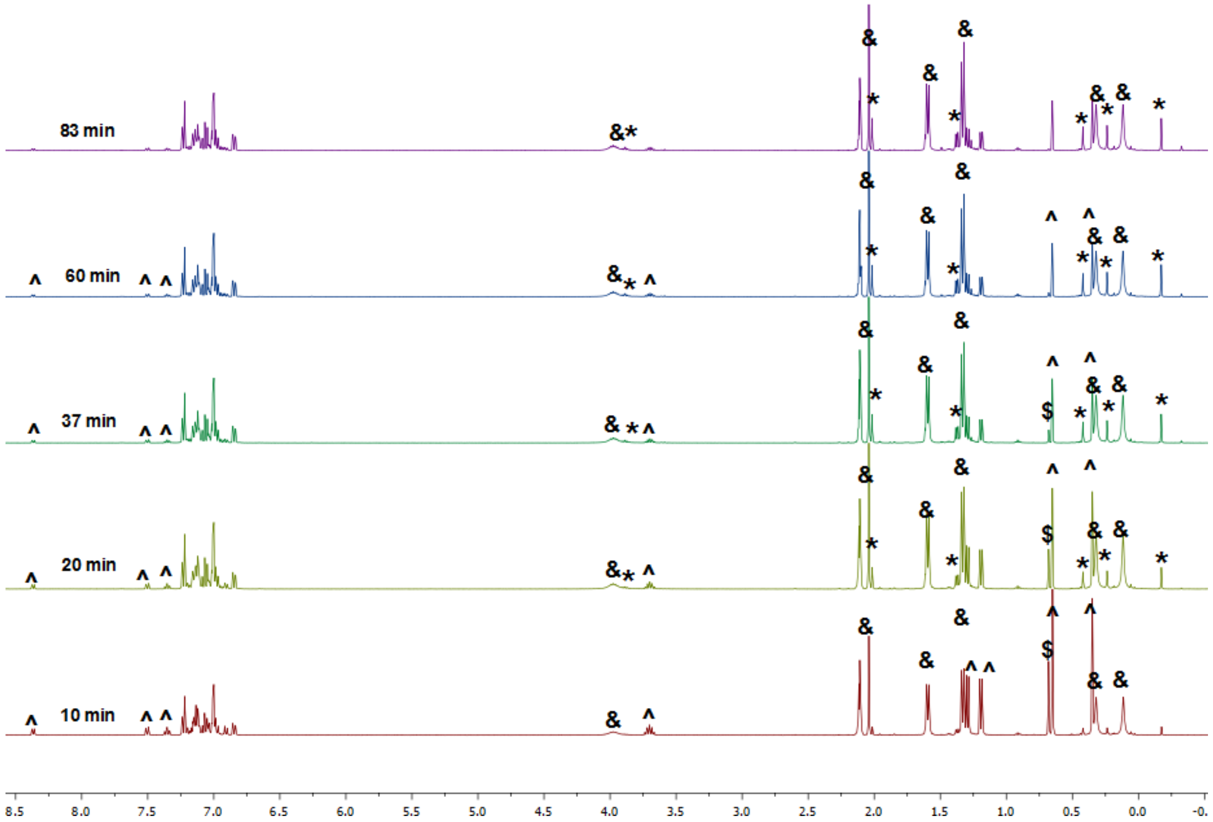

Figure S39. ^{1}H NMR spectra of the reaction of **1-HfMe₂** with 1 equiv. of MeCN in toluene-d_8 at room temperature after (from bottom to top) 10 min, 20 min, 37 min, 60 min and 83 min. Symbol (^) denotes **1-HfMe₂**, (*) –**1^{MeCN}-HfMe(N=CMe₂)**, (&) –**1^{MeCN}-HfMe₂** and ($) – MeCN.

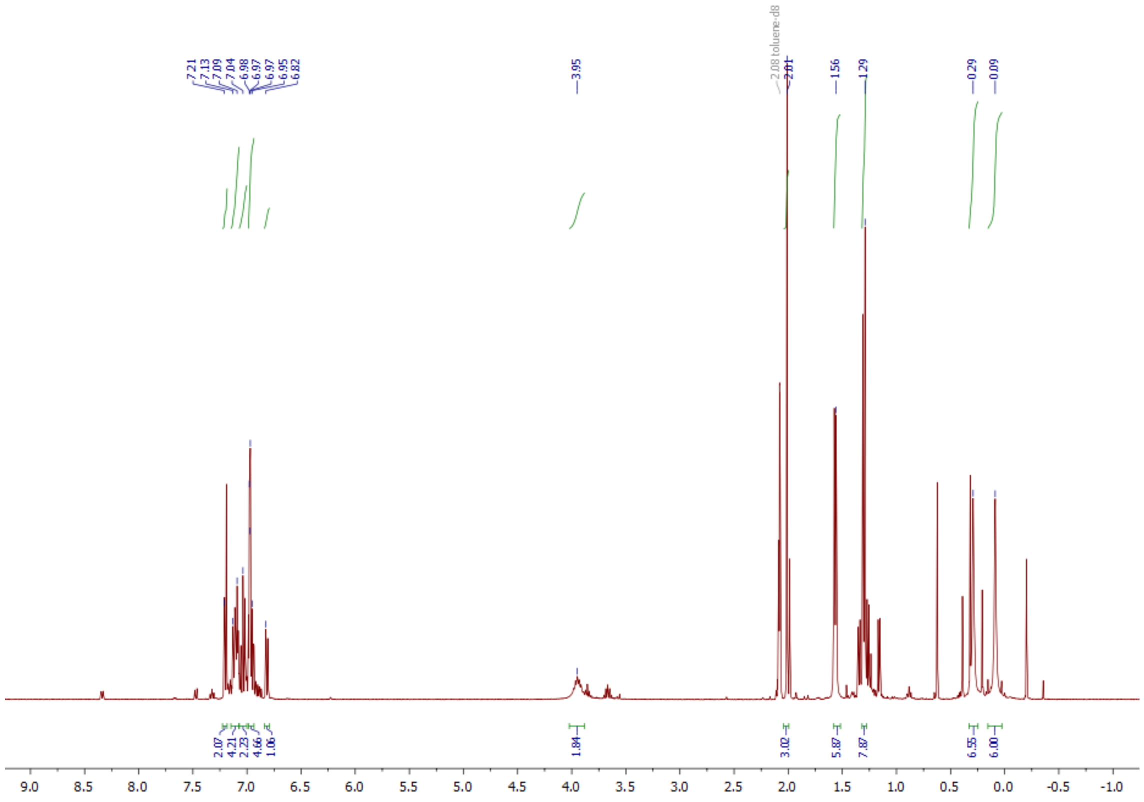

Figure S40. ^{1}H NMR spectrum of the reaction of **1-HfMe₂** with 1 equiv. of MeCN in toluene-d_8 after 83 min at room temperature.

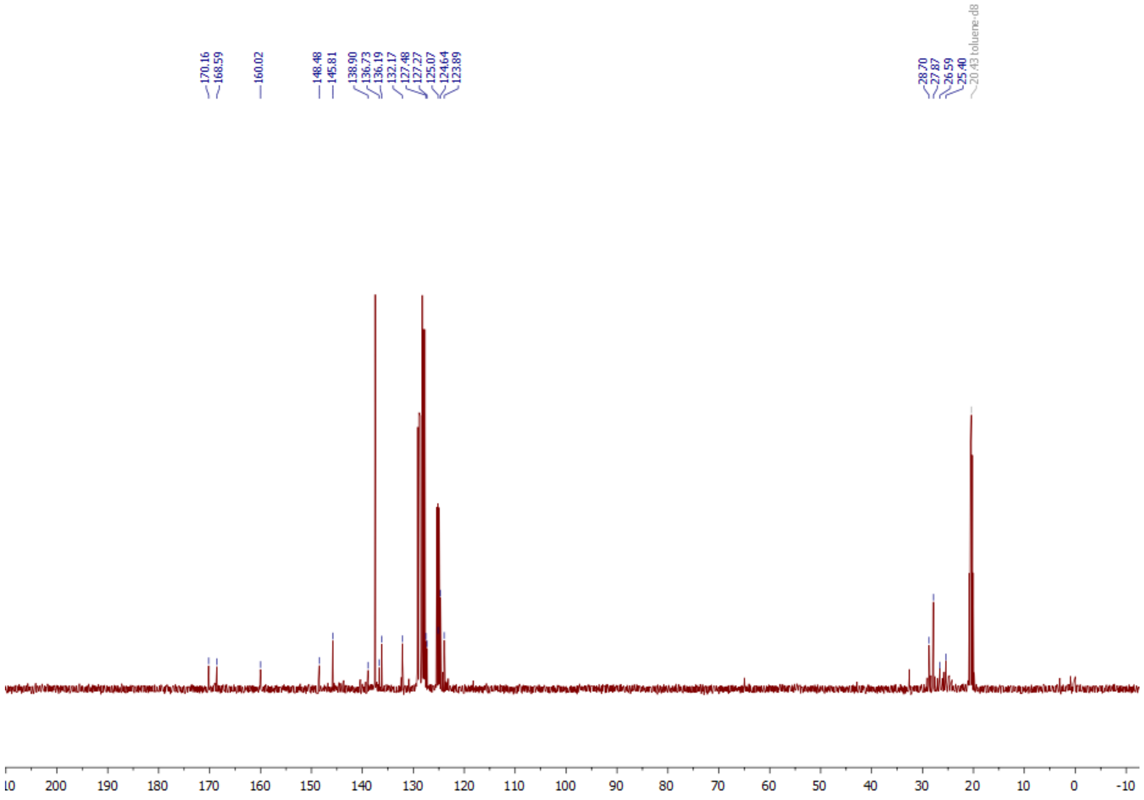

Figure S41. $^{13}C\{^{1}H\}$ NMR spectrum of the reaction of **1-HfMe₂** with 1 equiv. of MeCN in toluene-d_8 after 83 min at room temperature.

2.9. Complex 1-HfMe(N=CMe$_2$)

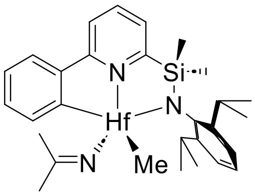

Complex **1-HfMe$_2$** (137 mg, 0.229 mmol) was dissolved in toluene in an 8-mL vial, and then acetonitrile (12.0 µL, 9.4 mg, 0.229 mmol) was added to the solution in one portion via Eppendorf pipette. The resulting orange solution was stirred overnight at 100 °C, then all volatiles were evaporated. The residue was recrystallized from hexane (1.0 mL) at -30 °C giving **1-HfMe(N=CMe$_2$)** as a yellow crystalline solid (78 mg, 54%). Anal. calc. for $C_{29}H_{39}HfN_3Si$: C, 54.75; H, 6.18; N, 6.60. Found: C, 55.02; H, 6.40; N, 6.35.

^{1}H NMR (400 MHz, toluene-d_8) δ_H: 8.34 (1H, m), 7.55 (1H, d, $^3J_{HH}$ = 7.8 Hz), 7.35 (1H, m), 7.23 (1H, dd, $^3J_{HH}$ = 8.2 Hz, J_2 = 1.1 Hz), 7.20 (1H, td, $^3J_{HH}$ = 7.5 Hz, $^4J_{HH}$ = 1.5 Hz), 7.08–7.13 (3H, m), 7.02 (1H, m), 6.93–6.99 (1H, m), 3.98 (1H, sept, $^3J_{HH}$ = 6.8 Hz, ArC*H*Me$_2$), 3.23 (1H, sept, $^3J_{HH}$ = 6.9 Hz, ArC*H*Me$_2$), 1.46 (6H, s, *Me*$_2$C=N), 1.37 (3H, d, $^3J_{HH}$ = 6.8 Hz, ArCH*Me*$_2$), 1.34 (3H, d, $^3J_{HH}$ = 6.9 Hz, ArCH*Me*$_2$), 1.03 (3H, d, $^3J_{HH}$ = 6.9 Hz, ArCH*Me*$_2$), 0.89 (3H, d, $^3J_{HH}$ = 6.8 Hz, ArCH*Me*$_2$), 0.63 (3H, s, *Me*$_2$Si), 0.42 (3H, s, *Me*Hf), 0.13 (3H, s, *Me*$_2$Si).

^{13}C{^{1}H} NMR (101 MHz, toluene-d_8) δ_C: 200.5 (*C*-Hf), 183.0, 171.2, 165.2, 146.8, 146.3, 143.9, 143.6, 141.7, 139.0, 130.2, 126.7, 123.9, 123.3, 123.2, 122.8, 118.3, 52.6, 33.1, 28.4, 27.7, 26.3, 25.6, 25.3, 23.1, 2.9, -1.2.

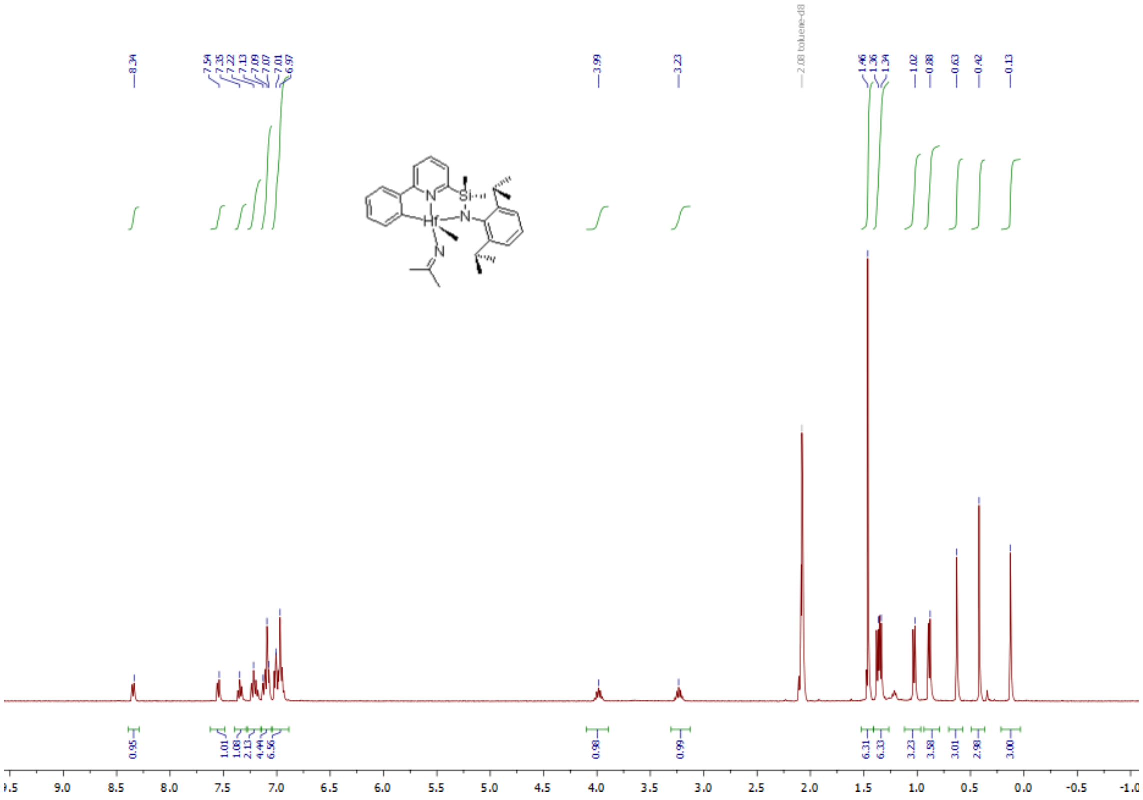

Figure S42. ^{1}H NMR spectra of **1-HfMe(N=CMe$_2$)** in toluene-d_8 at room temperature.

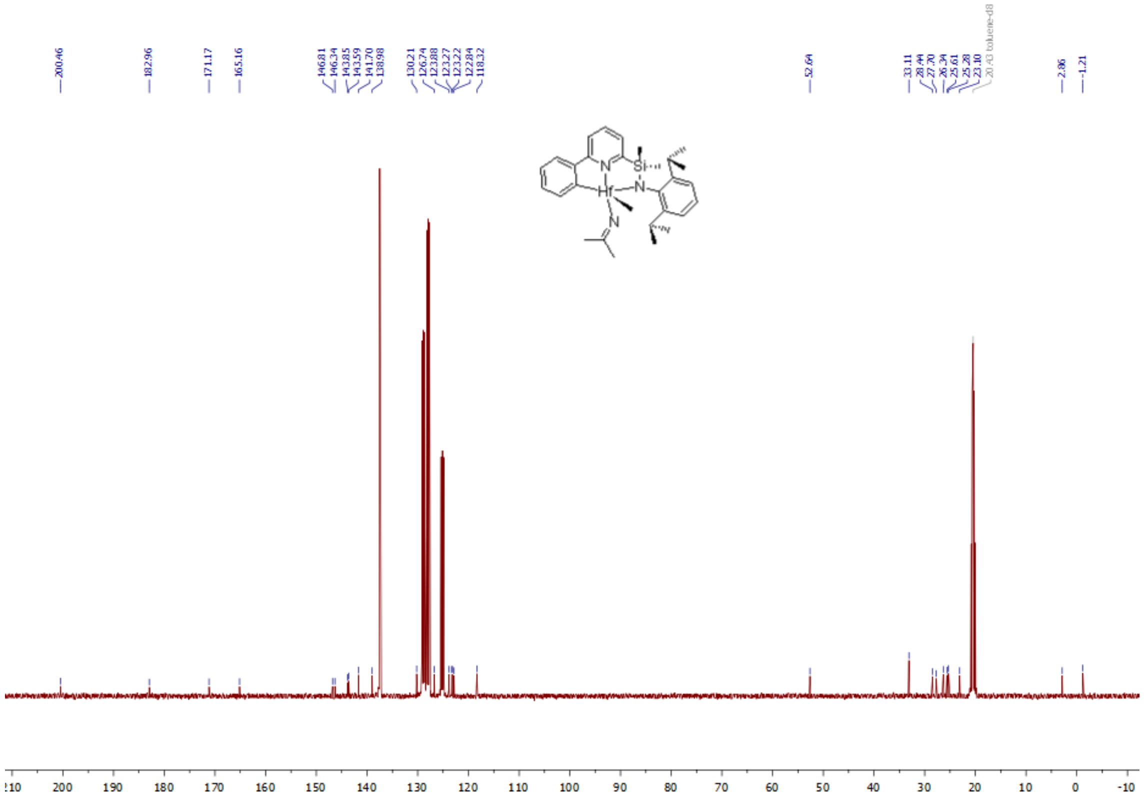

Figure S43. $^{13}C\{^1H\}$ NMR spectrum of **1-HfMe(N=CMe$_2$)** in toluene-d_8 at room temperature.

2.10. Complex 1^{MeCN}-HfMe(N=CMe$_2$)

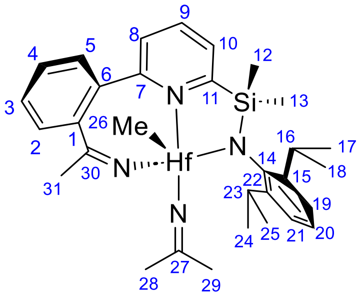

Method 1: Complex **1-HfMe$_2$** (258 mg, 0.433 mmol) was dissolved in 20 mL of a CH_2Cl_2 /hexane (1:1) mixture in 50 mL flask in a Glovebox. Next, acetonitrile (45.8 µL, 36.0 mg, 0.866 mmol) was added in one portion via Eppendorf pipette to the solution at room temperature. The obtained orange solution was stirred for 3 h at room temperature, and then the solvents were partially evaporated until precipitation occurred. The precipitate was filtered off while the mixture was still cold (the temperature decrease was caused by solvent evaporation), washed with cold hexane (kept in the freezer at -30 °C), and dried in vacuum giving **1^{MeCN}-HfMe(N=CMe$_2$)** as a bright-yellow solid (252 mg, 86%). Anal. calc. for $C_{31}H_{42}HfN_4Si$: C, 54.98; H, 6.25; N, 8.27. Found: C, 55.32; H, 6.40; N, 8.06.

Method 2: Complex **1-HfMe$_2$** (47.2 mg, 79 µmol) was dissolved in C_6D_6 (0.6 mL) or toluene-d_8 (0.6 mL) in J Young NMR tube. Next, acetonitrile (8.3 µL, 6.5 mg, 0.158 mmol) was added to the solution in one portion via Eppendorf pipette. After 10 min, the obtained mixture was analyzed by NMR spectroscopy (Figure S55).

^{1}H NMR (600 MHz, toluene-d_8) δ_H: 7.22 (1H, m, C(21)H), 7.18 (1H, m, C(19)H), 7.11–7.16 (2H, m, C(5)H and C(2)H), 7.06–7.09 (3H, m, C(9)H and C(10)H and C(20)H), 6.97–7.01 (2H, m, C(4)H and C(3)H), 6.93 (1H, m, C(8)H), 4.01 (1H, m, C(16)H), 3.92 (1H, m, C(23)H), 2.03 (3H, s, C(31)H), 1.65 (3H, d, $^3J_{HH}$ = 6.7 Hz, C(24)H), 1.35–1.38 (9H, m, C(17)H and C(18)H and C(25)H), 1.31 (6H, s, C(28)H and C(29)H), 0.41 (3H, s, C(12)H), 0.20 (3H, s, C(13)H), -0.06 (3H, s, C(26)H).

^{13}C{^{1}H} NMR (150 MHz, toluene-d_8) δ_C: 179.4 (C-27), 168.9 (C-11), 168.3 (C-30), 159.9 (C-7), 148.6 (C-1), 145.5 (C-14), 144.3 (C-22), 143.7 (C-15), 136.7 (C-6), 136.3 (C-9), 132.4 (C-5), 128.2 (C-3 and C-8), 127.7 (C-10), 127.2 (C-4), 123.9 (C-2 and C-19), 123.6 (C-21), 123.2 (C-20), 42.9 (C-26), 32.6 (C-28 and C-29), 29.1 (C-31), 28.0 (C-23), 27.5 (C-16), 27.0 (C-24), 26.0 (C-17), 25.0 (C-18), 24.3 (C-25), 3.0 (C-12), 0.0 (C-13).

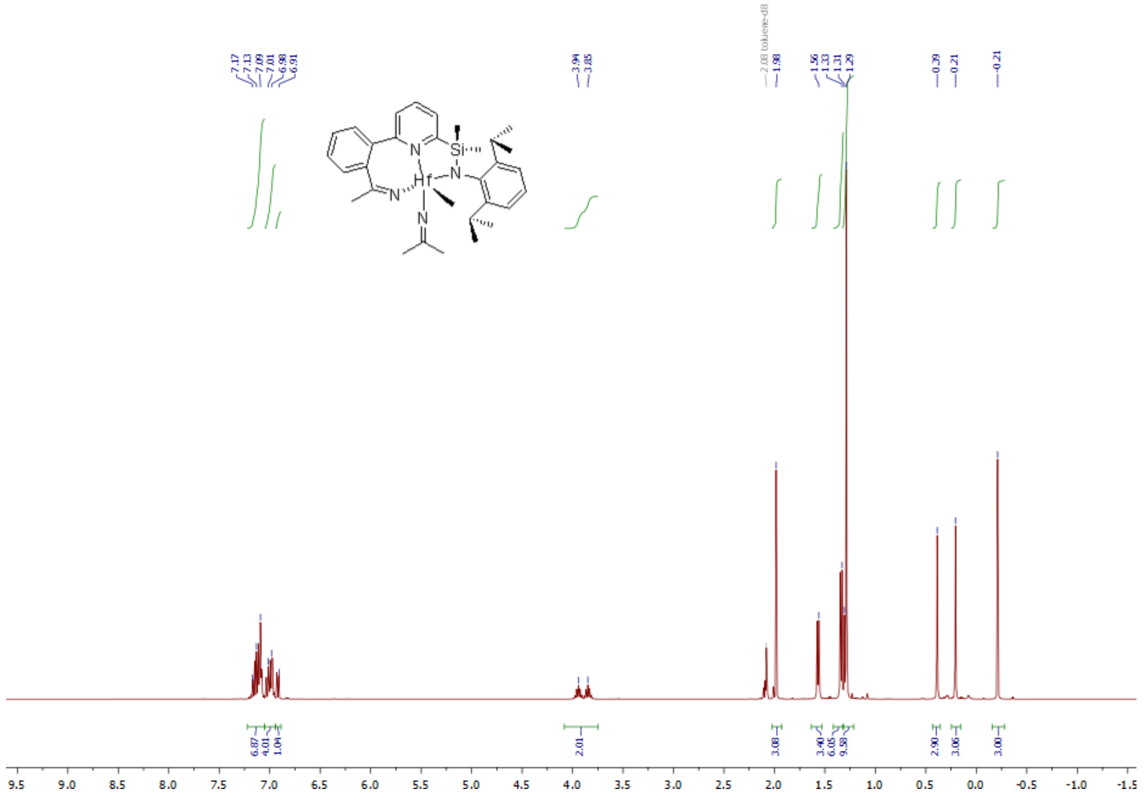

Figure S44. ^{1}H NMR spectrum of complex **1^{MeCN}-HfMe(N=CMe$_2$)** (obtained via method 1) in toluene-d_8 at room temperature.

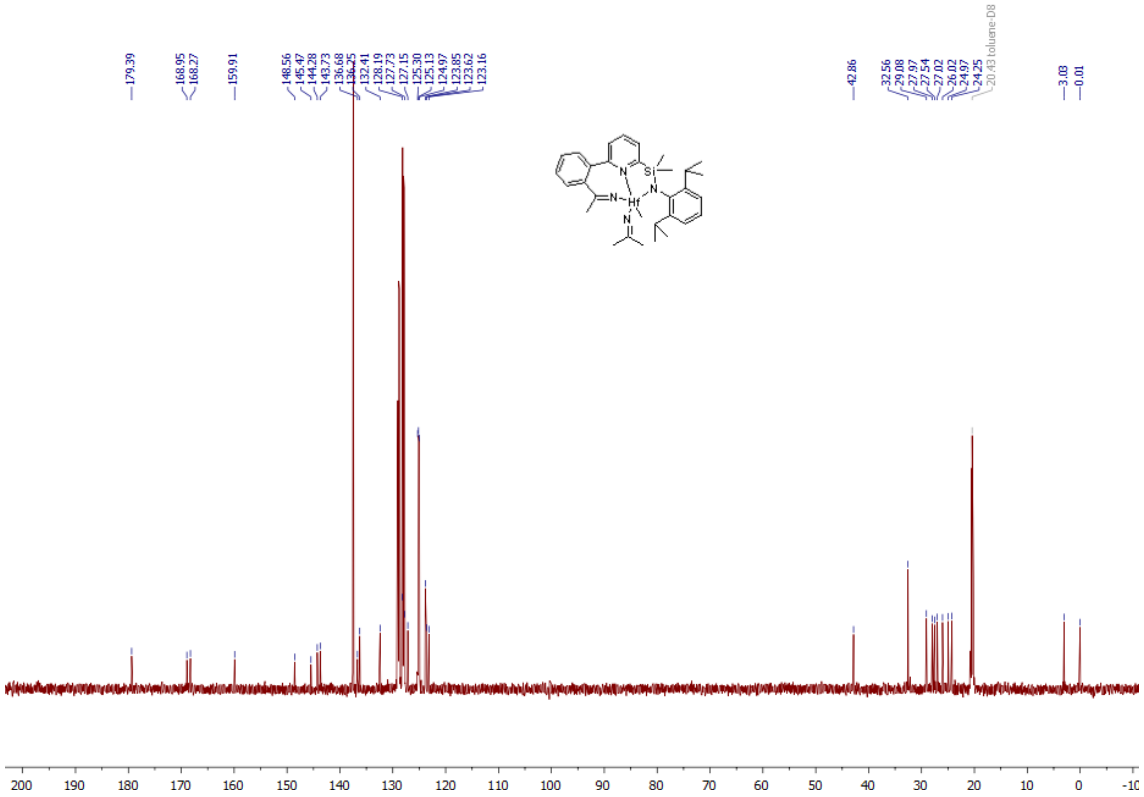

Figure S45. ^{13}C{^{1}H} NMR spectrum of complex **1^{MeCN}-HfMe(N=CMe$_2$)** (obtained via method 1) in toluene-d_8 at room temperature.

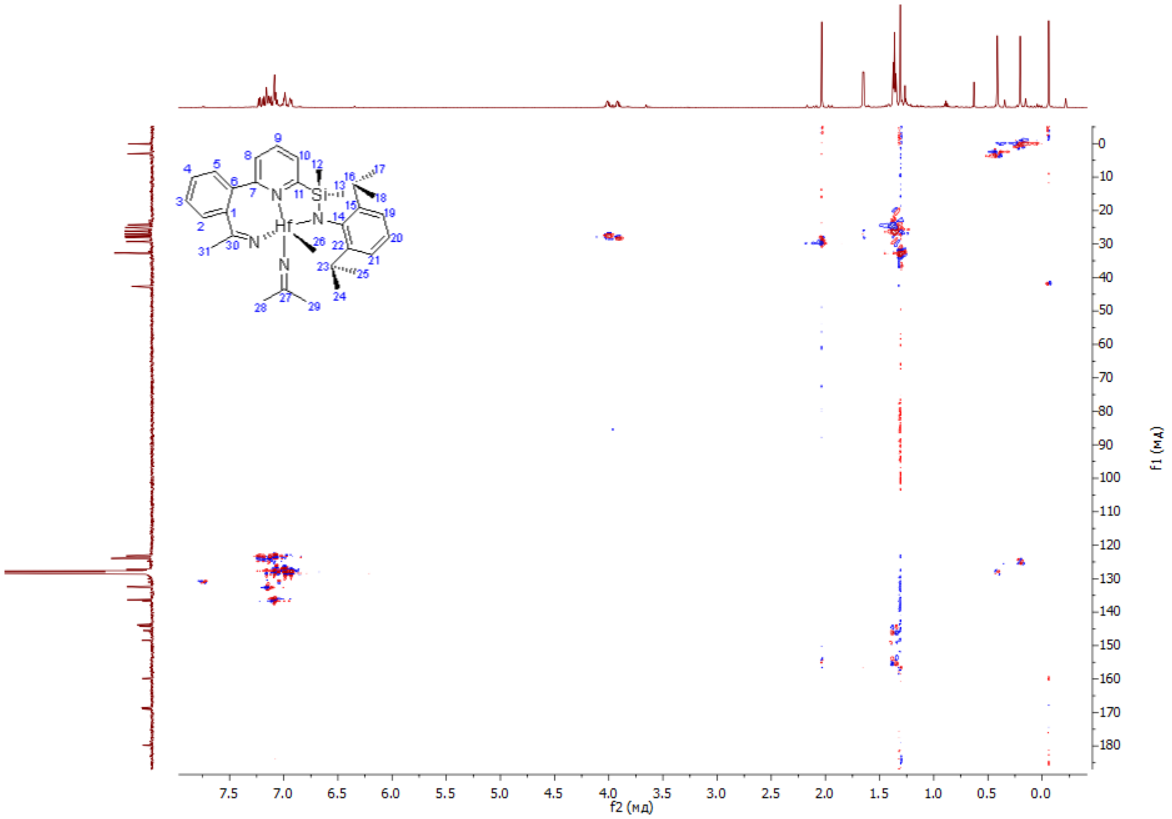

Figure S46. ^{13}C-^{1}H HSQC spectrum of **1^{MeCN}-HfMe(N=CMe$_2$)** in C_6D_6 at room temperature.

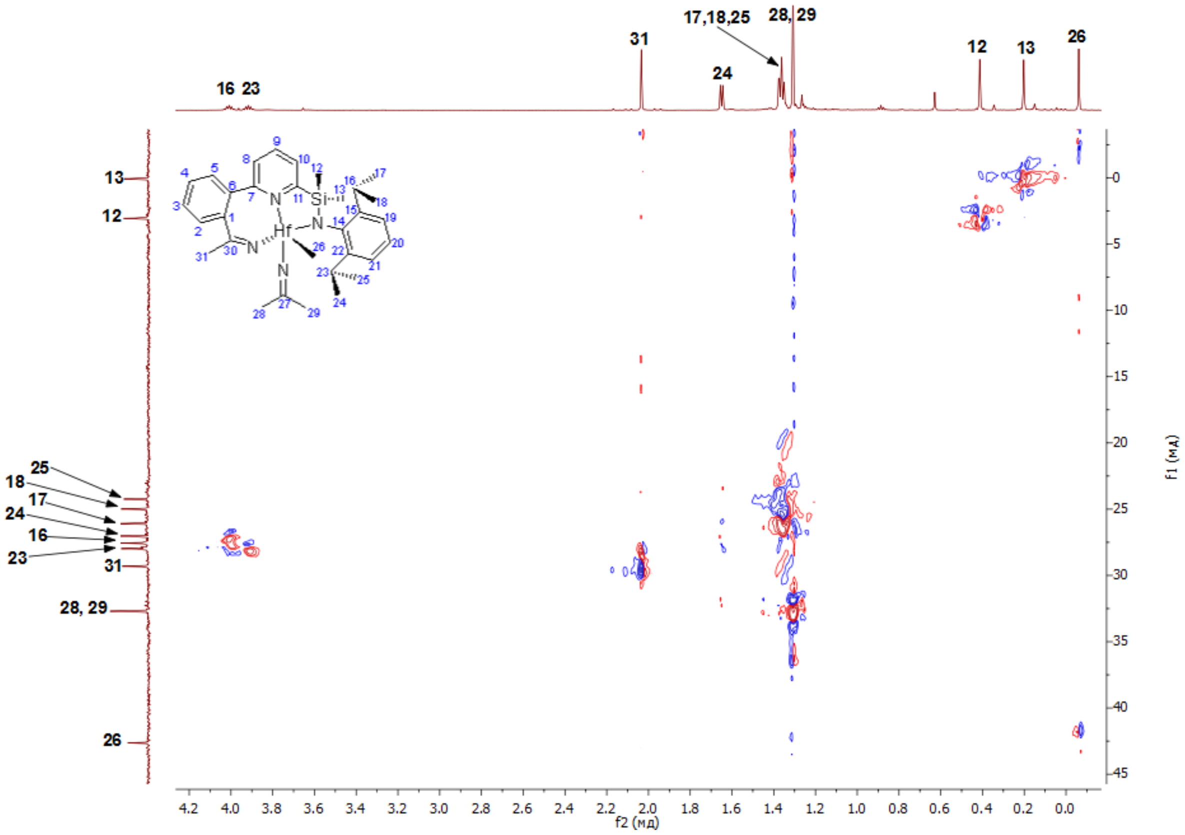

Figure S47. Fragment of ^{13}C-^{1}H HSQC spectrum of **1^{MeCN}-HfMe(N=CMe$_2$)** in C_6D_6 at room temperature.

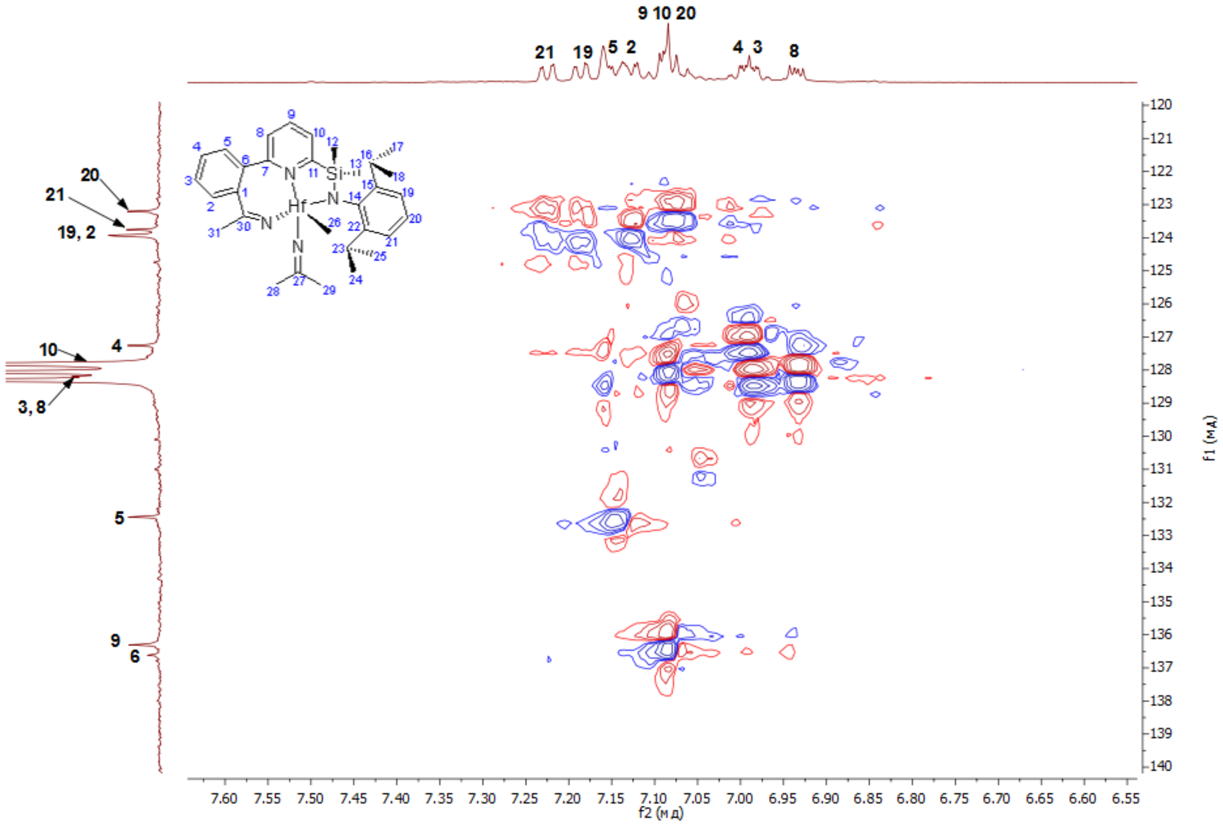

Figure S48. Fragment of ^{13}C-^{1}H HSQC spectrum of **1^{MeCN}-HfMe(N=CMe$_2$)** in C_6D_6 at room temperature.

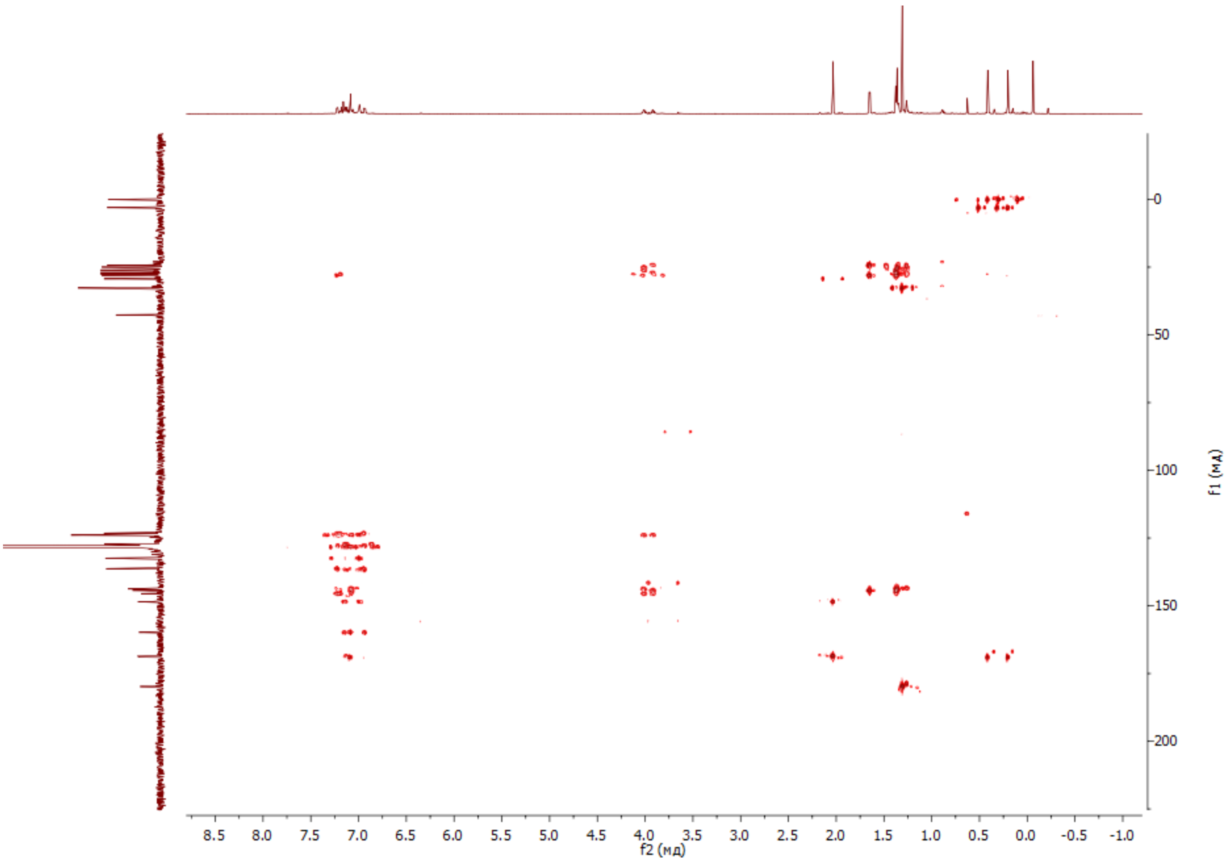

Figure S49. ^{13}C-^{1}H HMBC NMR spectrum of **1^{MeCN}-HfMe(N=CMe$_2$)** in C_6D_6 at room temperature.

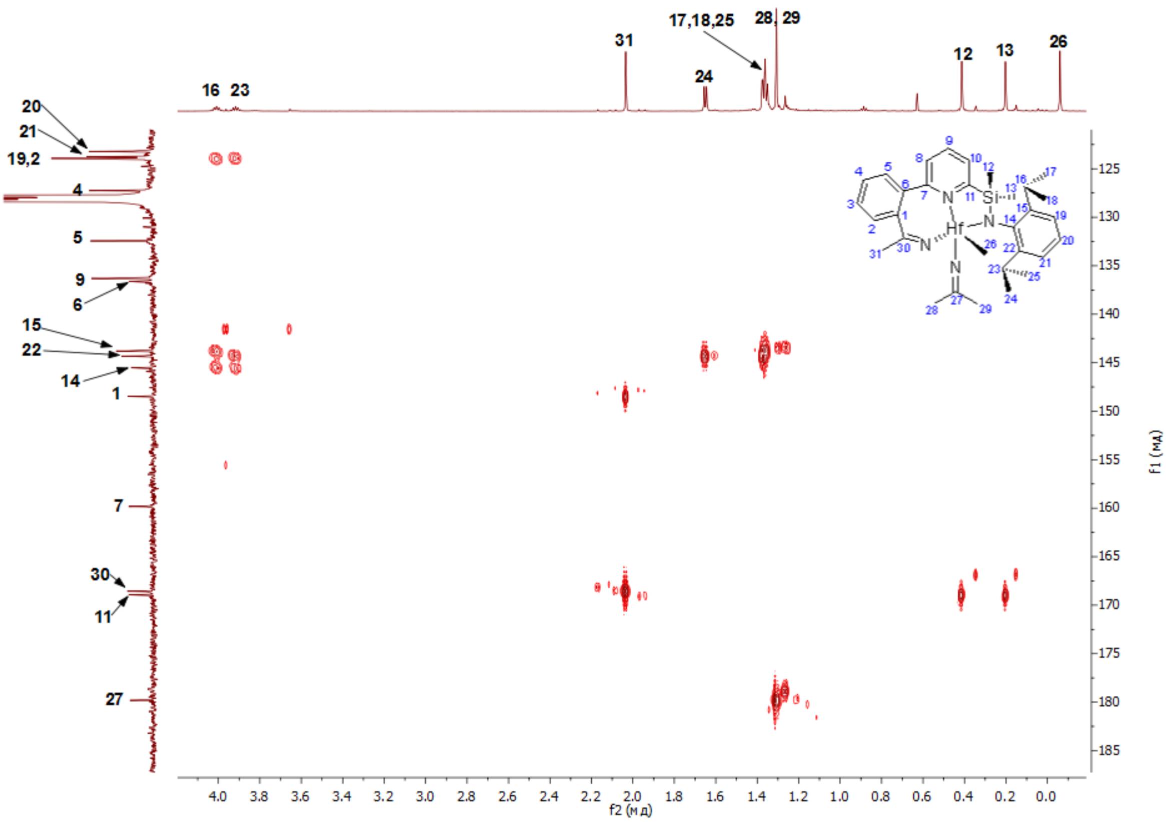

Figure S50. Fragment of ^{13}C-^{1}H HMBC NMR spectrum of **1^{MeCN}-HfMe(N=CMe$_2$)** in C_6D_6 at room temperature.

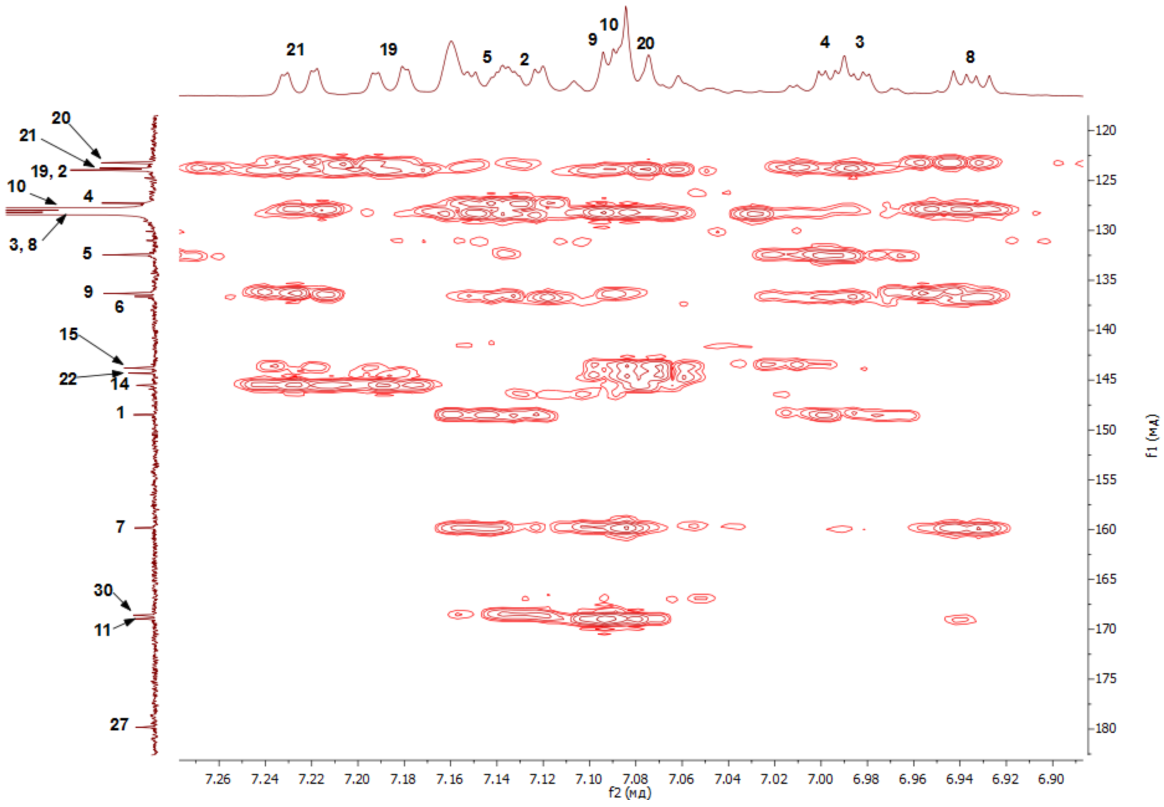

Figure S51. Fragment of ^{13}C-^{1}H HMBC NMR spectrum of **1^{MeCN}-HfMe(N=CMe$_2$)** in C_6D_6 at room temperature.

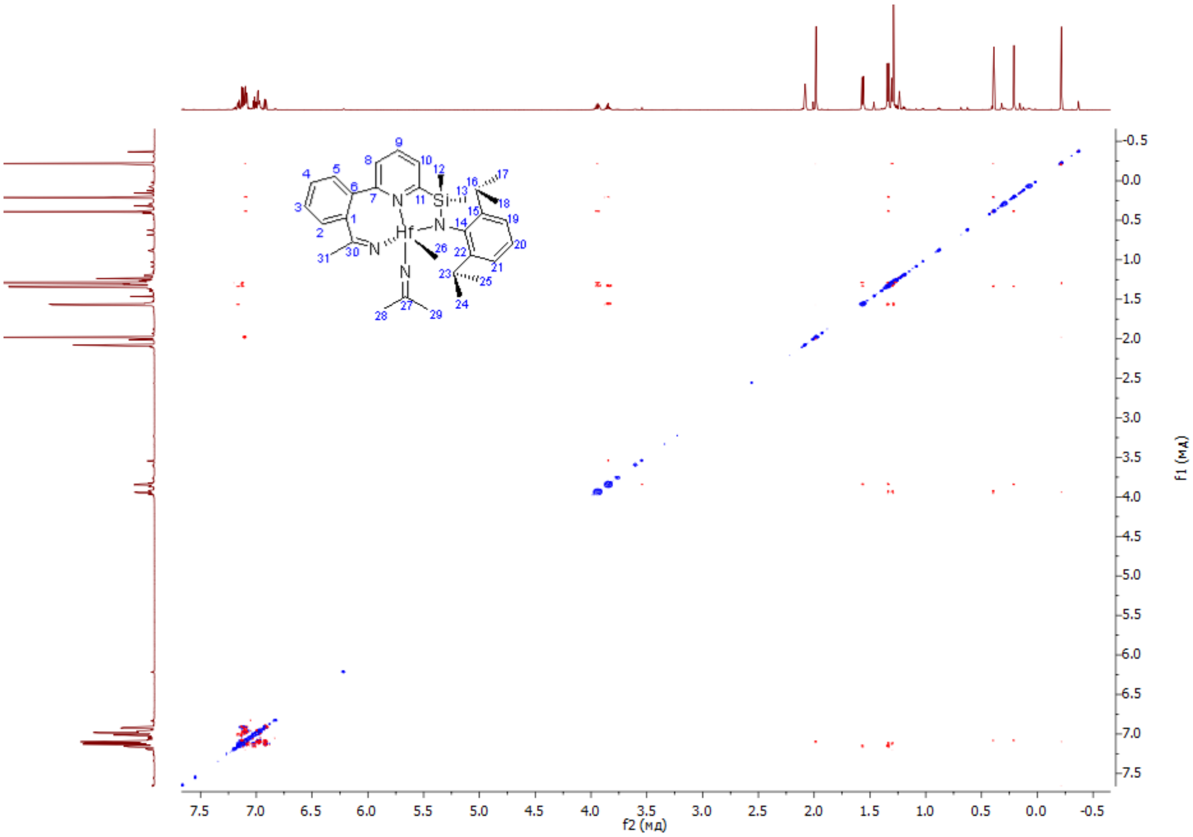

Figure S52. ^{1}H-^{1}H NOESY spectrum of **1^{MeCN}-HfMe(N=CMe$_2$)** in toluene-*d$_8$* at room temperature.

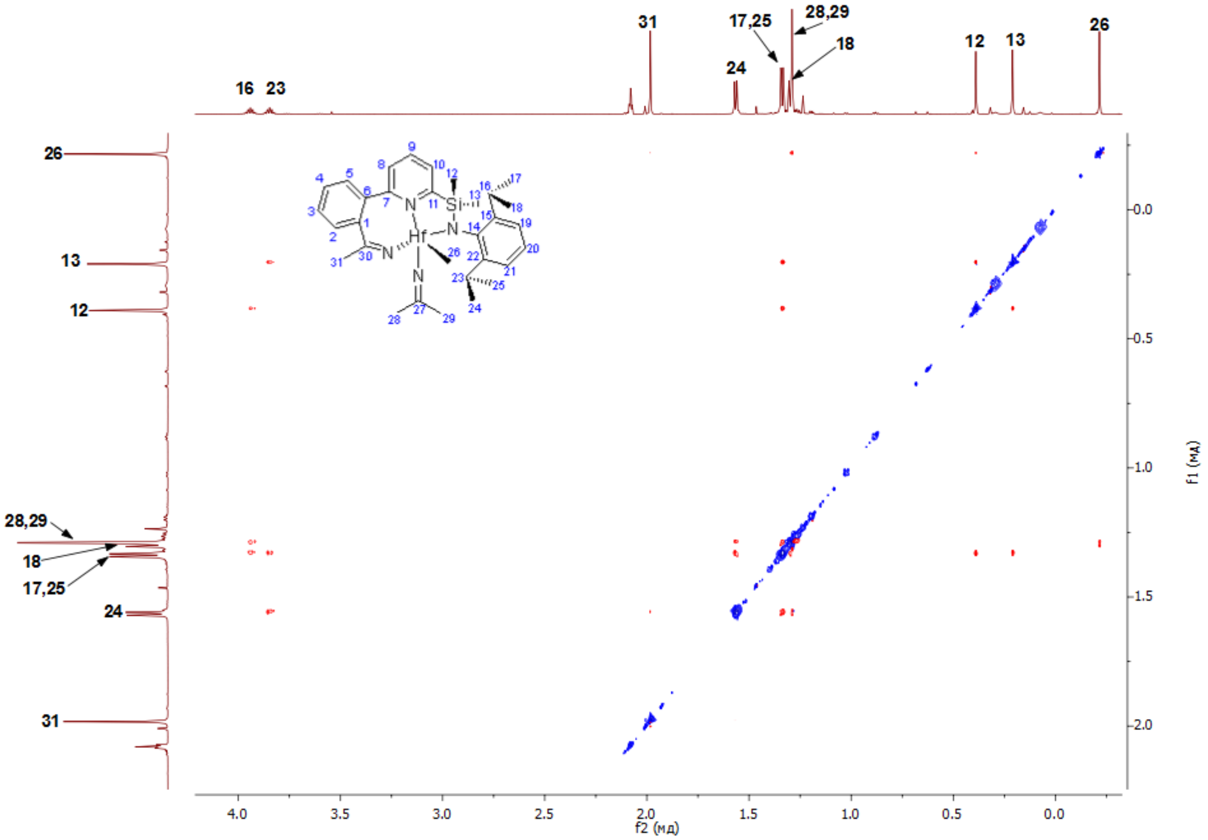

Figure S53. Fragment of ^{1}H-^{1}H NOESY spectrum of **1^{MeCN}-HfMe(N=CMe$_2$)** in toluene-*d$_8$* at room temperature.

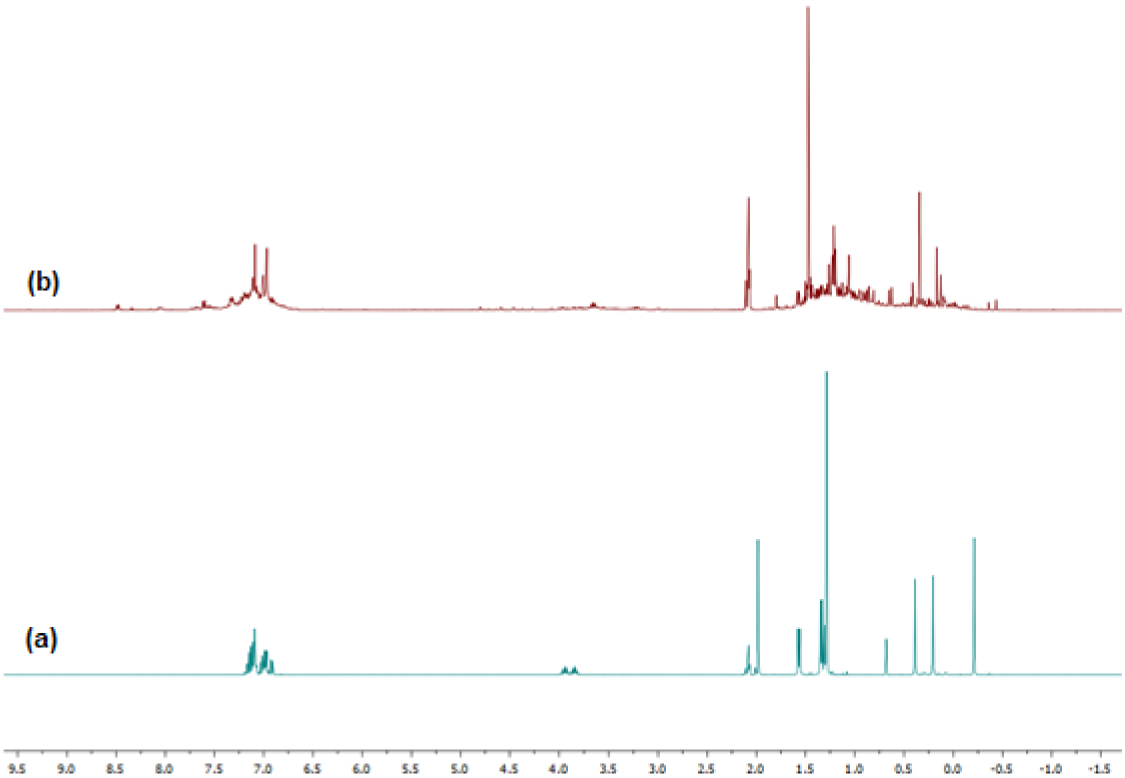

Figure S54. ^{1}H NMR of complex **1^{MeCN}-HfMe(N=CMe$_2$)** before **(a)** and after **(b)** heating at 100 °C in toluene-d_8 overnight.

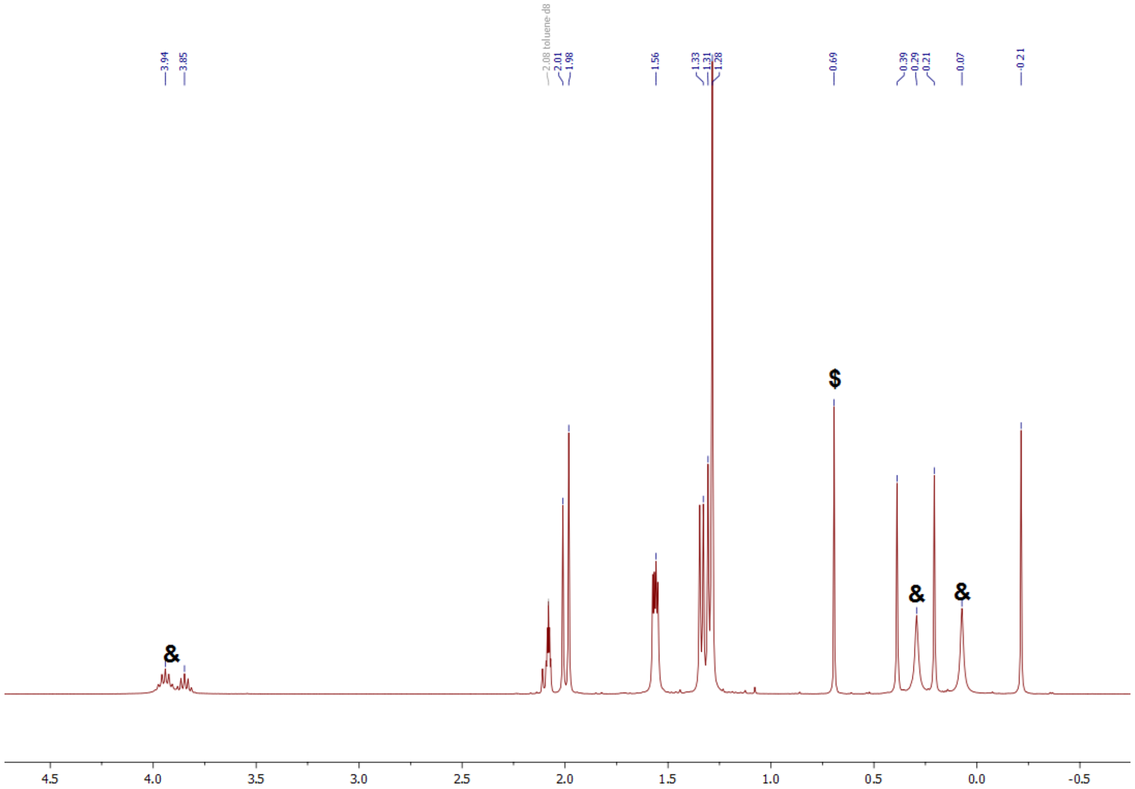

Figure S55. Fragment of ^{1}H NMR spectrum of a mixture of **1^{MeCN}-HfMe(N=CMe$_2$)**, **1^{MeCN}-HfMe$_2$**, and MeCN obtained after 10 min upon mixing **1-HfMe$_2$** and 2 equv. of MeCN in toluene-d_8 at room temperature (method 2). A character (**&**) denotes **1^{MeCN}-HfMe$_2$**, (**$**) – MeCN, and the rest of the signals belong to **1^{MeCN}-HfMe(N=CMe$_2$)**.

2.11. Complex 1^{tBuCN}-$HfCl_2$

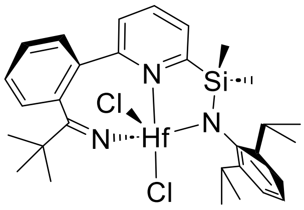

Complex **1-$HfCl_2$** (250 mg, 0.393 mmol) was dissolved in CH_2Cl_2 (15 mL), and then trimethylacetonitrile (47.9 µL, 36.0 mg, 0.433 mmol) was added to the solution in one portion via Eppendorf pipette at room temperature. The resulting yellow solution was stirred for 4 h at room temperature, then all volatiles were evaporated. The residue was washed with hexane (2 × 10 mL) and dried in vacuum to give **1^{tBuCN}-$HfCl_2$** as a bright yellow solid (220 mg, 78%). Anal. calc. for $C_{30}H_{39}Cl_2HfN_3Si$: C, 50.11; H, 5.47; N, 5.84. Found: C, 50.40; H, 5.62; N, 5.57.

^{1}H NMR (400 MHz, CD_2Cl_2) δ_H: 8.01 (1H, t, $^3J_{HH}$ = 7.8 Hz), 7.68 (1H, d, $^3J_{HH}$ = 7.5 Hz), 7.61 (1H, d, $^3J_{HH}$ = 7.7 Hz), 7.50–7.54 (1H, m), 7.44 (3H, m), 7.11–7.20 (3H, m), 3.81 (1H, sept, $^3J_{HH}$ = 6.7 Hz, ArC*H*Me$_2$), 3.64 (1H, sept, $^3J_{HH}$ = 6.8 Hz, ArC*H*Me$_2$), 1.41 (3H, d, $^3J_{HH}$ = 6.7 Hz, ArCH*Me*$_2$), 1.24–1.30 (9H, m, ArCH*Me*$_2$), 1.00 (9H, s, *tBu*C(Ar)=N), 0. 40 (3H, s, *Me*$_2$Si), 0.35 (3H, s, *Me*$_2$Si).

^{13}C{^{1}H} NMR (101 MHz, CD_2Cl_2) δ_C: 183.8, 167.7, 159.1, 146.7, 145.4, 145.2, 141.8, 138.6, 135.7, 132.0, 129.1, 128.7, 128.6, 127.9, 125.3, 124.6, 124.4, 124.1, 42.8 ((CH$_3$)$_3$<u>*C*</u>(Ar)=N), 28.2 ((<u>*C*</u>H$_3$)$_3$C(Ar)=N), 27.9, 27.8, 26.8, 26.4, 24.9, 24.7, 1.3 (*C*-Si), 0.8 (*C*-Si).

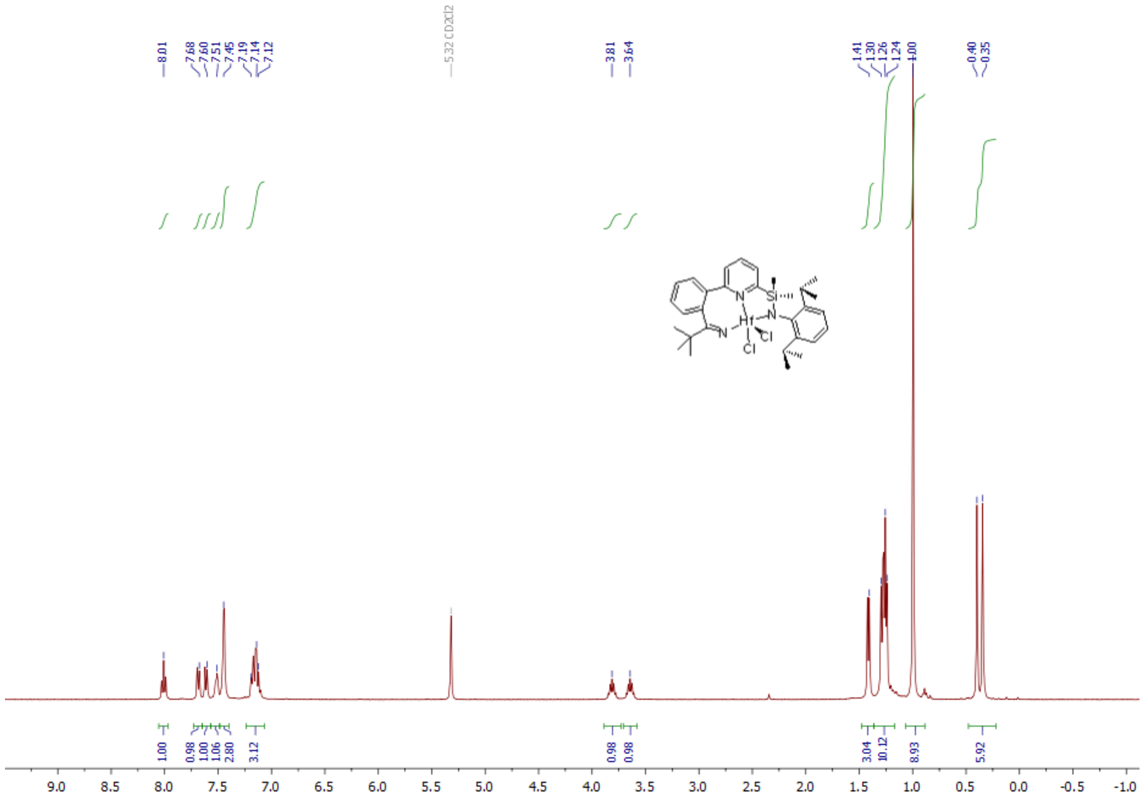

Figure S56. ^{1}H NMR spectrum of **1^{tBuCN}-$HfCl_2$** in CD_2Cl_2 at room temperature.

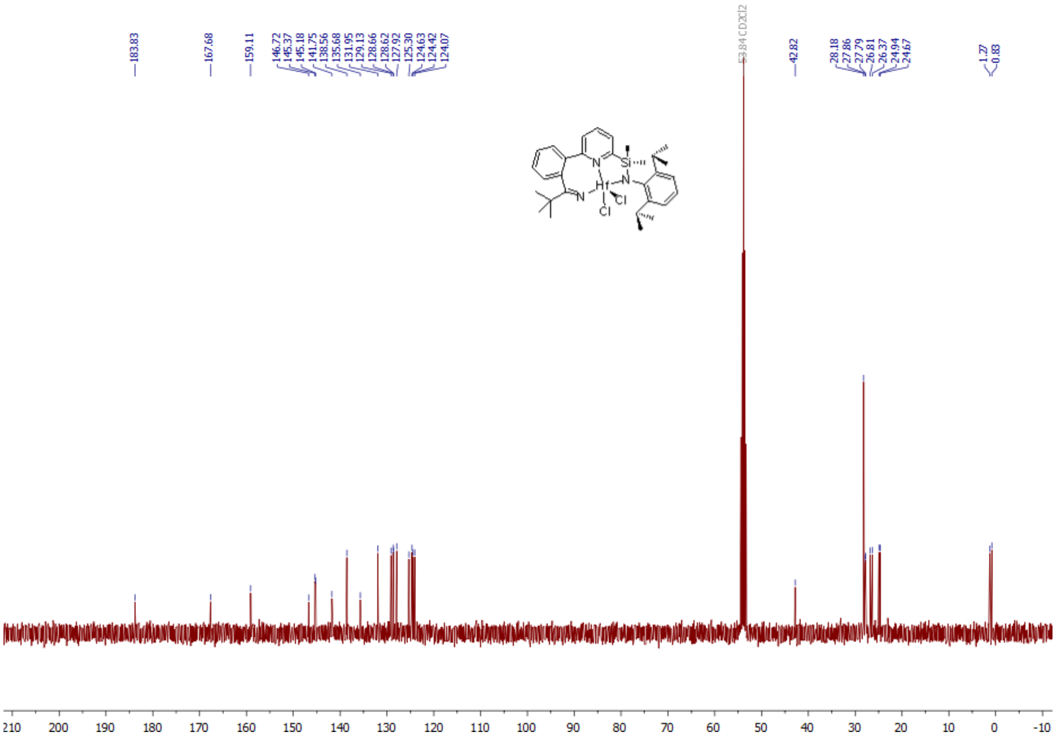

Figure S57. $^{13}C\{^1H\}$ NMR spectrum of $\mathbf{1^{\mathit{t}BuCN}}$**-HfCl$_2$** in CD_2Cl_2 at room temperature.

2.12. Complex 1^{tBuCN}-$HfMe_2$

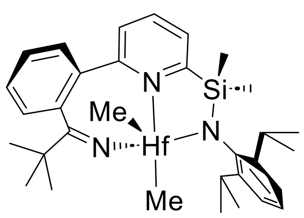

Method 1: Complex **1^{tBuCN}-$HfCl_2$** (150 mg, 0.209 mmol) was suspended in diethyl ether (10 mL), and then a solution of MeMgBr in diethyl ether (0.12 mL, 0.44 mmol, 3.7 M) was added via glass syringe at -30 °C. The resulting mixture was slowly warmed to room temperature and stirred for 2 h giving a yellow solution with white precipitate. Next, all volatiles were evaporated. The residue was extracted with room temperature hexane (50 mL) filtering the obtained slurry through a short pad of Celite®. The solvent was evaporated until yellow crystals precipitated. The precipitate was filtered off while the solution was still cold, washed with cold pentane (10 mL, kept in freezer at -30 °C), and dried in vacuum giving **1^{tBuCN}-$HfMe_2$** as a pale yellow solid (81 mg, 57%). Anal. Calc. for $C_{32}H_{45}HfN_3Si$: C, 56.66; H, 6.69; N, 6.20. Found: C, 56.93; H, 6.90; N, 5.95.

Method 2: Complex **1-$HfMe_2$** (30 mg, 50 µmol) was dissolved in toluene-d_8 (0.6 mL) in J Young NMR tube. Next, trimethylacetonitrile (5.6 µL, 4.2 mg, 50 µmol) was added to the solution in one portion via Eppendorf pipette. The obtained yellowish solution was kept for 7 days at room temperature and then analysed by NMR spectroscopy (Figure S59).

^{1}H NMR (400 MHz, toluene-d_8): δ_H 7.17–7.21 (2H, m), 7.11 (2H, m), 7.05 (2H, m), 6.99 (2H, m), 6.94 (1H, dd, $^3J_{HH}$ = 7.5 Hz, $^4J_{HH}$ = 1.4 Hz), 6.88 (1H, dd, $^3J_{HH}$ = 7.9 Hz, $^4J_{HH}$ = 1.4 Hz), 4.09 (1H, sept, $^3J_{HH}$ = 6.8 Hz, ArC*H*Me$_2$), 3.87 (1H, sept, $^3J_{HH}$ = 6.7 Hz, ArC*H*Me$_2$), 1.59 (3H, d, $^3J_{HH}$ = 6.7 Hz, ArCH*Me*$_2$), 1.48 (3H, d, $^3J_{HH}$ = 6.8 Hz, ArCH*Me*$_2$), 1.33 (3H, d, $^3J_{HH}$ = 6.8 Hz, ArCH*Me*$_2$), 1.31 (3H, d, $^3J_{HH}$ = 6.7 Hz, ArCH*Me*$_2$), 0.97 (9H, s, *tBu*C(Ar)=N), 0.34 (3H, s, *Me*$_2$Hf), 0.26 (3H, s, *Me*$_2$Hf), 0.17 (3H, s, *Me*$_2$Si), -0.09 (3H, s, *Me*$_2$Si).

^{13}C{^{1}H} NMR (101 MHz, toluene-d_8): δ_C 179.2, 168.7, 160.4, 148.0, 138.9, 136.7, 136.2, 131.1, 129.2, 128.3, 127.4, 126.9, 126.5, 125.1, 125.4, 124.7, 124.6, 123.9, 57.6, 53.2, 42.3, 28.5, 27.9, 27.8, 26.8, 26.5, 25.4, 25.3, 2.4 (*C*-Si), 1.0 (*C*-Si).

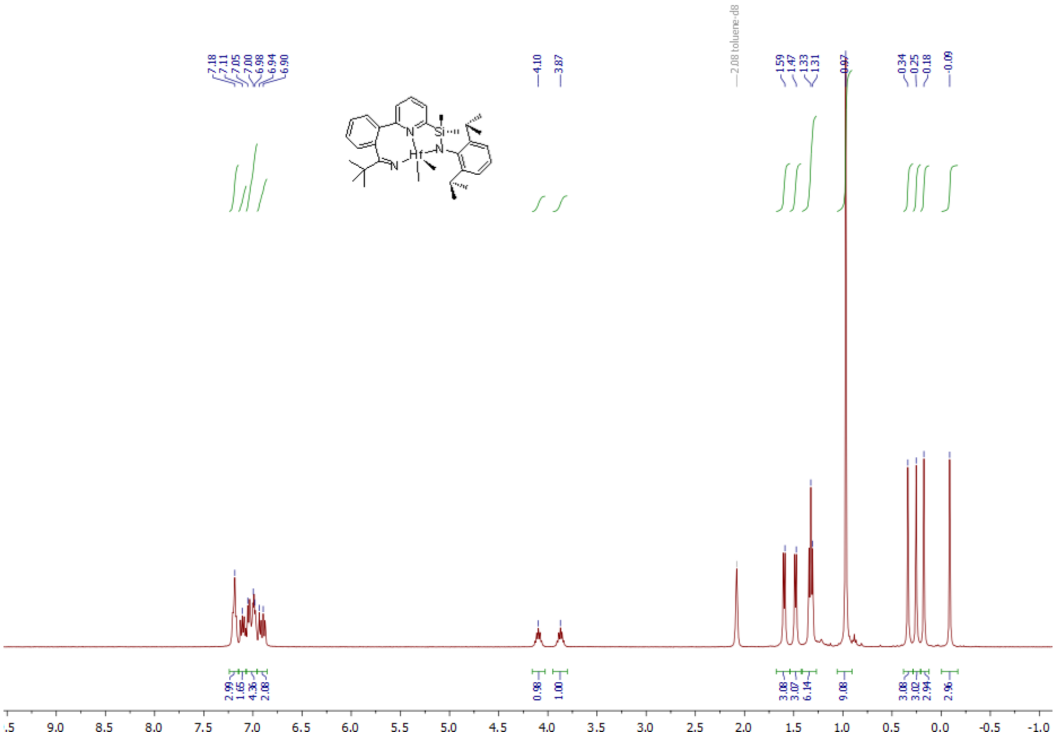

Figure S58. ^{1}H NMR spectrum of **1^{tBuCN}-HfMe$_2$** (obtained by method 1) in toluene-d_8 at room temperature.

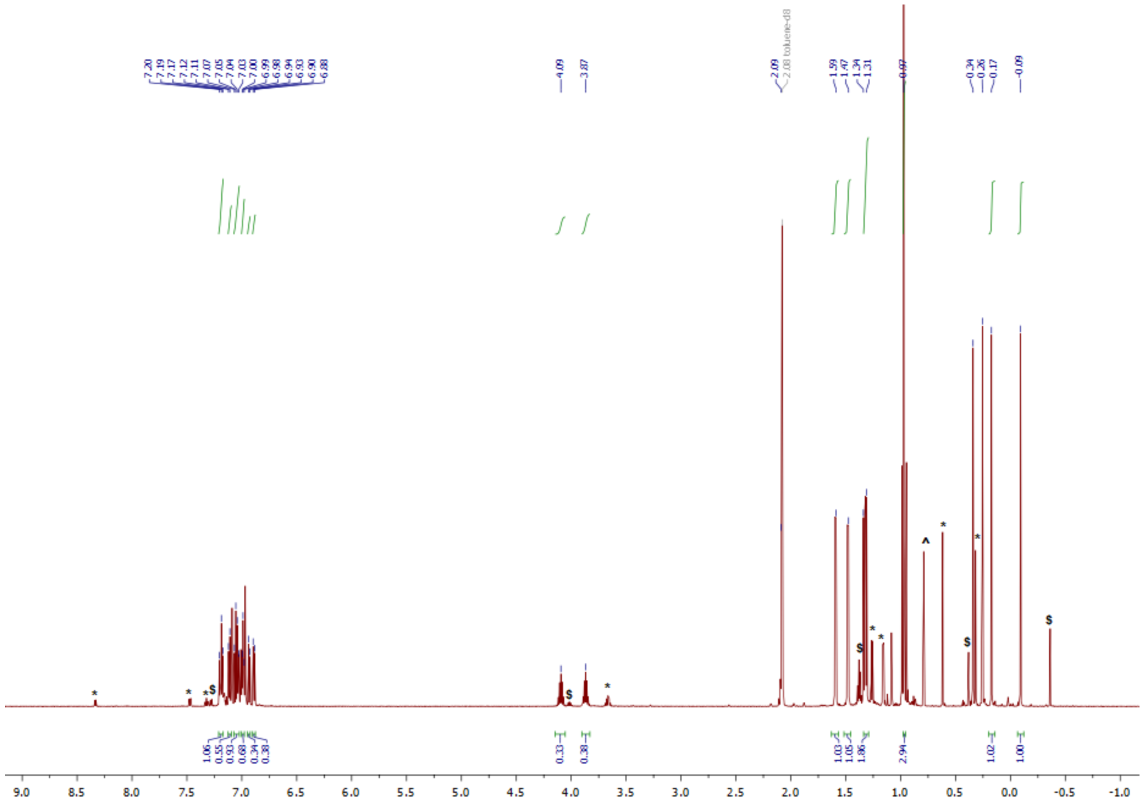

Figure S59. ^{1}H NMR spectrum of a mixture of **1-HfMe$_2$** with 1 equiv. of *t*BuCN in toluene-d_8 after 7 days at room temperature (method 2). A character (*****) denotes **1-HfMe$_2$**, (**$**) denotes **$1^{tBuCN}$-HfMe(N=CMe*t*Bu)**, and (**^**) denotes *t*BuCN.

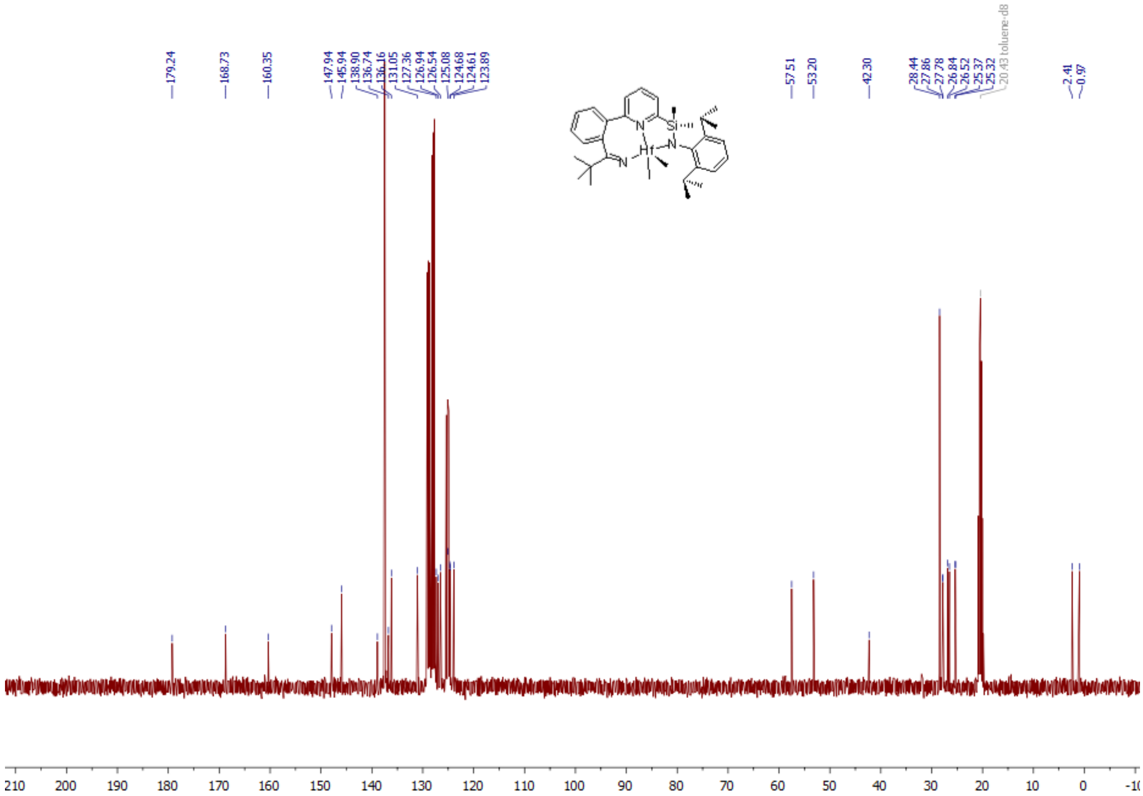

Figure S60. $^{13}C\{^{1}H\}$ NMR spectrum of **1**^{tBuCN}**-HfMe**$_2$ (obtained by method 1) in toluene-*d*$_8$ at room temperature.

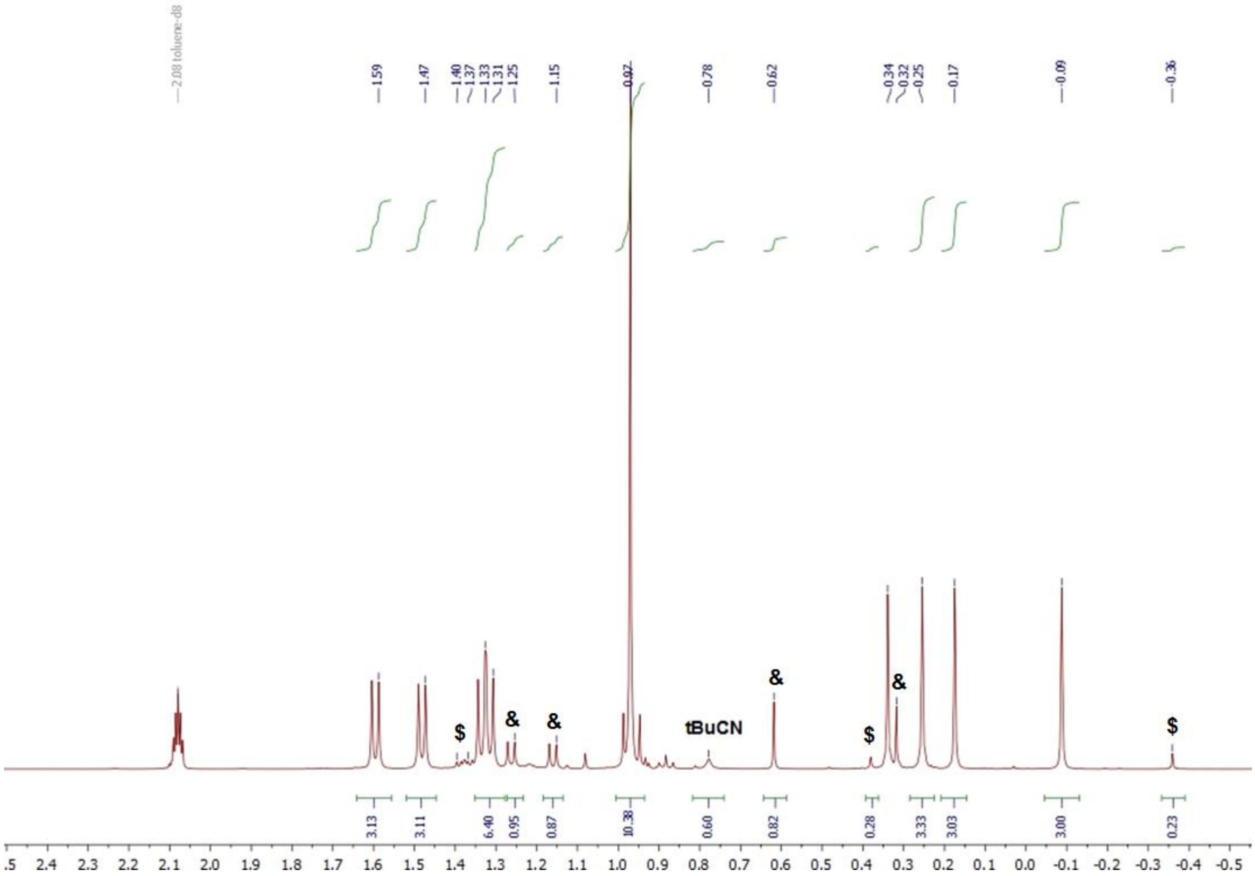

Figure S61. Fragment of ^{1}H NMR spectrum of complex **1**^{tBuCN}**-HfMe**$_2$ (obtained by method 1) after storing its solution in toluene-*d*$_8$ for a week at room temperature. Symbol (&) denotes **1-HfMe**$_2$; Symbol ($) denotes **1**^{tBuCN}**-HfMe(N=CMe*t*Bu)**.

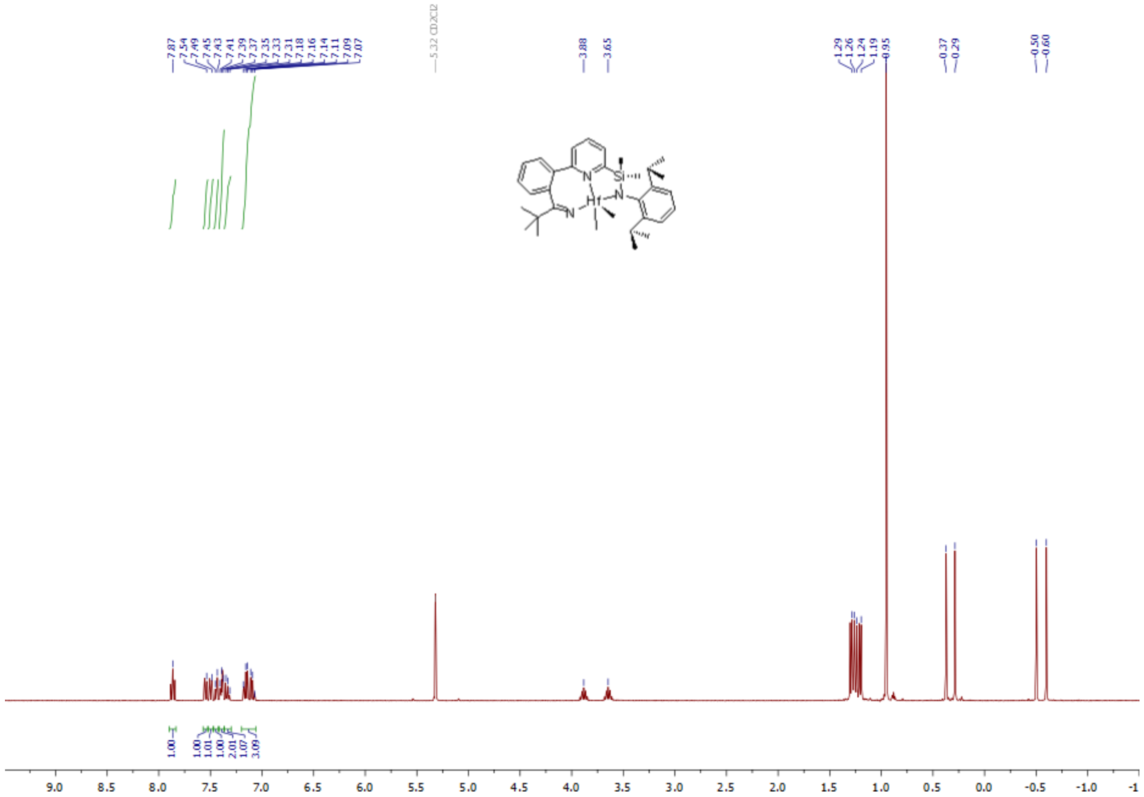

Figure S62. ^{1}H NMR spectrum of **1^{tBuCN}-HfMe$_2$** (obtained by method 1) in CD_2Cl_2 at room temperature.

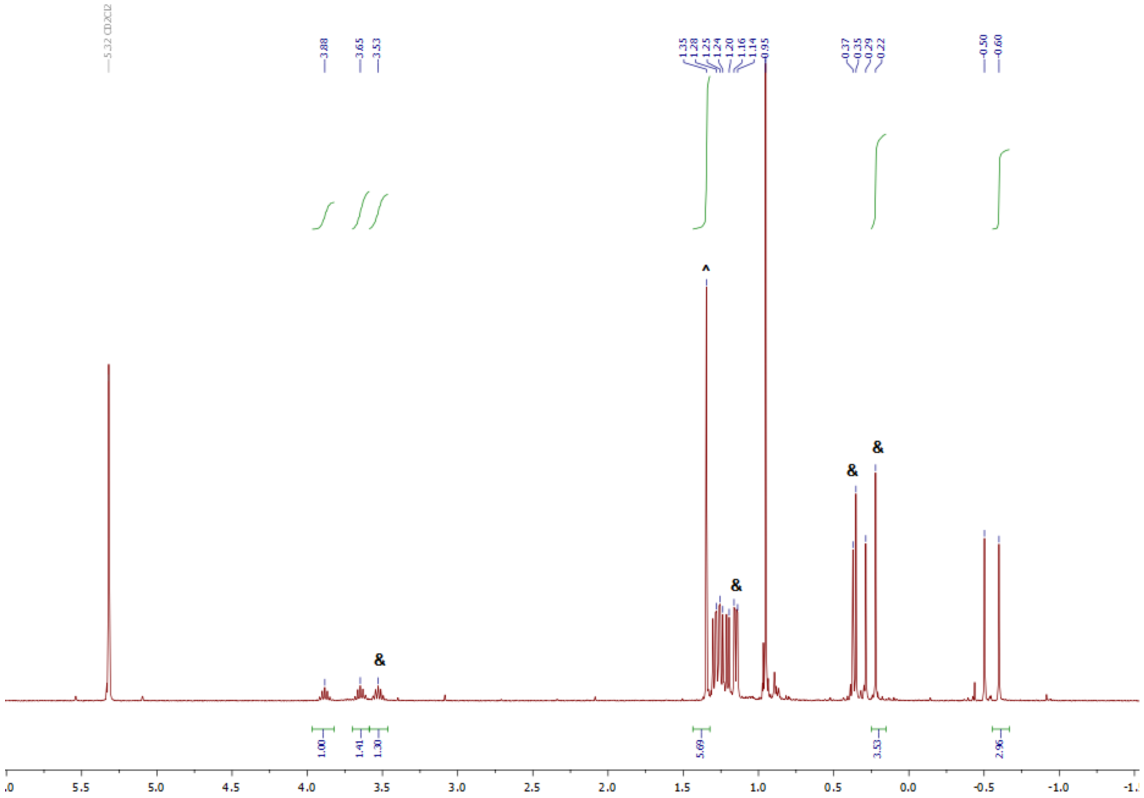

Figure S63. Fragment of ^{1}H NMR spectrum of complex **1^{tBuCN}-HfMe$_2$** (obtained by method 1) after storing its solution in CD_2Cl_2 for a week at room temperature. Symbol (&) denotes complex **1-HfMe$_2$**; symbol (^) denotes *t*BuCN. Estimated **1-HfMe$_2$**: **1^{tBuCN}-HfMe$_2$** ratio ≈ 1:2.

2.13. Complex 1-HfMe(N=CMe*t*Bu)

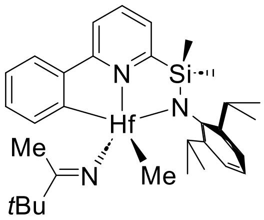

Complex **1-HfMe₂** (30.0 mg, 50 µmol) was dissolved in toluene-d_8 (0.6 mL) in a J Young NMR tube, and then trimethylacetonitrile (5.6 µL, 4.2 mg, 50 µmol) was added in one portion via Eppendorf pipette. The resulting yellow solution was heated for six days at 100 °C and then analyzed by NMR spectroscopy.

^{1}H NMR (400 MHz, toluene-d_8) δ_H: 8.14 (1H, m), 7.53 (1H, d, $^3J_{HH}$ = 7.8 Hz), 7.29 (1H, m), 7.21 (1H, m), 7.16 (1H, m), 7,07–7.12 (3H, m), 7.02 (1H, m), 6.97 (1H, m), 3.84 (1H, sept, $^3J_{HH}$ = 6.9 Hz, ArC*H*Me₂), 3.36 (1H, sept, $^3J_{HH}$ = 6.9 Hz, ArC*H*Me₂), 1.28 (6H, d, $^3J_{HH}$ = 6.9 Hz, ArCH*Me*₂), 1.27 (3H, s, *Me*C(*t*Bu)=N), 1.13 (9H, s, *tBu*), 1.04 (3H, d, $^3J_{HH}$ = 6.9 Hz, m, ArCH*Me*₂), 1.03 (3H, d, $^3J_{HH}$ = 6.9 Hz, ArCH*Me*₂), 0.6 (3H, s, *Me*₂Si), 0.42 (3H, s, *Me*Hf), 0.14 (3H, s, *Me*₂Si).

^{13}C{^{1}H} NMR (101 MHz, toluene-d_8) δ_C: 201.0 (*C*-Hf), 191.9, 171.0, 164.6, 147.1, 145.2, 143.9, 143.8, 141.2, 138.9, 130.1, 129.2, 126.7, 123.8, 123.4, 123.3, 123.0, 118.2, 50.7, 46.0, 28.6, 28.5, 28.1, 27.8, 27.7, 26.6, 26.5, 25.6, 25.2, 23.2, 2.3(*Me*₂Si), -1.0 (*Me*₂Si).

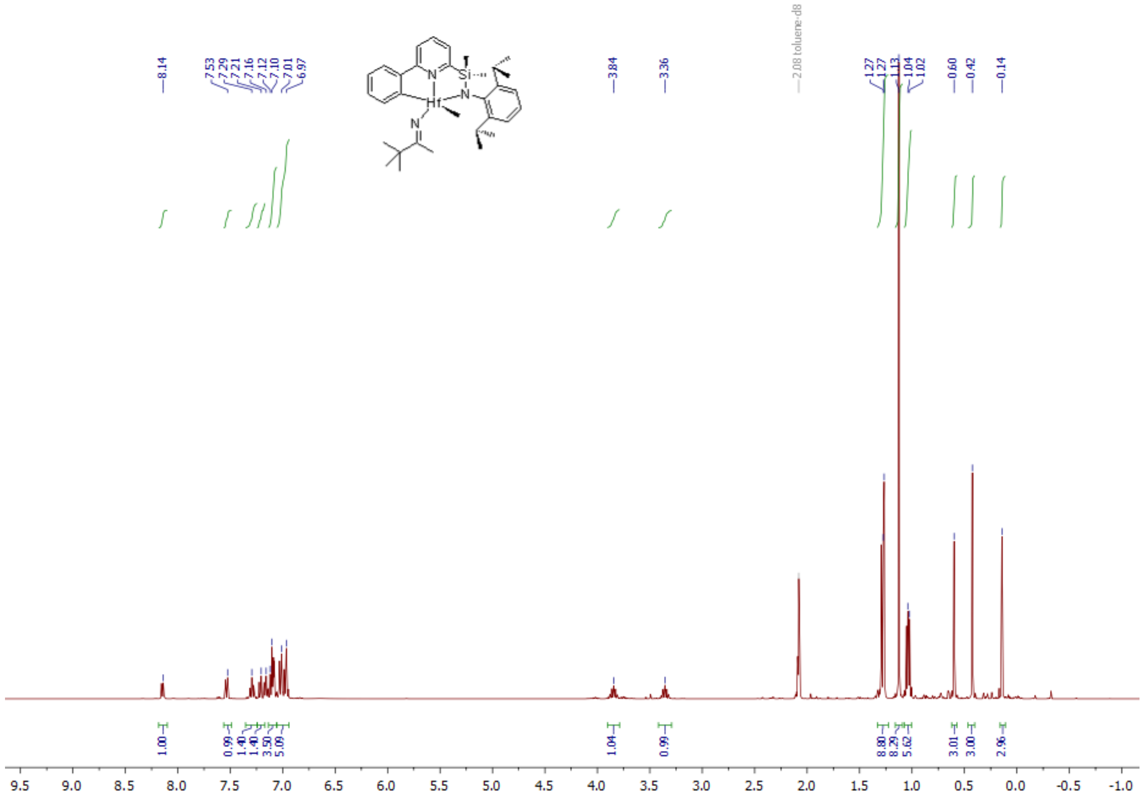

Figure S64. ^{1}H NMR spectrum of complex **1-HfMe(N=CMe*t*Bu)** in toluene-d_8 at room temperature.

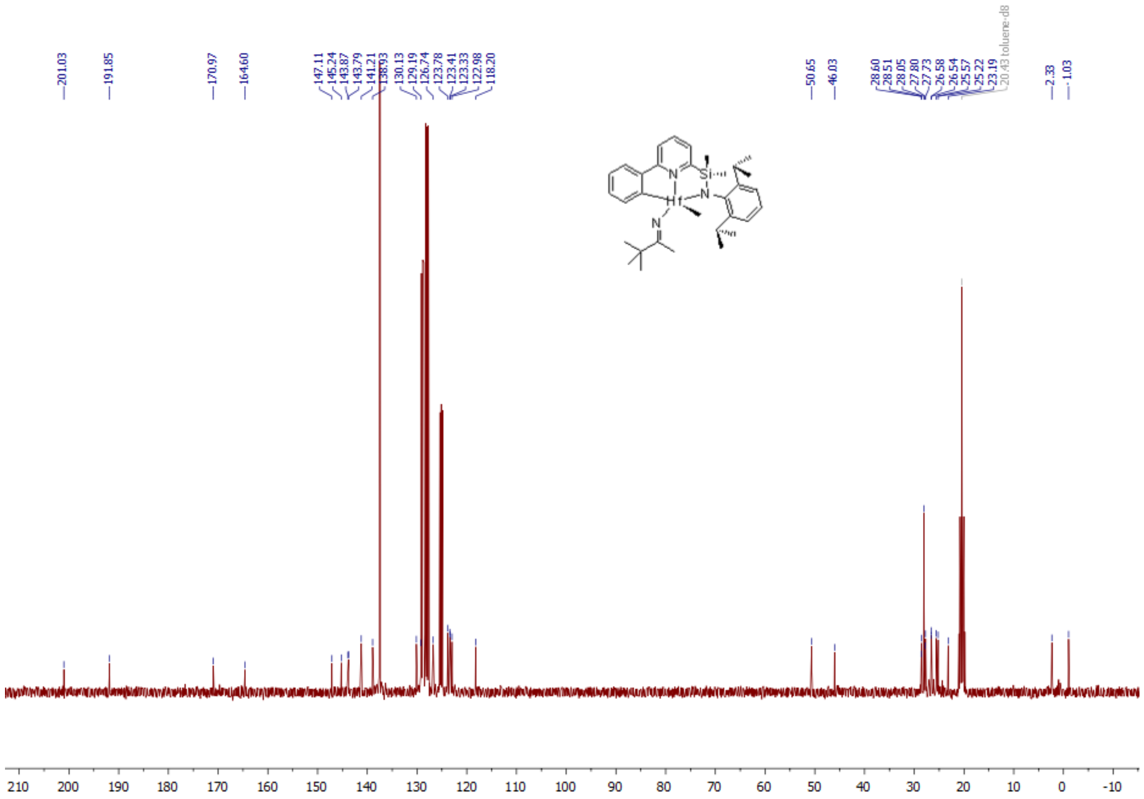

Figure S65. $^{13}C\{^{1}H\}$ NMR spectrum of complex **1-HfMe(N=CMe*t*Bu)** in toluene-d_8 at room temperature.

2.14. Complex 1^{tBuCN}-HfMe(N=CMe*t*Bu)

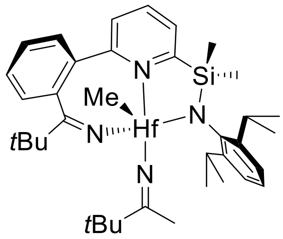

Complex **1-HfMe$_2$** (20.0 mg, 34 µmol) was dissolved in toluene-d_8 (0.6 mL) in a J Young NMR tube, and then trimethylacetonitrile (7.4 µL, 5.6 mg, 68 µmol) was added in one portion via Eppendorf pipette. The obtained yellow solution was kept at room temperature for a week, then heated for 32 h at 100 °C cooled and left at room temperature for an additional 24 h. The resulting yellow solution was analyzed by NMR spectroscopy.

^{1}H NMR (400 MHz, toluene-d_8) δ_H: 7.28 (1H, m), 7.14–7.18 (2H, m), 7.11 (2H, m), 7.04–7.07 (2H, m), 6.96–6.99 (2H, m), 4.02 (1H, sept, $^3J_{HH}$ = 6.8 Hz, ArC*H*Me$_2$), 3.88 (1H, sept, $^3J_{HH}$ = 6.8 Hz, ArC*H*Me$_2$), 1.39 (3H, d, $^3J_{HH}$ = 6.8 Hz, ArCH*Me*$_2$), 1.38 (3H, d, $^3J_{HH}$ = 6.8 Hz, ArCH*Me*$_2$), 1.37 (3H, d, $^3J_{HH}$ = 6.8 Hz, ArCH*Me*$_2$), 1.31 (3H, d, $^3J_{HH}$ = 6.8 Hz, ArCH*Me*$_2$), 1.08 (3H, s, *Me*C(*t*Bu)=N), 0.99 (9H, s, *t*Bu), 0.95 (9H, s, *t*Bu), 0.38 (3H, s, *Me*$_2$Si), 0.26 (3H, s, *Me*$_2$Si), -0.36 (3H, s, *Me*Hf).

^{13}C{^{1}H} NMR (101 MHz, toluene-d_8) δ_C: 188.1, 175.8, 168.9, 160.1, 147.9, 145.8, 143.9, 136.8, 136.3, 131.5, 127.5, 127.0, 126.3, 123.9, 123.8, 123.7, 123.0, 45.6, 45.5, 42.0, 28.6, 27.8, 27.72, 27.69, 26.7, 26.4, 25.4, 24.5, 24.4, 2.3, 0.7.

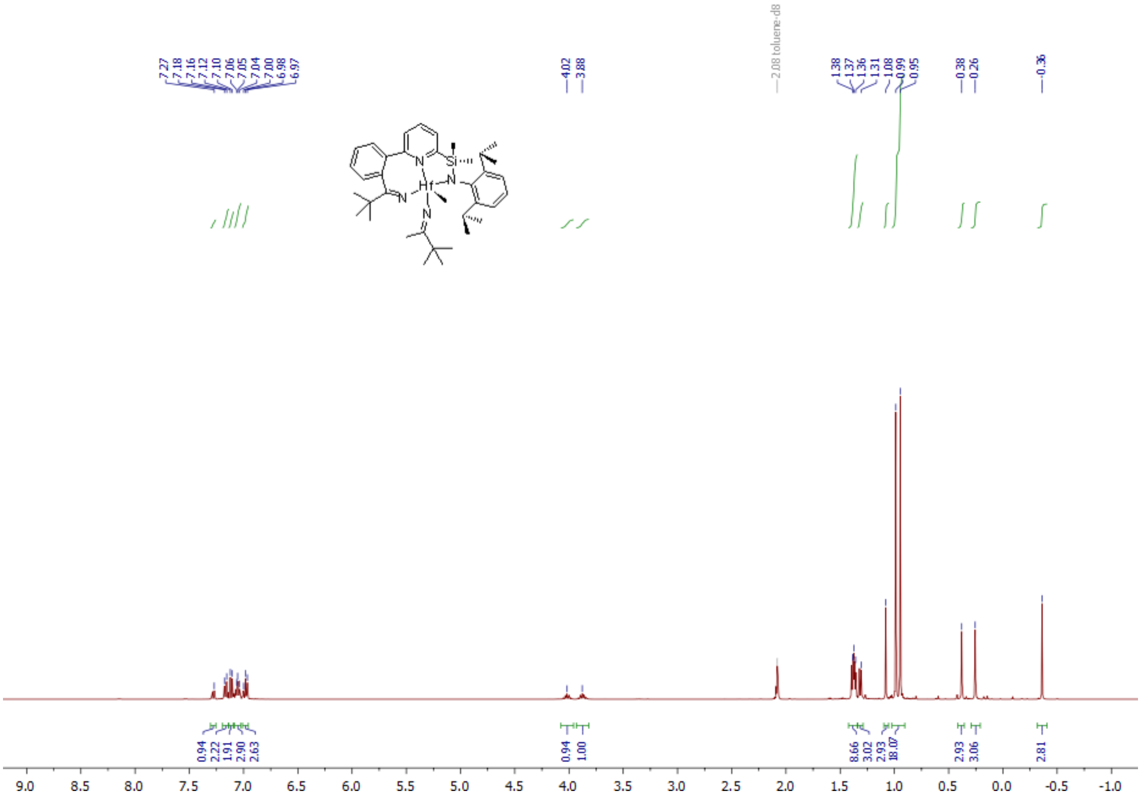

Figure S66. ^{1}H NMR spectrum of complex **1^{tBuCN}-HfMe(N=CMe*t*Bu)** in toluene-d_8 at room temperature.

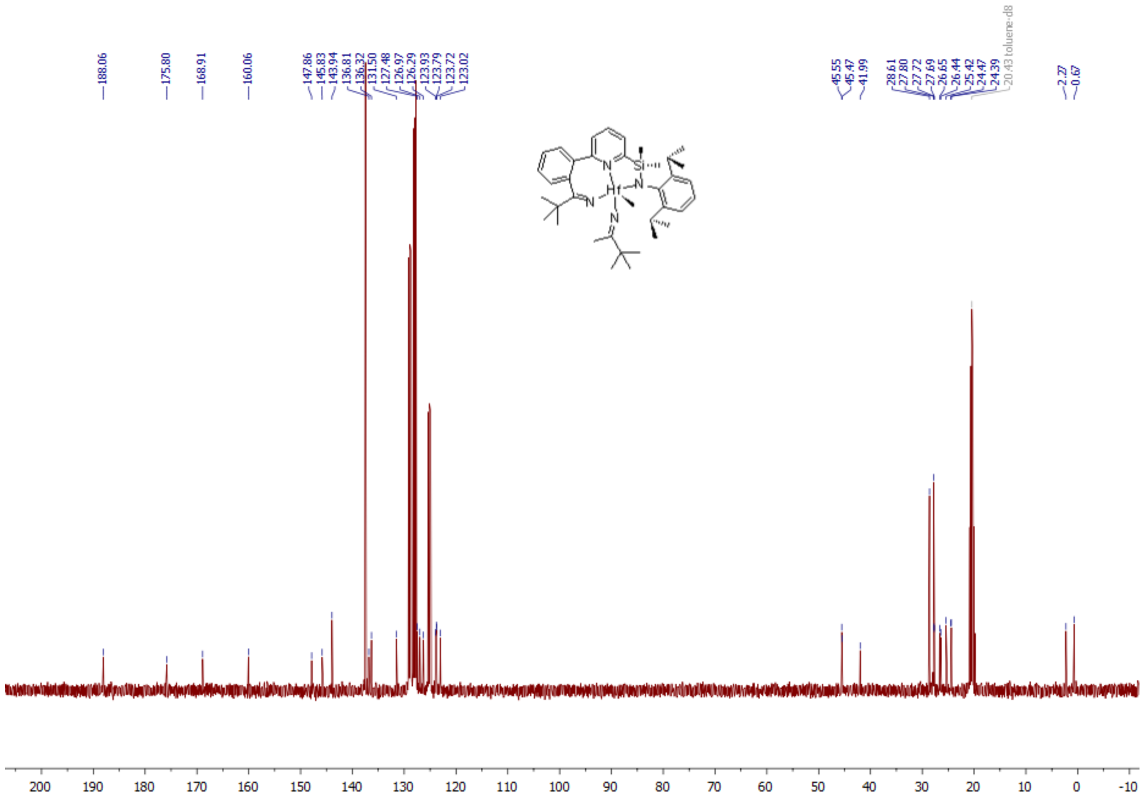

Figure S67. $^{13}C\{^1H\}$ NMR spectrum of complex **1^{tBuCN}-HfMe(N=CMe*t*Bu)** in toluene-d_8 at room temperature.

2.15. Complex 1^{PhCN}-$HfCl_2$

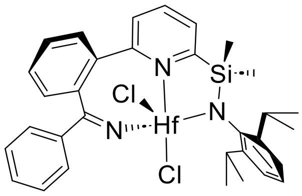

Complex **1-$HfCl_2$** (382 mg, 0.6 mmol) was dissolved in CH_2Cl_2 (20 mL), and then benzonitrile (60 µL, 62 mg, 0.60 mmol) was added in one portion via Eppendorf pipette at room temperature. The resulting bright yellow solution was stirred for 4 h at room temperature, then all volatiles were evaporated. The residue was washed with hexane (20 mL) and dried in vacuum giving **1^{PhCN}-$HfCl_2$** as a bright yellow solid (332 mg, 75%) of. Anal. calc. for $C_{32}H_{35}Cl_2HfN_3Si$: C, 52.00; H, 4.77; N, 5.69. Found: C, 52.41; H, 5.08; N, 5.44.

^{1}H NMR (400 MHz, CD_2Cl_2): δ_H 7.99 (1H, m), 7.69 (3H, m), 7.65 (1H, d, $^{3}J_{HH}$ = 8.0 Hz), 7.55–7.60 (2H, m), 7.49 (1H, m), 7.44 (2H, m), 7.36 (2H, m), 7.14–7.24 (3H, m), 3.76 (1H, sept, $^{3}J_{HH}$ = 6.7 Hz, ArC*H*Me$_2$), 3.70 (1H, sept, $^{3}J_{HH}$ = 6.7 Hz, ArC*H*Me$_2$), 1.57 (3H, d, $^{3}J_{HH}$ = 6.7 Hz, ArCH*Me*$_2$), 1.36 (3H, d, $^{3}J_{HH}$ = 6.7 Hz, ArCH*Me*$_2$), 1.27 (6H, m, ArCH*Me*$_2$), 0.45 (3H, s, *Me*2Si), 0.32 (3H, s, *Me*2Si).

$^{13}C\{^{1}H\}$ NMR (101 MHz, CD_2Cl_2): δ_C 171.9, 167.3, 158.6, 145.2, 145.0, 144.6, 141.8, 138.6, 138.5, 137.4, 132.9, 131.6, 130.1, 129.6, 129.2, 129.0, 128.9, 128.6, 126.4, 125.4, 124.57, 124.56, 28.1, 27.8, 26.9, 26.5, 25.1, 24.6, 1.8 (*Me*2Si), 0.4 (*Me*2Si).

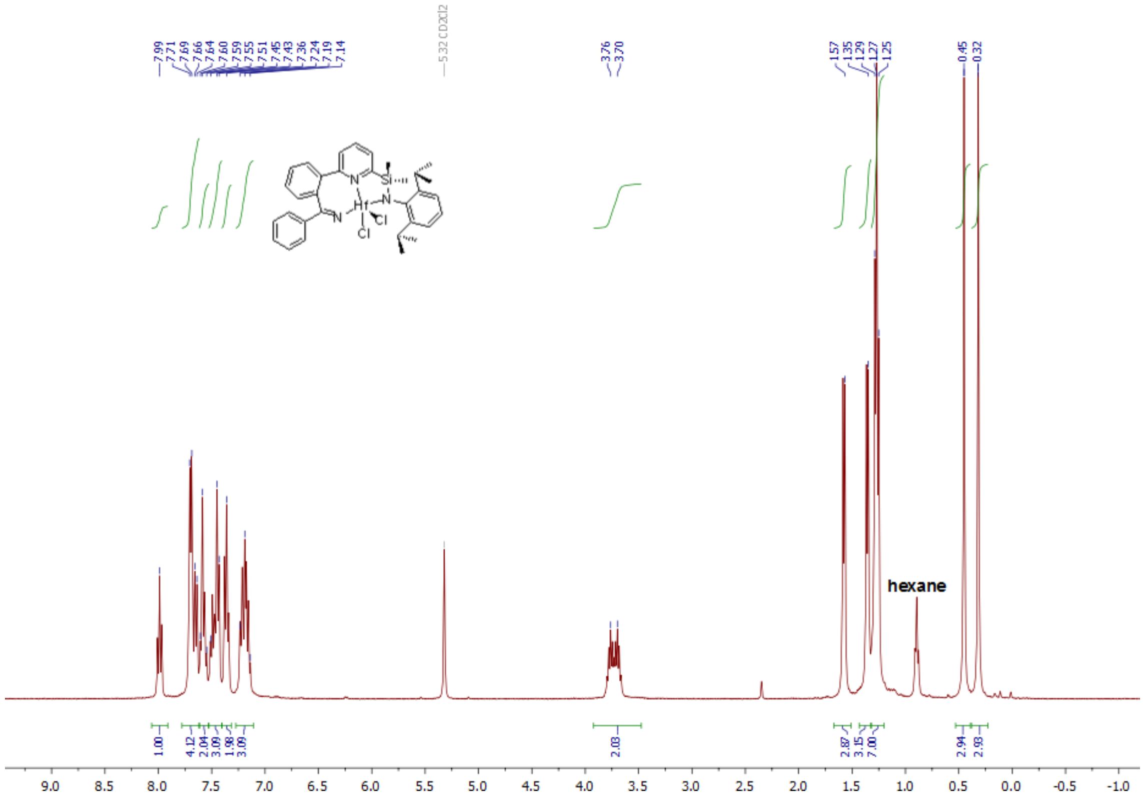

Figure S68. ^{1}H NMR spectrum of complex **1^{PhCN}-$HfCl_2$** in CD_2Cl_2 at room temperature.

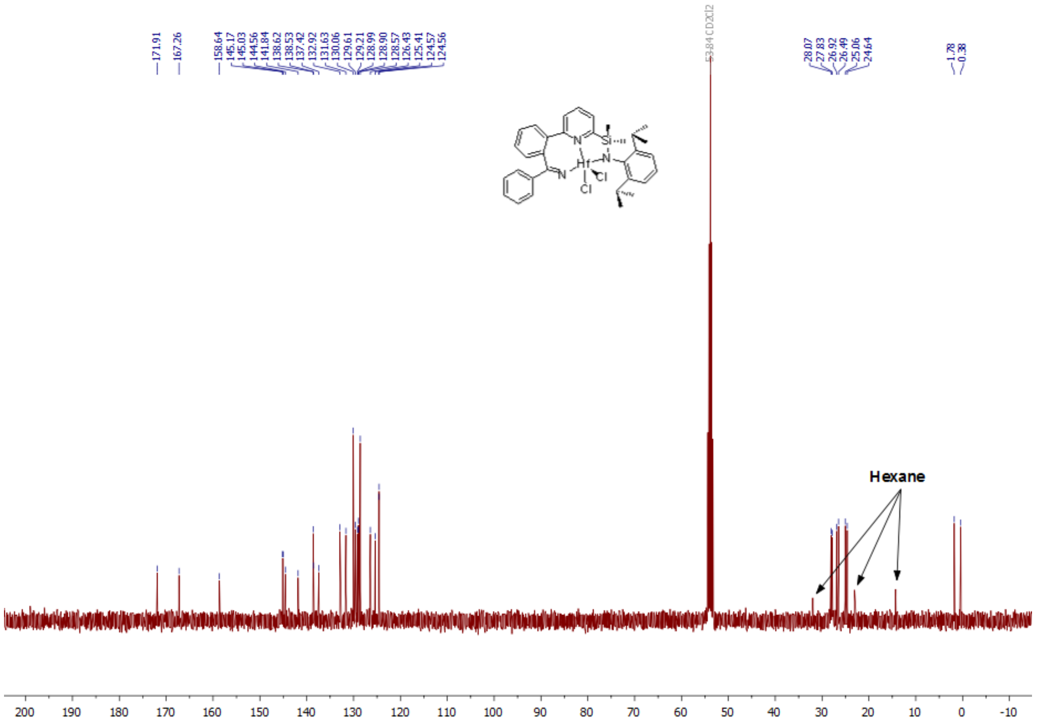

Figure S69. $^{13}C\{^1H\}$ NMR spectrum of complex **1^{PhCN}-$HfCl_2$** in CD_2Cl_2 at room temperature.

2.16. Complex 1^{PhCN}-HfMe$_2$

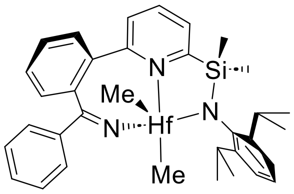

Method 1: Complex **1^{PhCN}-HfCl$_2$** (332 mg, 0.449 mmol) was dissolved in CH_2Cl_2 (30 mL), and then a solution of MeMgBr in diethyl ether (0.3 mL, 1.08 mmol, 3.6 M) was added in one portion via glass syringe at -30 °C. The resulting mixture was allowed to slowly warm and stirred for 4 h at room temperature. Next, all volatiles were evaporated, and the residue was extracted with CH_2Cl_2 (20 mL) filtering the formed suspension through a short pad of Celite®. Afterwards, hexane (30 mL) was added to the obtained orange filtrate, and the solution was evaporated until precipitation occurred. The precipitate was filtered off, washed with cold pentane (5 mL, kept in freezer at -30 °C) and cold diethyl ether (5 mL, kept in freezer at -30 °C), and then dried in vacuum giving **1^{PhCN}-HfMe$_2$** as a pale orange solid (259 mg, 83%). Anal. calc. for $C_{34}H_{41}HfN_3Si$: C, 58.48; H, 5.92; N, 6.02. Found: C, 58.77; H, 6.20; N, 5.74.

Method 2: Complex **1-HfMe$_2$** (20 mg, 34 µmol) was dissolved in toluene-d_8 (0.6 mL) in J Young NMR tube, and then benzonitrile (3.5 µL, 3.5 mg, 34 µmol) was added in one portion via Eppendorf pipette. After 2h, the obtained orange solution was analyzed by NMR spectroscopy.

^{1}H NMR (400 MHz, toluene-d_8) δ_H: 7.82 (2H, m), 7.22 (2H, m), 7.15 (2H, m), 6.96–7.11 (6H, m), 6.92 (1H, m), 6.88 (2H, m), 4.17 (1H, m, ArC*H*Me$_2$), 3.77 (1H, m, ArC*H*Me$_2$), 1.70 (3H, d, $^3J_{HH}$ = 6.7 Hz, ArCH*Me*$_2$), 1.55 (3H, d, $^3J_{HH}$ = 6.8 Hz, ArCH*Me*$_2$), 1.33 (3H, d, $^3J_{HH}$ = 6.7 Hz, ArCH*Me*$_2$), 1.25 (3H, d, $^3J_{HH}$ = 6.8 Hz, ArCH*Me*$_2$), 0.39 (3H, s, *Me*$_2$Hf), 0.28 (3H, s, *Me*$_2$Hf), 0.16 (3H, s, *Me*$_2$Si), 0.05 (3H, s, *Me*$_2$Si).

^{13}C{^{1}H} NMR (101 MHz, toluene-d_8) δ_C: 168.6, 168.5, 160.0, 146.2 145.9, 145.8, 139.8, 138.9, 138.5, 136.3, 131.9, 130.5, 129.6, 129.2, 127.6, 127.2, 125.9, 124.7, 56.5, 53.9, 28.0, 27.9, 27.2, 26.2, 25.7, 25.0, 3.4 (*Me*$_2$Si), -0.1 (*Me*$_2$Si).

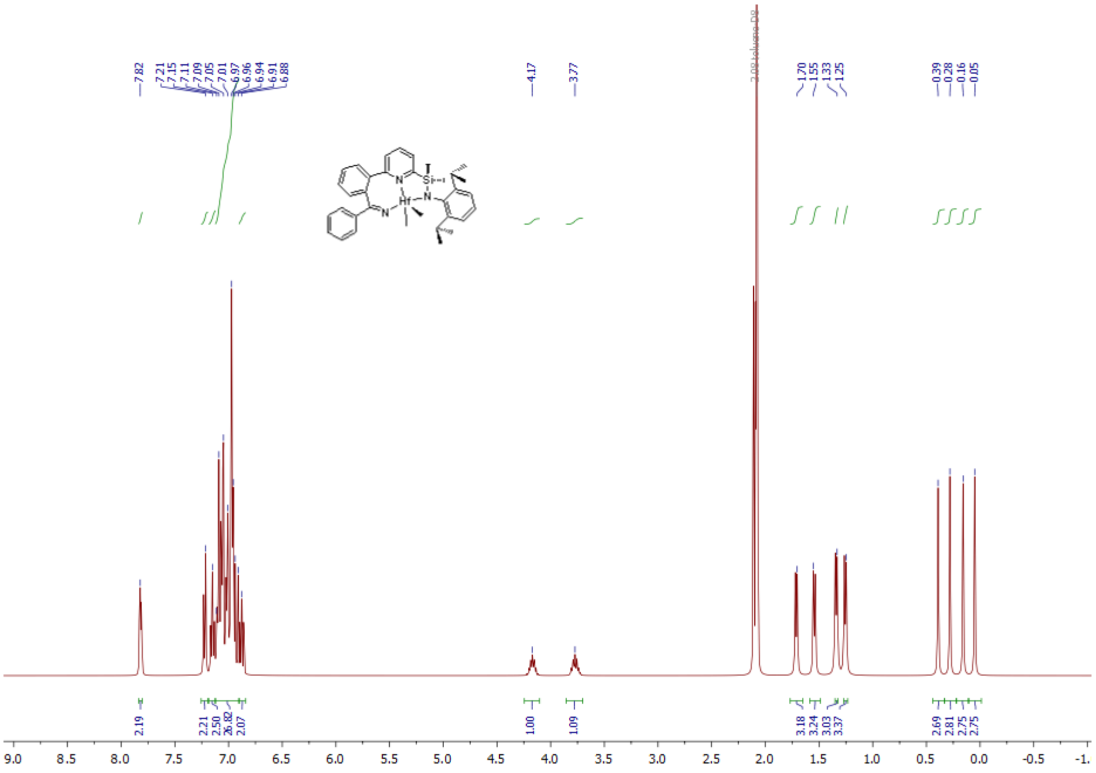

Figure S70. ^{1}H NMR spectrum of complex $\mathbf{1}^{\mathbf{PhCN}}$**-HfMe$_2$** (obtained by method 1) in toluene-d_8 at room temperature. The excessive integrals in aromatic region belong to the residue of toluene.

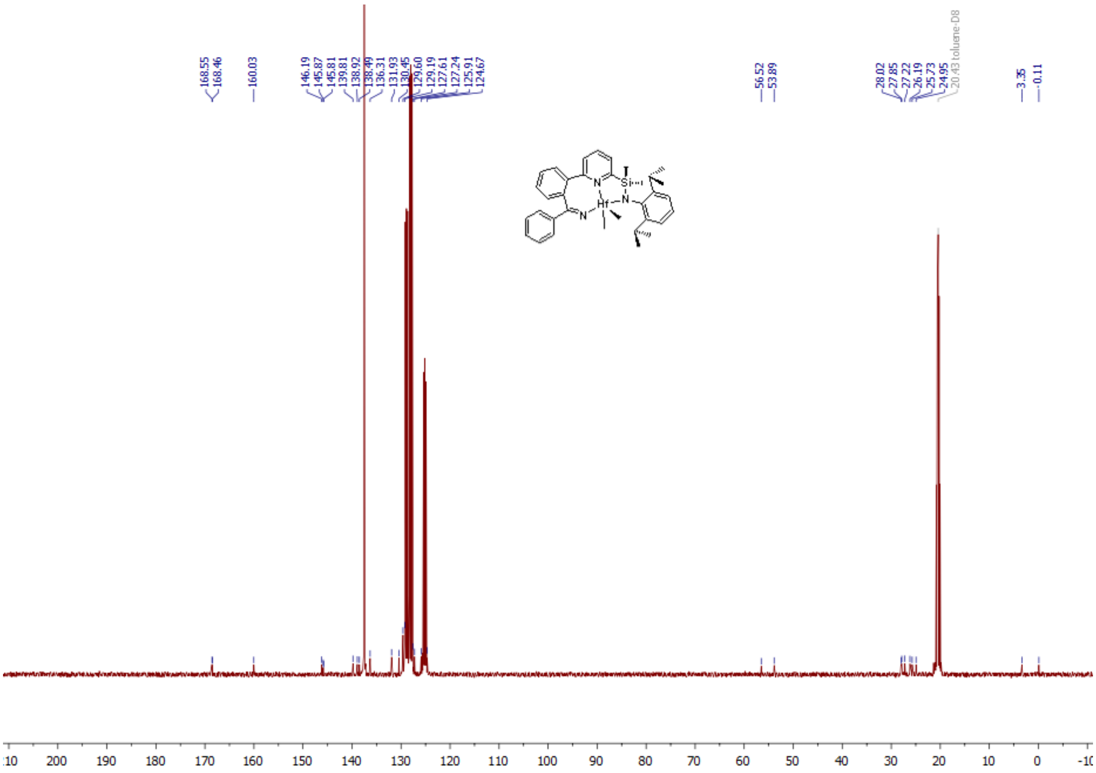

Figure S71. ^{13}C{^{1}H} NMR spectrum of complex $\mathbf{1}^{\mathbf{PhCN}}$**-HfMe$_2$** (obtained by method 1) in toluene-d_8 at room temperature.

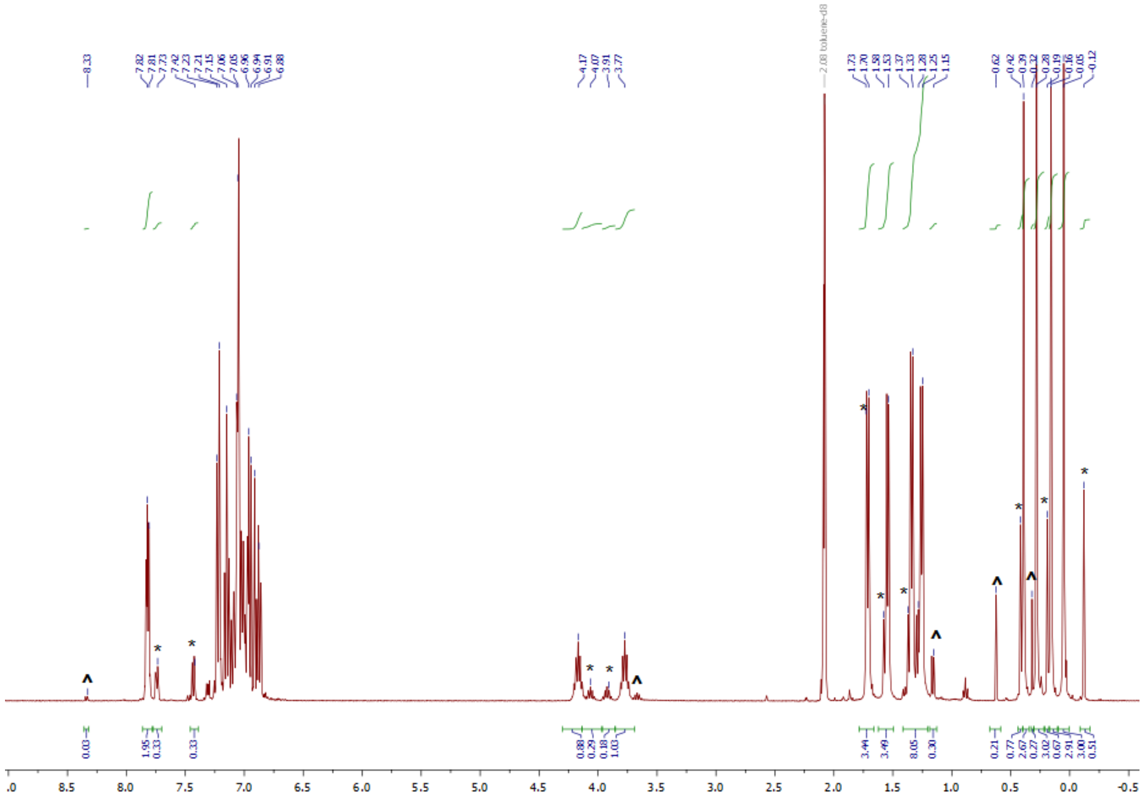

Figure S72. ^{1}H NMR spectrum of a mixture of **1-HfMe$_2$** with 1 equiv. of PhCN (obtained by method 2) in toluene-d_8 at room temperature. Characters (*****) and (**^**) denote **1-HfMe(N=CMePh)** and **1-HfMe$_2$**, respectively.

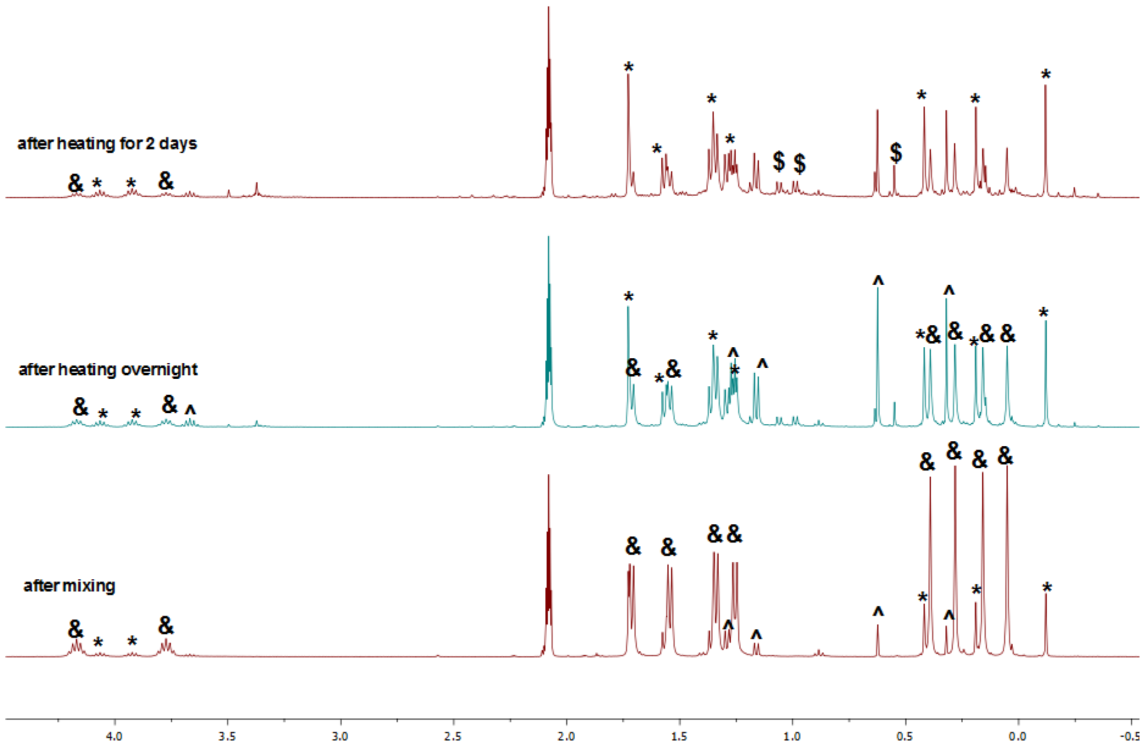

Figure S73. Fragments of ^{1}H NMR spectra of a mixture of **1-HfMe$_2$** with 1 equiv. of PhCN in toluene-d_8, from bottom to top: right after mixing, after heating overnight at 100 °C, and after heating for 2 days at 100 °C. A character (**$**) denotes possible signals of **1-HfMe(N=CMePh)**, (**^**) – signals of **1-HfMe$_2$**, (*****) – **1^{PhCN}-HfMe(N=CMePh)**, and (**&**) – **1^{PhCN}-HfMe$_2$**.

2.17. Complex 1^{PhCN}-HfMe(N=CMePh)

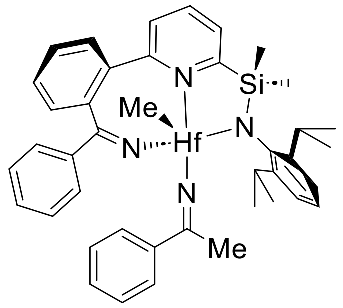

Method 1: Complex **1-HfMe$_2$** (150 mg, 0.252 mmol) was dissolved in mixture of CH_2Cl_2 (10 mL) and hexane (10 mL), and then benzonitrile (52.0 µL, 52.0 mg, 0.504 mmol) was added in one portion via Eppendorf pipette at room temperature. The obtained orange solution was stirred for 3 h at room temperature, and then the solvents were evaporated until precipitation occurred. The precipitate was filtered off, washed with cold hexane (5 mL, kept in freezer at -30 °C), and dried under vacuum giving **1^{PhCN}-HfMe(N=CMePh)** as a yellow solid (147 mg, 73%). Anal. calc. for $C_{41}H_{46}HfN_4Si$: C, 61.45; H, 5.79; N, 6.99. Found: C, 61.72; H, 6.03; N, 6.74.

Method 2: Complex **1-HfMe$_2$** (20.0 mg, 34 µmol) in toluene-d_8 (0.6 mL) J Young NMR tube, and then benzonitrile (6.9 µL, 6.9 mg, 68 µmol) was added in one portion via Eppendorf pipette. The obtained orange solution was left to stay overnight at room temperature and then analyzed by NMR spectroscopy.

^{1}H NMR (400 MHz, toluene-d_8) δ_H: 7.73–7.75 (m, 2H), 7.44 (m, 2H), 7.30–7.32 (m, 1H), 7.24 (m, 1H), 7.18–7.21 (m, 1H), 7.13 (m, 1H), 7.04–7.10 (m, 3H), 6.97–7.03 (m, 9H), 4.07 (sept, $^3J_{HH}$ = 6.7 Hz, 1H, ArC*H*Me$_2$), 3.92 (sept, $^3J_{HH}$ = 6.7 Hz, 1H, ArC*H*Me$_2$), 1.73 (3H, s, *Me*C(Ph)=N), 1.57 (3H, d, $^3J_{HH}$ = 6.7 Hz, ArCH*Me*$_2$), 1.35 (6H, m, ArCH*Me*$_2$), 1.29 (3H, d, $^3J_{HH}$ = 6.8 Hz, ArCH*Me*$_2$), 0.42 (3H, s, *Me*$_2$Si), 0.19 (3H, s, *Me*$_2$Si), -0.12 (3H, s, *Me*Hf).

^{13}C{^{1}H} NMR (101 MHz, toluene-d_8) δ_C: 173.8, 168.6, 166.4, 159.5, 146.1, 145.5, 144.2, 144.1, 141.5, 140.2, 138.3, 136.6, 132.3, 130.0, 129.9, 128.2, 128.13, 128.08, 127.9, 127.5, 127.4, 125.5, 124.09, 124.06, 123.4, 45.7, 28.7, 28.1, 27.8, 27.1, 26.3, 24.7, 24.2, 2.9 (*Me*$_2$Si), -0.1 (*Me*$_2$Si).

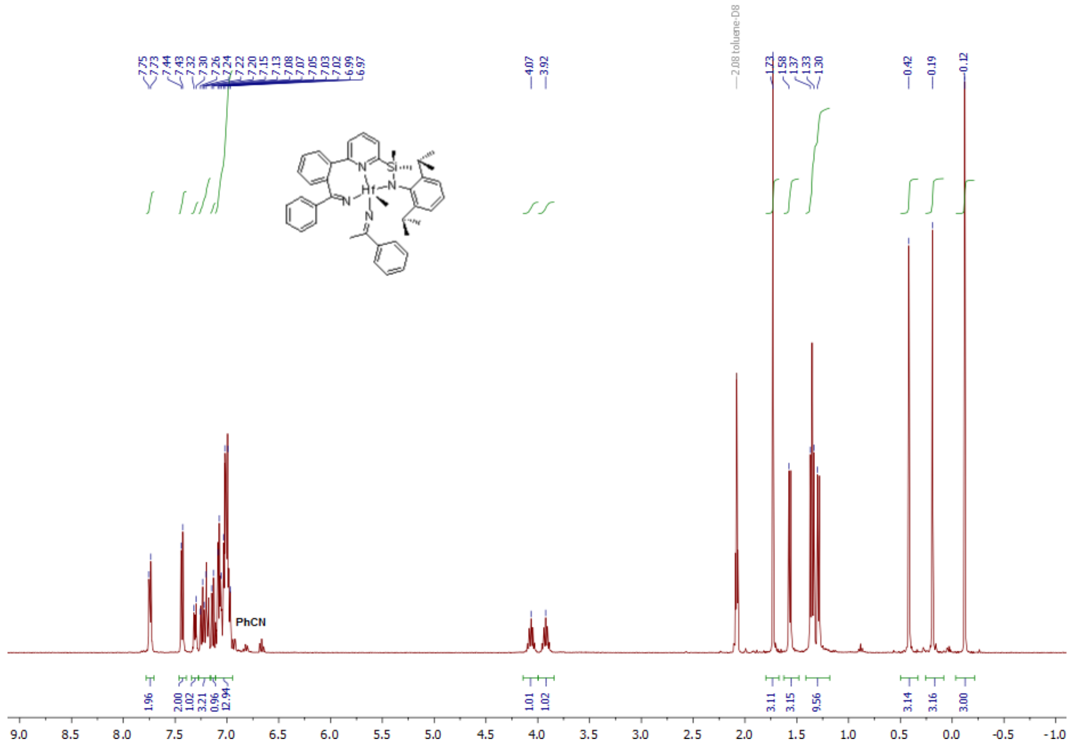

Figure S74. ^{1}H NMR spectrum of **1^{PhCN}-HfMe(N=CMePh)** (obtained by method 1) in toluene-*d$_8$* at room temperature.

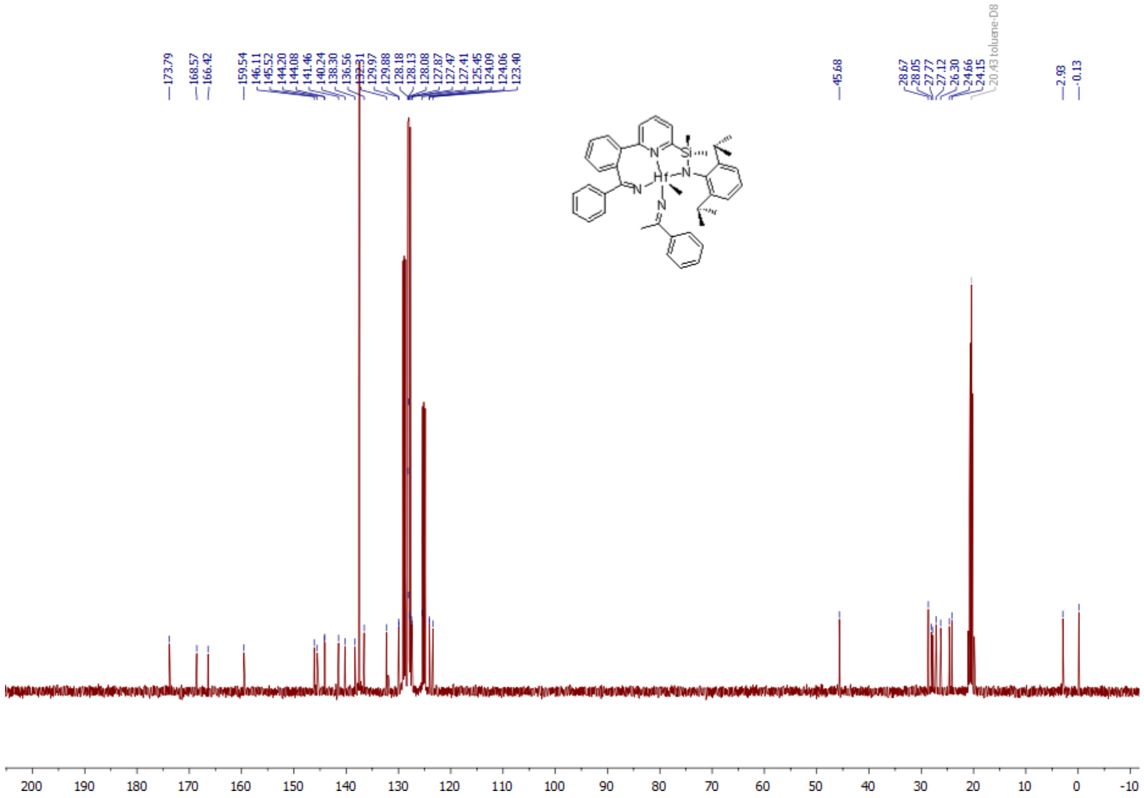

Figure S75. $^{13}C\{^{1}H\}$ NMR spectrum of complex **1^{PhCN}-HfMe(N=CMePh)** (obtained by method 1) in toluene-*d$_8$* at room temperature.

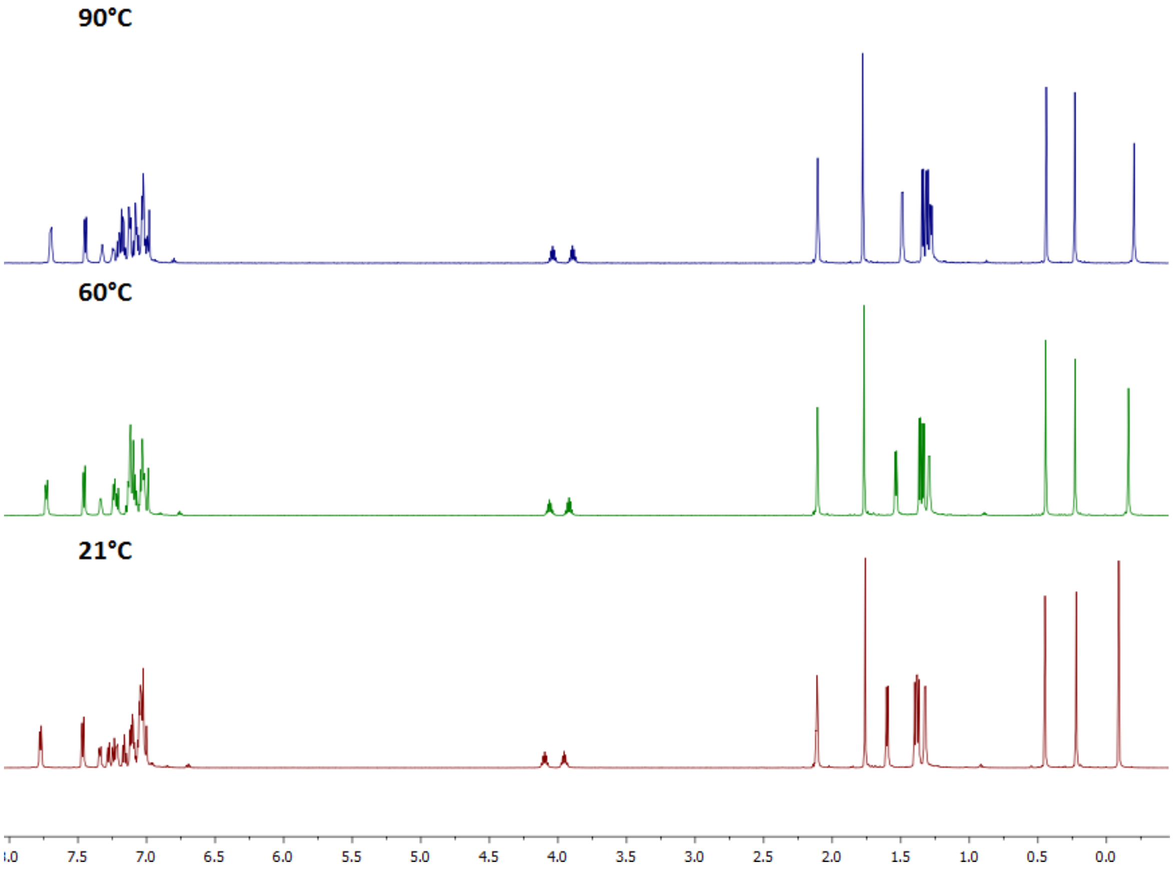

Figure S76. ^{1}H NMR spectra of **1^{PhCN}-HfMe(N=CMePh)** in toluene-d_8 at 21, 60 and 90 °C (from bottom to top). No *β*-aryl elimination was observed in this case.

2.18. Complex 2-$HfCl_2$

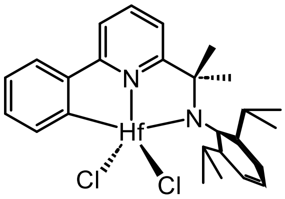

2,6-Diisopropyl-*N*-(2-(6-phenylpyridin-2-yl)propan-2-yl)aniline (1.0 g, 2.7 mmol) was dissolved in toluene (200 mL) in a pressure vessel, and then $HfCl_2Bn_2(Et_2O)$ (1.25 g, 2.7 mmol) was added in one portion. The obtained yellow solution was stirred in a pressure bottle at 75 °C overnight, then cooled down to room temperature and filtered through a short pad of Celite®. Afterwards, all volatiles were evaporated, and the residue was washed with hot hexane (30 mL) and then recrystallized from a toluene-hexane mixture giving **2-$HfCl_2$** as a yellowish crystalline solid (1.05 g, 63%). The mother liquor was concentrated, and the residue was recrystallized from toluene giving another portion of **2-$HfCl_2$** (0.25 g, 15%; combined yield 1.3 g, 78%). Anal. calc. for $C_{26}H_{30}Cl_2HfN_2$: C, 50.37; H, 4.88; N, 4.52. Found: C, 50.48; H, 5.01; N, 4.40.

^{1}H NMR (400 MHz, CD_2Cl_2) δ_H: 8.06–8.10 (2H, m), 7.89 (1H, m), 7.85 (1H, m), 7.42 (1H, m), 7.39 (2H, m), 7.22–7.30 (3H, m), 3.36 (2H, sept, $^3J_{HH}$ = 6.8 Hz, ArC*H*Me$_2$), 1.48 (6H, s, *Me*$_2$C), 1.24 (6H, d, $^3J_{HH}$ = 6.8 Hz, ArCH*Me$_2$*), 1.21 (6H, d, $^3J_{HH}$ = 6.8 Hz, ArCH*Me$_2$*).

^{13}C{^{1}H} NMR (101 MHz, CD_2Cl_2) δ_C: 198.1 (C_{Ar}-Hf), 174.9, 163.3, 149.6, 147.1, 143.2, 141.3, 139.6, 130.6, 130.2, 127.4, 125.0, 123.6, 119.2, 116.9, 74.1, 30.5, 28.8, 26.7, 24.5.

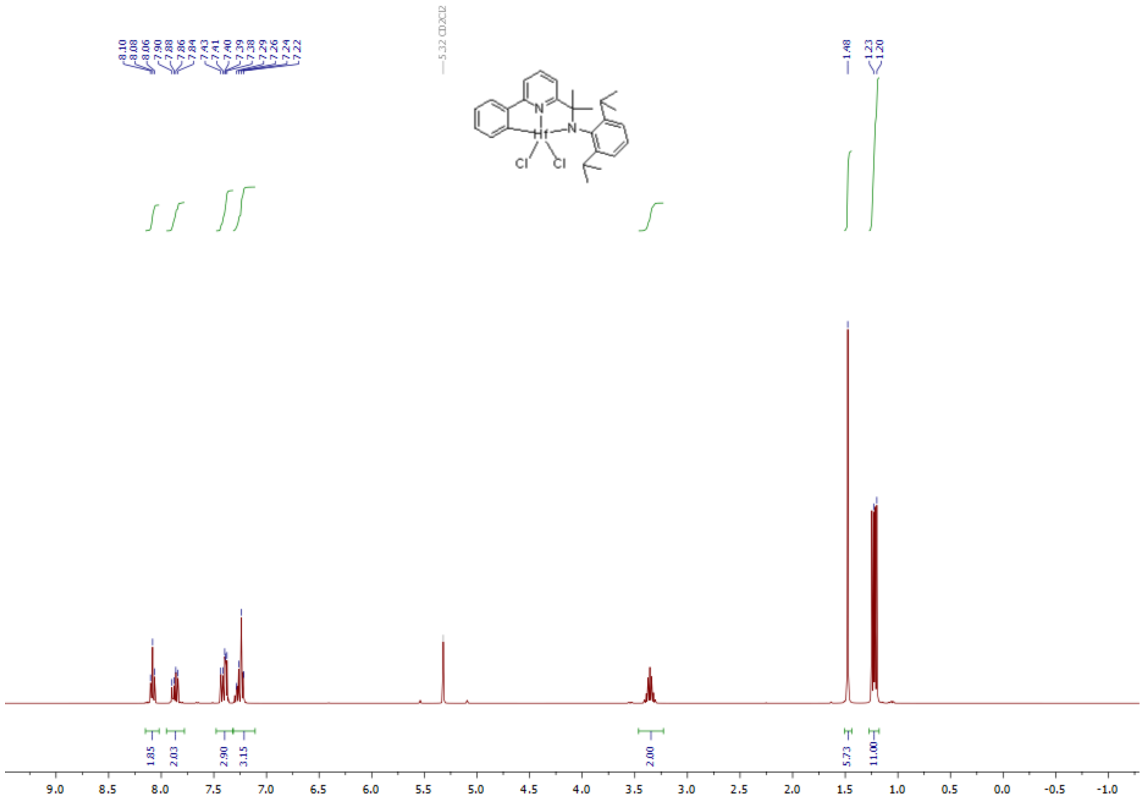

Figure S77. ^{1}H NMR spectrum of complex **2-$HfCl_2$** in CD_2Cl_2 at room temperature.

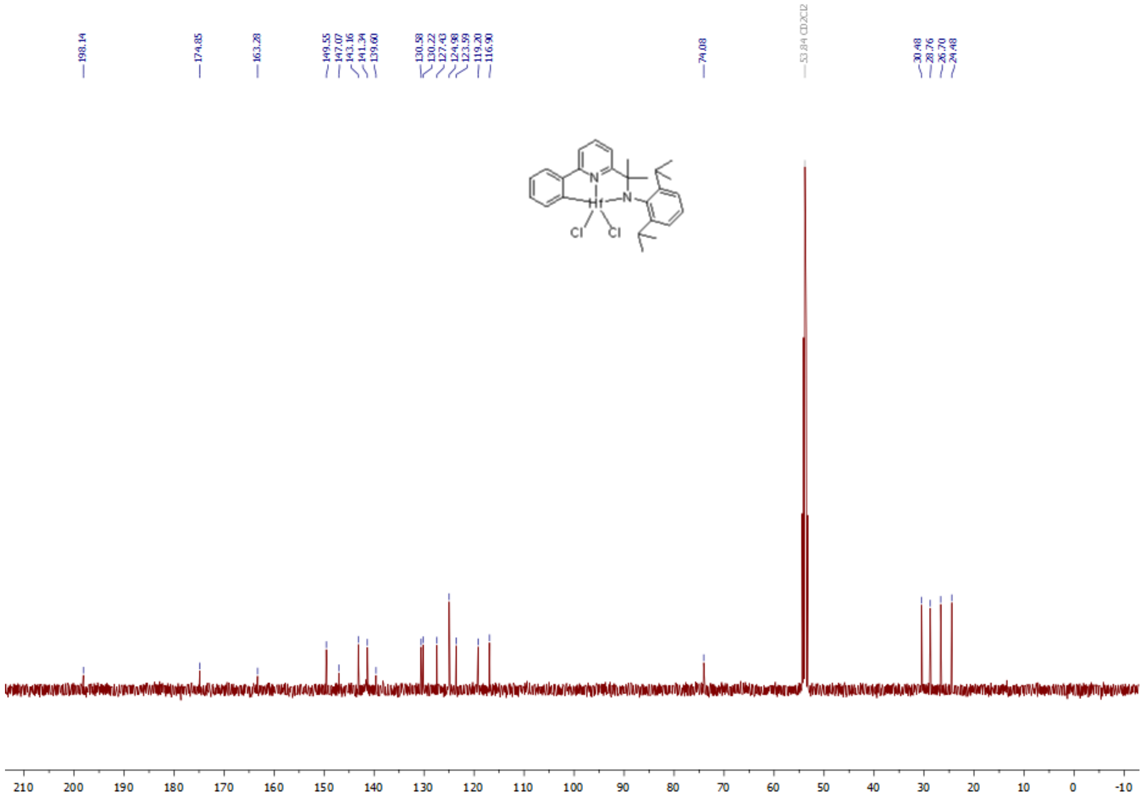

Figure S78. $^{13}C\{^{1}H\}$ NMR spectrum of complex **2-HfCl$_2$** in CD_2Cl_2 at room temperature.

2.19. Complex 2-$HfMe_2$

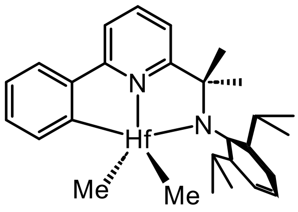

Complex **2-$HfCl_2$** (1.26 g, 2.0 mmol) was dissolved in CH_2Cl_2 (50 mL), and then solution of MeMgBr in diethyl ether (1.47 mL, 4.3 mmol, 2.9 M) was added via glass syringe at -30 °C. The resulting mixture was slowly warmed to room temperature and stirred overnight. Next, all volatiles were evaporated, and the residue was extracted with toluene filtering the obtained suspension through a short pad of Celite®. The solvents were evaporated, and the residue was recrystallized from a toluene-hexane mixture giving **2-$HfMe_2$** as a pale yellow crystalline solid (870 mg, 74%). Anal. calc. for $C_{28}H_{36}HfN_2$: C, 58.07; H, 6.27; N, 4.84. Found: C, 58.22; H, 6.35; N, 4.60.

^{1}H NMR (400 MHz, toluene-d_8) δ_H: 8.30 (1H, d, $^3J_{HH}$ = 6.8 Hz), 7.45 (1H, d, $^3J_{HH}$ = 7.8 Hz), 7.34 (1H, t, $^3J_{HH}$ = 7.1 Hz), 7.06–7.17 (5H, m), 6.97–7.01 (1H, m), 6.59 (1H, d, $^3J_{HH}$ = 7.7 Hz), 3.55 (2H, sept, $^3J_{HH}$ = 6.9 Hz, ArC*H*Me_2), 1.39 (6H, s, *Me*$_2$C(Py)NAr), 1.20 (6H, d, $^3J_{HH}$ = 6.9 Hz, ArCH*Me*$_2$), 1.17 (6H, d, $^3J_{HH}$ = 6.9 Hz, ArCH*Me*$_2$), 0.55 (6H, s, *Me*$_2$Hf).

^{13}C{^{1}H} NMR (101 MHz, toluene-d_8) δ_C: 201.7 (C_{Ar}-Hf), 175.3, 164.3, 149.5, 147.5, 141.5, 140.6, 138.9, 130.6, 128.5, 126.6, 124.5, 123.3, 117.0, 115.7, 71.5, 63.7, 32.6, 28.6, 26.1, 24.8.

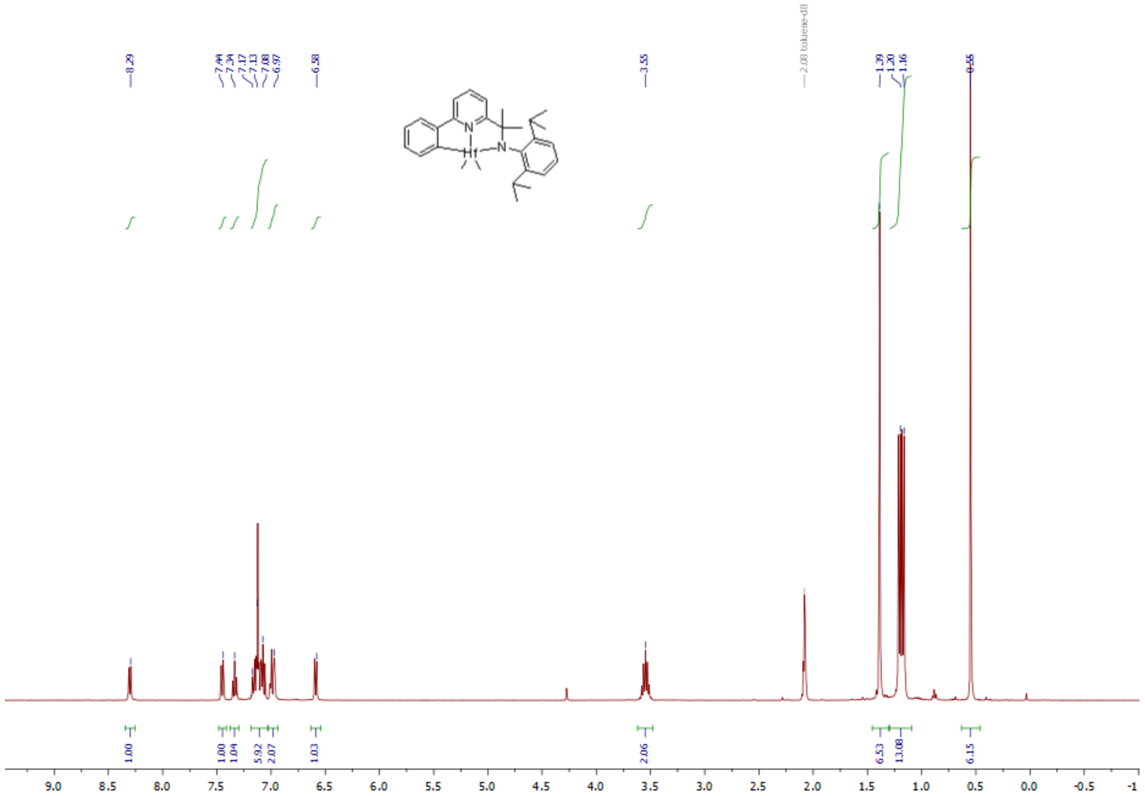

Figure S79. ^{1}H NMR spectrum of complex **2-$HfMe_2$** in toluene-d_8 at room temperature.

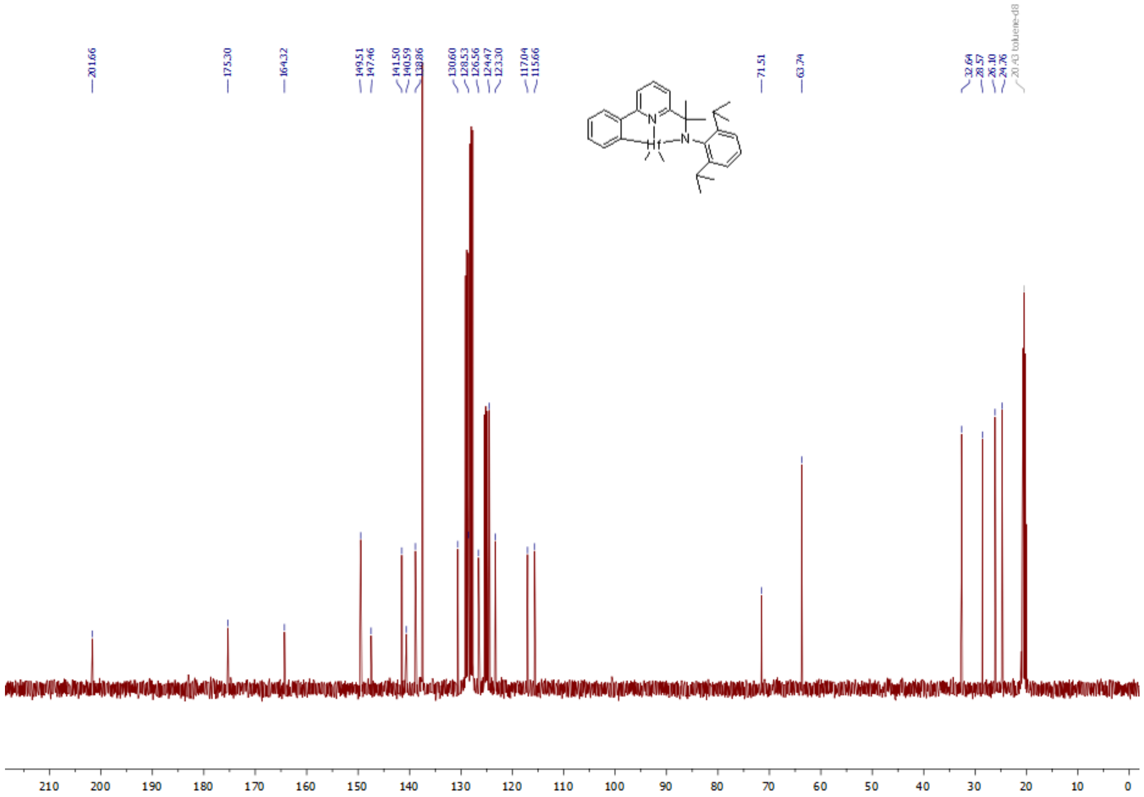

Figure S80. $^{13}C\{^1H\}$ NMR spectrum of complex **2-HfMe₂** in toluene-d_8 at room temperature.

2.20. Complex 2^{iPrCN}-$HfCl_2$

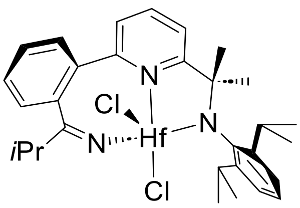

Complex **2-HfCl₂** (500 mg, 0.806 mmol) was dissolved in CH_2Cl_2 (30 mL), and then isobutyronitrile (72.7 µL, 56.0 mg, 0.806 mmol) was added in one portion via Eppendorf pipette at room temperature. The resulting yellow solution was stirred for 4 h at room temperature. Next, all volatiles were evaporated, and the residue was washed with hexane (10 mL) and dried in vacuum giving **2^{iPrCN}-$HfCl_2$** as a bright yellow solid (372 mg, 67%). Anal. calc. for $C_{30}H_{37}Cl_2HfN_3$: C, 52.30; H, 5.41; N, 6.10. Found: C, 52.64; H, 5.63; N, 5.84.

^{1}H NMR (400 MHz, CD_2Cl_2) δ_H: 8.02 (1H, t, $^3J_{HH}$ = 7.9 Hz), 7.57 (1H, d, $^3J_{HH}$ = 8.1 Hz), 7.47 (4H, m), 7.42 (1H, d, $^3J_{HH}$ = 7.7 Hz), 7.24–7.31 (3H, m), 3.65 (1H, m), 3.07 (1H, m), 1.49 (3H, s, *Me*$_2$C), 1.42 (3H, s, *Me*$_2$C), 1.37 (3H, d, $^3J_{HH}$ = 6.8 Hz), 1.27–1.29 (9H, m), 1.07 (3H, d, $^3J_{HH}$ = 6.4 Hz), 0.71 (3H, d, $^3J_{HH}$ = 7.1 Hz).

^{13}C{^{1}H} NMR (101 MHz, CD_2Cl_2) δ_C: 182.7, 171.2, 157.3, 150.0, 149.8, 147.1, 139.9, 138.4, 137.2, 132.5, 128.8, 128.6, 127.7, 127.4, 125.0, 125.0, 123.7, 120.9, 72.8, 37.2, 31.7, 30.3, 28.8, 28.6, 27.1, 26.9, 24.6, 24.4, 20.0, 18.9.

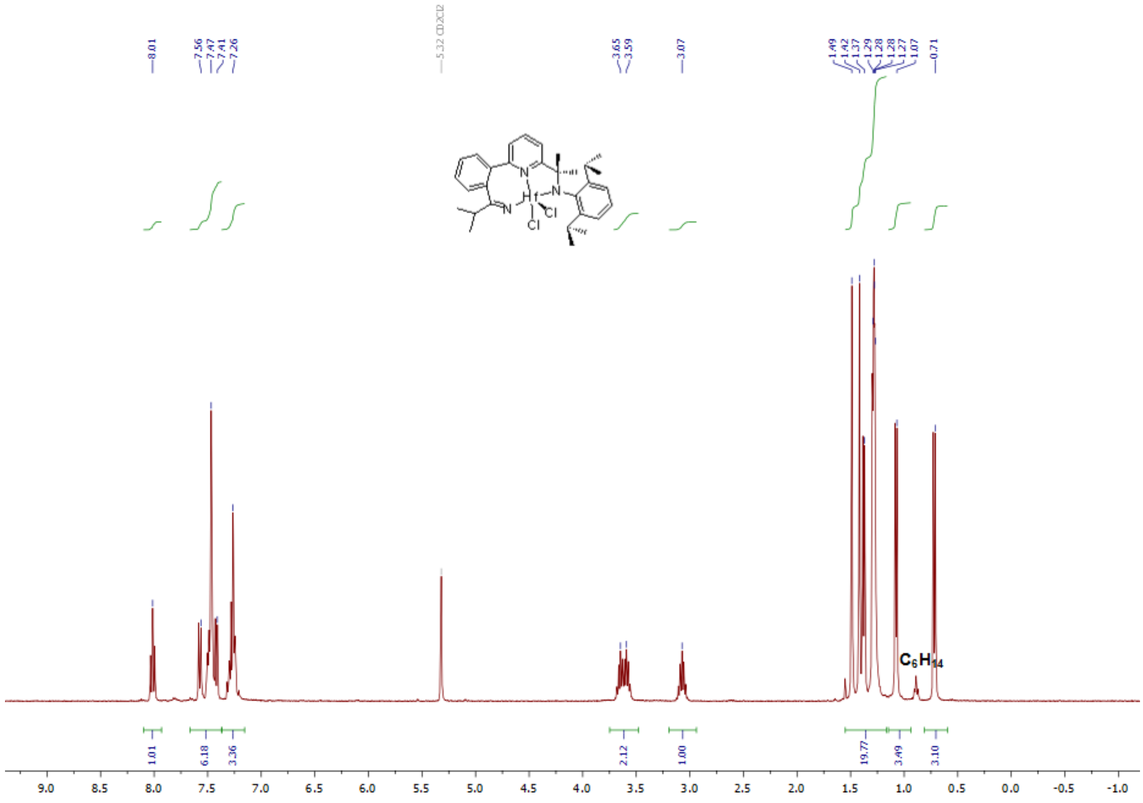

Figure S81. ^{1}H NMR spectrum of complex **2^{iPrCN}-$HfCl_2$** in CD_2Cl_2 at room temperature.

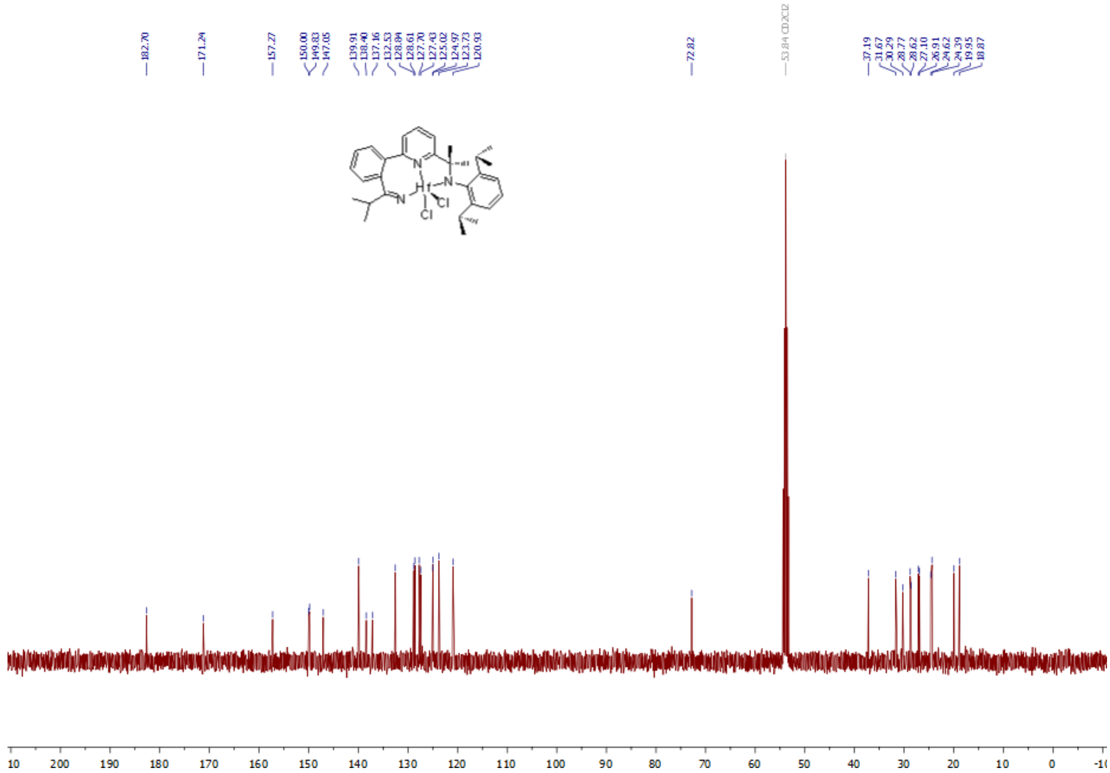

Figure S82. $^{13}C\{^{1}H\}$ NMR spectrum of complex **2^{iPrCN}-$HfCl_2$** in CD_2Cl_2 at room temperature.

2.21. Complex 2^{iPrCN}-$HfMe_2$

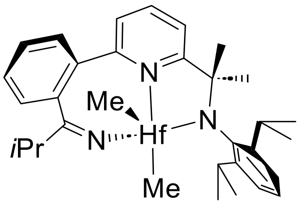

Method 1: Complex **2^{iPrCN}-$HfCl_2$** (250 mg, 0.386 mmol) was suspended in diethyl ether (30 mL), and then a solution of MeMgBr in diethyl ether (0.23 mL, 0.851 mmol, 3.7 M) was added in one portion via glass syringe at -30 °C. The resulting mixture was slowly warmed up to room temperature and stirred for 2 h. Next, all volatiles were evaporated, and the residue was extracted with hexane (100 mL) filtering the obtained suspension through a short pad of Celite®. The filtrate was concentrated in vacuum until a solid precipitated. This precipitate was filtered off while the solution was still cold, washed with cold pentane (10 mL, kept in the freezer at -30°C), and dried in vacuum to give **2^{iPrCN}-$HfMe_2$** as a pale yellowish solid (127 mg, 54%). Anal. calc. for $C_{32}H_{43}HfN_3$: C, 59.30; H, 6.69; N, 6.48. Found: C, 59.56; H, 6.91; N, 6.21.

Method 2: Complex **2-$HfMe_2$** (25.0 mg, 43 µmol) was dissolved in toluene-d_8 (0.6 mL) in J Young NMR tube, and then isobutyronitrile (3.4 µL, 2.6 mg, 43 µmol) was added to the solution in one portion via Eppendorf pipette. The obtained yellow solution was studied by NMR spectroscopy.

^1H NMR (400 MHz, toluene-d_8) δ_H: 7.20–7.27 (3H, m), 7.09 (1H, m), 6.96–7.03 (4H, m), 6.76 (2H, td, $^3J_{HH}$ = 7.9 Hz, $^4J_{HH}$ = 1.1 Hz), 4.16 (1H, m), 3.57 (1H, m), 2.85 (2H, sept, $^3J_{HH}$ = 6.8 Hz), 1.51 (3H, d, $^3J_{HH}$ = 6.7 Hz), 1.47 (3H, m), 1.46 (3H, s, *Me*$_2$C(Py)NAr), 1.34 (3H, d, $^3J_{HH}$ = 6.7 Hz), 1.28 (3H, d, $^3J_{HH}$ = 6.8 Hz), 1.25 (3H, s, *Me*$_2$C(Py)NAr), 1.16 (3H, d, $^3J_{HH}$ = 6.3 Hz), 1.66 (3H, d, $^3J_{HH}$ = 7.1 Hz), 0.07 (3H, s, *Me*Hf), -0.14 (3H, s, *Me*Hf).

$^{13}C\{^1H\}$ NMR (101 MHz, toluene-d_8) δ_C: 178.0, 171.8, 158.9, 150.5, 150.3, 148.2, 138.2, 137.9, 137.7, 131.8, 128.1, 127.1, 127.0, 125.8, 125.0, 124.8, 123.5, 118.9, 72.1, 53.8, 50.8, 37.1, 34.5, 28.9, 28.7, 28.4, 27.5, 26.9, 25.1, 24.7, 20.0, 19.2.

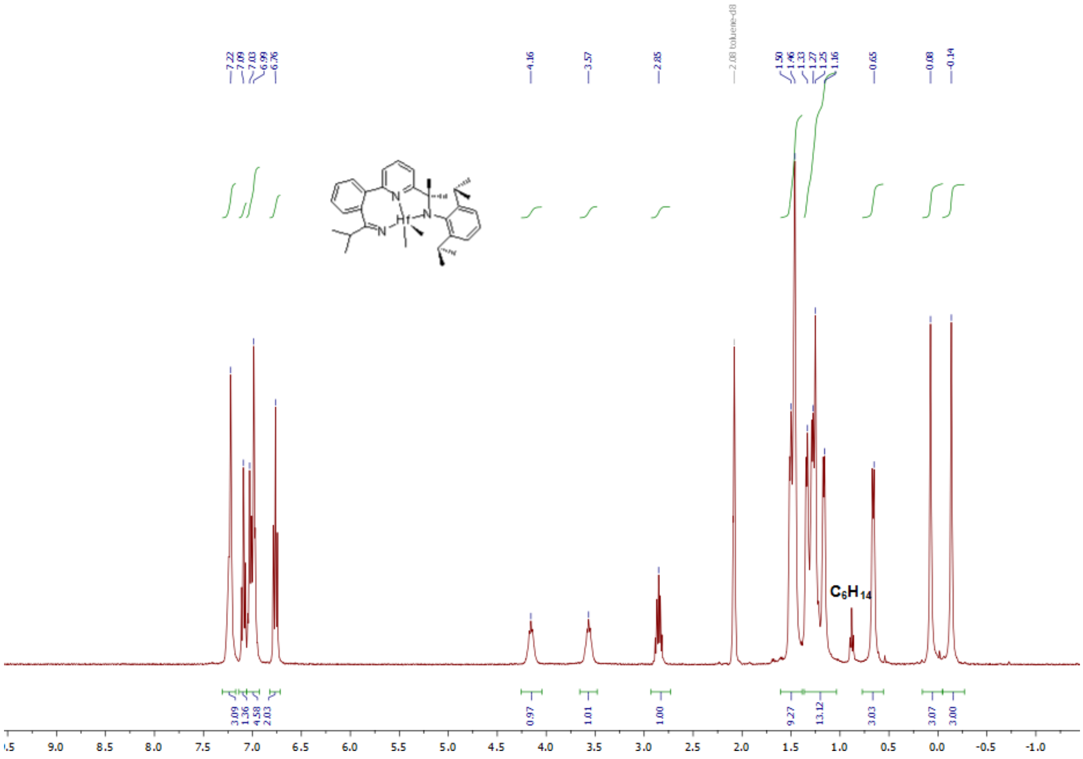

Figure S83. ^{1}H NMR spectrum of **2^{iPrCN}-HfMe$_2$** (obtained by method 1) in toluene-d_8 at room temperature.

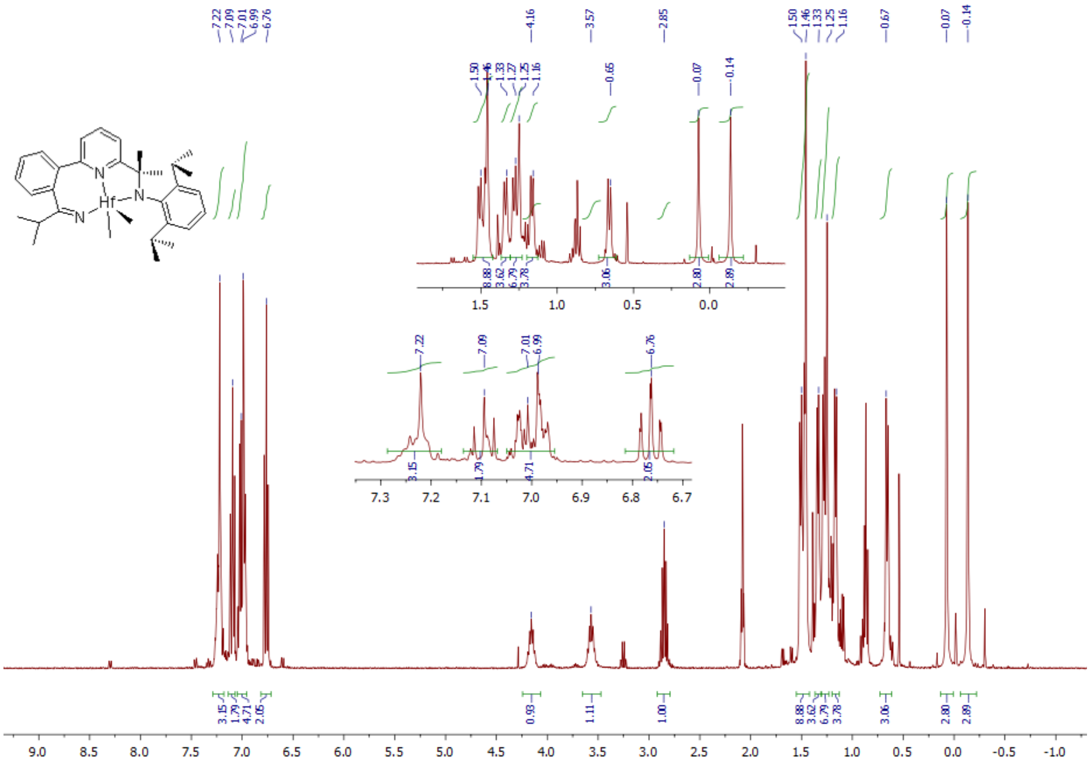

Figure S84. ^{1}H NMR spectrum of **2^{iPrCN}-HfMe$_2$** (obtained by method 2) in toluene-d_8 at room temperature.

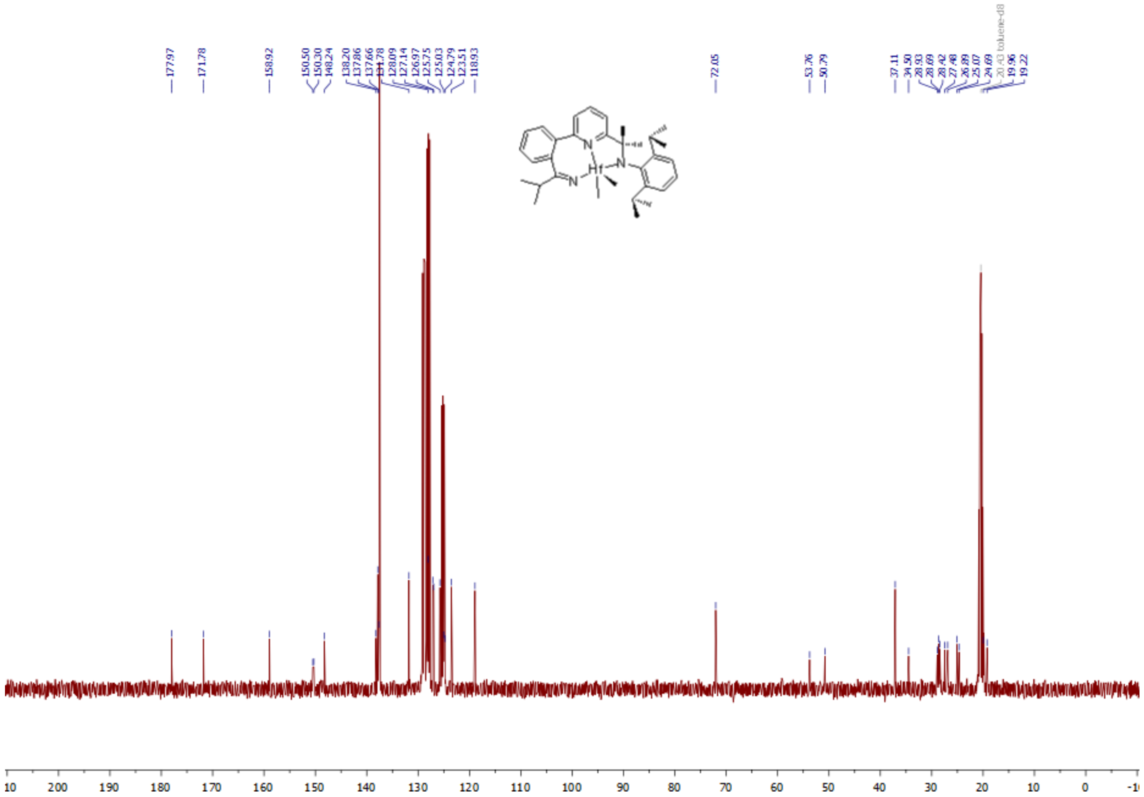

Figure S85. $^{13}C\{^{1}H\}$ NMR spectrum of **2^{iPrCN}-HfMe$_2$** (obtained by method 1) in toluene-d_8 at room temperature.

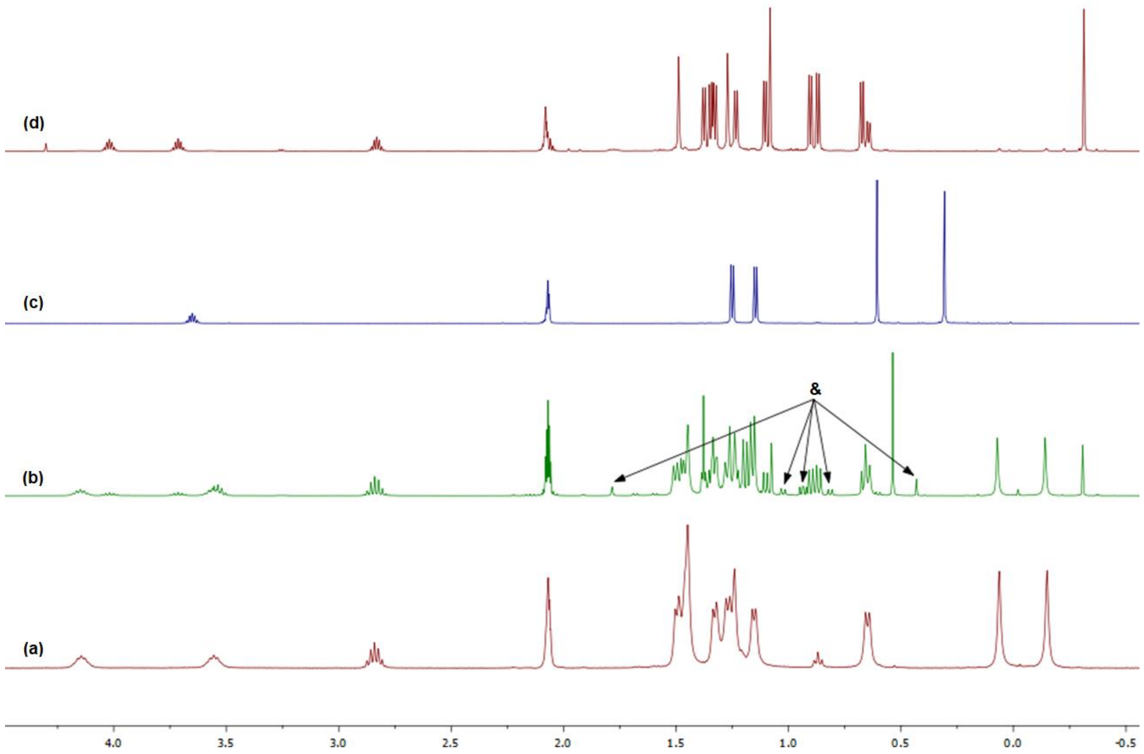

Figure S86. Fragments of ^{1}H NMR spectra of (from bottom to top): **(a) 2^{iPrCN}-HfMe$_2$** (freshly obtained), **(b) 2^{iPrCN}-HfMe$_2$** (after 8 days storage in solution at room temperature), **(c) 2-HfMe$_2$** and **(d) 2^{iPrCN}-HfMe(N=CMe*i*Pr)** in toluene-d_8 at room temperature. Symbol (&) denotes signals of complex **2-HfMe(N=CMe*i*Pr).**

2.22. Complex 2-HfMe(N=CMe*i*Pr)

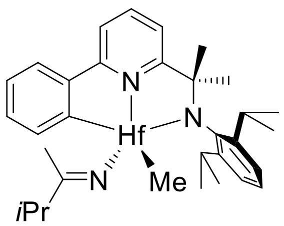

Method 1: Complex **2-HfMe$_2$** (250 mg, 0.432 mmol) was dissolved in toluene (5 mL) in an 8 mL vial, and then isobutyronitrile (39.0 µL, 30.0 mg, 0.434 mmol) was added in one portion via Eppendorf pipette. The resulting yellow solution was stirred overnight at 100 °C. Next, all volatiles were evaporated, and the residue was recrystallized from hexane (1.5 mL) at -30 °C. The precipitate was filtered off, washed with cold pentane (3 mL, kept in the freezer at -30 °C) and dried in vacuum giving **2-HfMe(N=CMe*i*Pr)** of as a yellow crystalline solid (147 mg, 53%). Additional crystallization from hexane allowed isolation of an analytically pure sample for elemental analysis. Anal. calc. for $C_{32}H_{43}HfN_3$: C, 59.30; H, 6.69; N, 6.48. Found: C, 59.44; H, 6.73; N, 6.40.

Method 2: Complex **2-HfMe$_2$** (25.0 mg, 43 µmol) was dissolved in toluene-d_8 (0.6 mL) in J Young NMR tube, and then isobutyronitrile (3.4 µL, 2.6 mg, 43 µmol) was added in one portion via Eppendorf pipette. The resulting yellow solution was heated overnight at 100 °C and then studied by NMR spectroscopy.

^{1}H NMR (600 MHz, toluene-d_8) δ_H: 6.37 (1H, m), 7.54 (1H, m), 7.34 (1H, m), 7.14–7.18 (3H, m), 7.10–7.13 (2H, m), 7.06 (1H, t, $^3J_{HH}$ = 7.7 Hz), 6.96–7.01 (1H, dd, $^3J_{HH}$ = 7.6 Hz, $^4J_{HH}$ = 1.7 Hz), 4.15 (1H, sept, $^3J_{HH}$ = 6.9 Hz), 2.87 (1H, m), 2.16 (1H, sept, $^3J_{HH}$ = 6.9 Hz), 1.79 (3H, s), 1.35 (3H, d, $^3J_{HH}$ = 6.8 Hz), 1.33 (3H, d, $^3J_{HH}$ = 6.9 Hz), 1.28 (3H, s), 1.17 (3H, s), 1.03 (3H, d, $^3J_{HH}$ = 6.9 Hz), 0.94 (6H, d, $^3J_{HH}$ = 6.9 Hz), 0.93 (3H, d, $^3J_{HH}$ = 6.9 Hz), 0.82 (3H, d, $^3J_{HH}$ = 6.9 Hz), 0.43 (3H, s, *Me*Hf).

^{13}C{^{1}H} NMR (151 MHz, toluene-d_8) δ_C: 199.6 (C_{Ar}-Hf), 189.8, 175.5, 164.6, 148.3, 148.0, 147.7, 145.6, 141.0, 140.5, 129.9, 129.2, 128.3, 124.2, 123.6, 123.3, 117.1, 115.7, 71.1, 53.1, 44.6, 37.5, 28.94, 28.91, 28.0, 27.2, 26.3, 26.2, 25.1, 23.6, 20.2, 20.1.

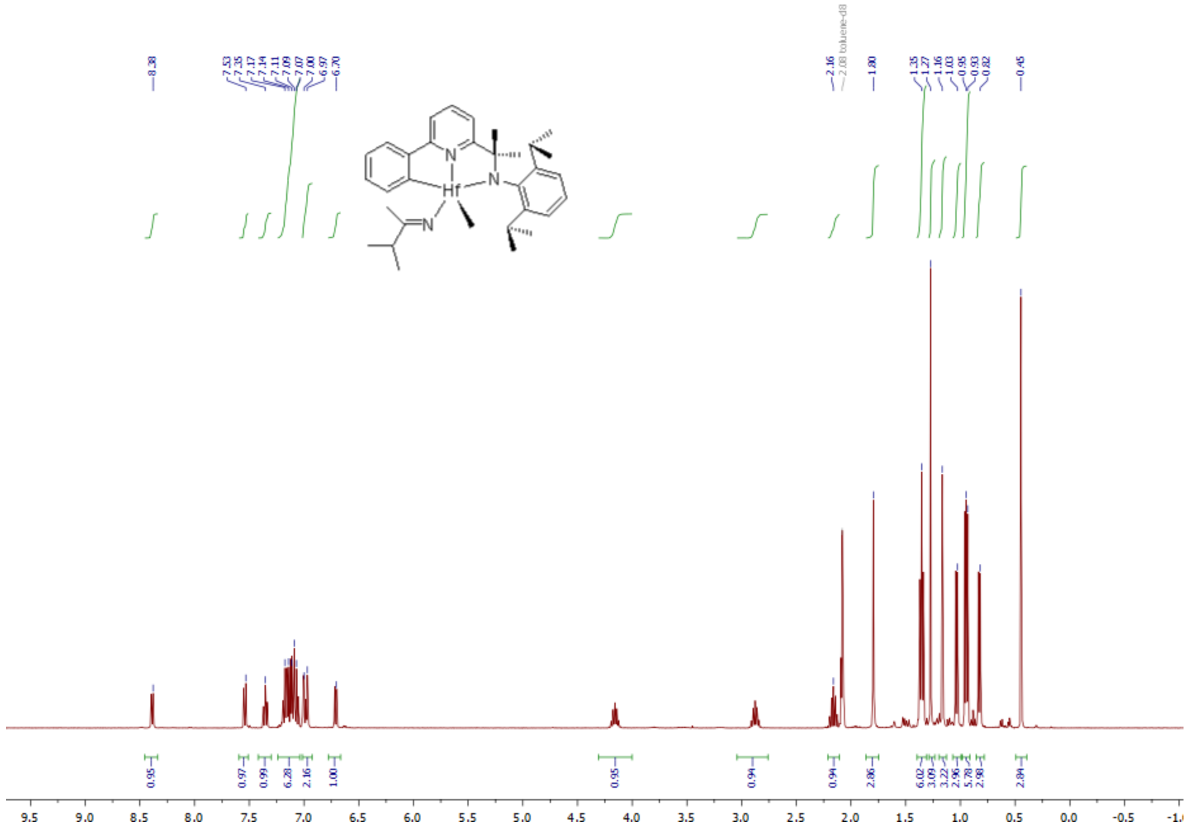

Figure S87. ^{1}H NMR of complex **2-HfMe(N=CMe*i*Pr)** (obtained by method 1) in toluene-d_8 at room temperature.

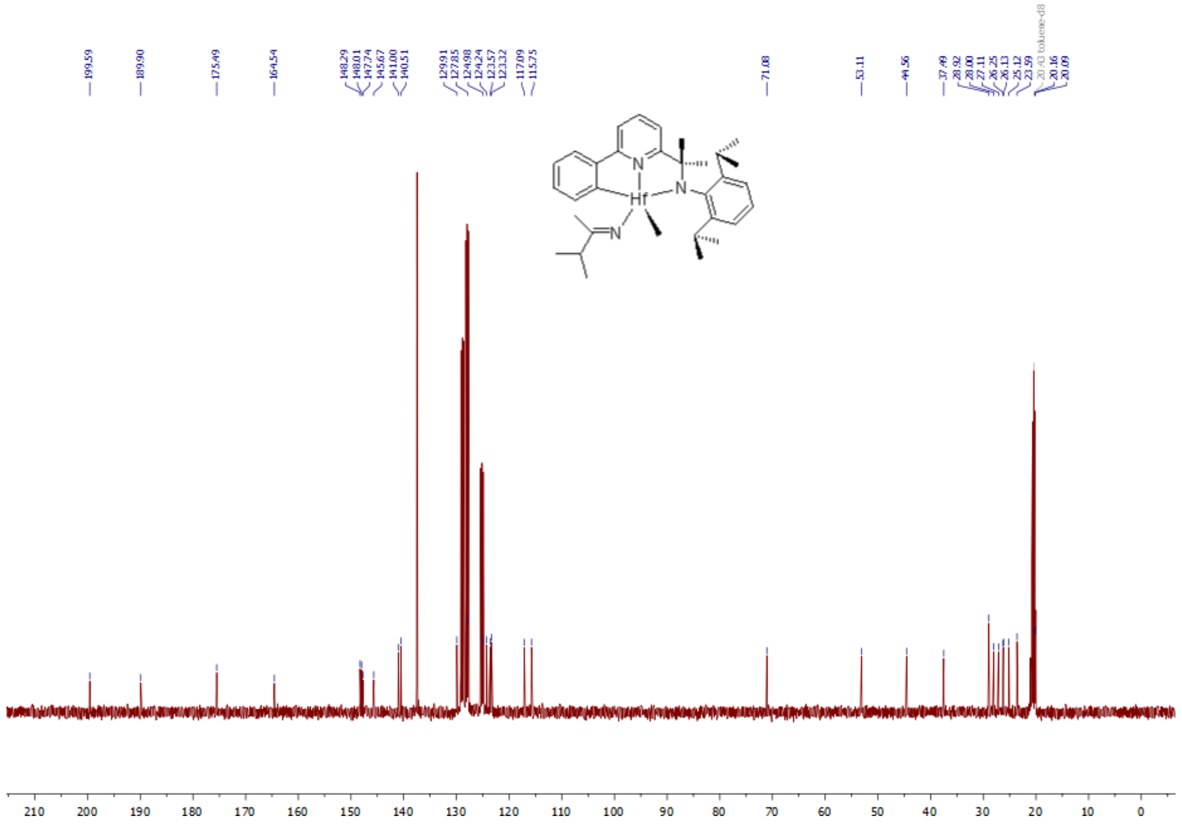

Figure S88. $^{13}C\{^{1}H\}$ NMR spectrum of complex **2-HfMe(N=CMe*i*Pr)** (obtained by method 1) in toluene-d_8 at room temperature.

2.23. Complex 2^{iPrCN}-HfMe(N=CMe*i*Pr)

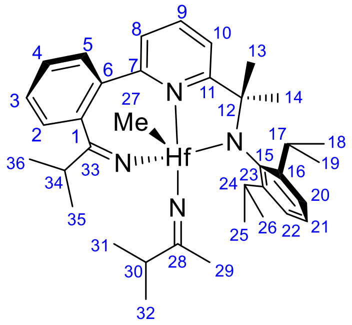

Complex **2-HfMe₂** (25.0 mg, 43 μmol) was dissolved in toluene-*d₈* (0.6 mL) in a J Young NMR tube, and then isobutyronitrile (6.8 μL, 5.2 mg, 86 μmol) was added in one portion via Eppendorf pipette. The resulting yellow solution was left overnight at room temperature and then studied by NMR spectroscopy.

^{1}H NMR (600 MHz, toluene-d_8): δ_H 7.14–7.20 (4H, m, C(20)H, C(22)H, C(9)H and C(21)H), 7.12 (2H, m, C(2)H and C(5)H), 6.99–7.04 (2H, m, C(3)H and C(4)H), 6.90 (1H, m, C(10)H), 6.85 (1H, m, C(8)H), 4.02 (1H, m, C(17)H), 3.71 (1H, m, C(24)H), 2.83 (1H, m, C(34)H), 2.07 (1H, m, C(30)H), 1.49 (3H, s, C(13)H), 1.37 (3H, d, $^3J_{HH}$ = 6.7 Hz, C(25)H), 1.34 (3H, d, $^3J_{HH}$ = 6.8 Hz, C(18)H), 1.32 (3H, d, $^3J_{HH}$ = 6.8 Hz, C(26)H), 1.27 (3H, s, C(14)H), 1.23 (3H, d, $^3J_{HH}$ = 6.7 Hz, C(19)H), 1.10 (3H, d, $^3J_{HH}$ = 6.6 Hz, C(35)H), 1.08 (3H, s, C(29)H), 0.90 (3H, d, $^3J_{HH}$ = 6.8 Hz, C(31)H), 0.86 (3H, d, $^3J_{HH}$ = 6.8 Hz, C(32)H), 0.67 (3H, d, $^3J_{HH}$ = 7.1 Hz, C(36)H), -0.31 (3H, s, C(27)H).

^{13}C{^{1}H} NMR (150 MHz, toluene-d_8) δ_C 185.0 (C-28), 175.2 (C-33), 172.2 (C-11), 158.4, 148.6, 148.5, 147.9, 144.9, 137.8, 132.0, 128.3, 126.8, 125.4, 124.9, 124.2, 124.0, 123.2, 119.7, 70.7 (C-12), 43.5 (C-30), 41.9, 37.1 (C-34), 34.0 (C-13), 28.7, 28.4 (C-14), 28.3 (C-17), 27.0, 26.5, 24.3, 24.0, 20.1, 19.9, 19.8, 19.3.

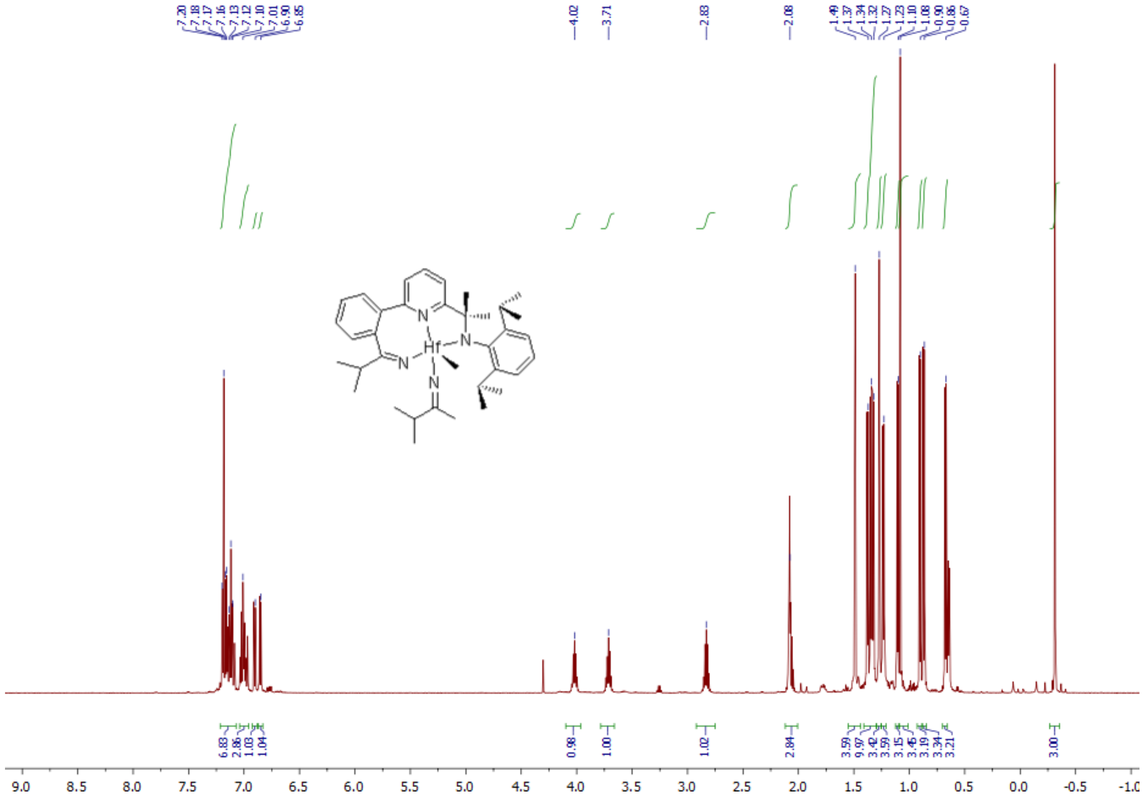

Figure S89. ^{1}H NMR spectrum of **2^{iPrCN}-HfMe(N=CMe*i*Pr)** in toluene-d_8 at room temperature.

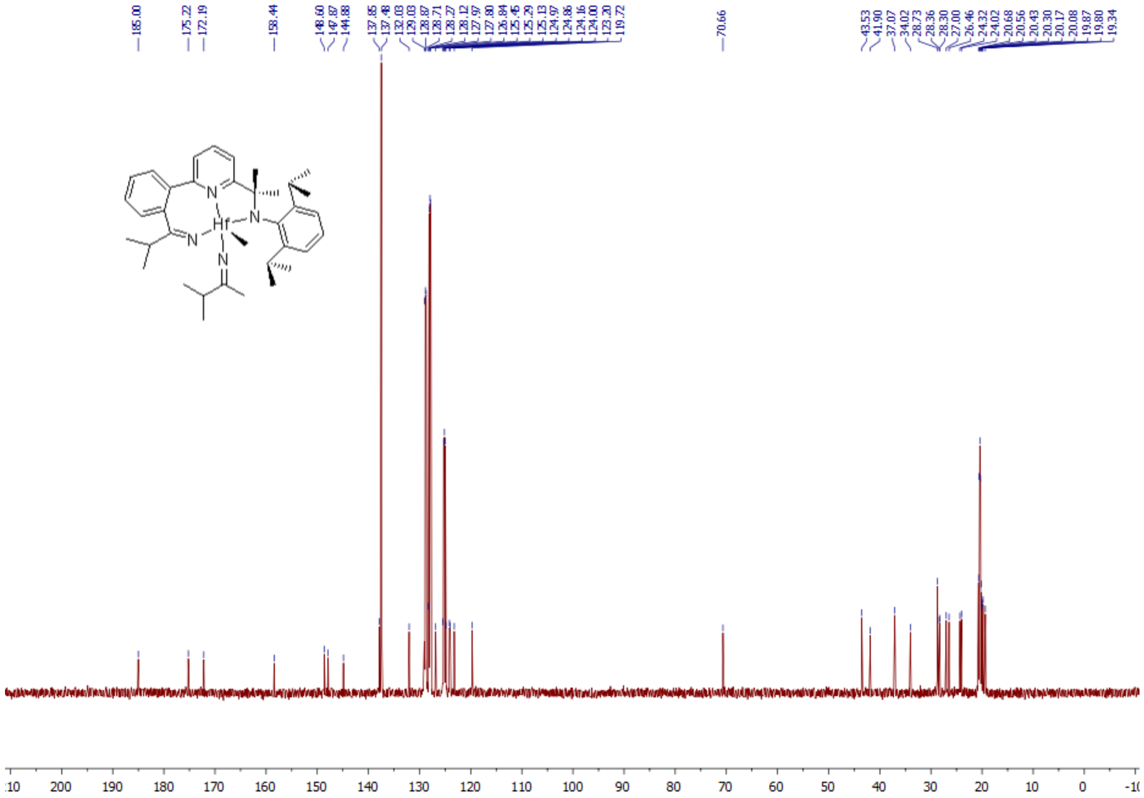

Figure S90. ^{13}C{^{1}H} NMR spectrum of complex **2^{iPrCN}-HfMe(N=CMe*i*Pr)** in toluene-d_8 at room temperature.

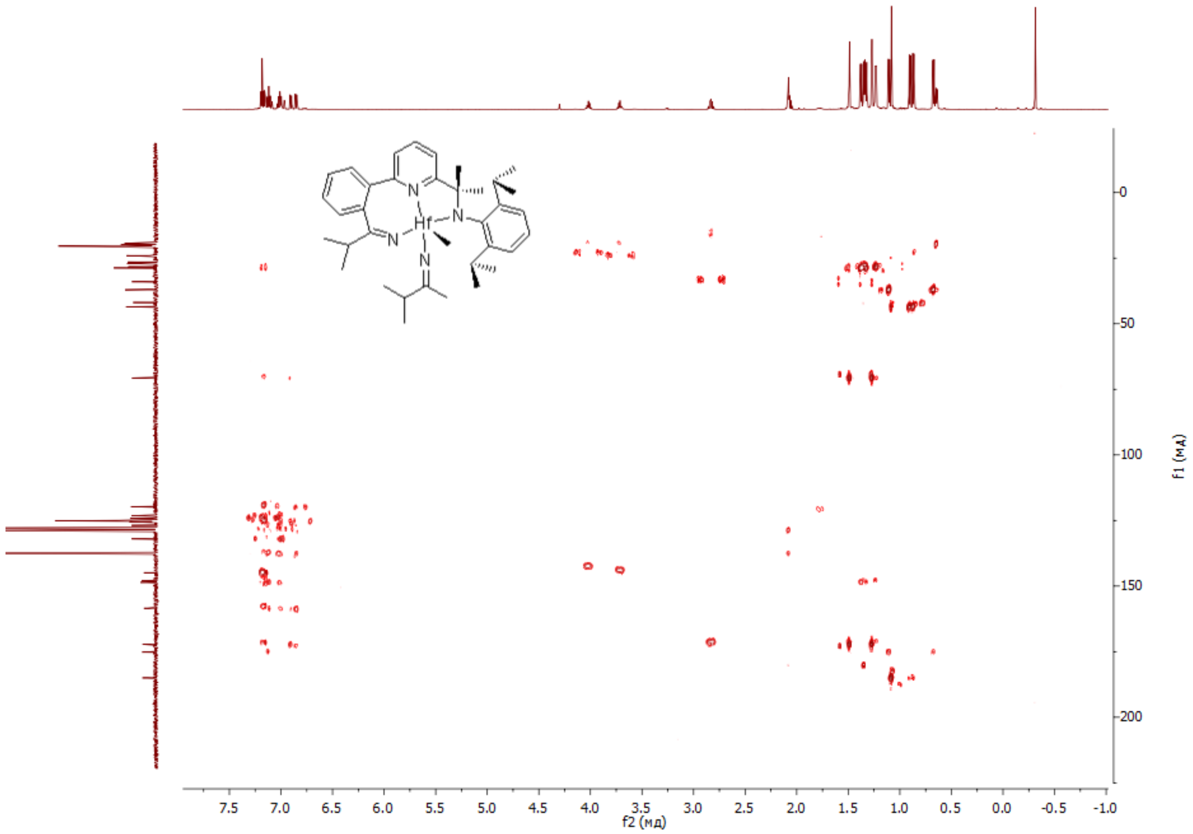

Figure S91. ^{13}C-^{1}H HMBC NMR spectrum of complex **2^{iPrCN}-HfMe(N=CMe*i*Pr)** in toluene-d_8 at room temperature.

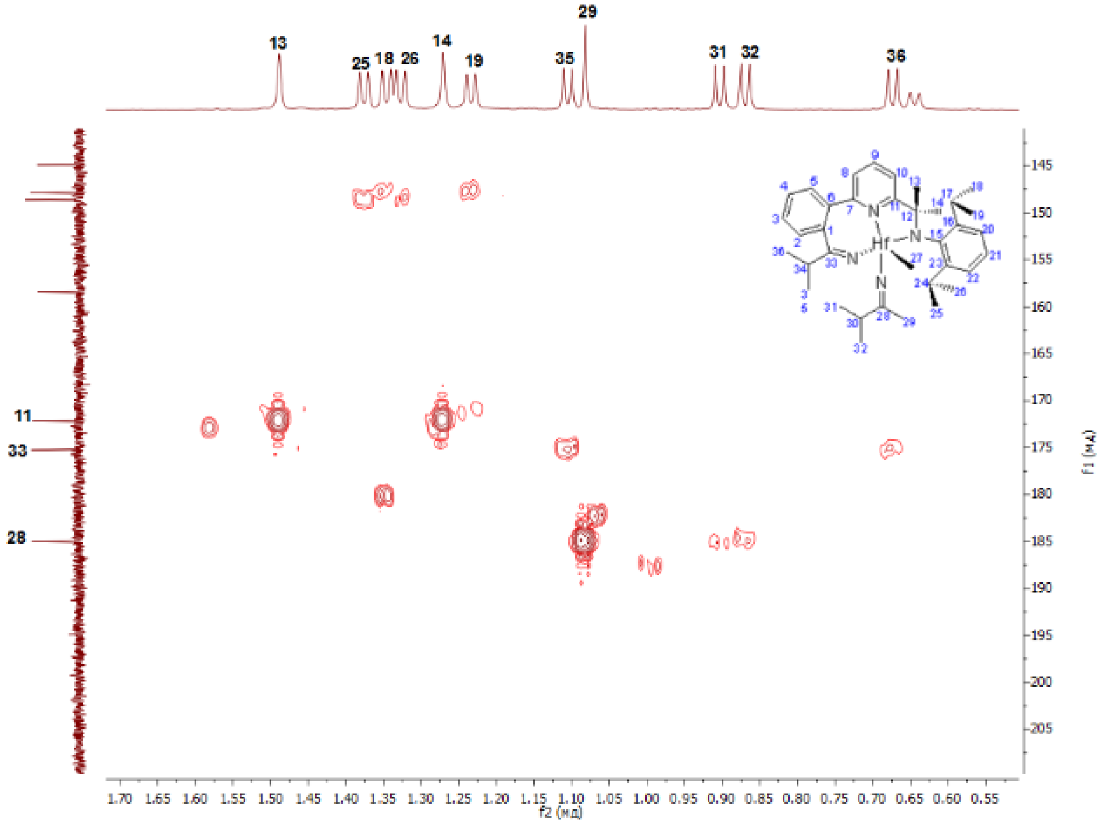

Figure S92. Fragment of ^{13}C-^{1}H HMBC NMR spectrum of complex **2^{iPrCN}-HfMe(N=CMe*i*Pr)** in toluene-d_8 at room temperature.

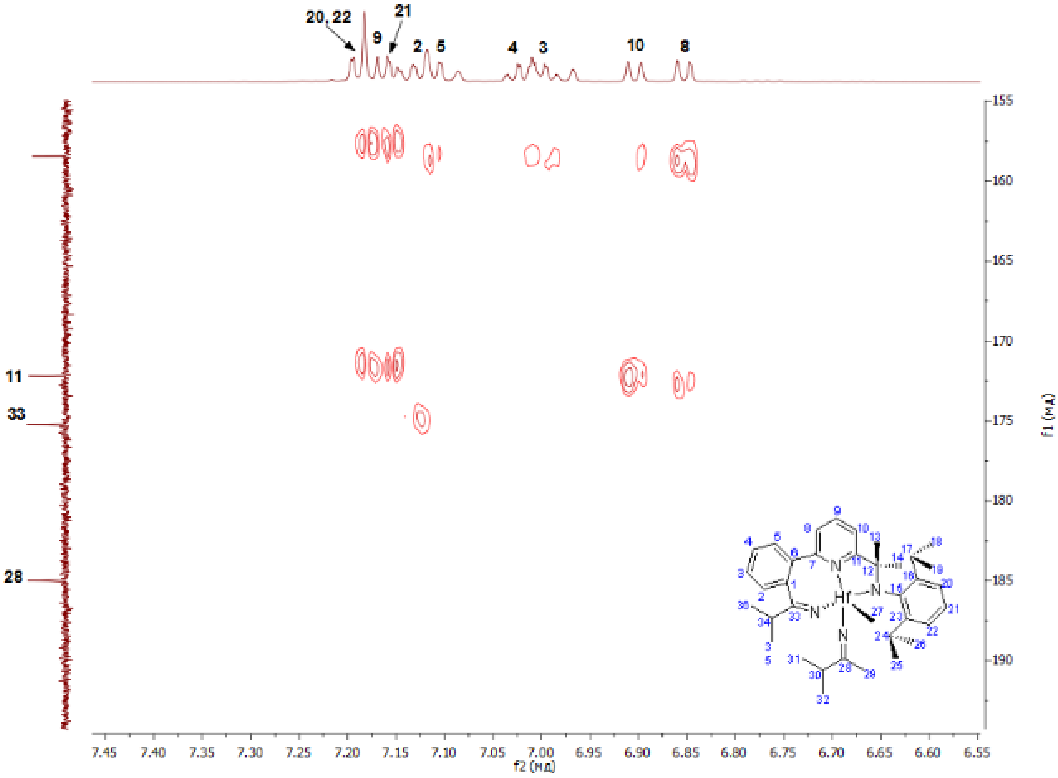

Figure S93. Fragment of ^{13}C-^{1}H HMBC NMR spectrum of complex **2^{iPrCN}-HfMe(N=CMe*i*Pr)** in toluene-d_8 at room temperature.

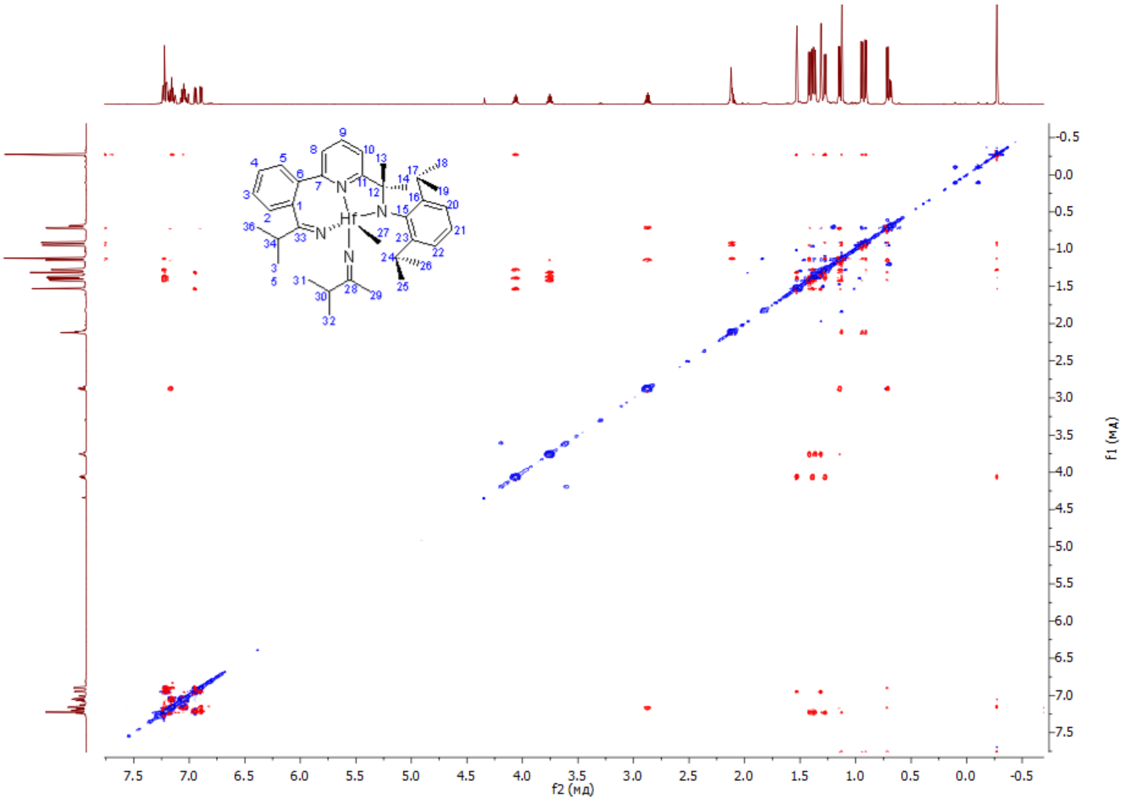

Figure S94. ^{1}H-^{1}H NOESY NMR spectrum of **2^{iPrCN}-HfMe(N=CMe*i*Pr)** in toluene-d_8 at room temperature.

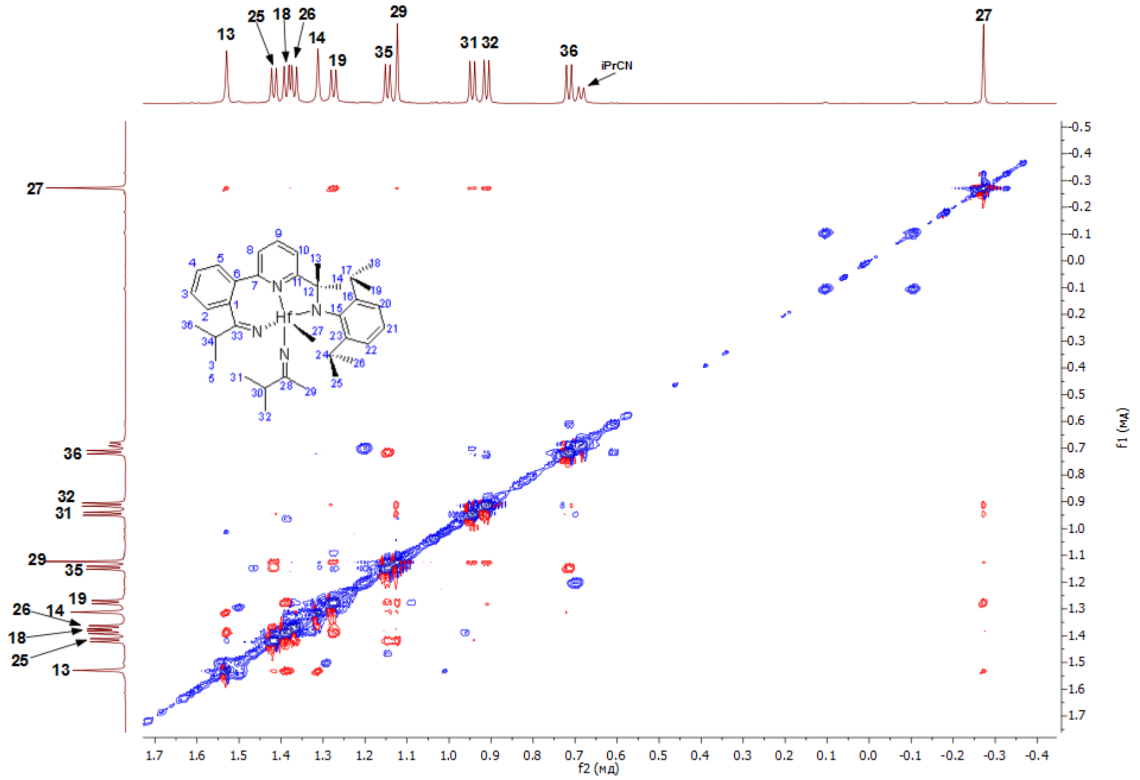

Figure S95. Fragment of ^{1}H-^{1}H NOESY NMR spectrum of **2^{iPrCN}-HfMe(N=CMe*i*Pr)** in toluene-*d$_8$* at room temperature.

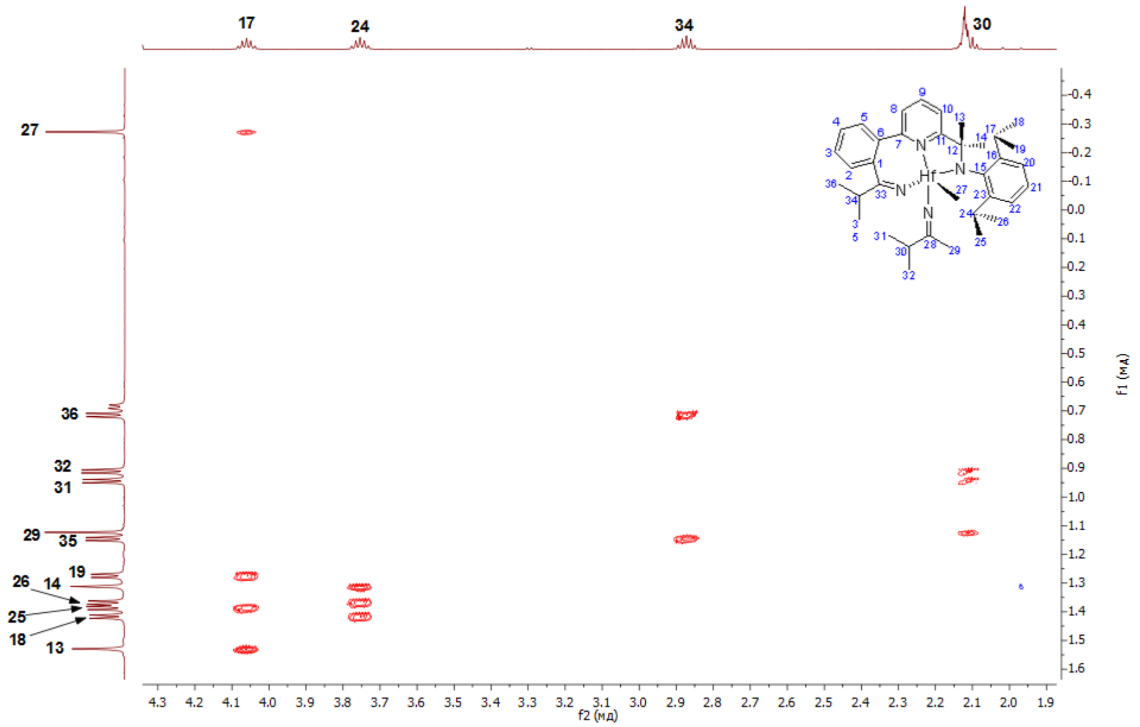

Figure S96. Fragment of ^{1}H-^{1}H NOESY NMR spectrum of **2^{iPrCN}-HfMe(N=CMe*i*Pr)** in toluene-*d$_8$* at room temperature.

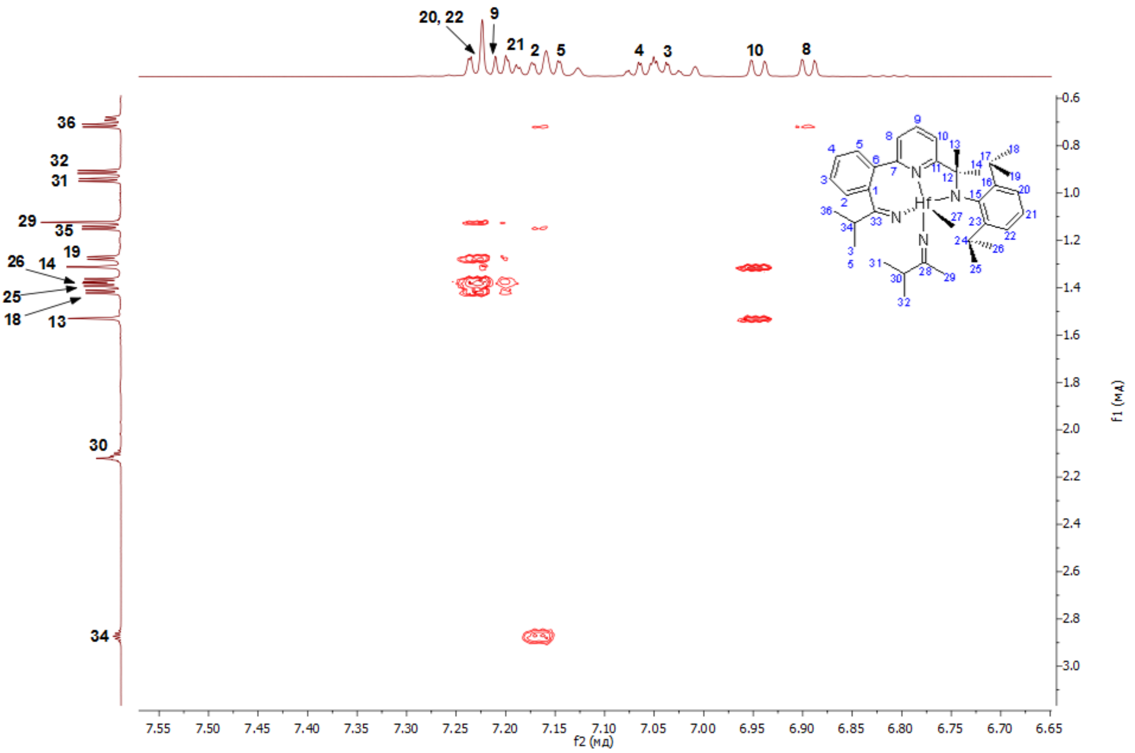

Figure S97. Fragment of ^{1}H-^{1}H NOESY NMR spectrum of 2^{iPrCN}-HfMe(N=CMe*i*Pr) in toluene-d_8 at room temperature.

2.24. Complex 2-ZrMe$_2$

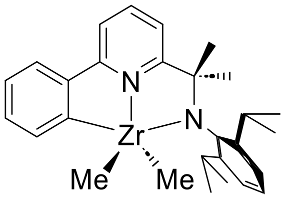

2,6-Diisopropyl-*N*-(2-(6-phenylpyridin-2-yl)propan-2-yl)aniline (2.00 g, 5.37 mmol) was dissolved in toluene (50 mL) and cooled to -40 °C, then a solution of *n*BuLi in hexanes (2.26 mL, 5.64 mmol, 2.5 M) was added dropwise via syringe at -40 °C. The obtained yellowish suspension was slowly warmed up and then stirred for 2 h at room temperature. Next, $ZrCl_4$ (1.25 g, 5.37 mmol) was added to the resulting mixture in one portion, and the obtained suspension was stirred for 2 h at 100 °C. After that, the resulting brown slurry was cooled down to room temperature, and solution of MeMgBr in diethyl ether (6.48 mL, 18.8 mmol, 2.9 M) was added in one portion via syringe. The obtained dark brown mixture was stirred overnight at room temperature, and then filtered through a short pad of Celite® remove the brown sludge. The filtrate was evaporated to dryness, and the residue was triturated with toluene (50 mL). The formed brown-yellow solid was filtered off and additionally washed with toluene until the washes were almost colourless. The combined toluene filtrates were stripped to dryness under vacuum to give a dark brown powder. This crude product was further purified by recrystallization from a toluene-hexane mixture giving **2-ZrMe$_2$** as a yellowish crystalline material (1.26 g, 48% yield). Anal. calc. for $C_{28}H_{36}N_2Zr$: C, 68.38; H, 7.38; N, 5.70. Found: C, 68.56; H, 7.55; N, 5.51.

^{1}H NMR (400 MHz, toluene-*d$_8$*) δ_H: 8.33 (1H, d, $^3J_{HH}$ = 6.7 Hz), 7.40 (1H, d, $^3J_{HH}$ = 7.8 Hz), 7.25 (1H, m), 7.06–7.16 (5H, m), 6.96–7.01 (1H, m), 6.58 (1H, d, $^3J_{HH}$ = 7.1 Hz), 3.50 (2H, sept, $^3J_{HH}$ = 6.9 Hz, ArC*H*Me$_2$), 1.40 (6H, s, *Me*$_2$C(Py)NAr), 1.16 (12H, m, ArCH*Me$_2$*), 0.74 (6H, s, *Me*$_2$Zr).

^{13}C{^{1}H} NMR (101 MHz, toluene-*d$_8$*) δ_C: 188.1 (C_{Ar}–Zr), 175.0, 164.5, 150.0, 146.2, 141.5, 138.7, 137.8, 130.2, 128.5, 127.1, 124.6, 122.7, 116.8, 115.5, 71.4, 50.4, 32.4, 28.6, 25.9, 24.9.

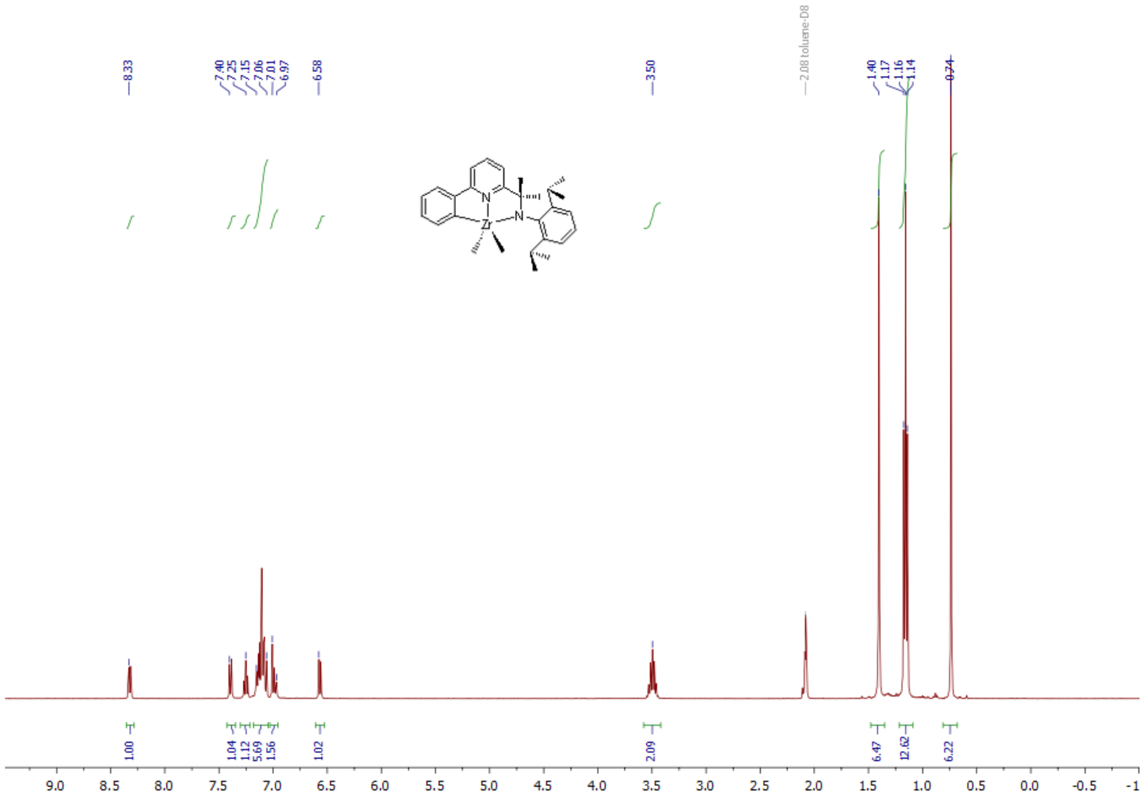

Figure S98. ^{1}H NMR spectrum of complex **2-ZrMe$_2$** in toluene-*d$_8$* at room temperature.

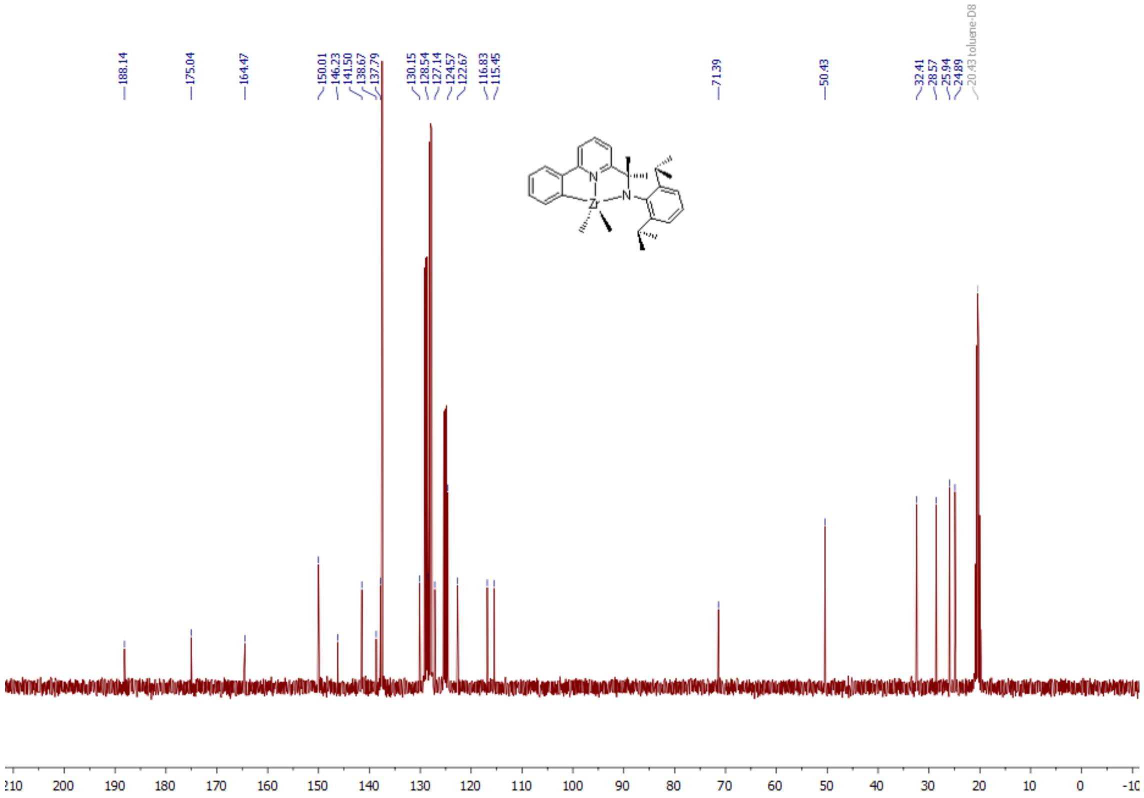

Figure S99. $^{13}C\{^{1}H\}$ NMR spectrum of complex **2-ZrMe$_2$** in toluene-*d$_8$* at room temperature.

2.25. Complex 2^{iPrCN}-ZrMe(N=CMe*i*Pr)

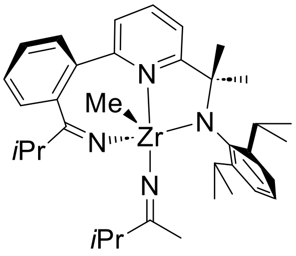

Complex **2-ZrMe$_2$** (34 mg, 69 µmol) was dissolved in toluene-d_8 (0.6 mL) in a J Young NMR tube, and then isobutyronitrile (12.3 µL, 9.5 mg, 138 µmol) was added in one portion via Eppendorf pipette. The resulting yellow solution was studied by NMR spectroscopy.

^{1}H NMR (400 MHz, toluene-d_8) δ_H: 7.14–7.19 (4H, m), 7.04–7.12 (2H, m), 6.97–7.02 (2H, m), 6.92 (1H, d, $^3J_{HH}$ = 7.9 Hz), 6.85 (1H, d, $^3J_{HH}$ = 7.5 Hz), 4.01 (1H, sept, $^3J_{HH}$ = 6.9 Hz), 3.63 (1H, sept, $^3J_{HH}$ = 6.7Hz), 2.84 (1H, sept, $^3J_{HH}$ = 6.6 Hz), 2.09 (1H, m), 1.52 (3H, s), 1.41 (3H, d, $^3J_{HH}$ = 6.7 Hz), 1.33 (3H, d, $^3J_{HH}$ = 6.9 Hz), 1.31 (3H, d, $^3J_{HH}$ = 6.9 Hz), 1.27 (3H, s, *Me*$_2$C(Py)NAr), 1.25 (3H, m), 1.24 (3H, s, *Me*$_2$C(Py)NAr), 1.09 (3H, d, $^3J_{HH}$ = 6.5 Hz), 0.88 (6H, m), 0.62 (3H, d, $^3J_{HH}$ = 7.0 Hz), -0.16 (3H, s, *Me*Zr).

^{13}C{^{1}H} NMR (101 MHz, toluene-d_8) δ_C: 183.0, 174.5, 172.2, 158.7, 148.7, 147.8, 147.7, 143.9, 137.79, 137.75, 132.1, 128.1, 126.9, 125.2, 125.1, 124.2, 124.0, 123.3, 119.3, 70.3, 42.3, 36.4, 34.1, 32.4, 28.8, 28.3, 27.2, 26.9, 26.5, 24.5, 24.0, 20.2, 20.1, 20.0, 19.4, 19.3.

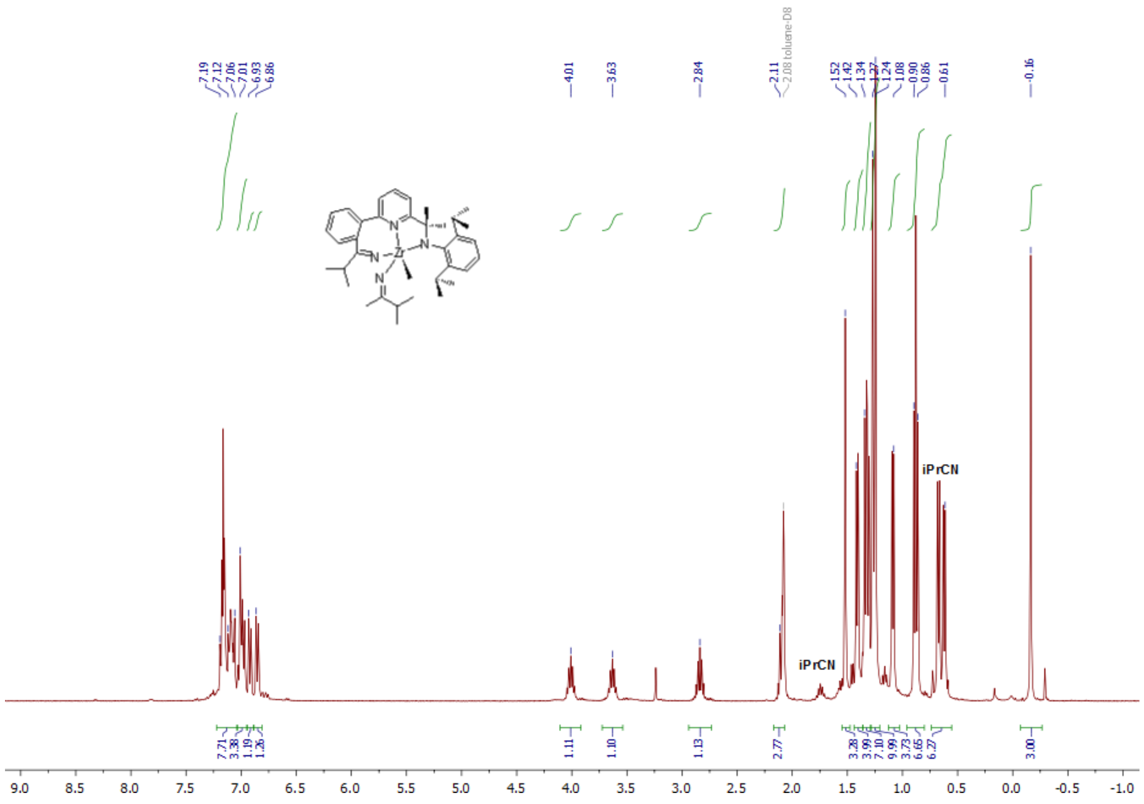

Figure S100. ^{1}H NMR spectrum of **2^{iPrCN}-ZrMe(N=CMe*i*Pr)** in toluene-d_8 at room temperature.

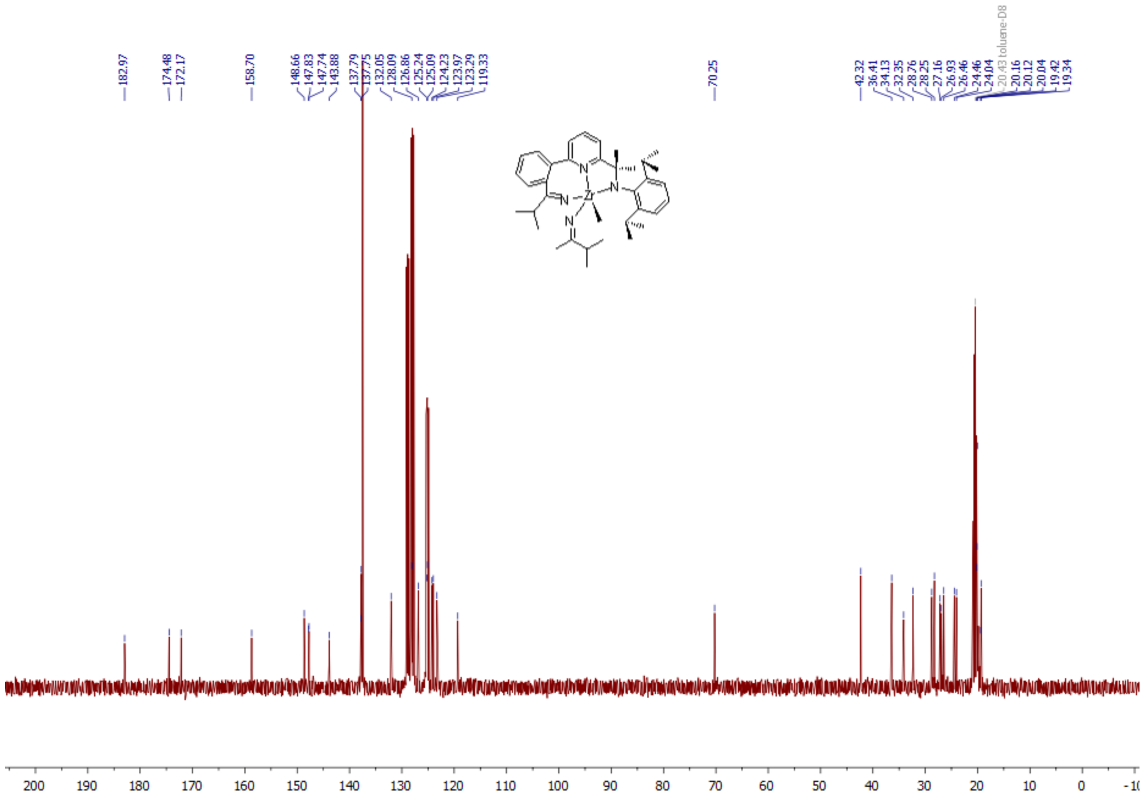

Figure S101. $^{13}C\{^{1}H\}$ NMR spectrum of complex **2^{iPrCN}-ZrMe(N=CMe*i*Pr)** in toluene-d_8 at room temperature.

2.26. Complex 2-Zr(N=CMe*i*Pr)$_2$

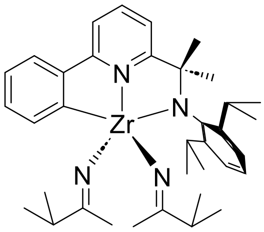

Complex **2-ZrMe$_2$** (200 mg, 0.407 mmol) was dissolved in toluene (5 mL) in an 8-mL vial, and then isobutyronitrile (73.0 µL, 56.2 mg, 0.814 mmol) was added in one portion via Eppendorf pipette. The resulting orange solution was stirred for 3 h at 100 °C. Next, all volatiles were evaporated, and the residue was recrystallized from of hexanes (1.2 mL) at -30 °C giving **2-Zr(N=CMe*i*Pr)$_2$** as an orange crystalline solid (181 mg, 71%) of. Anal. calc. for $C_{36}H_{50}N_4Zr$: C, 68.63; H, 8.00; N, 8.89. Found: C, 68.86; H, 8.21; N, 8.66.

^{1}H NMR (400 MHz, toluene-*d$_8$*) δ_H: 8.28 (1H, m), 7.58 (1H, d, $^3J_{HH}$ = 7.6 Hz), 7.18–7.24 (5H, m), 7.10 (2H, m), 6.73 (1H, m), 3.49 (2H, sept, $^3J_{HH}$ = 6.9 Hz), 2.12 (2H, sept, $^3J_{HH}$ = 6.9 Hz), 1.55 (6H, s), 1.47 (6H, s), 1.21 (6H, d, $^3J_{HH}$ = 6.9 Hz), 1.17 (6H, d, $^3J_{HH}$ = 6.9 Hz), 0.82 (6H, d, $^3J_{HH}$ = 6.9 Hz), 0.81 (6H, d, $^3J_{HH}$ = 6.9 Hz).

^{13}C{^{1}H} NMR (101 MHz, toluene-*d$_8$*) δ_C: 188.9 (C_{Ar}–Zr), 184.3, 174.9, 165.0, 147.7, 147.4, 146.8, 140.3, 140.1, 128.4, 126.8, 124.51, 124.49, 123.7, 122.9, 116.0, 115.5, 70.2, 42.28, 42.25, 32.0, 28.48, 28.46, 27.75, 27.73, 26.49, 26.46, 24.44, 24.41, 20.1, 19.9.

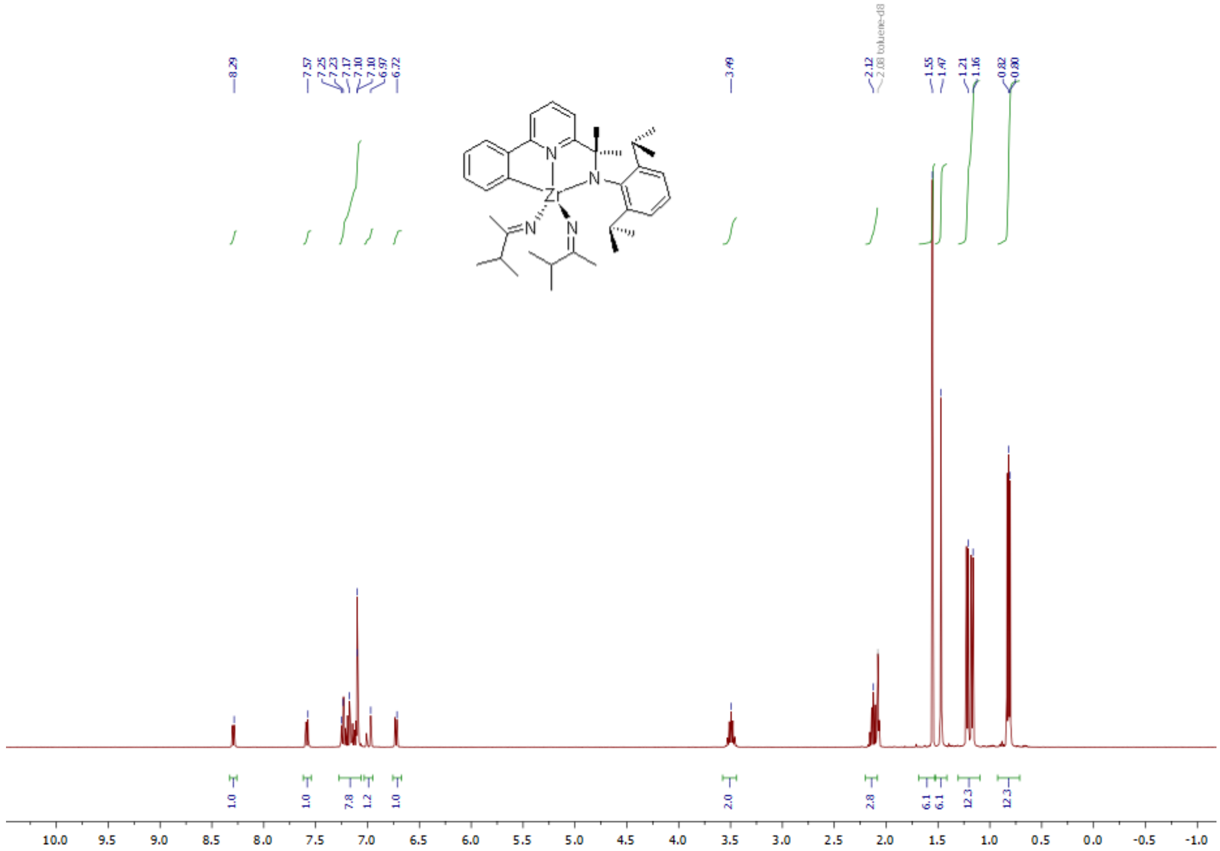

Figure S102. ^{1}H NMR spectrum of complex **2-Zr(N=CMe*i*Pr)$_2$** in toluene-*d$_8$* at room temperature.

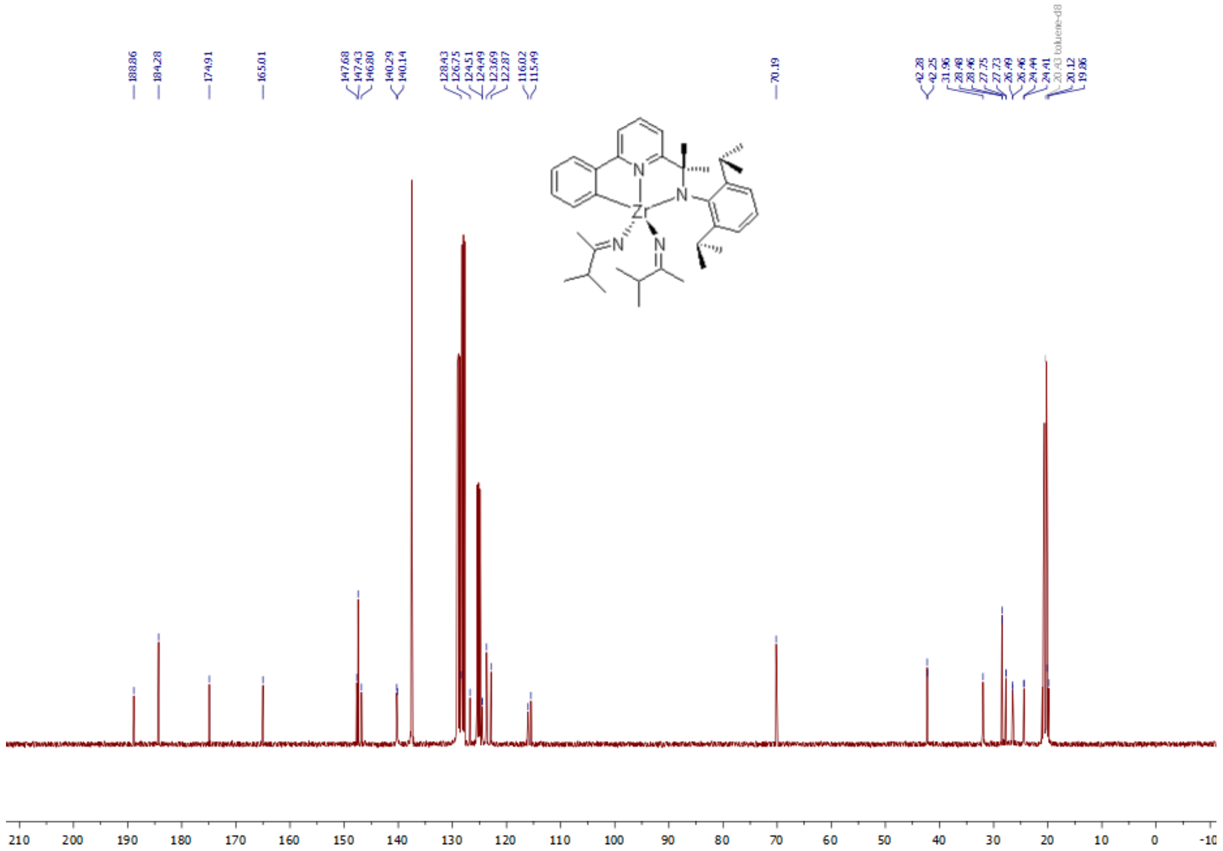

Figure S103. $^{13}C\{^1H\}$ NMR spectrum of complex **2-Zr(N=CMe*i*Pr)$_2$** in toluene-d_8 at room temperature.

2.27. Complex 3-HfCl$_2$

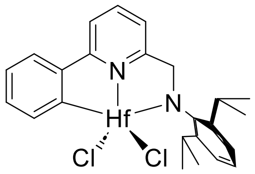

2,6-Diisopropyl-*N*-((6-phenylpyridin-2-yl)methyl)aniline (1.00 g, 2.9 mmol) was dissolved in toluene (200 mL), and then $HfCl_2Bn_2(Et_2O)$ (1.34 g, 2.7 mmol) was added in one portion. The obtained yellow solution was stirred overnight in a pressure bottle at 75 °C. Next, the obtained solution, which contained a small amount of insoluble material, was filtered through a short pad of Celite®; the Celite® was additionally washed with hot hexane (30 mL). The filtrate was evaporated to dryness, and the residue was recrystallized from a toluene-hexane mixture giving **3-HfCl$_2$** (1.04 g, 60%) as a yellowish crystalline solid. The mother liquor was evaporated to dryness, and the residue was recrystallized from toluene giving one more portion of **3-HfCl$_2$** (0.26 g, 15%; total yield: 1.30 g, 75%). Anal. calc. for $C_{24}H_{26}Cl_2HfN_2$: C, 48.70; H, 4.43; N, 4.73. Found: C, 48.85; H, 4.64; N, 4.59.

^{1}H NMR (400 MHz, CD_2Cl_2) δ_H: 8.02 (1H, m), 8.00–8.02 (1H, m), 7.82 (2H, m), 7.42 (1H, m), 7.34–7.38 (2H, m), 7.21–7.30 (3H, m), 5.36 (2H, s, C*H*$_2$(Py)NAr), 3.49 (2H, sept, $^3J_{HH}$ = 6.8 Hz, ArC*H*Me$_2$), 1.34 (6H, d, $^3J_{HH}$ = 6.8 Hz, ArCH*Me*$_2$), 1.17 (6H, d, $^3J_{HH}$ = 6.8 Hz, ArCH*Me*$_2$).

^{13}C{^{1}H} NMR (101 MHz, CD_2Cl_2) δ_C: 197.1 (C_{Ar}–Hf), 166.8, 164.9, 147.8, 147.0, 142.8, 142.7, 139.3, 130.6, 129.9, 127.5, 124.8, 123.6, 119.2, 116.7, 68.6 (*C*H$_2$(Py)NAr), 28.2, 27.7, 23.8.

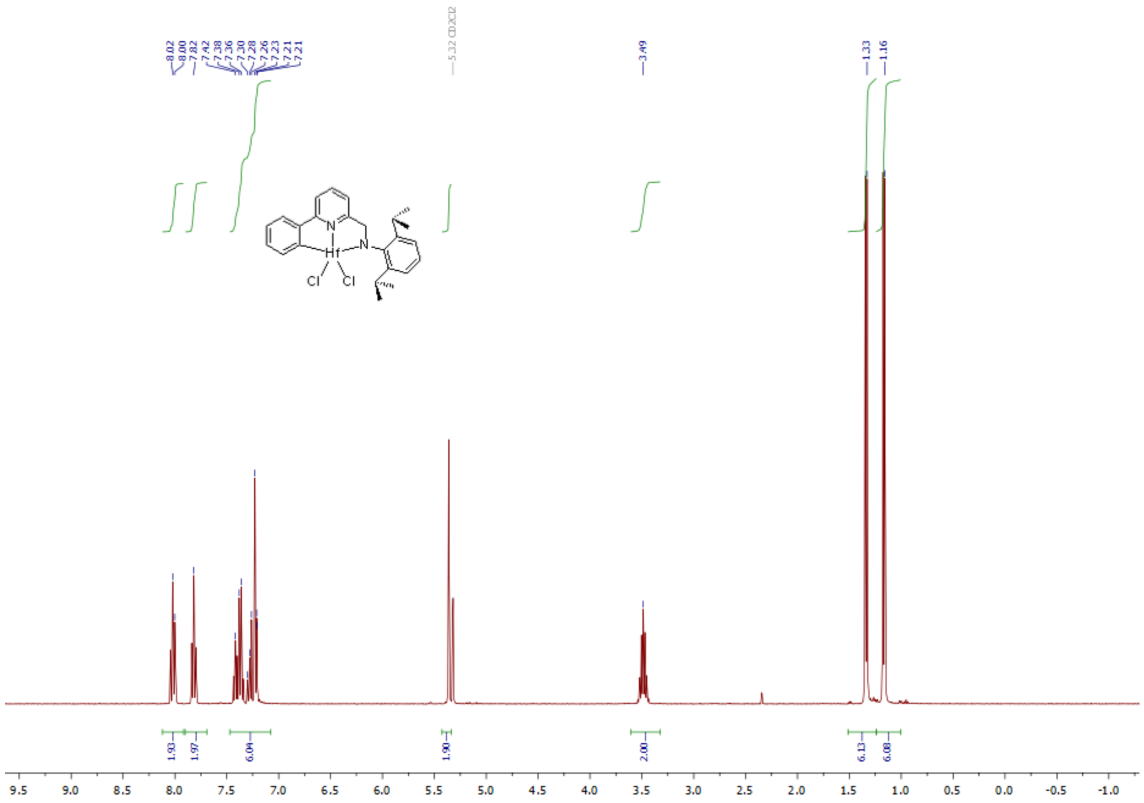

Figure S104. ^{1}H NMR spectrum of complex **3-HfCl$_2$** in CD_2Cl_2 at room temperature.

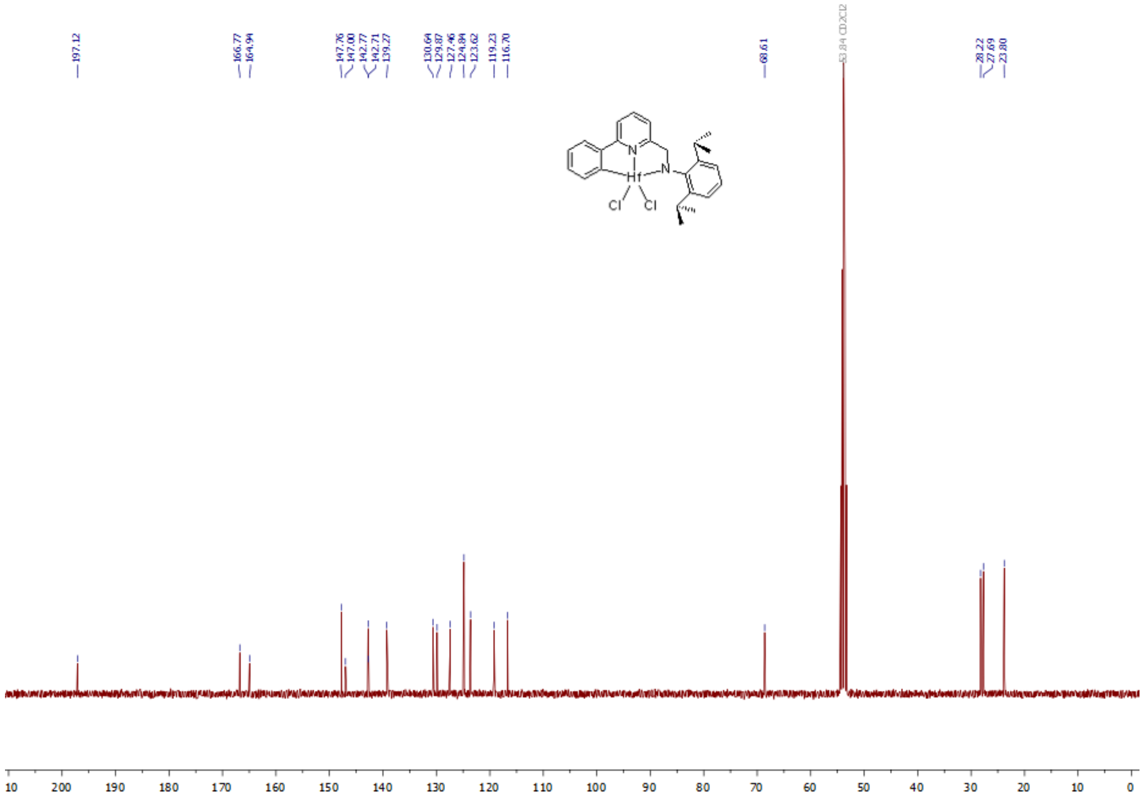

Figure S105. $^{13}C\{^{1}H\}$ NMR spectrum of complex **3-HfCl$_2$** in CD_2Cl_2 at room temperature.

2.28. Complex 3-HfMe$_2$

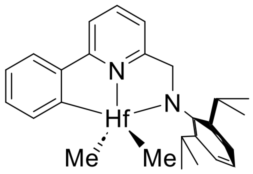

1.60 mL of 2.9 M MeMgBr (4.64 mmol) in diethyl ether was added via glass syringe to a solution of 1.30 g (2.2 mmol) of **Hf-HfCl$_2$** in 50 mL of dichloromethane at -30 °C. The resulting mixture was stirred overnight at room temperature and then evaporated to dryness. The crude product was extracted from the residue using hot hexanes. The obtained suspension was filtered through a short pad of Celite®. The filtrate was evaporated to dryness, and the residue was recrystallized from a toluene-hexanes mixture giving 800 mg (67%) of **3-HfMe$_2$** as a pale yellow crystalline solid. Anal. calc. for $C_{26}H_{32}HfN_2$: C, 56.67; H, 5.85; N, 5.08. Found: C, 56.82; H, 6.03; N, 4.89.

^{1}H NMR (400 MHz, toluene-d_8) δ_H: 8.27 (1H, d, $^3J_{HH}$ = 6.8 Hz), 7.38 (1H, d, $^3J_{HH}$ = 7.8 Hz), 7.33 (1H, t, $^3J_{HH}$ = 7.1 Hz), 7.19 (3H, m), 7.14 (1H, m), 6.89–6.97 (2H, m), 6.39 (1H, d, $^3J_{HH}$ = 7.2 Hz), 5.07 (2H, s, C*H$_2$*(Py)NAr), 3.71 (2H, sept., $^3J_{HH}$ = 6.8 Hz, ArC*H*Me$_2$), 1.33 (d, $^3J_{HH}$ = 6.8 Hz, 6H, ArCH*Me$_2$*), 1.19 (d, $^3J_{HH}$ = 6.8 Hz, 6H, ArCH*Me$_2$*), 0.58 (s, 6H, *Me$_2$*Hf).

^{13}C{^{1}H} NMR (101 MHz, toluene-d_8) δ_C: 202.0 (C_{Ar}–Hf), 166.2, 164.7, 147.7, 147.3, 144.8, 140.7, 138.6, 130.7, 128.5, 126.6, 124.5, 123.1, 117.7, 115.2, 68.4, 64.4, 28.4, 28.0, 24.0.

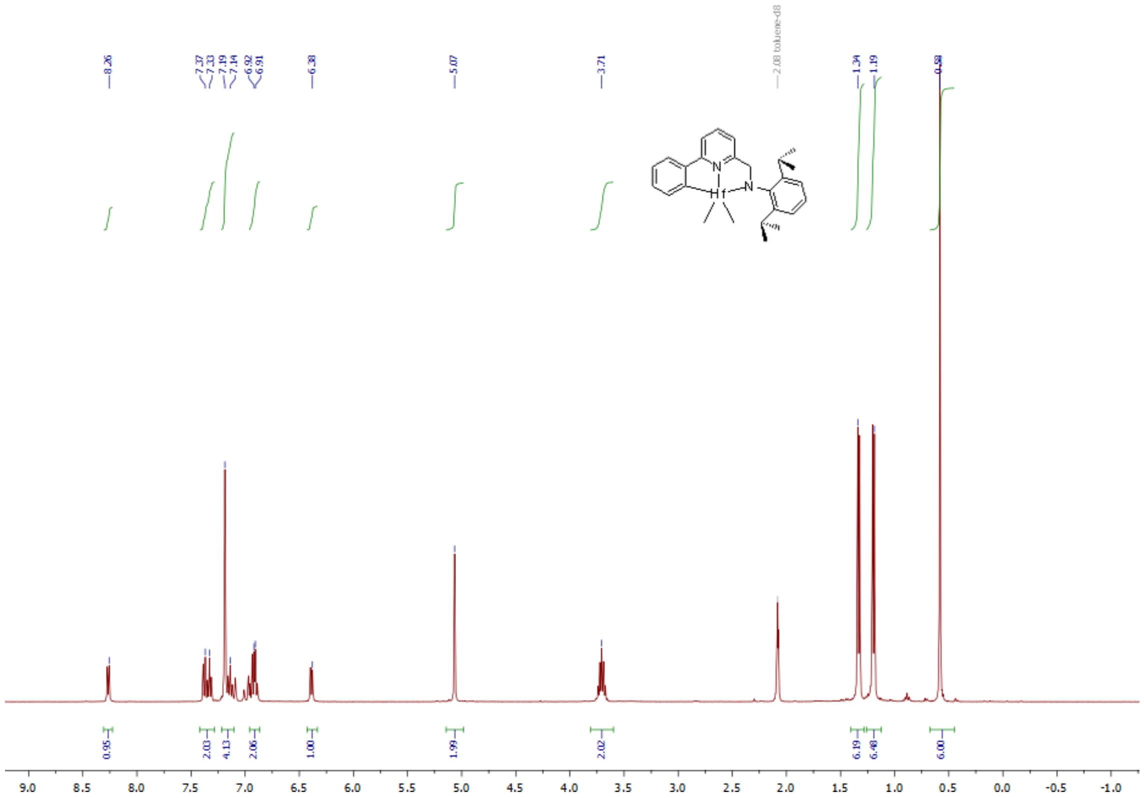

Figure S106. ^{1}H NMR spectrum of complex **3-HfMe$_2$** in toluene-d_8 at room temperature.

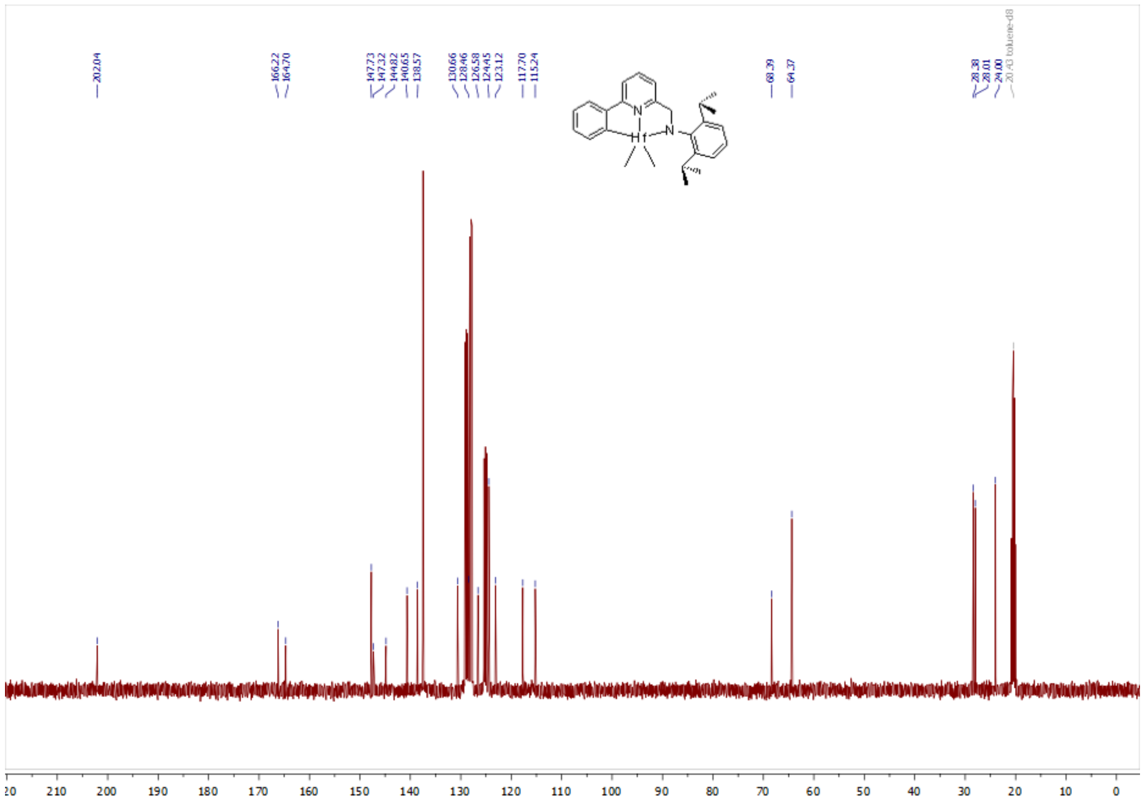

Figure S107. $^{13}C\{^1H\}$ NMR spectrum of complex **3-HfMe$_2$** in toluene-d_8 at room temperature.

2.29. Complex 3^{iPrCN}-HfCl$_2$

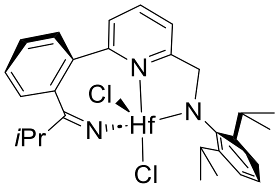

Complex **3-HfCl$_2$** (500 mg, 0.810 mmol) was dissolved in CH_2Cl_2 (30 mL), and then isobutyronitrile (72.7 µL, 56.0 mg, 0.81 mmol) was added in one portion via Eppendorf pipette at room temperature. The resulting bright yellow solution was stirred at room temperature for 4 h. Next, all volatiles were evaporated and the residue was washed with hexane (30 mL) and dried in vacuum giving **3^{iPrCN}-HfCl$_2$** as a bright yellow solid (400 mg, 72%) of. Anal. calc for $C_{28}H_{33}Cl_2HfN_3$: C, 50.88; H, 5.03; N, 6.36. Found: C, 51.16; H, 5.27; N, 6.20.

^{1}H NMR (400 MHz, CD_2Cl_2) δ_H: 7.98 (1H, t, $^3J_{HH}$ = 7.8 Hz), 7.47 (3H, m), 7.43 (2H, m), 7.21 (4H, m), 5.13 (1H, d, $^2J_{HH}$ = 20.4 Hz, C*H$_2$*(Py)NAr), 5.08 (1H, d, $^2J_{HH}$ = 20.4 Hz, C*H$_2$*(Py)NAr), 3.58 (1H, sept, $^3J_{HH}$ = 6.9 Hz, ArC*H*Me$_2$), 3.50 (1H, sept, $^3J_{HH}$ = 6.8 Hz, ArC*H*Me$_2$), 3.08 (1H, m, Me$_2$C*H*C(Ar)=N), 1.38 (3H, d, $^3J_{HH}$ = 6.8 Hz), 1.31 (3H, d, $^3J_{HH}$ = 6.8 Hz), 1.25 (6H, d, $^3J_{HH}$ = 6.9 Hz), 1.08 (3H, d, $^3J_{HH}$ = 6.3 Hz), 0.75 (3H, d, $^3J_{HH}$ = 7.10 Hz).

^{13}C{^{1}H} NMR (101 MHz, CD_2Cl_2) δ_C: 184.2, 162.1, 158.0, 146.8, 146.7, 145.9, 145.6, 139.5, 136.8, 132.4, 129.0, 128.8, 127.3, 126.7, 124.6, 124.3, 124.0, 120.6, 66.9, 37.3, 28.3, 28.1, 26.0, 25.6, 25.4, 24.8, 19.9, 18.6.

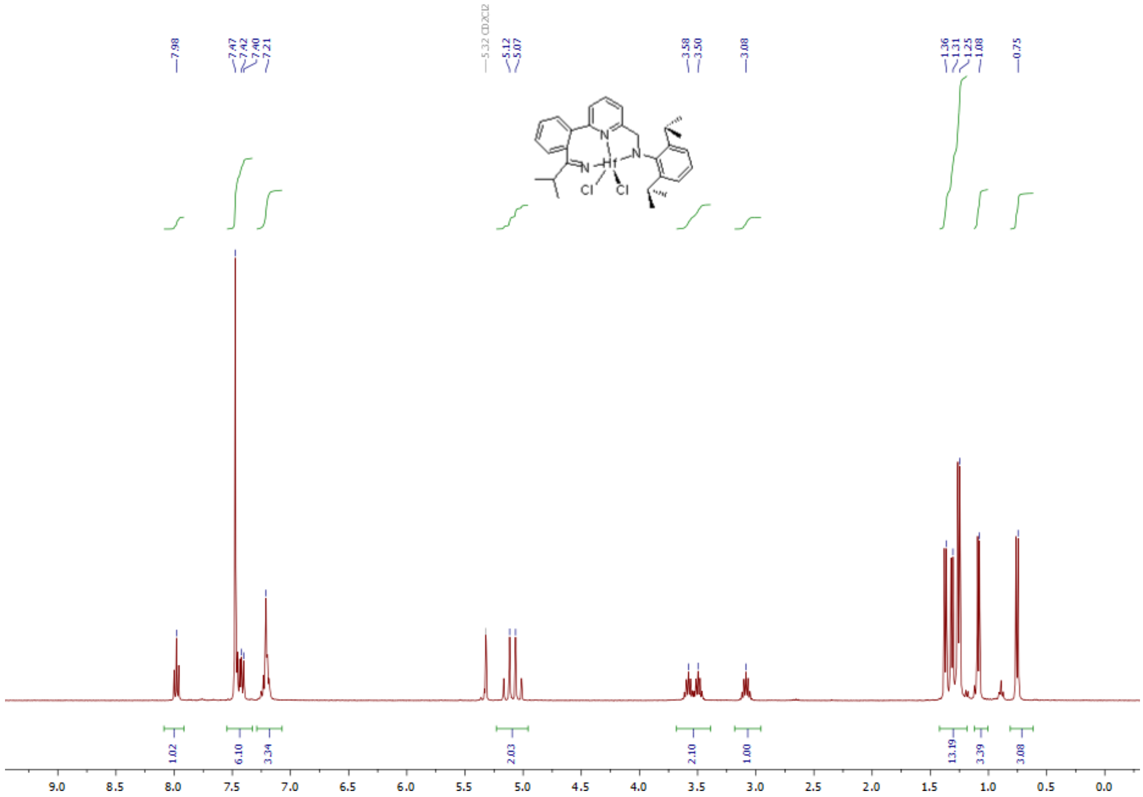

Figure S108. ^{1}H NMR spectrum of complex **3^{iPrCN}-HfCl$_2$** in CD_2Cl_2 at room temperature.

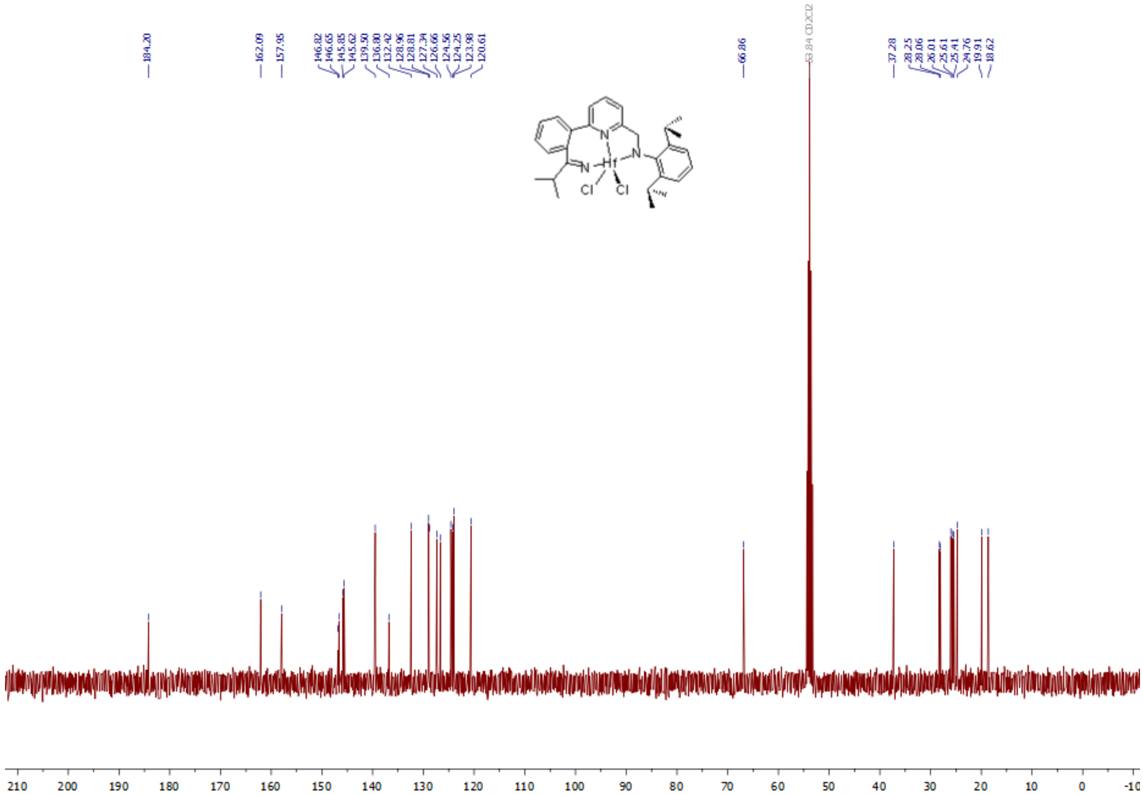

Figure S109. $^{13}C\{^{1}H\}$ NMR spectrum of complex **3^{iPrCN}-HfCl$_2$** in CD_2Cl_2 at room temperature.

2.30. Complex 3^{iPrCN}-HfMe$_2$

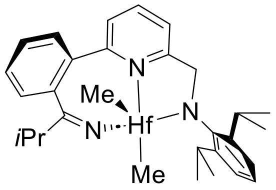

0.44 mL of 3.6 M MeMgBr (1.584 mmol) in diethyl ether was added via glass syringe to a solution of 400 mg (0.605 mmol) of complex **3^{iPrCN}-HfCl$_2$** in 20 mL of diethyl ether at -30 °C. The obtained mixture was stirred for 2 h at room temperature and then evaporated to dryness. The residue was extracted with 200 mL of hexanes, and the thus obtained suspension was filtered through a short pad of Celite®. The filtrate was concentrated until precipitation occurred. This precipitate was filtered off, washed with cold pentane, and dried in vacuum to give 220 mg (59%) of **3^{iPrCN}-HfMe$_2$** as a pale yellow solid. Anal calc. for $C_{30}H_{39}HfN_3$: C, 58.10; H, 6.34; N, 6.78. Found: C, 58.30; H, 6.59; N, 6.55.

^{1}H NMR (400 MHz, toluene-d_8) δ_H: 7.20–7.27 (3H, m), 6.98–7.06 (4H, m), 6.97 (1H, t, $^3J_{HH}$ = 7.8 Hz), 6.70 (1H, d, $^3J_{HH}$ = 7.6 Hz), 6.51 (1H, d, $^3J_{HH}$ = 7.6 Hz), 5.12 (1H, d, $^2J_{HH}$ = 20.2 Hz, CH_2(Py)NAr), 4.92 (1H, d, $^2J_{HH}$ = 20.2 Hz, CH_2(Py)NAr), 4.19 (1H, m), 3.62 (1H, m), 2.86 (1H, sept, $^3J_{HH}$ = 6.8 Hz), 1.54 (3H, d, $^3J_{HH}$ = 6.8 Hz), 1.53 (3H, d, $^3J_{HH}$ = 6.8 Hz), 1.36 (3H, d, $^3J_{HH}$ = 6.9 Hz), 1.30 (3H, d, $^3J_{HH}$ = 6.9 Hz), 1.21 (3H, d, $^3J_{HH}$ = 6.5 Hz), 0.69 (3H, d, $^3J_{HH}$ = 7.1 Hz), 0.20 (3H, s, Me_2Hf), -0.10 (3H, s, Me_2Hf).

^{13}C{^{1}H} NMR (101 MHz, toluene-d_8) δ_C: 179.4, 162.5, 159.1, 148.0, 146.4, 146.2, 146.1, 137.0, 131.8, 128.2, 127.3, 126.2, 124.5, 124.2, 123.7, 119.0, 68.2, 55.2, 51.1, 37.2, 28.2, 28.1, 26.4, 26.2, 25.5, 25.1, 19.9, 18.9.

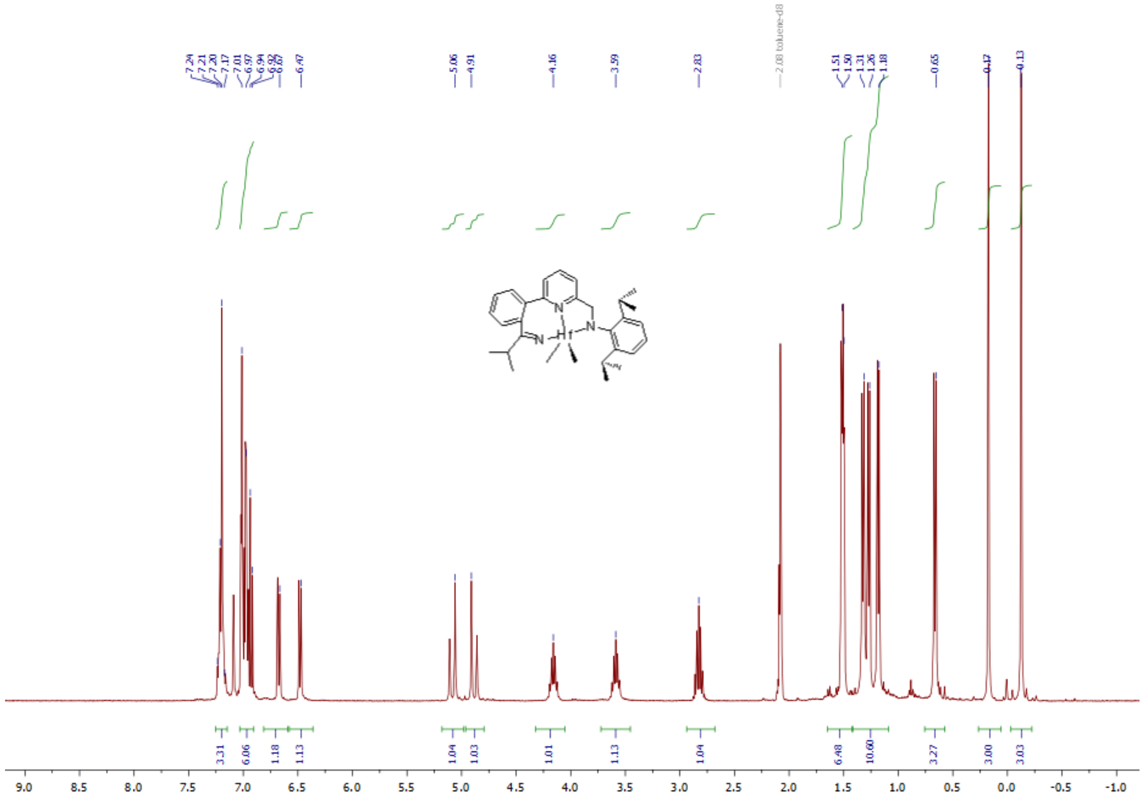

Figure S110. ^{1}H NMR spectrum of complex **3^{iPrCN}-HfMe$_2$** in toluene-d_8 at room temperature.

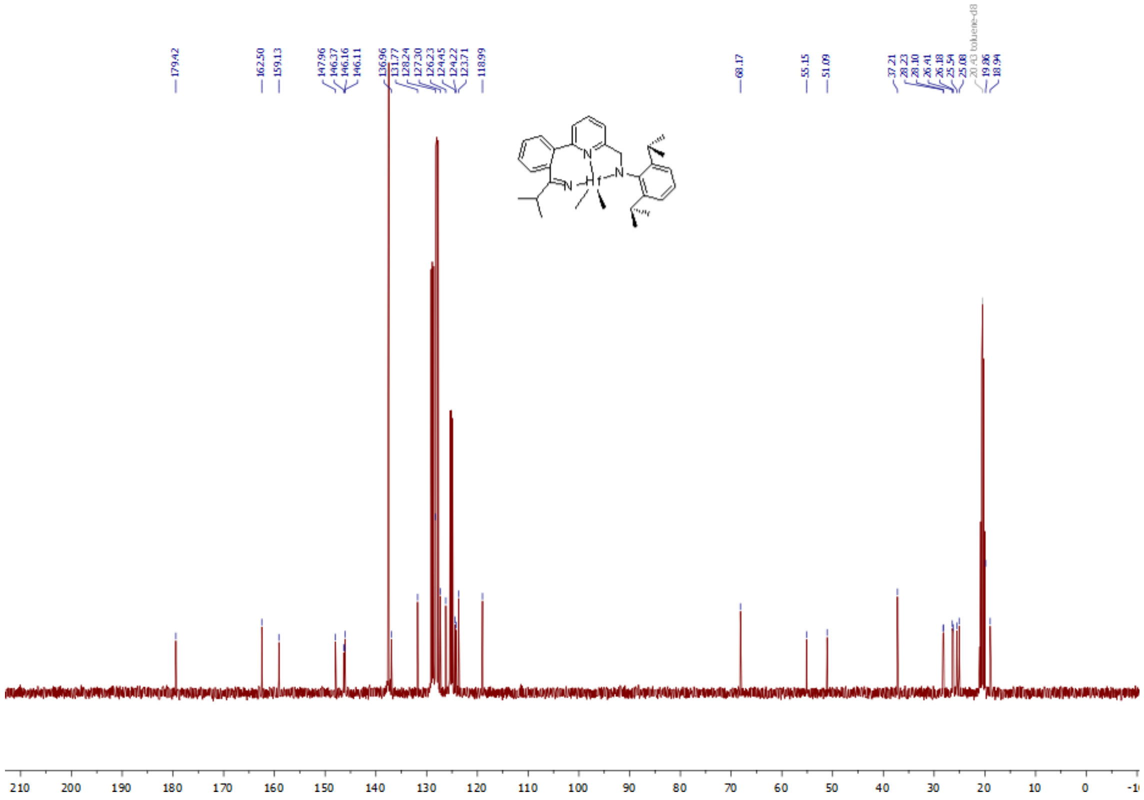

Figure S111. $^{13}C\{^{1}H\}$ NMR spectrum of complex **3^{iPrCN}-HfMe$_2$** in toluene-d_8 at room temperature.

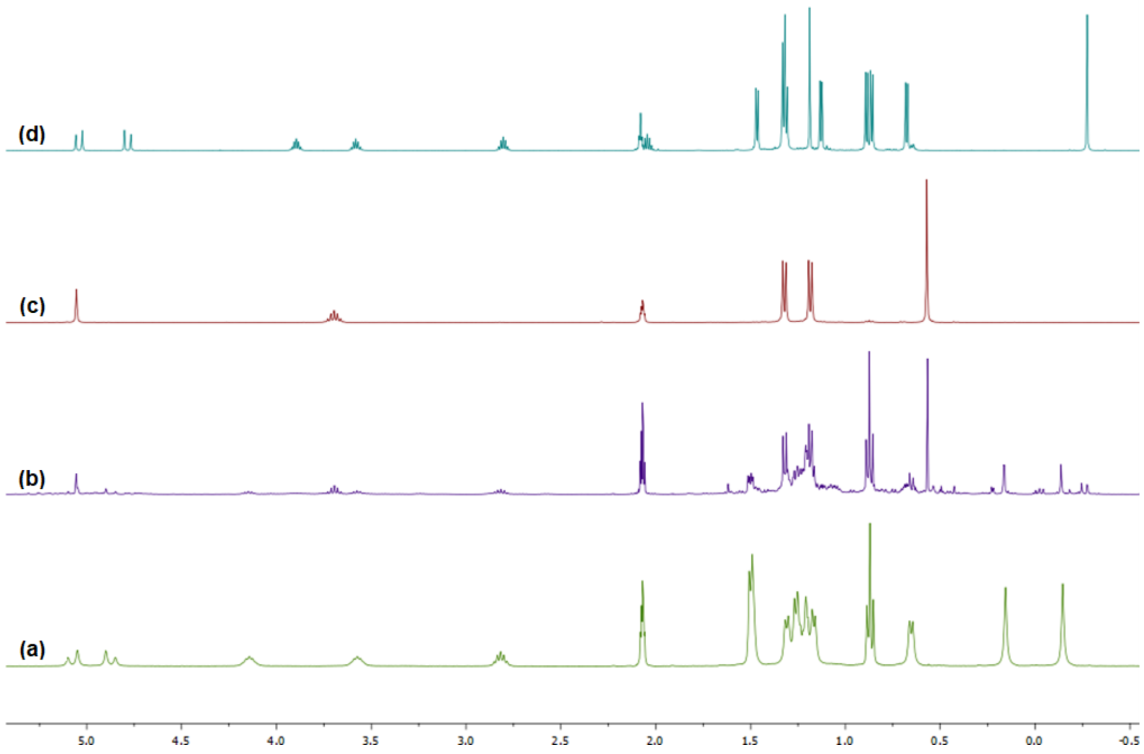

Figure S112. Fragments of ^{1}H NMR spectra of (from bottom to top) **(a) 3^{iPrCN}-HfMe$_2$** (freshly obtained), **(b) 3^{iPrCN}-HfMe$_2$** (after 8 days storage in solution at room temperature), **(c) 3-HfMe$_2$** and **(d) 3^{iPrCN}-HfMe(N=CMe*i*Pr)** in toluene-d_8 at room temperature.

2.31. Complex 3^{iPrCN}-HfMe(N=CMe*i*Pr)

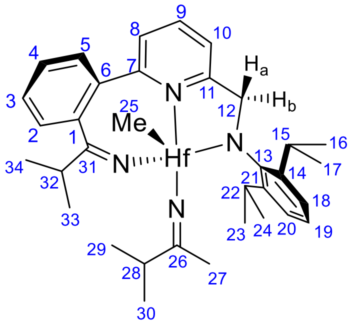

Complex **3-HfMe$_2$** (20 mg, 0.036 mmol) was dissolved in toluene-*d$_8$* (0.6 mL) in a J Young NMR tube, and then isobutyronitrile (3.2 µL, 2.5 mg, 0.072 mmol) was added in one portion via Eppendorf pipette at room temperature. The resulting yellow solution was studied by NMR spectroscopy.

^{1}H NMR (400 MHz, toluene-*d$_8$*) δ_H: 7.13–7.19 (4H, m, C(5)H, C(18)H, C(19)H and C(20)H), 7.06 (1H, m, C(2)H), 6.99–7.03 (3H, m, C(3)H, C(9)H and C(4)H), 6.79 (1H, d, $^3J_{HH}$ = 7.6 Hz, C(8)H), 6.56 (1H, d, $^3J_{HH}$ = 7.8 Hz, C(10)H), 5.04 (1H, d, $^2J_{HH}$ = 20.3 Hz, C(12)H$_a$), 4.78 (1H, d, $^2J_{HH}$ = 20.3 Hz, C(12)H$_b$), 3.90 (1H, sept, $^3J_{HH}$ = 6.9 Hz, C(15)H), 3.58 (1H, sept, $^3J_{HH}$ = 6.9 Hz, C(22)H), 2.81 (1H, sept, $^3J_{HH}$ = 6.8 Hz, C(32)H), 2.05 (1H, sept, $^3J_{HH}$ = 6.9 Hz, C(28)H), 1.47 (3H, d, $^3J_{HH}$ = 6.8 Hz, C(23)H) 1.33 (6H, d, $^3J_{HH}$ = 6.9 Hz, C(16)H and C(17)H), 1.32 (3H, d, $^3J_{HH}$ = 6.9 Hz, C(24)H), 1.19 (3H, s, C(27)H), 1.13 (3H, d, $^3J_{HH}$ = 6.8 Hz, C(33)H), 0.89 (3H, d, $^3J_{HH}$ = 6.9 Hz, C(29)H), 0.86 (3H, d, $^3J_{HH}$ = 6.9 Hz, C(30)H), 0.68 (3H, d, $^3J_{HH}$ = 6.9 Hz, C(34)H), -0.27 (3H, s, C(25)H).

^{13}C{^{1}H} NMR (101 MHz, toluene-*d$_8$*) δ_C: 186.2, 176.79, 163.1, 159.0, 150.2, 148.4, 145.5, 145.4, 137.2, 137.1, 131.8, 128.3, 126.9, 125.2, 124.9, 124.0, 123.7, 123.6, 119.4, 66.8, 43.7, 40.9, 37.3, 28.5, 28.0, 27.9, 25.7, 25.62, 25.57, 25.5, 19.99, 19.98, 19.95, 19.1.

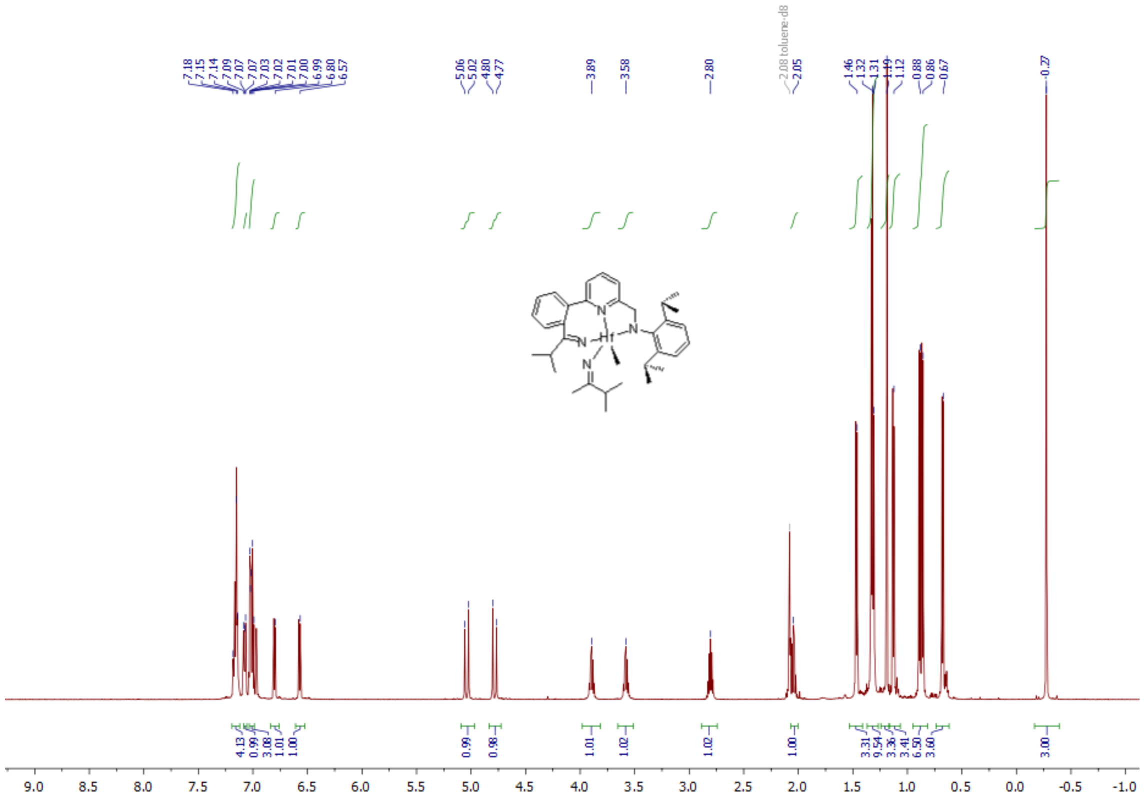

Figure S113. ^{1}H NMR spectrum of complex **3^{iPrCN}-HfMe(N=CMe*i*Pr)** in toluene-d_8 at room temperature.

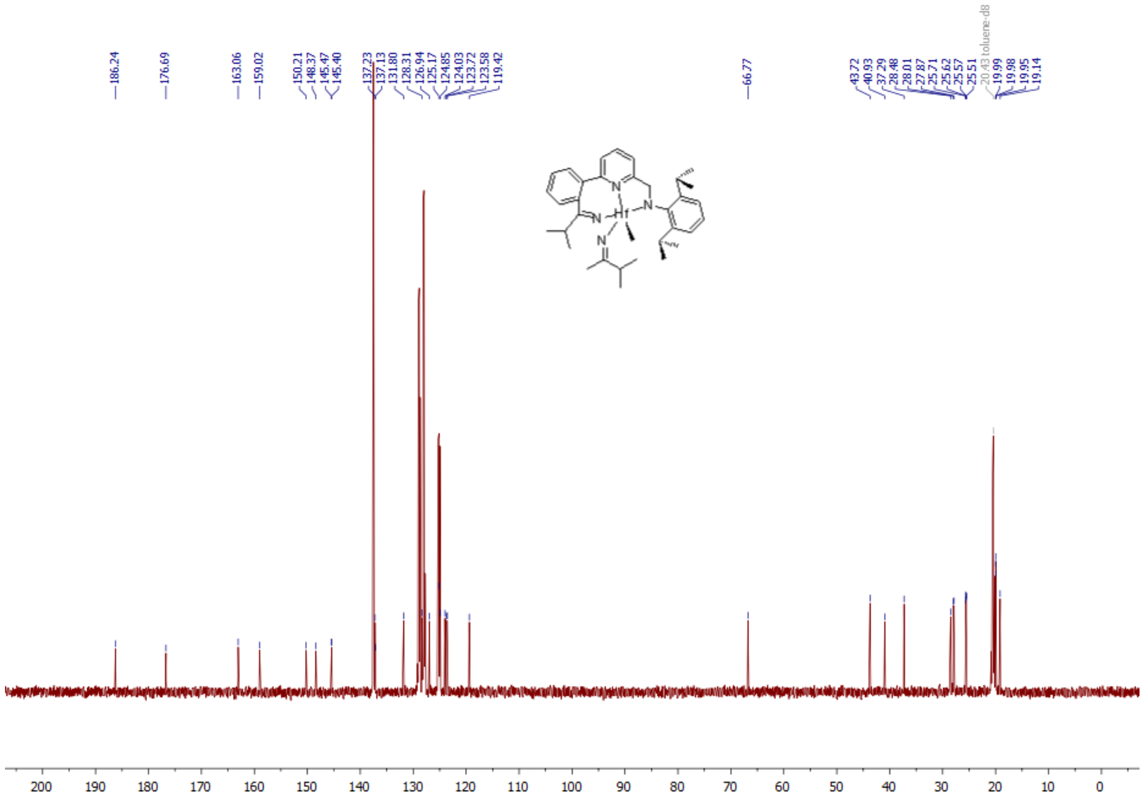

Figure S114. ^{13}C{^{1}H} NMR spectrum of complex **3^{iPrCN}-HfMe(N=CMe*i*Pr)** in toluene-d_8 at room temperature.

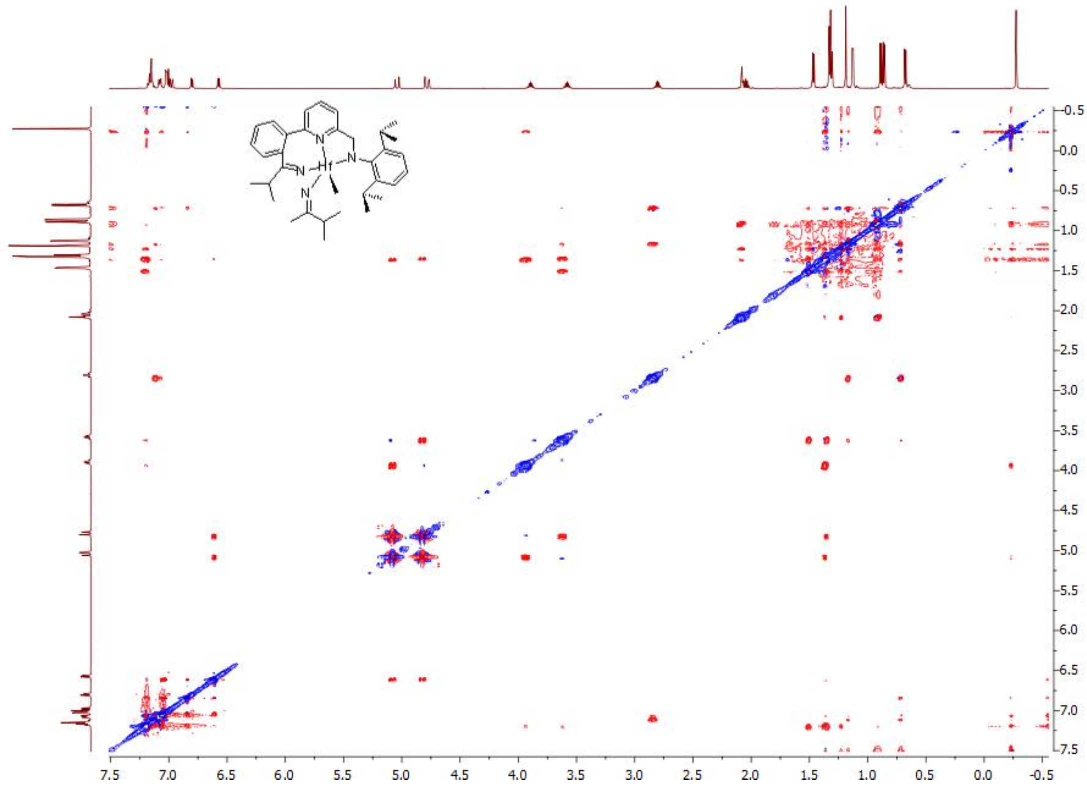

Figure S115. ^{1}H-^{1}H NOESY NMR spectrum of **3^{iPrCN}-HfMe(N=CMe*i*Pr)** in toluene-d_8 at room temperature.

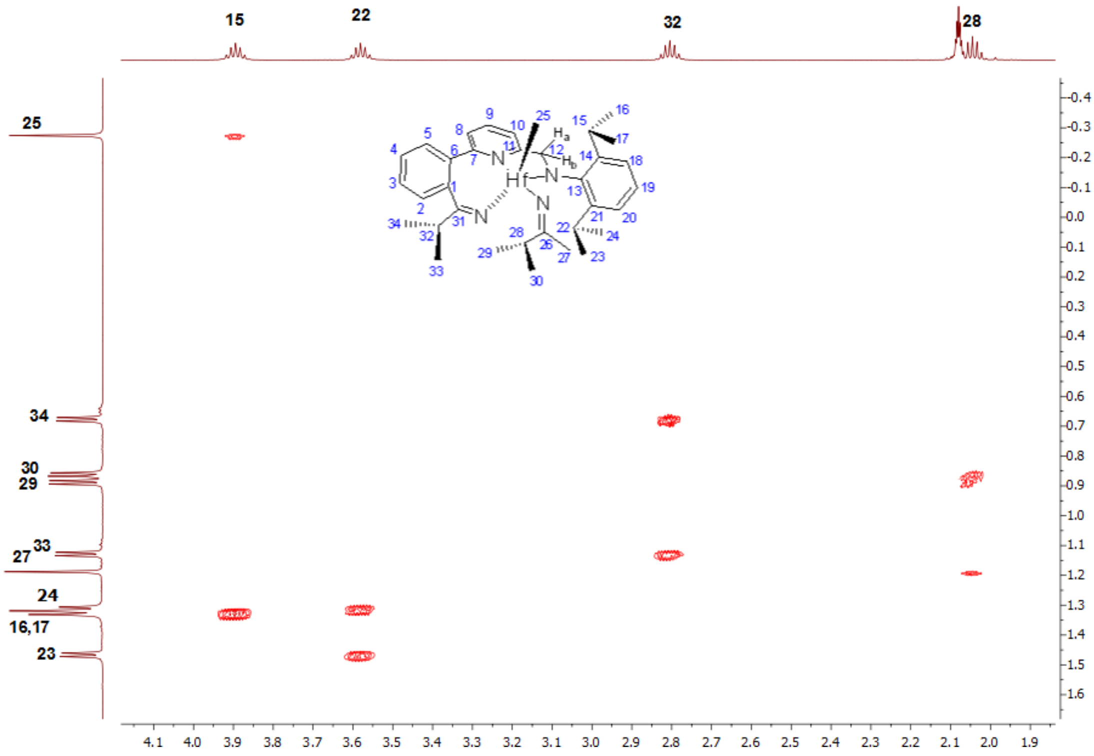

Figure S116. Fragment of ^{1}H-^{1}H NOESY NMR spectrum of **3^{iPrCN}-HfMe(N=CMe*i*Pr)** in toluene-d_8 at room temperature.

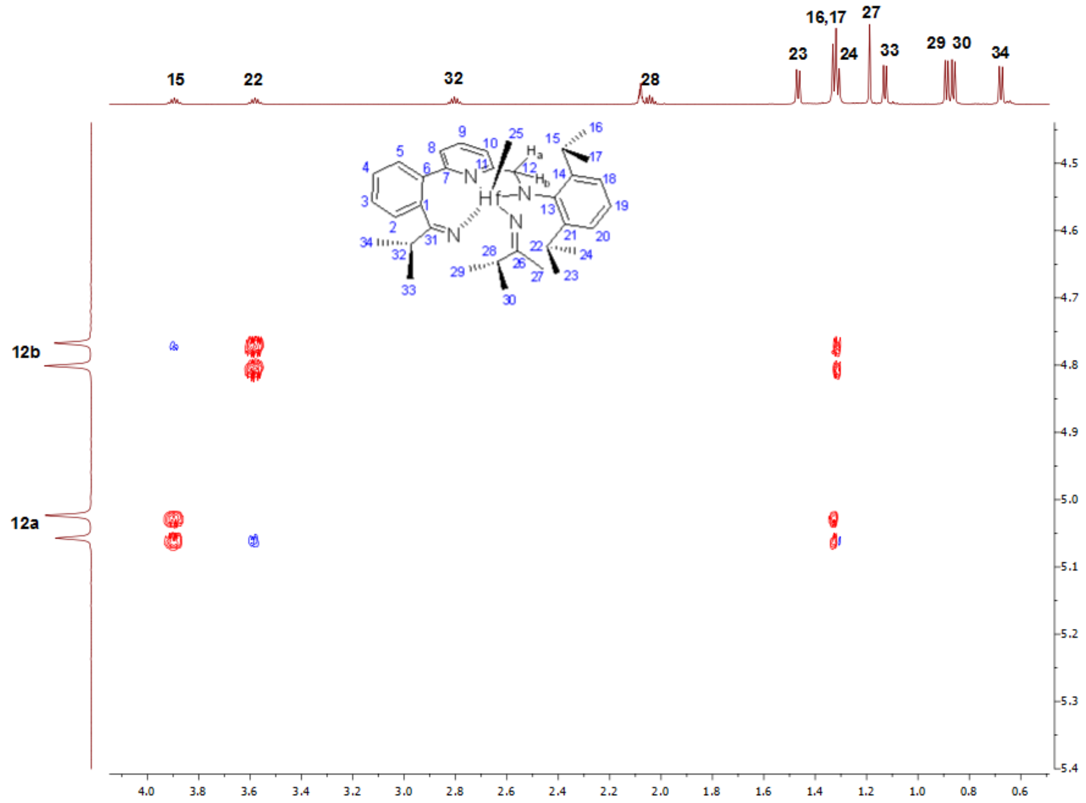

Figure S117. Fragment of ^{1}H-^{1}H NOESY NMR spectrum of 3^{iPrCN}-HfMe(N=CMe*i*Pr) in toluene-d_8 at room temperature.

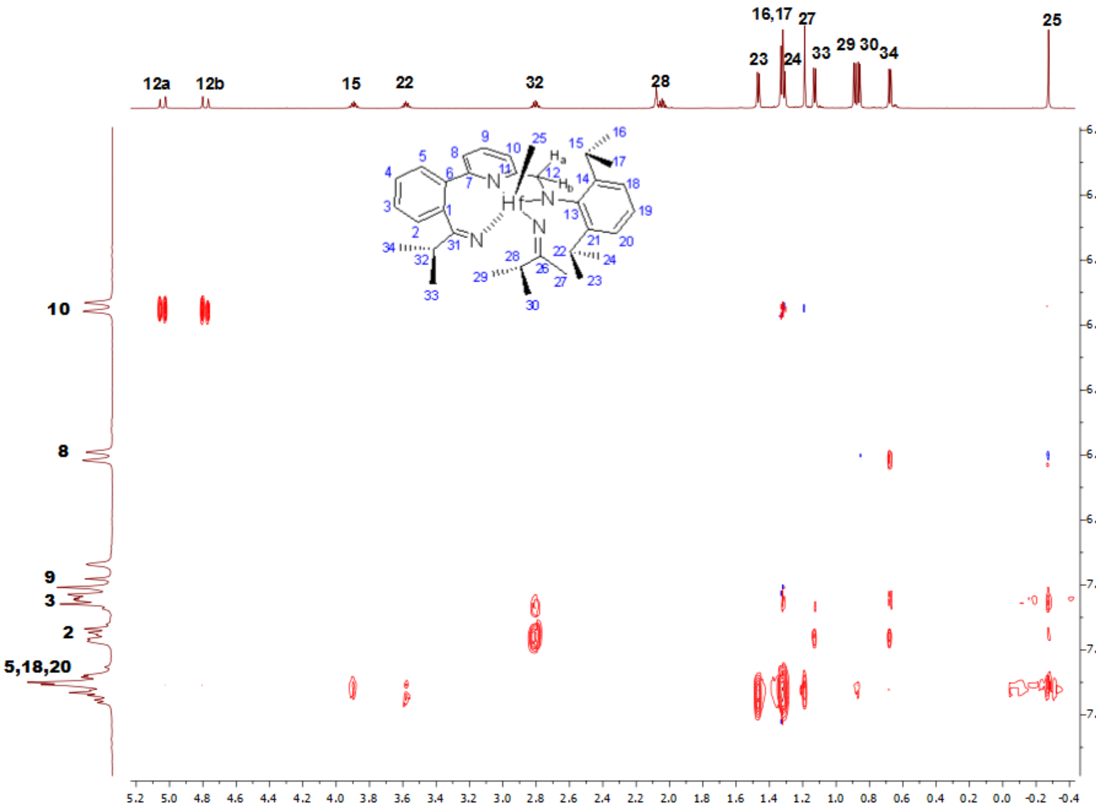

Figure S118. Fragment of ^{1}H-^{1}H NOESY NMR spectrum of 3^{iPrCN}-HfMe(N=CMe*i*Pr) in toluene-d_8 at room temperature.

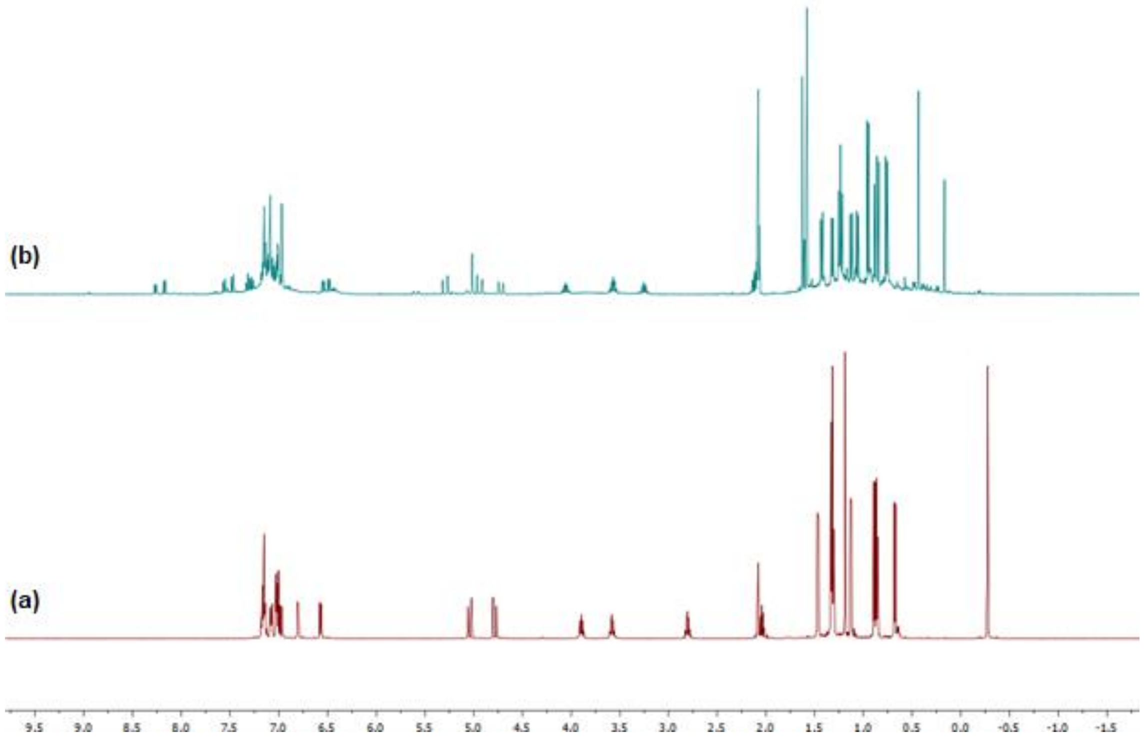

Figure S119. Degradation of complex **3^{iPrCN}-HfMe(N=CMe*i*Pr)** (spectrum **(a)**) after heating at 100 °C in toluene-d_8 overnight (spectrum **(b)**).

2.32. Substitution of an isobutyronitrile moiety with benzonitrile moiety in 1^{iPrCN}-HfCl₂.

Complex **1^{iPrCN}-HfCl$_2$** (29.3 mg, 41 µmol) was dissolved in *o*-dichlorobenzene-*d$_4$* (0.6 mL) in a J Young NMR tube, and then benzonitrile (4.3 mg, 41 µmol) was added in one portion via Eppendorf pipette at room temperature. This mixture was studied by NMR spectroscopy.

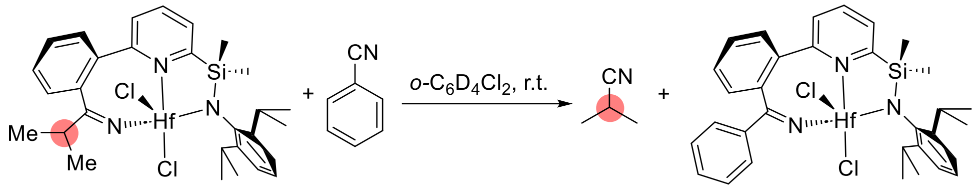

Scheme S2. Reaction of Substitution of isobutyronitrile moiety with benzonitrile moiety in **1^{iPrCN}-HfCl$_2$**.

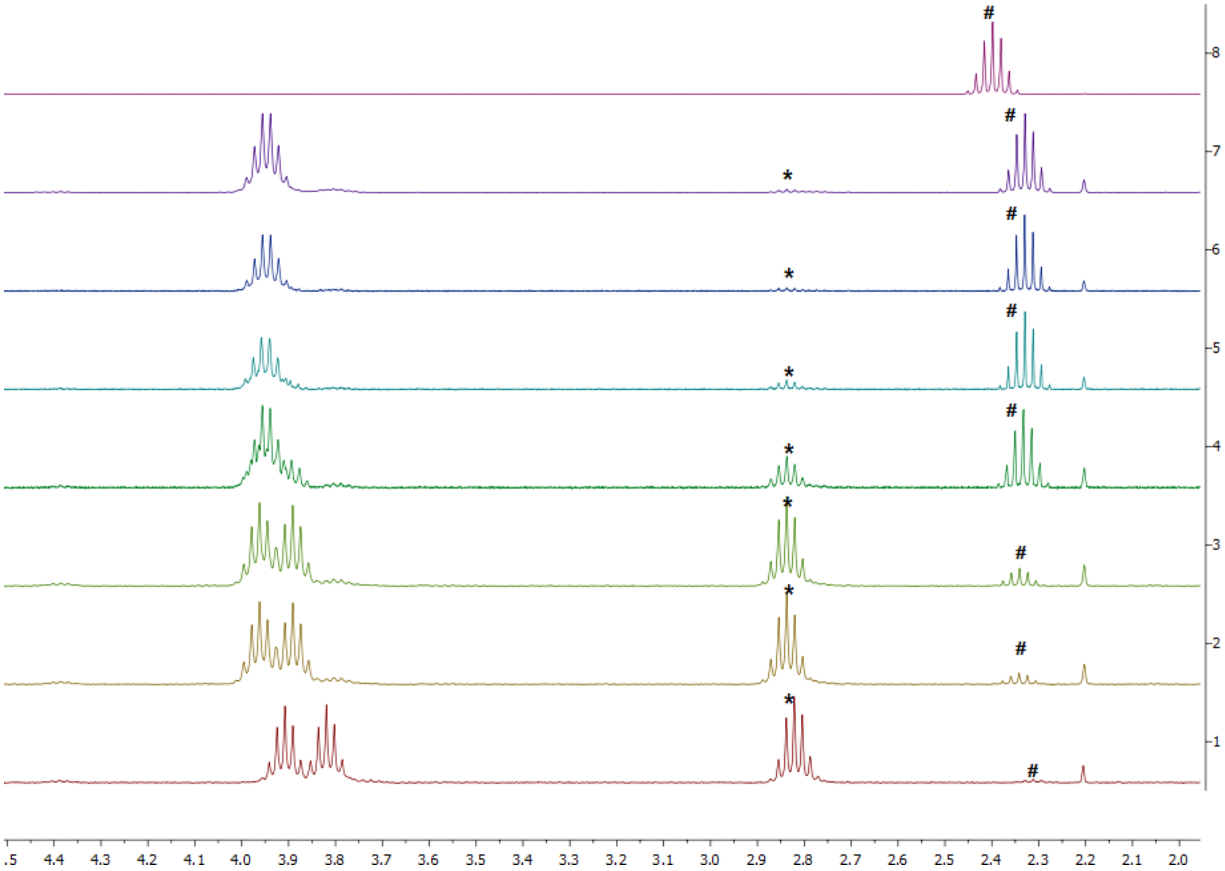

Figure S120. Fragment of ^{1}H NMR spectra of a mixture of **1^{iPrCN}-HfCl$_2$**, **1^{PhCN}-HfCl$_2$** and *i*PrCN after various time upon mixing of **1^{iPrCN}-HfCl$_2$** and 1 equiv. of PhCN in *o*-dichlorobenzene-*d$_4$* at room temperature. A character (*****) denotes N=C-C*H*-Me$_2$ in **1^{iPrCN}-HfCl$_2$**, (**#**) – "free" *i*PrCN. From bottom to top: 1 - only **1^{iPrCN}-HfCl$_2$**, 2- 15 min mix time, 3 - 1 h, 4 - 17 h, 5 - 46 h, 6 - 94 h, 7 - 120 h, 8 - pure *i*PrCN. Some peak shifts are observed possibly due to minor changes in coordination environment.

3. Variable temperature van't Hoff study of the equilibrium of 1^{iPrCN}-HfMe(N=CMe*i*Pr) and 1-HfMe(N=CMe*i*Pr)

Complex **1^{iPrCN}-HfMe(N=CMe*i*Pr)** was dissolved in toluene-d_8 and transferred to a sealed J. Young NMR tube. The tube was inserted into a temperature-controlled NMR probe, and ^{1}H NMR spectra were collected at 330, 340, 345, 350 and 360 K, allowing 20 min for equilibration at each temperature. Concentrations of **1^{iPrCN}-HfMe(N=CMe*i*Pr)** and **1-HfMe(N=CMe*i*Pr)** were determined by integration of the aniline isopropyl C-H resonance for the respective complexes, and *i*PrCN concentration was assumed to be equal to [**1-HfMe(N=CMe*i*Pr)**]. The equilibrium constant of the reaction was calculated according to the equation:

$$K_{obs} = \frac{[\mathbf{1-HfMe(N=CMe}\boldsymbol{i}\mathbf{Pr)}]^2}{[\mathbf{1}^{\boldsymbol{i}\mathbf{PrCN}}\mathbf{-HfMe(N=CMe}\boldsymbol{i}\mathbf{Pr)}]}$$

The plot of ln(K_{obs}) as function of T^{-1} was fit by a line according to the equation:

$$\ln(K_{obs}) = -\frac{\Delta H}{RT} + \frac{\Delta S}{R}$$

The enthalpy and entropy of the reaction were extracted from the slope and intercept, respectively.

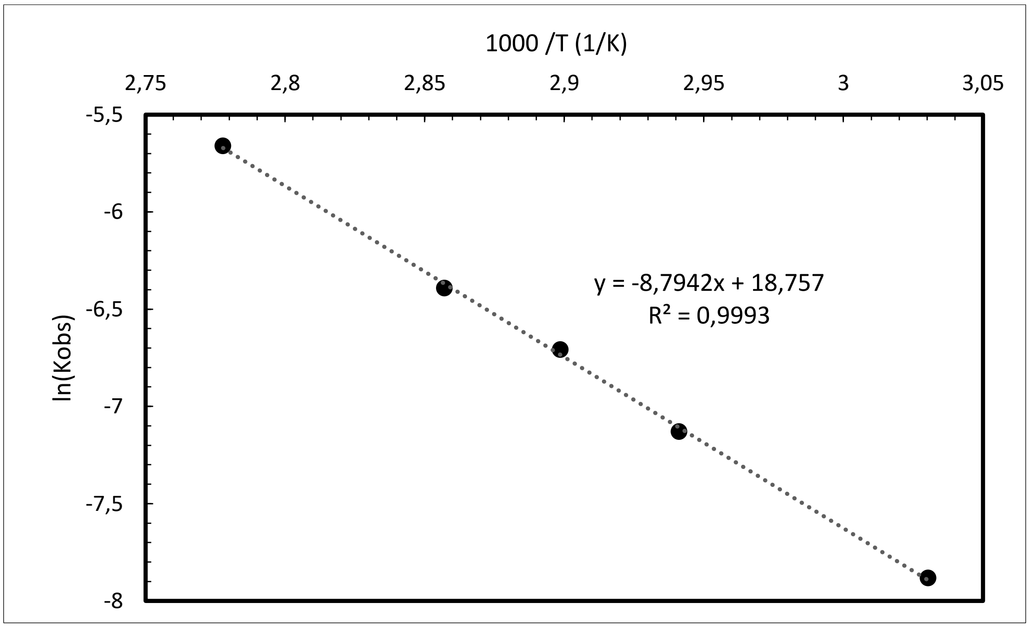

Figure S121. Van't Hoff plot derived from variable temperature ^{1}H NMR spectra of the equilibrium between **1^{iPrCN}-HfMe(N=CMe*i*Pr)** and **1-HfMe(N=CMe*i*Pr)** in toluene-d_8 from 330 to 360 K.

4. Variable temperature van't Hoff study of the equilibrium of **1^{iPrCN}-HfCl$_2$** and **1-HfCl$_2$**

Complex **1^{iPrCN}-HfCl$_2$** was dissolved in toluene-d_8 and transferred to a sealed J. Young NMR tube. The tube was inserted into a temperature-controlled NMR probe, and ^{1}H NMR spectra were collected at 348, 353, 358, 363 and 368 K, allowing 20 min for equilibration at each temperature. Concentrations of **1^{iPrCN}-HfCl$_2$** and **1-HfCl$_2$** were determined by integration of the aniline isopropyl C-H resonance for the respective complexes, and *i*PrCN concentration was assumed to be equal to [**1-HfCl$_2$**]. The equilibrium constant of the reaction was calculated according to the equation:

$$K_{obs} = \frac{[\mathbf{1-HfCl_2}]^2}{[1^{\boldsymbol{iPrCN}} - \mathbf{HfCl_2}]}$$

The plot of ln(K_{obs}) as function of T^{-1} was fit by a line according to the equation:

$$\ln(K_{obs}) = -\frac{\Delta H}{RT} + \frac{\Delta S}{R}$$

The enthalpy and entropy of the reaction were extracted from the slope and intercept, respectively.

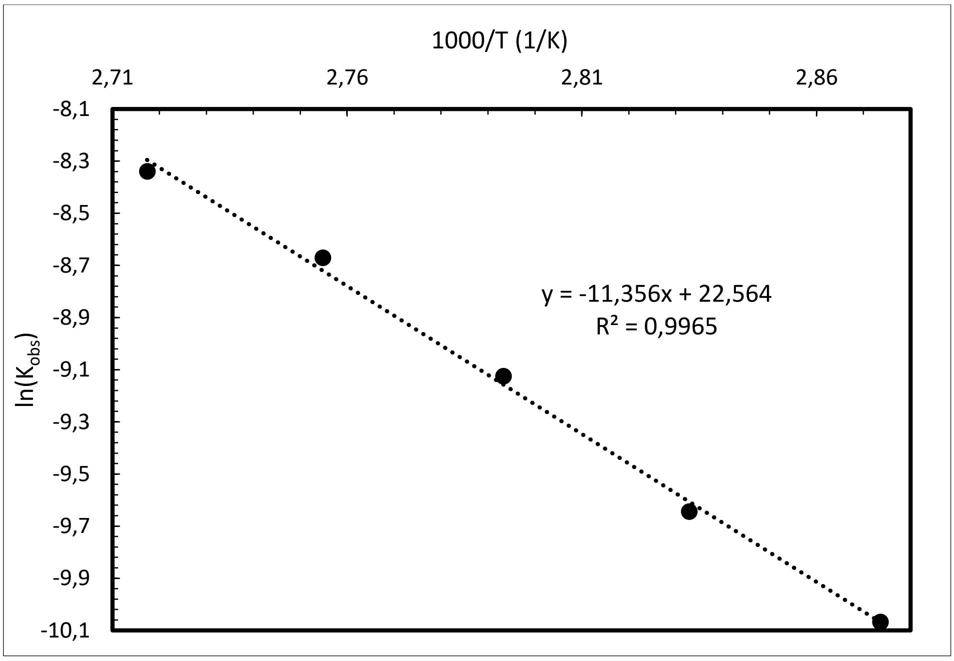

Figure S122. Van't Hoff plot derived from variable temperature ^{1}H NMR spectra of the equilibrium between **1^{iPrCN}-HfCl$_2$** and **1-HfCl$_2$** in toluene-d_8 from 348 to 368 K.

5. Variable temperature van't Hoff study of the equilibrium of 1^{iPrCN}-$HfCl_2$ and 1-$HfCl_2$

Complex **1^{iPrCN}-$HfCl_2$** was dissolved in *o*-$C_6D_4Cl_2$ and transferred to a sealed J. Young NMR tube. The tube was inserted into a temperature-controlled NMR probe, and ^{1}H NMR spectra were collected at 330, 340, 350, 360 and 368 K, allowing 20 min for equilibration at each temperature. Concentrations of **1^{iPrCN}-$HfCl_2$** and **1-$HfCl_2$** were determined by integration of the aniline isopropyl C-H resonance for the respective complexes, and *i*PrCN concentration was assumed to be equal to [**1-$HfCl_2$**]. The equilibrium constant of the reaction was calculated according to the equation:

$$K_{obs} = \frac{[\mathbf{1-HfCl_2}]^2}{[\mathbf{1^{iPrCN}-HfCl_2}]}$$

The plot of ln(K_{obs}) as function of T^{-1} was fit by a line according to the equation:

$$\ln(K_{obs}) = -\frac{\Delta H}{RT} + \frac{\Delta S}{R}$$

The enthalpy and entropy of the reaction were extracted from the slope and intercept, respectively.

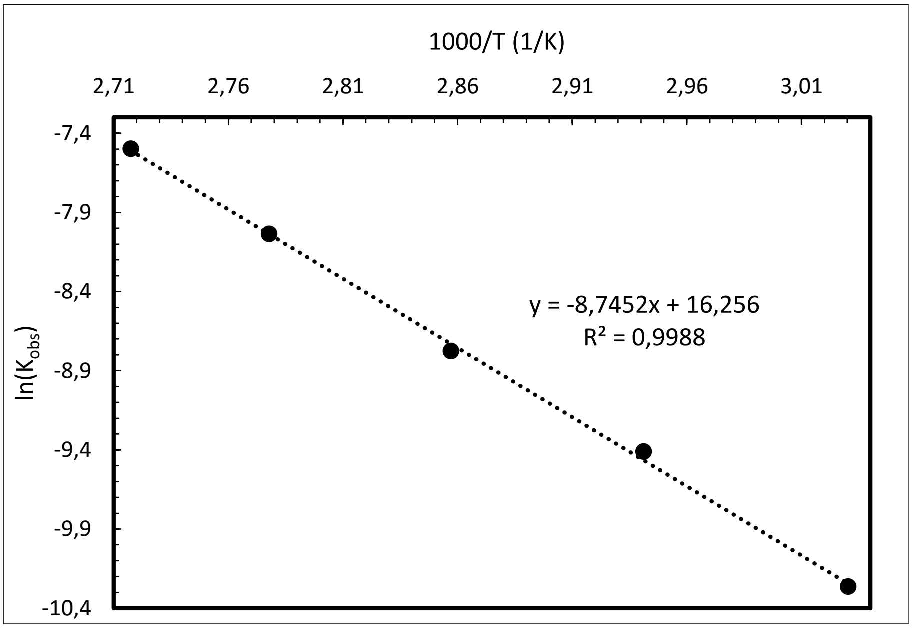

Figure S123. Van't Hoff plot derived from variable temperature ^{1}H NMR spectra of the equilibrium between **1^{iPrCN}-$HfCl_2$** and **1-$HfCl_2$** in *o*-$C_6D_4Cl_2$ from 348 to 368 K.

6. Spin saturation transfer difference for **1^{iPrCN}-HfCl$_2$**

Spin saturation transfer difference experiments [4] were used to determine the activation parameters of dissociation of **1^{iPrCN}-HfCl$_2$** into **1-HfCl$_2$** and *i*PrCN. In a glovebox, **1^{iPrCN}-HfCl$_2$** was dissolved in *ortho*-dichlorodeuterobenzene in a J. Young NMR tube.

Data acquisitions were performed using a standard 1D STD pulse program "stddiff" (provided by the manufacturer) in a similar fashion to the experiments reported in the literature [4–6]. At each of the selected temperatures (345–365 K), series of spectra, both with on-resonance and off-resonance irradiation, were acquired using different saturation times (d20, 0.35 – 32 s). For each of the selected temperatures, the relaxation delay (d1) was kept constant and was equal to the longest saturation time in the group of experiments. 50 ms selective Gaussian pulses were used to saturate the signal of interest as well as for spectra with off-resonance irradiation.

On-resonance frequency was selected at 2.319 ppm (CH proton of isobutyronitrile), which affected the resonance at 2.758 ppm (corresponding CH proton of **1^{iPrCN}-HfCl$_2$**). The off-resonance frequency was set at 40 ppm. For all the SSTD experiments, the spectral width was set between 1.6 and 4.6 ppm. Integration was carried out using Topspin 4.1.1.

The spectra were phase corrected, and the difference spectra were obtained followed by baseline correction for the off-resonance and difference spectra.

The spin saturation transfer difference (SSTD) factor, η_{SSTD}, values were calculated as the value of the integral of resonance at 2.758 ppm obtained from the difference spectrum divided by the integral of the same resonance obtained from the off-resonance spectrum. These values were then plotted versus saturation time, and an exponential function was fitted to these values allowing us to obtain the rate constants.

$$\eta_{SSTD}(t) = \frac{k}{\left(1/_{T_1} + k\right)}(1 - \exp(-(1/_{T_1} + k) \cdot t)$$

$$\eta_{SSTD}(t) = \frac{a}{b}(1 - \exp(-b \cdot t)$$

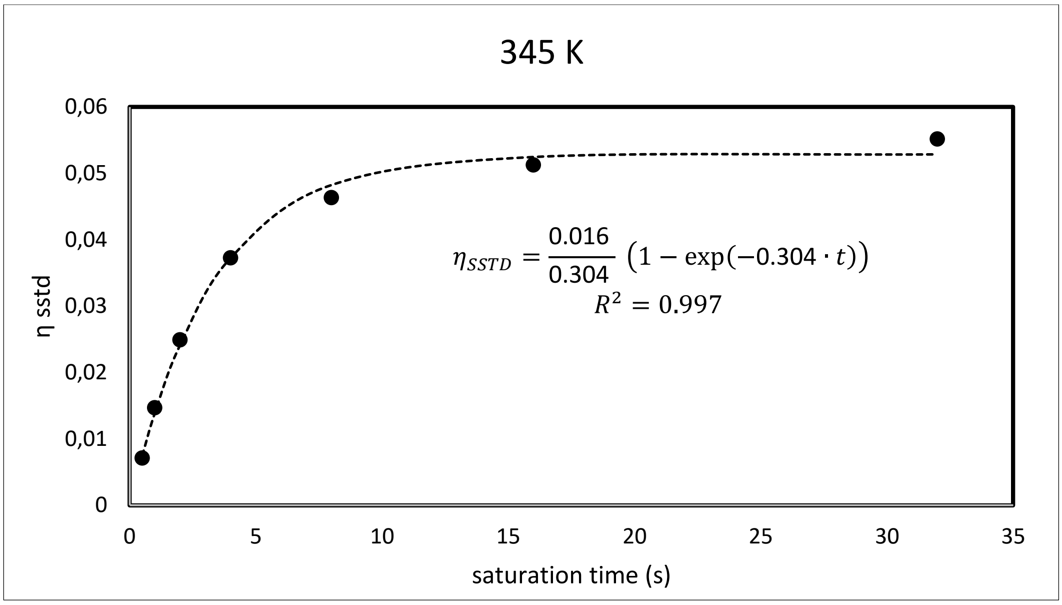

Figure S124. Plot of SSTD factor versus saturation times at 345 K with the exponential fit.

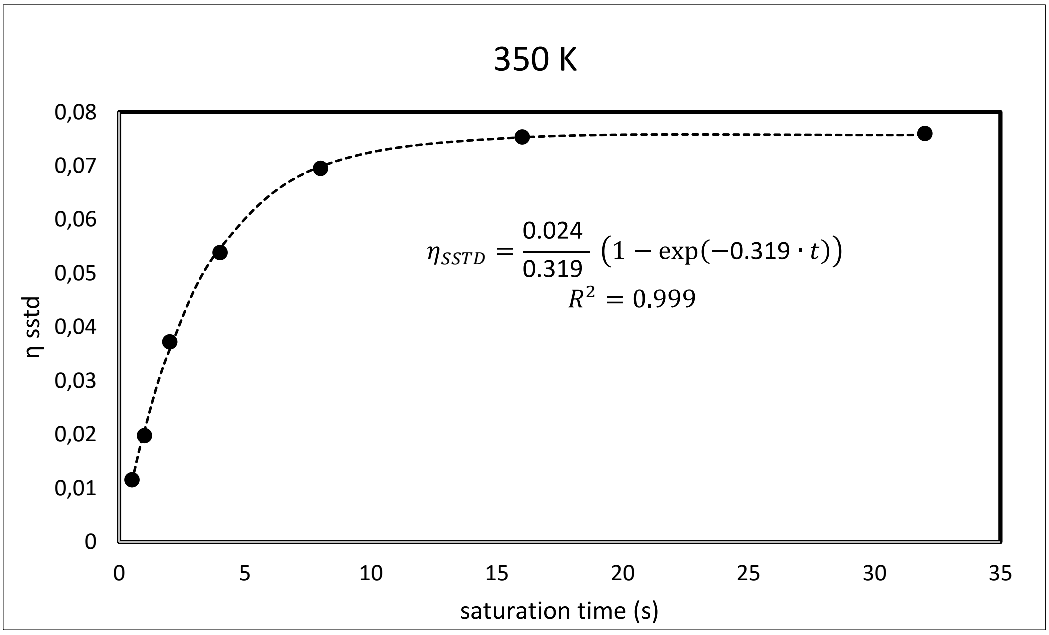

Figure S125. Plot of SSTD factor versus saturation times at 350 K with the exponential fit.

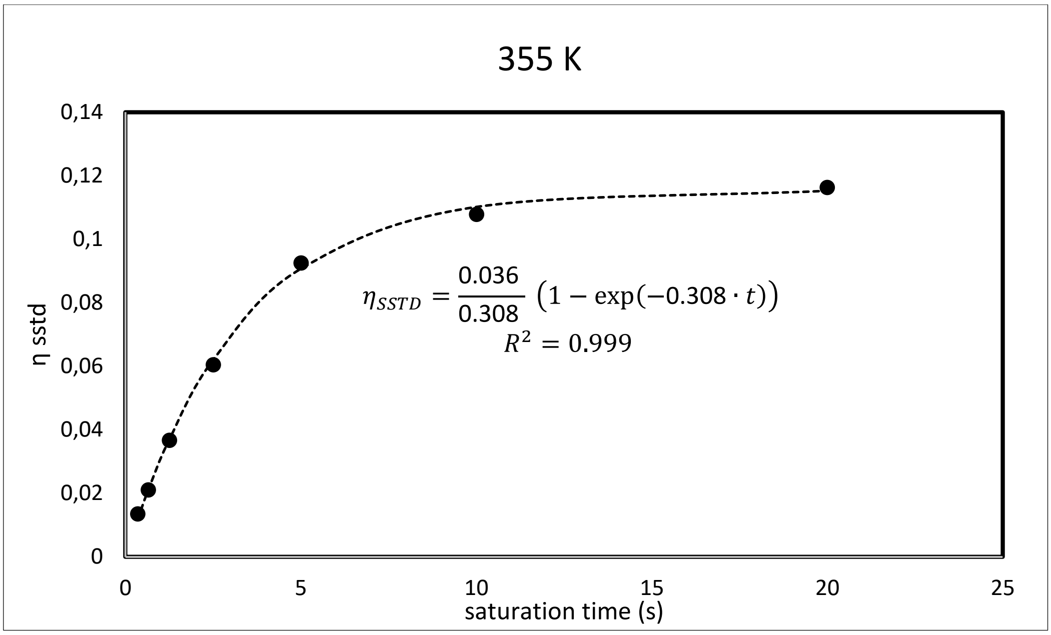

Figure S126. Plot of SSTD factor versus saturation times at 355 K with the exponential fit.

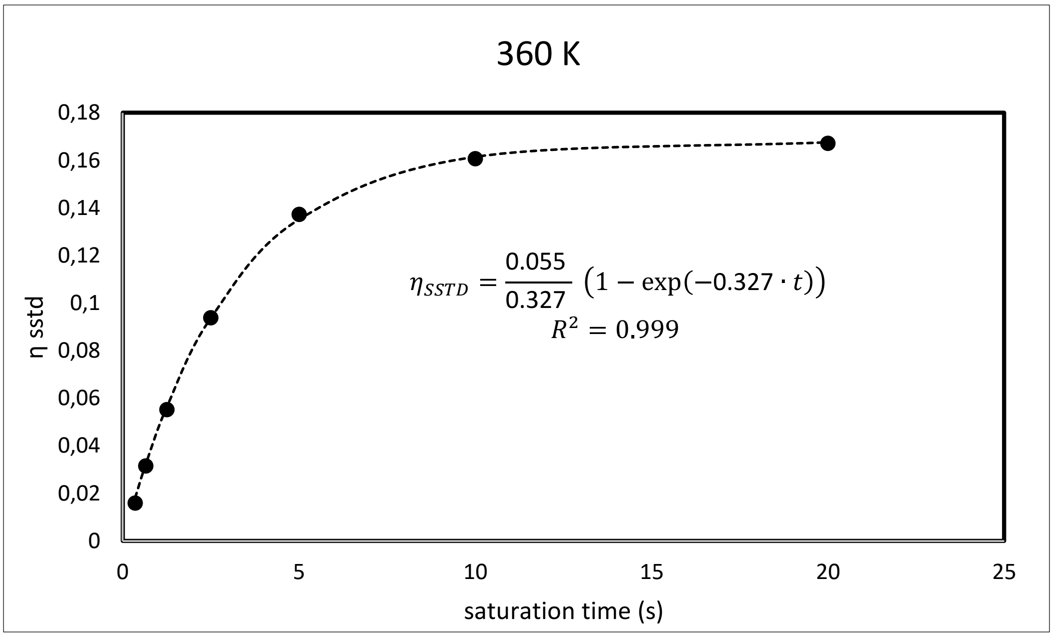

Figure S127. Plot of SSTD factor versus saturation times at 360 K with the exponential fit.

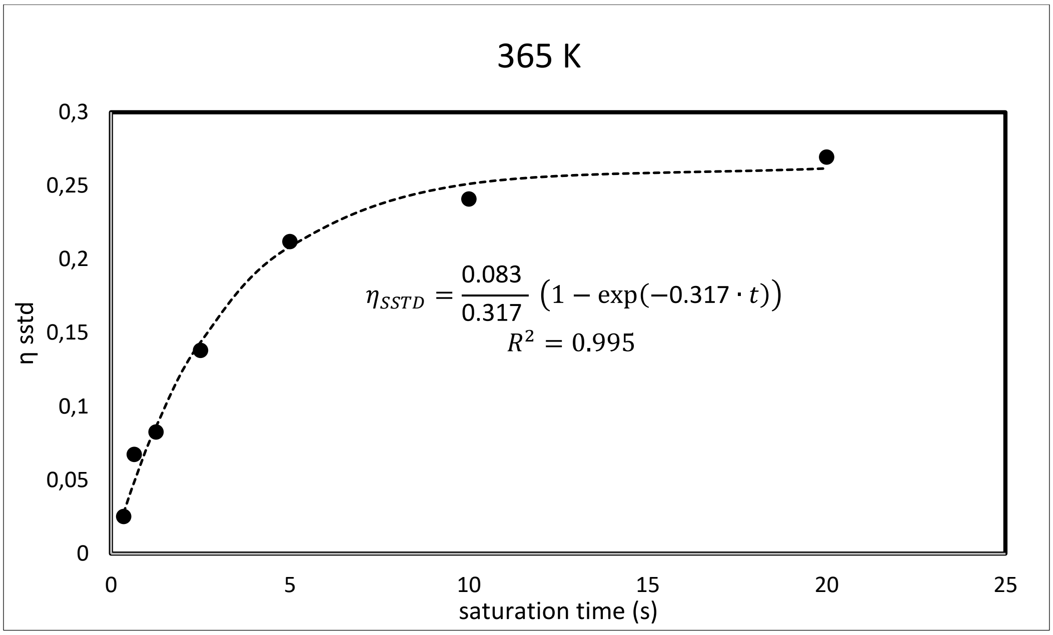

Figure S128. Plot of SSTD factor versus saturation times at 365 K with the exponential fit.

In order to determine the activation parameters of the reaction the obtained rate constants were fitted to a linearized form of the Eyring equation, the slope and the intercept were used to determine the enthalpy and entropy of activation, respectively. The errors in the rate constants were evaluated to be 5% based on our previous experience of using this method. Uncertainties in the activation parameters were estimated from the propagation of error formulae [7].

Table S1. The obtained rate constants for the process of *β*-aryl elimination from **1^{iPrCN}-HfCl$_2$** at various temperature in *ortho*-dichlorodeuterobenzene.

T (K)	*k* (s^{-1})	ln(*k*/T)	1000/T
345	0.016	-9.972	2.899
350	0.024	-9.579	2.857
355	0.036	-9.210	2.817
360	0.055	-8.788	2.778
365	0.083	-8.386	2.740

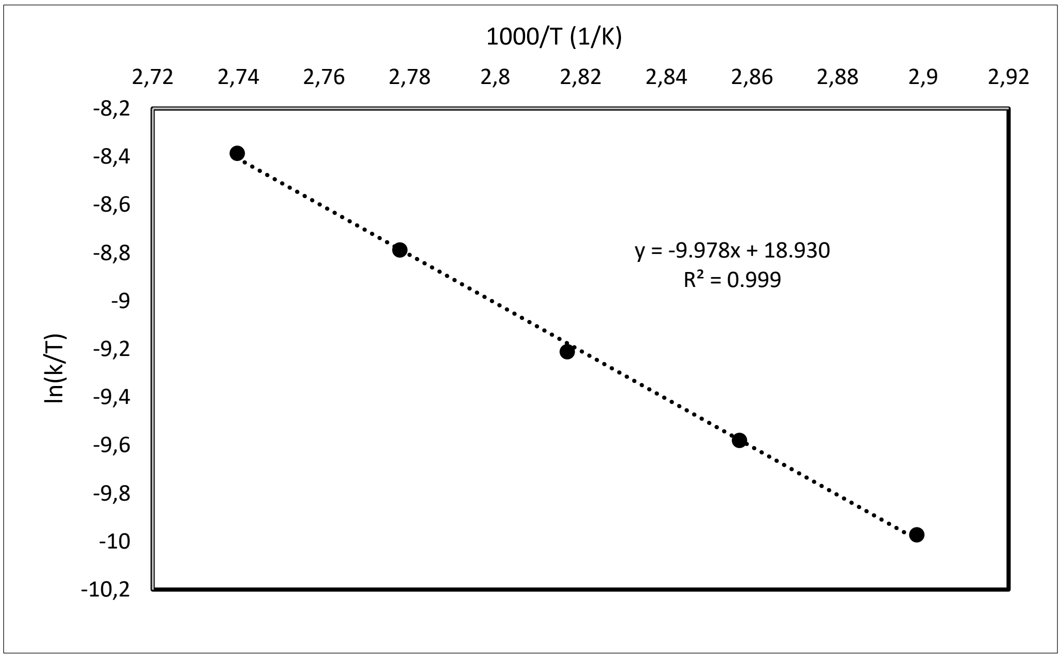

Figure S129. Eyring plot for the rates of the **1^{iPrCN}-HfCl$_2$** to dissociate to **1-HfCl$_2$** and *i*PrCN in *ortho*-dichlorodeuterobenzene at heating.

$$ln\left(\frac{k}{T}\right) = ln\left(\frac{k_B}{h}\right) + \frac{\Delta S^{\neq}}{R} - \frac{\Delta H^{\neq}}{R} \cdot \frac{1}{T}$$

$$\Delta H^{\neq} = 83.0\ \pm 7.1\ kJ \cdot mol^{-1}$$

$$\Delta S^{\neq} = -40.2\ \pm 20.0\ J \cdot mol^{-1} \cdot K^{-1}$$

$$\Delta G^{\neq}_{298} = 94.9\ \pm 13.0\ kJ \cdot mol^{-1} = 22.7\ \pm 3.1\ kcal \cdot mol^{-1}$$

7. Crystal structure determinations.

X-ray experiments were carried out using Bruker D8 Quest diffractometer with Photon III detector (l(Mo-*Ka*)=0.71073 Å, graphite monochromator, w-scans) at 120 °K. All structures were solved by direct methods and refined by the full-matrix least-squares procedure in anisotropic approximation for non-hydrogen atoms. All the hydrogen atoms were placed in geometrically calculated positions and included in the refinement using a riding approximation. The details of data collection and crystal structures refinement for which we used SAINT Plus [8], SADABS [9] and SHELXL-2018-3 [10] program packages are summarized in Tables S2–S8. Crystallographic data for **1^{iPrCN}-HfMe(Cl/Br)**, **1-HfMe$_2$**, **1^{tBuCN}-HfMe$_2$**, **1-HfMe(N=CMe$_2$)**, **1^{iPrCN}-HfMe(N=CMe*i*Pr)**, **1^{PhCN}-HfMe(N=CMePh)** and **2-Zr(N=CMe*i*Pr)$_2$** have been deposited with the Cambridge Crystallographic Data Center, CCDC Nos. 2301574–2301580. Copies of this information may be obtained from the Director, CCDC, 12 Union Road, Cambridge CB2 1EZ, UK (Fax: +44 1223 336033; e-mail: deposit@ccdc.cam.ac.uk or www.ccdc.cam.ac.uk).

Table S2. Crystal data and structure refinement for **1^{iPrCN}-HfMe(Cl/Br)** (CCDC 2301578).

Empirical formula	$C_{30}H_{40}Br_{0.50}Cl_{0.50}HfN_3Si$
Formula weight	706.91
Temperature/K	120(2)
Crystal system	monoclinic
Space group	$P2_1$
a/Å	9.2803(7)
b/Å	37.819(3)
c/Å	9.6856(7)
α/°	90
β/°	114.1197(15)
γ/°	90
Volume/Å^3	3102.6(4)
Z	4
ρ_{calc}g/cm^3	1.513
μ/mm^{-1}	4.110
F(000)	1412
Crystal size/mm^3	0.325 × 0.289 × 0.276
Radiation	MoKα (λ = 0.71073)
2Θ range for data collection/°	2.154 to 28.998
Index ranges	-12 ≤ h ≤ 12, -51 ≤ k ≤ 51, -13 ≤ l ≤ 13
Reflections collected	37966
Independent reflections	16404 [R_{int} = 0.0478]
Data/restraints/parameters	16404/23/677
Goodness-of-fit on F^2	1.020
Final R indexes [I>=2σ (I)]	R_1 = 0.0447, wR_2 = 0.0915
Final R indexes [all data]	R_1 = 0.0520, wR_2 = 0.0942
Largest diff. peak/hole / e Å^{-3}	1.648/-2.558

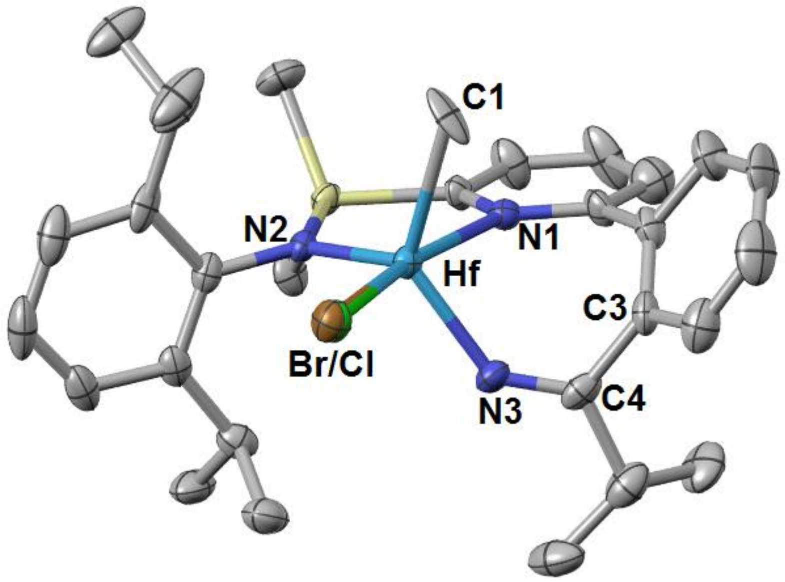

Figure S130. Solid state molecular structure of complex **1^{iPrCN}-HfMe(Cl/Br)** with ellipsoids drawn at 50% probability level. The hydrogen atoms are removed for clarity.

Table S3. Crystal data and structure refinement for **1-HfMe₂** (CCDC 2301574).

Empirical formula	$C_{27}H_{36}HfN_2Si$
Formula weight	595.16
Temperature/K	120(2)
Crystal system	triclinic
Space group	P-1
a/Å	9.6477(7)
b/Å	10.3442(8)
c/Å	15.3145(12)
α/°	88.4150(10)
β/°	72.4220(10)
γ/°	63.5670(10)
Volume/Å^3	1294.42(17)
Z	2
ρ_{calc}g/cm^3	1.527
μ/mm^{-1}	4.092
F(000)	596.0
Crystal size/mm^3	0.2 × 0.17 × 0.15
Radiation	MoKα (λ = 0.71073)
2Θ range for data collection/°	2.812 to 52.044
Index ranges	-11 ≤ h ≤ 11, -12 ≤ k ≤ 12, -18 ≤ l ≤ 18
Reflections collected	12591
Independent reflections	5091 [R_{int} = 0.0453, R_{sigma} = 0.0459]
Data/restraints/parameters	5091/0/288
Goodness-of-fit on F^2	1.028
Final R indexes [I>=2σ (I)]	R_1 = 0.0338, wR_2 = 0.0881
Final R indexes [all data]	R_1 = 0.0360, wR_2 = 0.0892
Largest diff. peak/hole / e Å^{-3}	2.99/-2.55

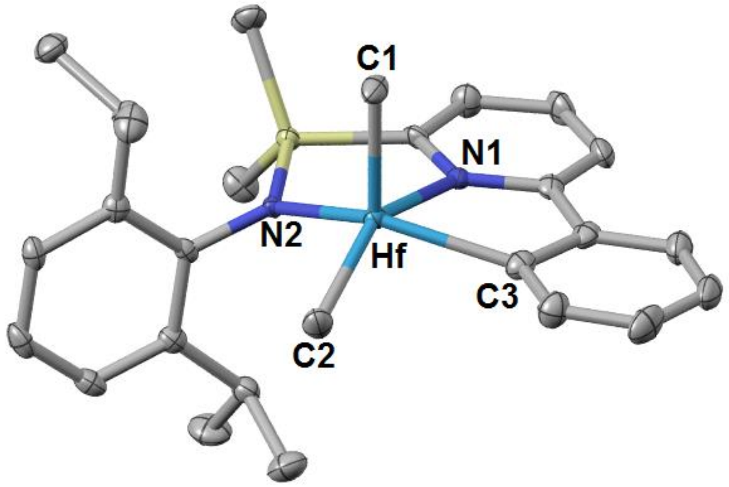

Figure S131. Solid state molecular structure of complex **1-HfMe₂** with ellipsoids drawn at 50% probability level. The hydrogen atoms are removed for clarity.

Table S4. Crystal data and structure refinement for **1**^{tBuCN}**-HfMe$_2$** (CCDC 2301577).

Empirical formula	$C_{32}H_{45}HfN_3Si$
Formula weight	678.29
Temperature/K	120(2)
Crystal system	monoclinic
Space group	P 1 2_1 1
a/Å	9.4880(3)
b/Å	38.1993(14)
c/Å	9.6016(3)
α/°	90
β/°	114.0880(10)
γ/°	90
Volume/Å^3	3176.92(18)
Z	4
ρ_{calc}g/cm^3	1.418
μ/mm^{-1}	3.345
F(000)	1376.0
Crystal size/mm^3	0.17 × 0.14 × 0.09
Radiation	MoKα (λ = 0.71073)
2Θ range for data collection/°	2.56 to 28.26
Index ranges	-12 ≤ h ≤ 12, -52 ≤ k ≤ 52, -13 ≤ l ≤ 13
Reflections collected	16654
Independent reflections	16654 [R_{int} = 0.0513, R_{sigma} = 0.0850]
Data/restraints/parameters	16654/1/678
Goodness-of-fit on F^2	1.032
Final R indexes [I>=2σ (I)]	R_1 = 0.0524, wR_2 = 0.0903
Final R indexes [all data]	R_1 = 0.0469, wR_2 = 0.0935
Largest diff. peak/hole / e Å^{-3}	1.614/-1.244

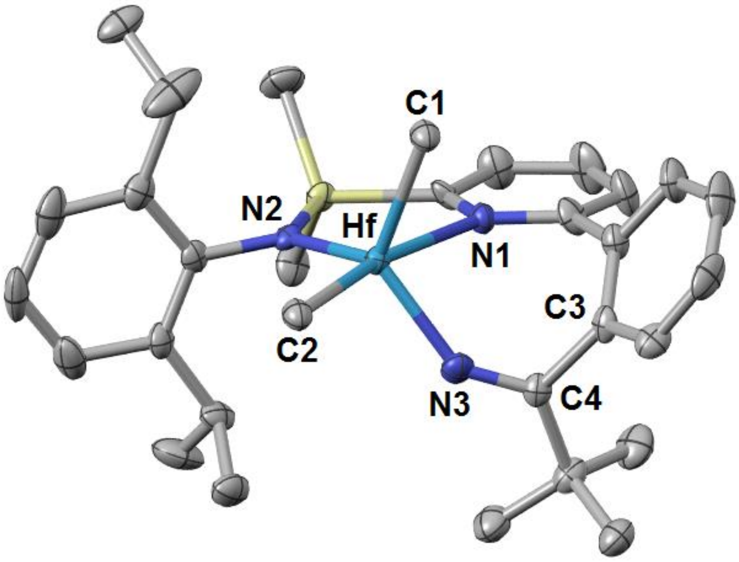

Figure S132. Solid state molecular structure of complex **1**^{tBuCN}**-HfMe$_2$** with ellipsoids drawn at 50% probability level. The hydrogen atoms are removed for clarity.

Table S5. Crystal data and structure refinement for **1-HfMe(N=CMe$_2$)** (CCDC 2301576).

Empirical formula	$C_{27}H_{36}HfN_2Si$
Formula weight	636.21
Temperature/K	112(2)
Crystal system	triclinic
Space group	P-1
a/Å	9.4363(4)
b/Å	9.4941(4)
c/Å	16.9652(7)
α/°	83.6590(10)
β/°	80.4910(10)
γ/°	70.2840(10)
Volume/Å^3	1408.68(10)
Z	2
ρ_{calc}g/cm^3	1.500
μ/mm^{-1}	3.766
F(000)	640.0
Crystal size/mm^3	0.32 × 0.15 × 0.12
Radiation	MoKα (λ = 0.71073)
2Θ range for data collection/°	2.314 to 28.498
Index ranges	-12 ≤ h ≤ 12, -12 ≤ k ≤ 12, -22 ≤ l ≤ 22
Reflections collected	21015
Independent reflections	7091 [R_{int} = 0.0398, R_{sigma} = 0.0472]
Data/restraints/parameters	7091/0/319
Goodness-of-fit on F^2	1.013
Final R indexes [I>=2σ (I)]	R_1 = 0.0301, wR_2 = 0.0553
Final R indexes [all data]	R_1 = 0.0369, wR_2 = 0.0572
Largest diff. peak/hole / e Å^{-3}	2.627/-1.192

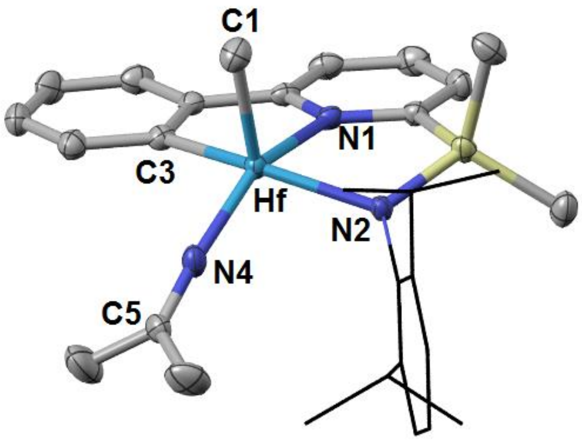

Figure S133. Solid state molecular structure of complex **1-HfMe(N=CMe$_2$)** with ellipsoids drawn at 50% probability level. The hydrogen atoms are removed for clarity.

Table S6. Crystal data and structure refinement for **1**^{iPrCN}**-HfMe(N=CMe*i*Pr)** (CCDC 2301580).

Empirical formula	$C_{35}H_{50}HfN_4Si$
Formula weight	733.37
Temperature/K	120(2)
Crystal system	orthorhombic
Space group	Pbca
a/Å	16.1939(14)
b/Å	15.9751(10)
c/Å	27.1492(16)
α/°	90
β/°	90
γ/°	90
Volume/Å^3	7023.5(9)
Z	8
ρ_{calc}g/cm^3	1.387
μ/mm^{-1}	3.032
F(000)	2992.0
Crystal size/mm^3	0.146 × 0.139 × 0.122
Radiation	MoKα (λ = 0.71073)
2Θ range for data collection/°	1.941 to 28.999
Index ranges	-22 ≤ h ≤ 22, -20 ≤ k ≤ 21, -35 ≤ l ≤ 37
Reflections collected	67522
Independent reflections	9319 [R_{int} = 0.1257, R_{sigma} = 0.0707]
Data/restraints/parameters	9319/0/382
Goodness-of-fit on F^2	1.036
Final R indexes [I>=2σ (I)]	R_1 = 0.0431, wR_2 = 0.0846
Final R indexes [all data]	R_1 = 0.0661, wR_2 = 0.0978
Largest diff. peak/hole / e Å^{-3}	1.902/-1.151

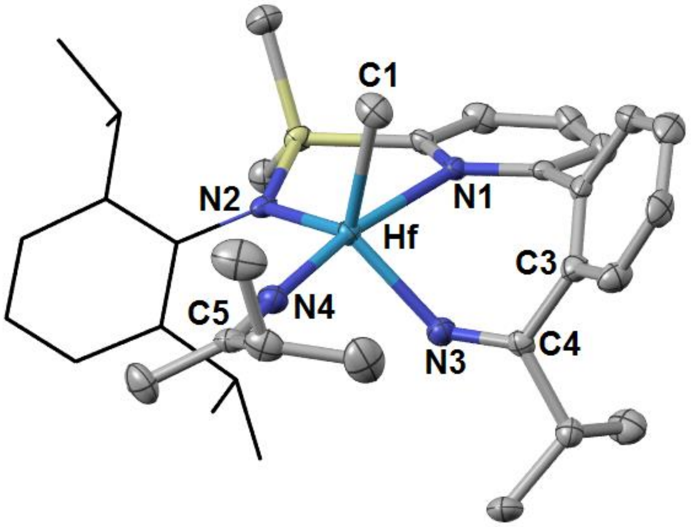

Figure S134. Solid state molecular structure of complex **1**^{iPrCN}**-HfMe(N=CMe*i*Pr)** with ellipsoids drawn at 50% probability level. The hydrogen atoms are removed for clarity.

Table S7. Crystal data and structure refinement for **1^{PhCN}-HfMe(N=CMePh)** (CCDC 2301579).

Empirical formula	$C_{41}H_{46}HfN_4Si$
Formula weight	801.40
Temperature/K	120(2)
Crystal system	trigonal
Space group	R$\bar{3}$
a/Å	44.5128(8)
b/Å	44.5128(8)
c/Å	9.3817(3)
α/°	90
β/°	90
γ/°	120
Volume/Å^3	16098.4(8)
Z	18
ρ_{calc}g/cm^3	1.488
μ/mm^{-1}	2.984
F(000)	7308.0
Crystal size/mm^3	0.16 × 0.13 × 0.12
Radiation	MoKα (λ = 0.71073)
2Θ range for data collection/°	1.830 to 28.995
Index ranges	$-60 \leq h \leq 58$, $-59 \leq k \leq 60$, $-12 \leq l \leq 12$
Reflections collected	53933
Independent reflections	9491 [R_{int} = 0.0882, R_{sigma} = 0.0600]
Data/restraints/parameters	9491/0/432
Goodness-of-fit on F^2	1.025
Final R indexes [I>=2σ (I)]	R_1 = 0.0406, wR_2 = 0.0814
Final R indexes [all data]	R_1 = 0.0530, wR_2 = 0.0872
Largest diff. peak/hole / e Å^{-3}	1.179/-1.007

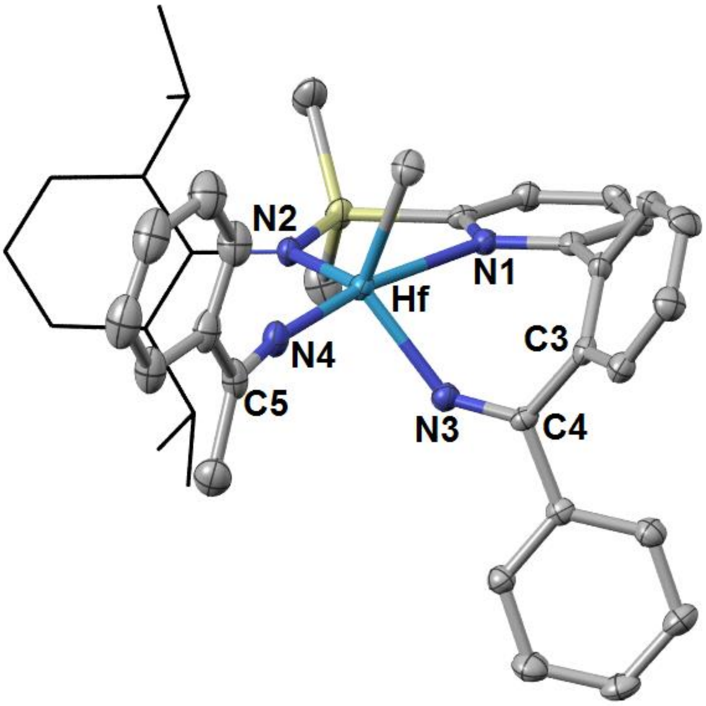

Figure S135. Solid state molecular structure of complex **1^{PhCN}-HfMe(N=CMePh)** with ellipsoids drawn at 50% probability level. The hydrogen atoms are removed for clarity.

Table S8. Crystal data and structure refinement for **2-Zr(N=CMe*i*Pr)$_2$** (2301575).

Empirical formula	$C_{36}H_{50}N_4Zr$
Formula weight	630.02
Temperature/K	120(2)
Crystal system	triclinic
Space group	P$\bar{1}$
a/Å	10.4428(7)
b/Å	12.0367(10)
c/Å	15.1051(10)
α/°	84.604(4)
β/°	76.557(3)
γ/°	65.977(4)
Volume/Å^3	1686.7(2)
Z	2
ρ_{calc}g/cm^3	1.240
μ/mm^{-1}	0.355
F(000)	668.0
Crystal size/mm^3	0.135 × 0.118 × 0.109
Radiation	MoKα (λ = 0.71073)
2Θ range for data collection/°	2.19 to 25.30
Index ranges	-12 ≤ h ≤ 12, -14 ≤ k ≤ 14, -18 ≤ l ≤ 18
Reflections collected	13141
Independent reflections	6606 [R_{int} = 0.0613, R_{sigma} = 0.0928]
Data/restraints/parameters	6606/53/418
Goodness-of-fit on F^2	1.051
Final R indexes [I>=2σ (I)]	R_1 = 0.0611, wR_2 = 0.1401
Final R indexes [all data]	R_1 = 0.0744, wR_2 = 0.1538
Largest diff. peak/hole / e Å^{-3}	1.334/-0.556

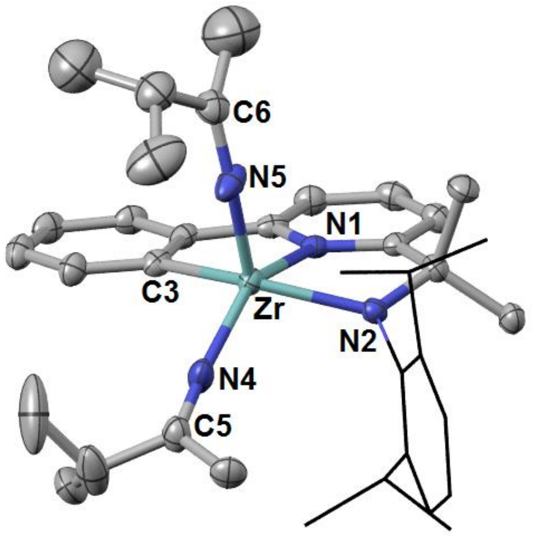

Figure S136. Solid state molecular structure of complex **2-Zr(N=CMe*i*Pr)$_2$**with ellipsoids drawn at 50% probability level. The hydrogen atoms are removed for clarity.

Table S9. Selected Bond Distances (Å) and Angles (deg) for **1-HfMe₂**, **1^{iPrCN}-HfMe(Cl/Br)**, **1^{tBuCN}-HfMe$_2$**, **1-HfMe(N=CMe$_2$)**, **1^{iPrCN}-HfMe(N=CMe*i*Pr)**, **1^{PhCN}-HfMe(N=CMePh)** and **2-Zr(N=CMe*i*Pr)$_2$**.

Metric	1-HfMe$_2$	1^{iPrCN}-HfMe(Cl/Br)	1^{tBuCN}-HfMe$_2$	1-HfMe (N=CMe$_2$)	1^{iPrCN}-HfMe (N=CMe*i*Pr)	1^{PhCN}-HfMe (N=CMePh)	2-Zr (N=CMe*i*Pr)$_2$
CCDC	2301574	2301578	2301577	2301576	2301580	2301579	2301575
d [Hf–C1]	2.204(5)	2.320(11)	2.221(10)	2.208(4)	2.249(5)	2.248(4)	—
d [Hf–C2]	2.242(5)	—	2.286(9)	—	—	—	—
d [M[a]–C3]	2.271(5)	—	—	2.285(3)	—	—	2.316(4)
d [M[a]–N1]	2.328(4)	2.335(8)	2.434(7)	2.362(3)	2.404(4)	2.421(3)	2.341(3)
d [M[a]–N2]	2.105(4)	2.054(9)	2.090(9)	2.114(2)	2.097(3)	2.083(3)	2.114(3)
d [Hf–N3]	—	2.034(8)	2.034(9)	—	2.053(4)	2.068(3)	—
d [M[a]–N4]/ *d* [M[a]–N5]	—	—	—	1.979(3)	1.997(4)	1.998(3)	2.023(4) /1.999(3)
d [C3–C4]	—	1.496(15)	1.526(14)	—	1.510(6)	1.508(5)	—
d [N3–C4]	—	1.273(13)	1.250(12)	—	1.267(6)	1.267(5)	—
d [N4–C5]/ *d* [N5–C6]	—	—	—	1.259(4)	1.257(6)	1.272(5)	1.262(5) /1.247(10)
∠ [Hf–N3–C4]	—	134.5(8)	142.0(8)	—	136.3(3)	138.9(3)	—
∠ [M[a]–N4(N5)–C5(C6)]	—	—	—	169.8(3)	174.8(4)	168.6(3)	173.3(3) /168.4(6)
∠ [N3–C4–C3]	—	120.0(9)	116.5(9)	—	120.2(4)	119.9(3)	—
∠ Dihedral [Py]-[Ph]	0.84	55.95	56.12	2.46	63.08	60.27	1.74

[a] M = Hf for **1-HfMe$_2$**, **1^{iPrCN}-HfMe(Cl/Br)**, **1^{tBuCN}-HfMe$_2$**, **1-HfMe(N=CMe$_2$)**, **1^{iPrCN}-HfMe(N=CMe*i*Pr)**, **1^{PhCN}-HfMe(N=CMePh)** and M = Zr for **2-Zr(N=CMe*i*Pr)$_2$**.

8. Computational Details

Geometries were fully optimized using the Gaussian 16 software package [11]. Data collection was done with Bopt [12]. All relevant minima and transition states were fully optimized in the gas phase at the TPSSh [13] level of theory employing correlation-consistent polarized valence double-ζ Dunning (DZ) basis sets with cc-pVDZ [14] quality from the EMSL basis set exchange library [15], using a small core pseudopotential on all metals [16]. All calculations were performed using standard Gaussian 16 SCF convergence criteria and the density fitting approximation (resolution of identity, RI) [17–20]. The nature of each stationary point was checked with an analytical second-derivative calculation (no imaginary frequency for minima, exactly one imaginary frequency for transition states, corresponding to the reaction coordinate). Transition states were located using a relaxed potential energy scan to arrive at a suitable transition-state guess, followed by optimization using the Gaussian 16 Berny algorithm (opt=ts). Single-point energies (SPE) were calculated by employing triple-Dunning (TZ) basis sets (cc-pVTZ quality) while retaining the TPSSh functional. Solvent effects (toluene, ε = 2.38) with the Solvent Model based on Density (SMD) [21] were included in the single point calculations. Enthalpies and Gibbs free energies were then obtained from TZ single-point energies and thermal corrections from the TPSS/cc-pVDZ-(PP) vibrational analyses (at 298 K and 1 atm). Intrinsic reaction coordinate calculations were performed with Gaussian 16. Intrinsic reaction coordinate calculations were performed with Gaussian 16; both endpoints were then fully optimized. IboView v20211019-RevA was employed for the IBO calculations and visualization employing SP Molden output files from Gaussian (utilizing the following input: *#p gfinput IOP(6/7=3) tpssh/def2TZVP/auto pseudo=read denfit int=grid=ultrafine*) [7–9]. NBO analysis was performed with the 3.1 version integrated in Gaussian [22].

Animated gifs illustrating intrinsic bond orbitals changes along the reaction coordinate for *i*PrCN insertion into **1**-$HfCl_2$ and **1**-$HfMe_2$ can be found at the journal website as separate .gif files:

d4sc02173h1.gif – *i*PrCN insertion into Hf-C_{Ar} in **1**-$HfCl_2$

d4sc02173h2.gif – *i*PrCN insertion into Hf-C_{Ar} in **1**-$HfCl_2$, π-system of the Ar

d4sc02173h3.gif – *i*PrCN insertion into Hf-Me in **1**-$HfMe_2$

d4sc02173h4.gif – *i*PrCN insertion into Hf-C_{Ar} in **1**-$HfMe_2$, π-system of the Ar

d4sc02173h4.gif – *i*PrCN insertion into Hf-C_{Ar} in **1**-$HfMe_2$

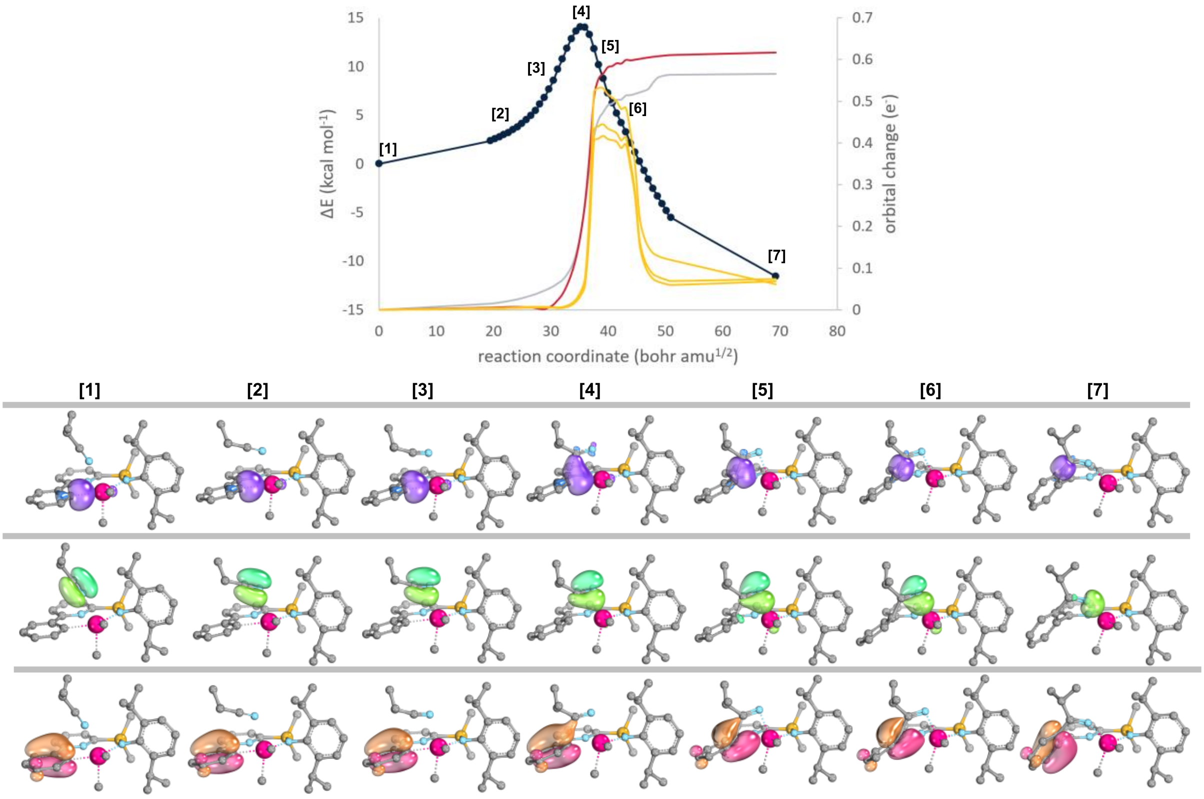

Figure S137. Top: Plot of the root of the sum of square deviations (RSSD) of the partial charge distribution changes along the IRC for the *i*PrCN in the Hf–C_{Ar} bond leading to **1**^{iPrCN}**-HfMe$_2$** via **TS-1**^{iPrCN}**-HfMe$_2$**. Hf–C_{Ar} (red), the C≡N π (grey) and the three aryl based π IBOs. Bottom, Depiction of the Hf–C_{Ar} (purple, top), C≡N π (green, middle), and one selected aryl π IBO (orange-red) along the IRC. H-atoms omitted for clarity.

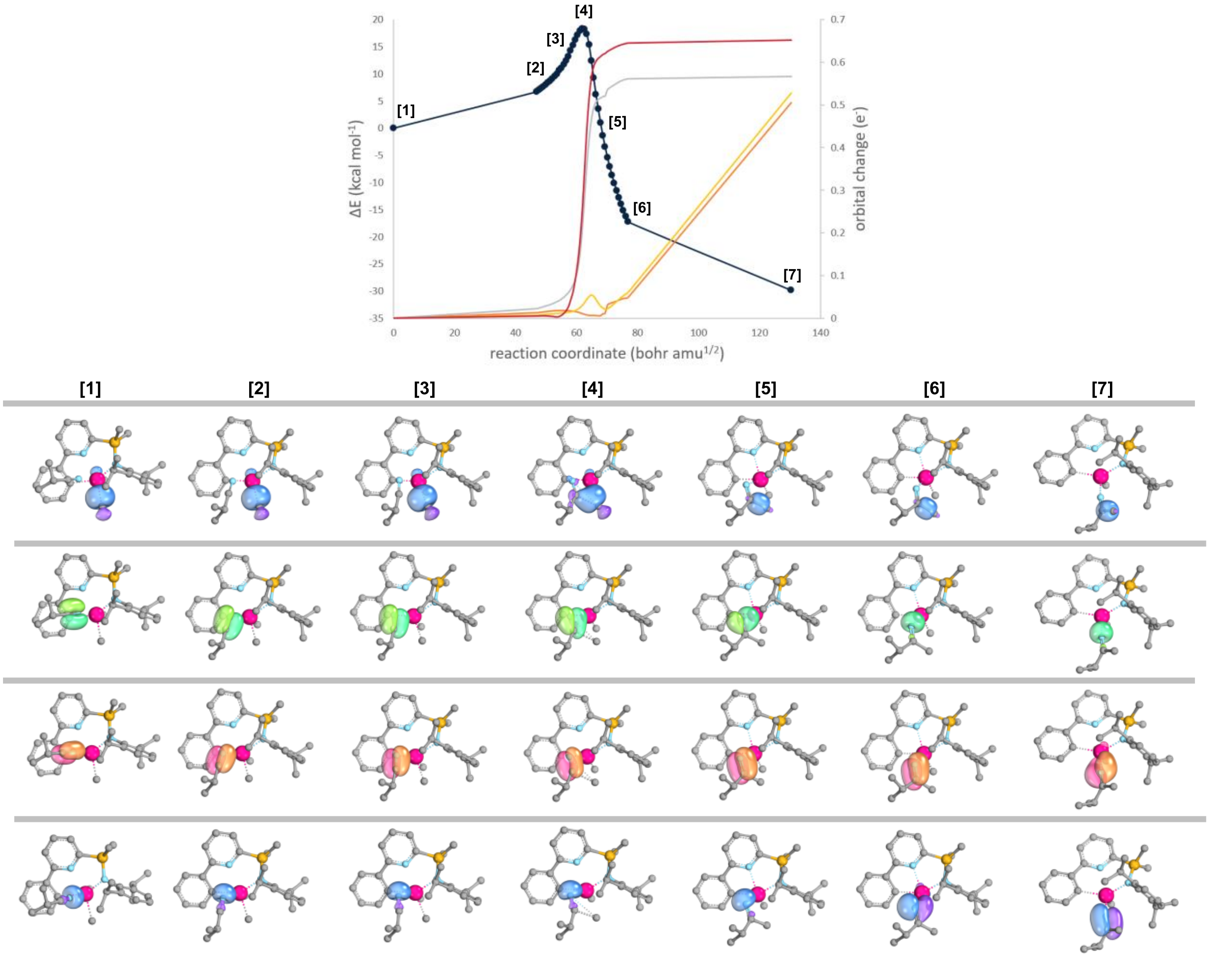

Figure S138. Top: Plot of the root of the sum of square deviations (RSSD) of the partial charge distribution changes along the IRC for the *i*PrCN in the Hf–CH_3 bond leading to **1-HfMe(N=CMe*i*Pr)** via **TS-1-HfMe(N=CMe*i*Pr)**. Hf–CH_3 (red, top), the two C≡N π (grey, yellow) and the C≡N σ (orange) IBOs. Bottom, Depiction of the Hf–CH_3 (purple, top), C≡N π (green and orange, middle), and C≡N σ IBO (blue, bottom) along the IRC. H-atoms omitted for clarity.

Table S10. System **1-HfMe$_2$**. Final Energies, Enthalpies and Gibbs free energies. In Hartree. Differences in kcal mol^{-1}.

Name	Formula	Energy (TPSSh/DZ)	Energy (TPSSh(SMD)TZ)	D0	ZPE	EnthalpyCorr (p=1.0, t=298)	EntropyCorr (p=1.0, t=298)	E	H	G	E	H	G	ΔE	ΔH	ΔG
						with Dispersion										
***i* PrCN**	C4H7N	-211.4084397	-211.4740847	-0.00528	0.101902	0.10885725	0.035715238	-211.377	-211.370512	-211.406227						
**1-HfMe$_2$**	C27H36HfN2Si	-1498.492875	-1498.842589	-0.07587	0.551686	0.589536562	0.107927107	-1498.37	-1498.32892	-1498.43685	-1709.74	-1709.7	-1709.84	0.0	0.0	0.0
**1-HfMe$_2$-*i* PrCN adduct**	C31H43HfN3Si	-1709.912184	-1710.319074	-0.09307	0.655406	0.700426924	0.123097817	-1709.76	-1709.71172	-1709.83482	-1709.76	-1709.71	-1709.83	-7.8	-7.7	5.2
**TS-1^{iPrCN}-HfMe$_2$**	C31H43HfN3Si	-1709.891705	-1710.292855	-0.09808	0.655723	0.699325164	0.11747094	-1709.74	-1709.69161	-1709.80909	-1709.74	-1709.69	-1709.81	5.7	4.9	21.3
**TS-1-HfMe(N=CMe*i*Pr)**	C31H43HfN3Si	-1709.88489	-1710.287088	-0.09664	0.656937	0.700226619	0.117283756	-1709.73	-1709.6835	-1709.80079	-1709.73	-1709.68	-1709.8	10.9	10.0	26.5
**1^{iPrCN}-HfMe$_2$**	C31H43HfN3Si	-1709.932468	-1710.337823	-0.09432	0.657308	0.701343746	0.120357904	-1709.77	-1709.7308	-1709.85116	-1709.77	-1709.73	-1709.85	-19.2	-19.7	-5.1
**1-HfMe(N=CMe*i*Pr)**	C31H43HfN3Si	-1709.961994	-1710.364581	-0.09258	0.659766	0.703742634	0.12138006	-1709.8	-1709.75342	-1709.8748	-1709.8	-1709.75	-1709.87	-33.4	-33.9	-19.9

Table S11. System **1-HfCl$_2$**. Final Energies, Enthalpies and Gibbs free energies. In Hartree. Differences in kcal mol^{-1}.

Name	Formula	Energy (TPSSh/DZ)	Energy (TPSSh(SMD)TZ)	D0	ZPE	EnthalpyCorr (p=1.0, t=298)	EntropyCorr (p=1.0, t=298)	E	H	G	E	H	G	ΔE	ΔH	ΔG
***i* PrCN**	C4H7N	-211.4084397	-211.467766	-0.00528	0.101902	0.108862645	0.035738612	-211.371	-211.364188	-211.399926						
**1^{iPrCN}-HfCl$_2$**	C29H37Cl2HfN3Si	-2550.724659	-2551.098125	-0.09231	0.592773	0.634540646	0.116227371	-2550.6	-2550.55589	-2550.67212	-2550.6	-2550.56	-2550.67	0	0	0
**TS-1^{iPrCN}-HfCl$_2$**	C29H37Cl2HfN3Si	-2550.684913	-2551.058636	-0.09585	0.59068	0.632360855	0.11484356	-2550.56	-2550.52213	-2550.63697	-2550.56	-2550.52	-2550.64	21.2	21.2	22.1
**1-HfCl$_2$_*i* PrCN_coord**	C29H37Cl2HfN3Si	-2550.707426	-2551.082751	-0.09188	0.591006	0.633762897	0.118865955	-2550.58	-2550.54087	-2550.65974	-2550.58	-2550.54	-2550.66	8.8	9.4	7.8
**1-HfCl$_2$_*i* PrCN_coordTS**	C29H37Cl2HfN3Si	-2550.691065	-2551.067587	-0.09092	0.59005	0.632734726	0.121599635	-2550.57	-2550.52578	-2550.64738	-2550.57	-2550.53	-2550.65	18.3	18.9	15.5
**1-HfCl$_2$**	C25H30Cl2HfN2Si	-2339.281019	-2339.600544	-0.07465	0.487137	0.522724406	0.103285025	-2339.19	-2339.15247	-2339.25576	-2550.56	-2550.52	-2550.66	24.1	24.6	10.3

Table S12. Distortion energies. In Hartree. Differences in kcal mol^{-1}.

	Fragment	Fragment Energy	Relaxed Energy	ΔE	Σ(ΔE)
**TS-1^{iPrCN}-HfMe$_2$**	*i* PrCN	-211.4510224	-211.4740847	14.5	27.9
	Hf	-1498.821115	-1498.842589	13.5	
**TS-1-HfMe(N=CMe*i*Pr)**	*i* PrCN	-211.4528499	-211.4740847	13.3	32.0
	Hf	-1498.812894	-1498.842589	18.6	

Table S13. TS analysis. Distances in Å.

	C-C	Hf-C_{Me}	Hf-C_{Ar}	Hf-N	C=N
**1-HfMe$_2$**	3.476	2.2544	2.3223	2.4379	1.1621
**TS-1^{iPrCN}-HfMe$_2$**	2.1121	n/a	2.4389	2.2581	1.206
**TS-1-HfMe(N=CMe*i*Pr)**	2.2061	2.4093	n/a	2.232	1.207

9. Kinetic curves for the *i*PrCN extrusion from $\mathbf{1}^{iPrCN}$-$HfMe_2$ at different concentrations

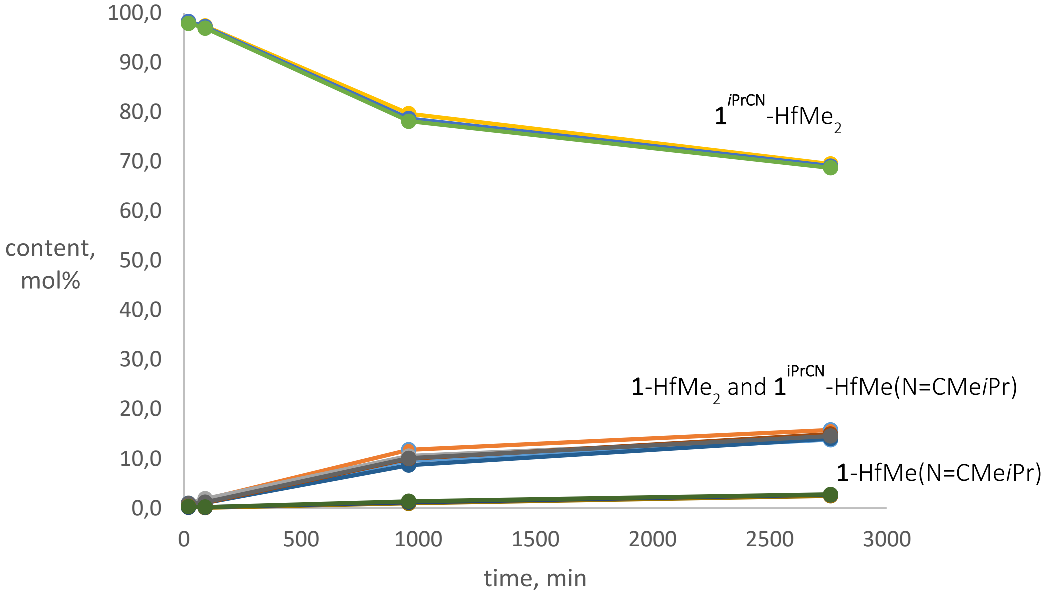

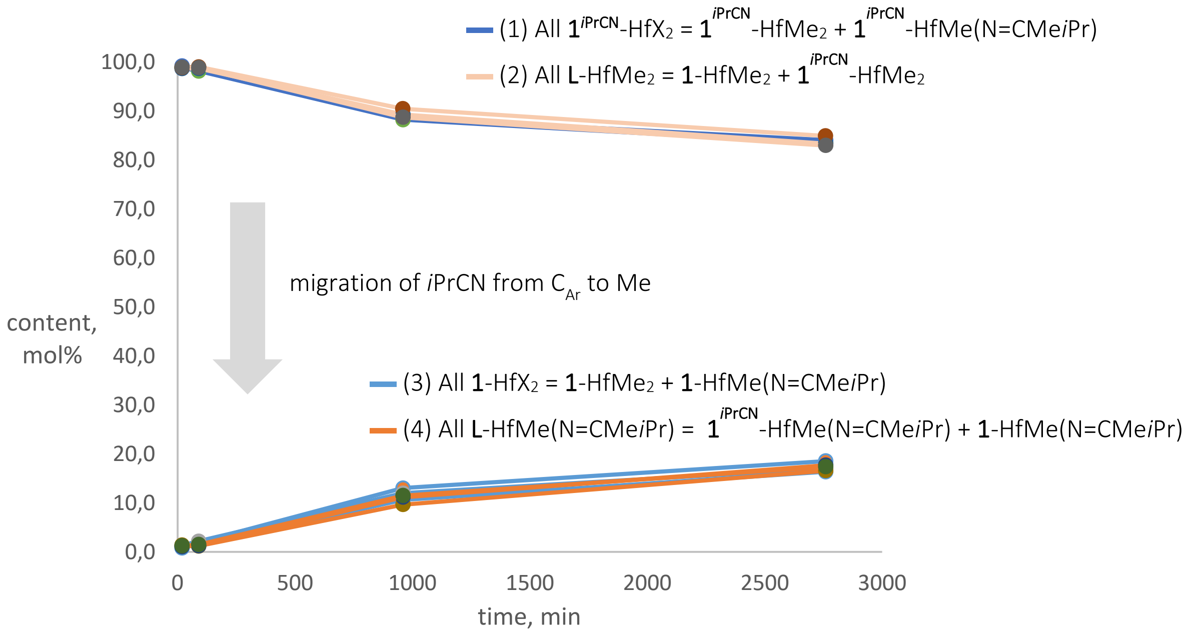

Figure S139. Identical kinetic curves for the *i*PrCN extrusion from $\mathbf{1}^{iPrCN}$-$HfMe_2$ and reinsertion into Hf-Me at different concentrations (5, 10 and 15 mg $\mathbf{1}^{iPrCN}$-$HfMe_2$, 0.6 mL toluene-d_8, J. Young NMR tube, room temperature). Top: with relative concentrations of individual components of the mixture (12 curves in total). Bottom: with sums of concentrations of all complexes containing the following moieties: (1) *i*PrCN inserted into Hf-C_{Ar} bond (i.e. $\mathbf{1}^{iPrCN}$-ligand) (2) $HfMe_2$, (3) Hf-C_{Ar} (i.e. **1**-ligand), (4) *i*PrCN inserted into Hf-Me bond (i.e. ketimide (N=CMe*i*Pr) ligand).

10. References

[1] P. S. Kulyabin, D. V. Uborsky, A. Z. Voskoboynikov, J. A. M. Canich, J. R. Hagadorn, *Dalton Trans.* **2020**, *49*, 6693-6702.

[2] G. J. Domski, J. M. Eagan, C. D. Rosa, R. D. Girolamo, A. M. LaPointe, E. B. Lobkovsky, G. Talarico, G. W. Coates, *ACS Catal.* **2017**, *7*, 6930–6937.

[3] G. J. Domski, E. B. Lobkovsky, G. W. Coates, *Macromolecules* **2007**, *40*, 3510–3513.

[4] M. T. Quirós, J. Angulo, M. P. Muñoz, *Chem. Commun.* **2015**, *51*, 10222–10225.

[5] J. Légaré Lavergne, A. Jayaraman, L. C. Misal Castro, É. Rochette, F.-G. Fontaine, *J. Am. Chem. Soc.* **2017**, *139*, 14714–14723.

[6] S. Liu, M. P. Conley, R. R. Schrock, *Organometallics* **2023**, *42*, 1087–1093.

[7] P. M. Morse, M. D. Spencer, S. R. Wilson, G. S. Girolami, *Organometallics* **1994**, *13*, 1646–1655.

[8] SMART and SAINT, Release 5.0, Area Detector control and Integration Software, Bruker AXS, Analytical X-Ray Instruments, Madison, Wisconsin, USA, **1998**.

[9] G. M. Sheldrick, SADABS: A Program for Exploiting the Redundancy of Area-detector X-Ray Data, University of Göttingen, Göttingen, Germany, **1999**.

[10] G. M. Sheldrick, SHELXL-97 Program for Solution and Refinement of Crystal Structure, Bruker AXS Inc., Madison, WI-53719, USA, **1997**.

[11] M. J. Frisch, G. W. Trucks, H. B. Schlegel, G. E. Scuseria, M. A. Robb, J. R. Cheeseman, G. Scalmani, V. Barone, G. A. Petersson, H. Nakatsuji, X. Li, M. Caricato, A. V. Marenich, J. Bloino, B. G. Janesko, R. Gomperts, B. Mennucci, H. P. Hratchian, J. V. Ortiz, A. F. Izmaylov, J. L. Sonnenberg, D. Williams-Young, F. Ding, F. Lipparini, F. Egidi, J. Goings, B. Peng, A. Petrone, T. Henderson, D. Ranasinghe, V. G. Zakrzewski, J. Gao, N. Rega, G. Zheng, W. Liang, M. Hada, M. Ehara, K. Toyota, R. Fukuda, J. Hasegawa, M. Ishida, T. Nakajima, Y. Honda, O. Kitao, H. Nakai, T. Vreven, K. Throssell, J. A. Montgomery, Jr., J. E. Peralta, F. Ogliaro, M. J. Bearpark, J. J. Heyd, E. N. Brothers, K. N. Kudin, V. N. Staroverov, T. A. Keith, R. Kobayashi, J. Normand, K. Raghavachari, A. P. Rendell, J. C. Burant, S. S. Iyengar, J. Tomasi, M. Cossi, J. M. Millam, M. Klene, C. Adamo, R. Cammi, J. W. Ochterski, R. L. Martin, K. Morokuma, O. Farkas, J. B. Foresman, and D. J. Fox, Gaussian 16, revision A.03; Gaussian, Inc.: Wallingford, CT, **2016**.

[12] P. H. M. Budzelaar, *J. Comput. Chem.* **2007**, 28 (13), 2226-2236.

[13] J. M. Tao, J. P. Perdew, V. N. Staroverov, G. E. Scuseria, *Phys. Rev. Lett.* **2003**, *91*, 146401.

[14] D. Figgen, K. A. Peterson, M. Dolg, H. Stoll, *J. Chem. Phys.* **2009**, *130*, 164108.

[15] B. P. Pritchard, D. Altarawy, B. Didier, T. D. Gibson, T. L. Windus, *J. Chem. Inf. Model.* **2019**, *59*, 4814-4820.

[16] P. Schwerdtfeger, *ChemPhysChem* **2011**, *12*, 3143-3155.

[17] J. L. Whitten, *J. Chem. Phys.* **1973**, *58*, 4496-4501.

[18] E. J. Baerends, D. E. Ellis, P. Ros, *Chem. Phys.* **1973**, *2*, 41–51.

[19] M. Feyereisen, G. Fitzgerald, A. Komornicki, *Chem. Phys. Lett.* **1993**, *208*, 359–363.

[20] O. Vahtras, J. Almlöf, M. W. Feyereisen, *Chem. Phys. Lett.* **1993**, *213*, 514–518.

[21] A. V. Marenich, C. J. Cramer, D. G. Truhlar, *J. Phys. Chem. B* **2009**, *113*, 6378–6396.

[22] E. D. Glendening, A. E. Reed, J. E. Carpenter, F. Weinhold, NBO Version 3.1; University of Wisconsin: Madison, WI, USA, **1998**.
